# Supplementary material for: Shedding light on the nature of the catalytically active species in photocatalytic reactions using Bi2O3 semiconductor
Source: Nat Commun. 2021 Jan 27;12:625. doi: 10.1038/s41467-020-20882-x (PMC7841156; doi:10.1038/s41467-020-20882-x)
Supplement: Supplementary file 1 — Supplementary Information [file 41467_2020_20882_MOESM1_ESM.pdf]

Shedding light on the nature of the catalytically active species in photocatalytic reactions using Bi_2O_3 semiconductor

Paola Riente,[1*] Mauro Fianchini,[2] Patricia Llanes,[2] Miquel A. Pericàs[2, 3] and Timothy Noël[1,4*]

[1] Department of Chemical Engineering and Chemistry, Micro Flow Chemistry and Synthetic Methodology, Eindhoven University of Technology, Eindhoven, The Netherlands.

[4] Current address: Flow Chemistry Group, van 't Hoff Institute for Molecular Sciences (HIMS), University of Amsterdam (UvA), Science Park 904, 1098 XH, Amsterdam, The Netherlands.

E-mail: p.riente.paiva@tue.nl, t.noel@uva.nl

[2] Institute of Chemical Research of Catalonia (ICIQ), The Barcelona Institute of Science and Technology (BIST), E-43007 Tarragona, Spain

E-mail: mfianchini@iciq.es, pllanes@iciq.es, mapericas@iciq.es

[3] Departament de Quimica Inorgànica i Orgànica, Universitat de Barcelona, 08028-Barcelona, Spain

Supplementary Information

Contents

1. General experimental 3

1.1. Reactor set up 3

2. UV-vis spectra measurements 4

2.1. ATRA reaction and diethyl bromomalonate (DEBM) experiment in the absence of Bi_2O_3 4

2.2. UV-vis spectra of the organic substrates 6

2.3. Bi_2O_3 light-driven ATRA reaction in different solvents 7

2.4. Control experiments in the presence of trimethyl sulfonium bromide 8

3. GC-MS 9

4. Crystal data, data collection and structure refinement parameters 12

4.1. Single Crystal X-Ray Methods 12

5. Computational methods 15

5.1. Scoring computation *vs.* experiment 16

5.2. Chelation of diethyl bromomalonate onto Bi^{3+}: formation of Bi^{3+}•••O bonds 17

6. Supplementary references 49

1. General experimental

Unless otherwise stated, all starting materials were commercially available in the best grade and were used without further purification. Reagents and solvents were purchased from Sigma Aldrich and TCI and, when needed, stored under an argon atmosphere. Technical solvents were purchased from VWR International and Biosolve and were used as received. NMR spectra were recorded at room temperature on a Bruker Advance 400, operating at 400 MHz (^{1}H) and 100 MHz (^{13}C). UV-vis measurements were carried out on a Shimadzu UV-2501PC spectrophotometer equipped with a photomultiplier detector, double beam optics, and D2 and W light source. The product of the ATRA reaction (diethyl 2-(2-bromo-6-hydroxyhexyl)malonate, **A**) derived from the reaction between 5-hexen-ol and diethyl bromomalonate is known and it was characterized by a comparison of its physical and spectroscopic properties described in the literature.[1] Mass spectra were performed using a GC-MS combination (Shimadzu GC-2010 Plus coupled to a Mass spectrometer, Shimadzu GCMS-QP2010 Ultra) with an auto sample unit (AOC-20i Shimadzu).

1.1. Reactor set up

The vial containing the reaction mixture equipped with septum was loaded with the reagents and placed inside a homemade 3D-printed reactor equipped with white LED strippers (~23 W). The cooling of the system was granted by a stream of compressed air inserted at the bottom of the reactor holder.

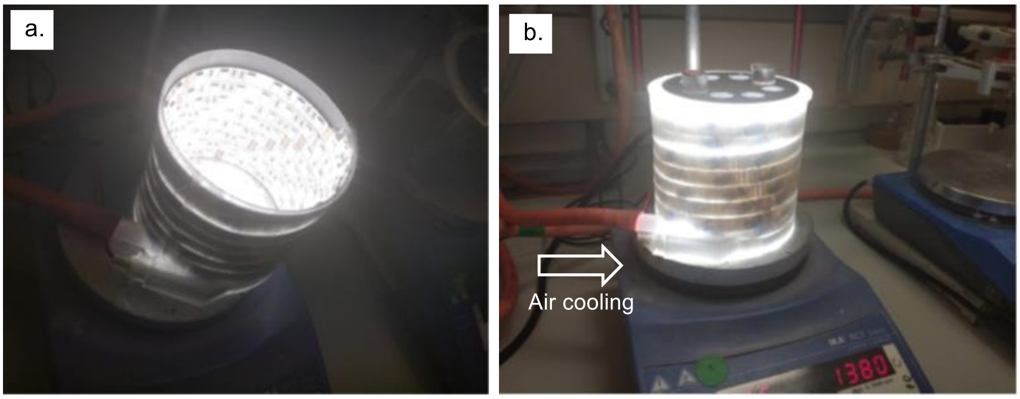

Supplementary Figure 1| Pictures of the photomicroreactor employed for the ATRA reaction. a, White LED strip placed in the beaker of the reactor holder. **b,** View of the photochemical reactor during operation.

2. UV-vis spectra measurements

Samples were collected with a syringe and passed through a Millipore filter (0.22 μm), placed in a quartz cuvette (1 cm), and diluted with the appropriated solvent before the UV-vis analysis.

2.1. ATRA reaction and diethyl bromomalonate (DEBM) experiment in the absence of Bi_2O_3

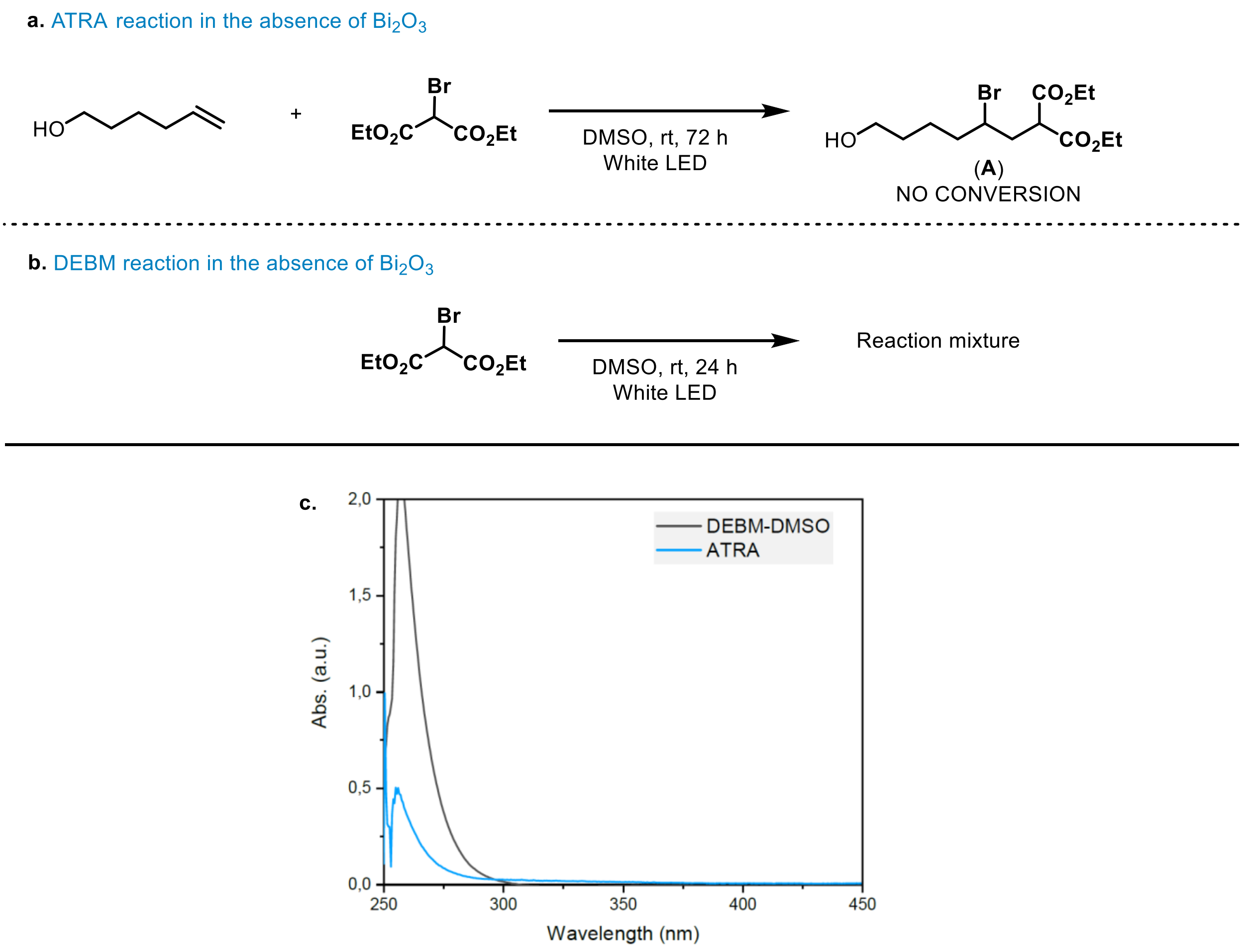

Supplementary Figure 2| ATRA reaction and DEBM experiment in the absence of Bi_2O_3. **a,** ATRA reaction carried out in the absence of Bi_2O_3 under irradiation. **b,** DEBM experiment carried out in the absence of Bi_2O_3 under irradiation. **c,** UV-vis spectra of the ATRA reaction (72 h) and diethyl bromomalonate (24 h) in DMSO under irradiation (23 W with white LED) in the absence of Bi_2O_3. The concentration of diethyl bromomalonate in both analyses is 6.7 mM.

2.2. UV-vis spectra of the organic substrates

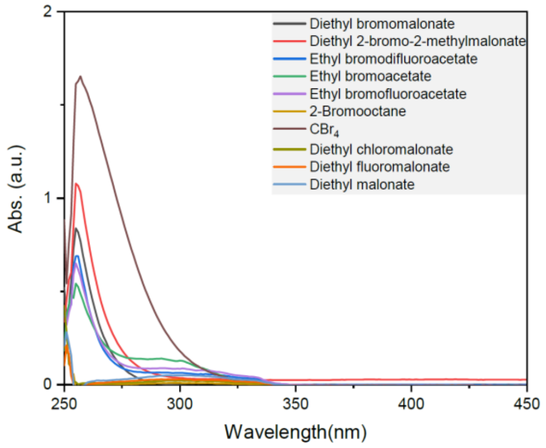

Supplementary Figure 3| UV-vis spectra of the organic substrates in DMSO. The substrates concentrations are 6.7 mM except for CBr_4 for which the measurement was performed at a concentration of 0.83 mM.

2.3. Bi_2O_3 light-driven ATRA reaction in different solvents

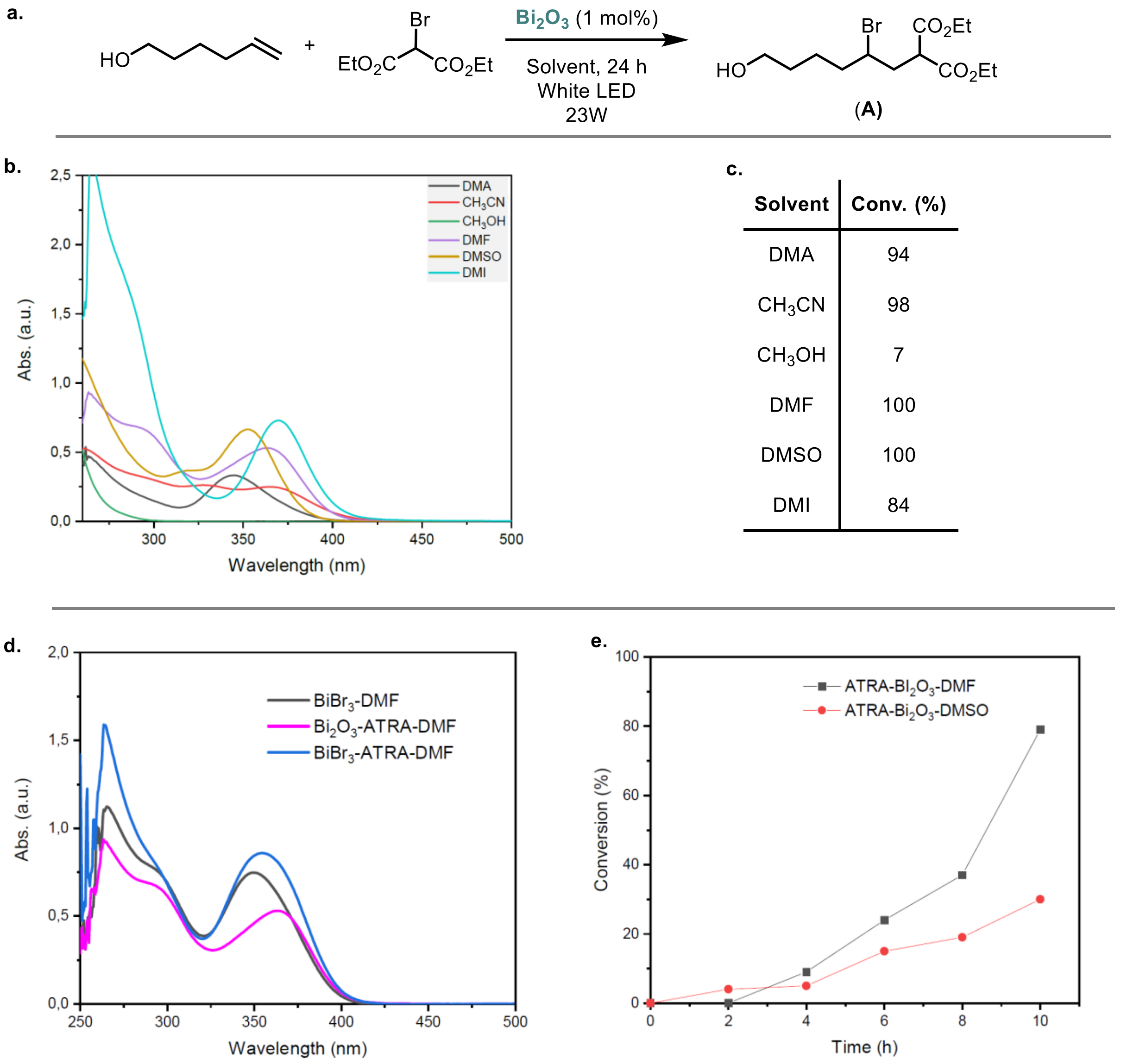

Supplementary Figure 4| Bi_2O_3 light-driven ATRA reaction in different solvents. a, ATRA reaction. **b**, UV-vis spectra of the ATRA reaction in different solvents. The DEBM concentration is 6.7 mM. **c,** Conversion measured by ^{1}H NMR of the ATRA reaction in different solvents. *Reaction conditions*: Diethyl bromomalonate (1 mmol), 5-hexen-1-ol (1.1 mmol), Bi_2O_3 (1 mol%), solvent (2 mL), light source (white LED ~23 W). Dimethylacetamide (DMA) and 1,3-dimethyl-2-imidazolidinone (DMI). **d,** UV-vis spectra of the ATRA reaction in DMF

photocatalyzed by Bi_2O_3 and $BiBr_3$. **e**. Kinetic study of the ATRA reaction photocatalyzed by Bi_2O_3 in DMF and DMSO. Conversions were obtained by ^{1}H NMR of the reaction mixture.

2.4. Control experiments in the presence of trimethyl sulfonium bromide

Procedure for reactions I and II. To a degassed solution of $BiBr_3$ (1 mol%) and trimethyl sulfonium bromide (3 mol%) in dry DMSO (2 mL) was added diethyl bromomalonate (1 mmol). The reaction vial for reaction **II** was placed inside a homemade 3D-printed reactor equipped with white LED strippers (~23 W). For reaction **I**, a sample was taken after 1 hour of stirring in the dark to be analyzed by UV-vis spectrometer. For reaction **II**, a sample was taken after 24 h under irradiation.

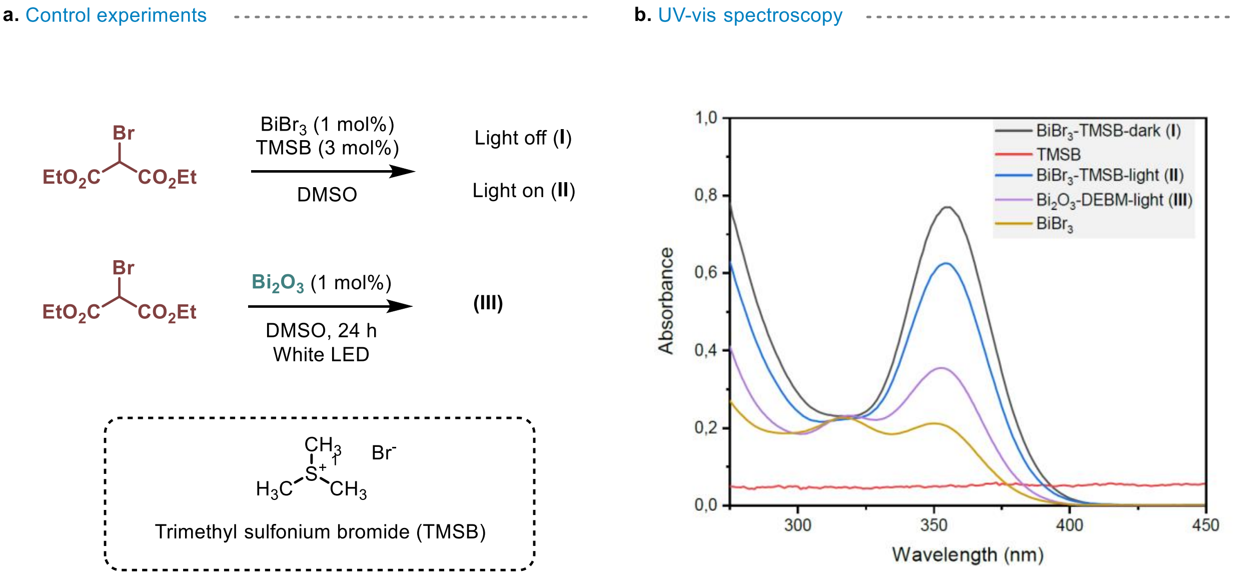

Supplementary Figure 5| Bi_2O_3 light-driven ATRA reaction in different solvents. a, Control experiments. **b**, Normalized UV-vis spectrum.

3. GC-MS

Procedure: To a degassed suspension of Bi_2O_3 (1 mol%) in dry DMSO (2 mL) was added diethyl bromomalonate (1 mmol). The reaction vial was placed inside a homemade 3D-printed reactor equipped with white LED strippers (~23 W). A sample was taken and diluted in CH_2Cl_2 after 24 hours of stirring to be analyzed by GC-MS.

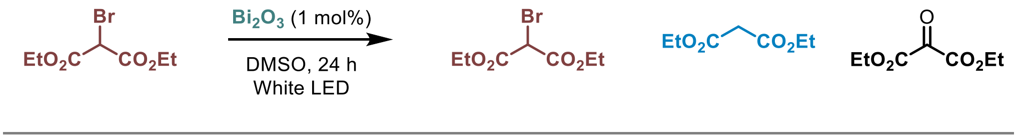

C:\Users\fnSTW167\Desktop\Data\PaolaRiente\PRP422.qgd

Chromatogram (All TIC)

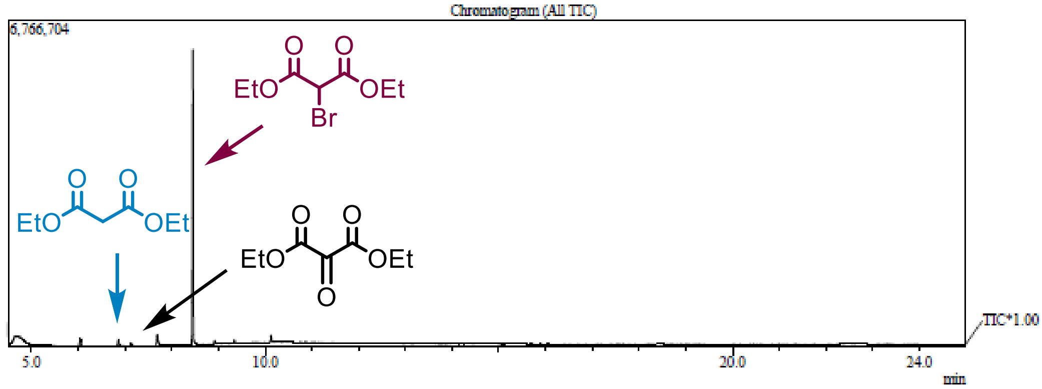

Chromatogram (Zoom)

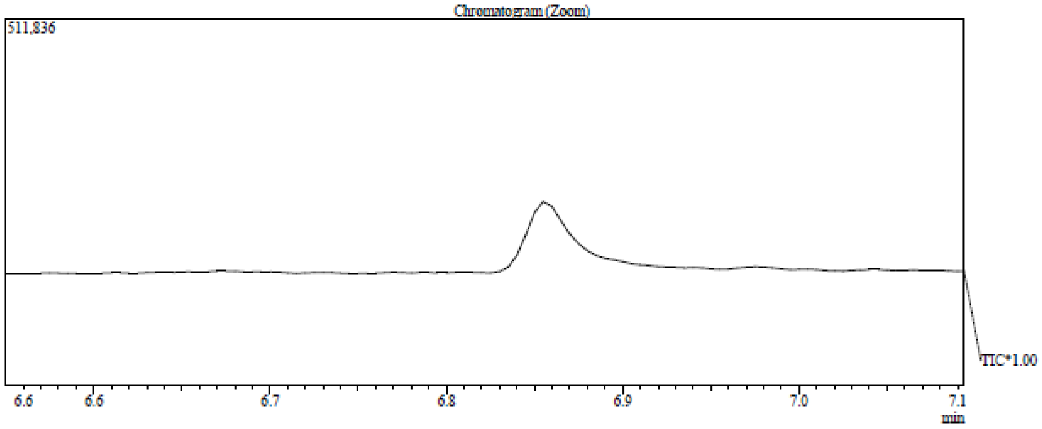

Spectrum

Line#:1 R.Time:6.855(Scan#:472)
MassPeaks:445
RawMode:Single 6.855(472) BasePeak:115.05(31970)
BG Mode:None Group 1 - Event 1 Scan

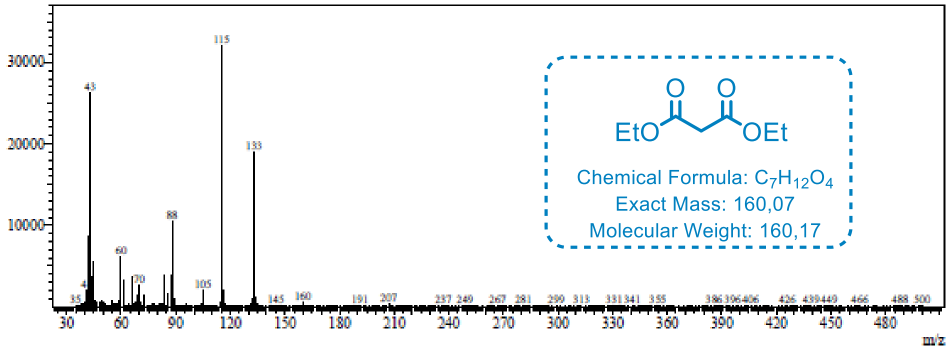

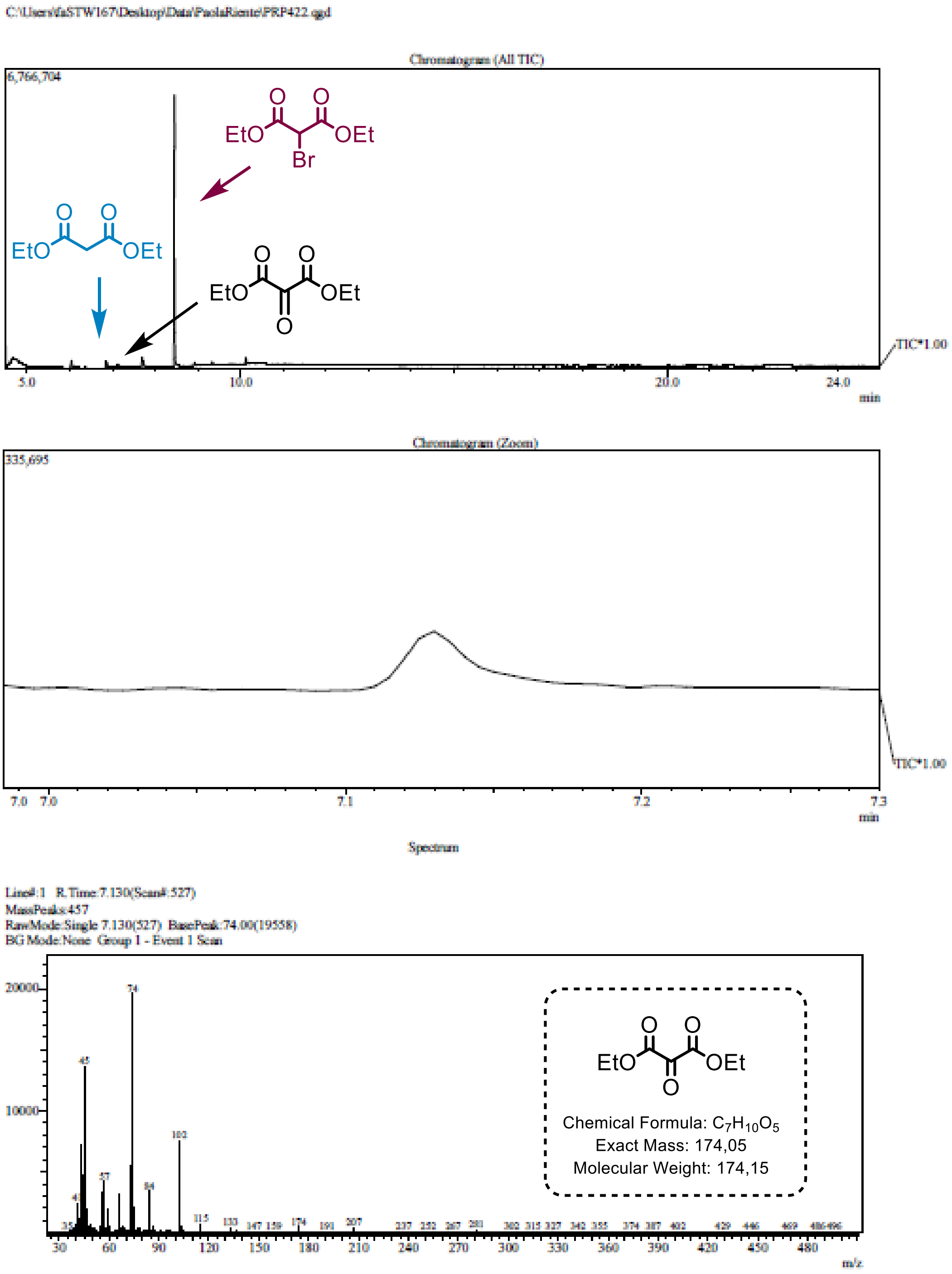

Supplementary Figure 6| GC-MS chromatography for the reaction between Bi_2O_3 and DEBM under irradiation.

4. Crystal data, data collection, and structure refinement parameters

CCDC 2021709 contains the supplementary crystallographic data for the structure presented in this paper.

4.1. Single Crystal X-Ray Methods

Crystal preparation: The crystals grew spontaneously in an NMR tube after 1 month from the reaction mixture containing Bi_2O_3 (1 mol%), DEBM (1 mmol) in DMSO-d6 after 24 h of irradiation. Any attempt to repeat the crystallization was unsuccessful.

Data collection: Crystal structure determination for the sample was carried out using an Apex DUO Kappa 4-axis goniometer equipped with an APPEX 2 4K CCD S5 area detector, a Microfocus Source E025 IuS using MoKα radiation, Quazar MX multilayer Optics as monochromator, and an Oxford Cryosystems low-temperature device Cryostream 700 plus (T= -173 °C). *Crystal structure determination for the sample:* Full-sphere data collection was used with ω and φ scans. Programs used: Data collection and data reduction Bruker APEX-2 (v2014.9-0).[2]

Structure Solution and Refinement: Crystal structure solution was achieved using the computer program SHELXT[3] Missing atoms were subsequently located from difference Fourier synthesis and added to the atom list. Least-squares refinement on F2 using all measured intensities was carried out using the program SHELXL 2018.[4] All non-hydrogen atoms were refined including anisotropic displacement parameters.

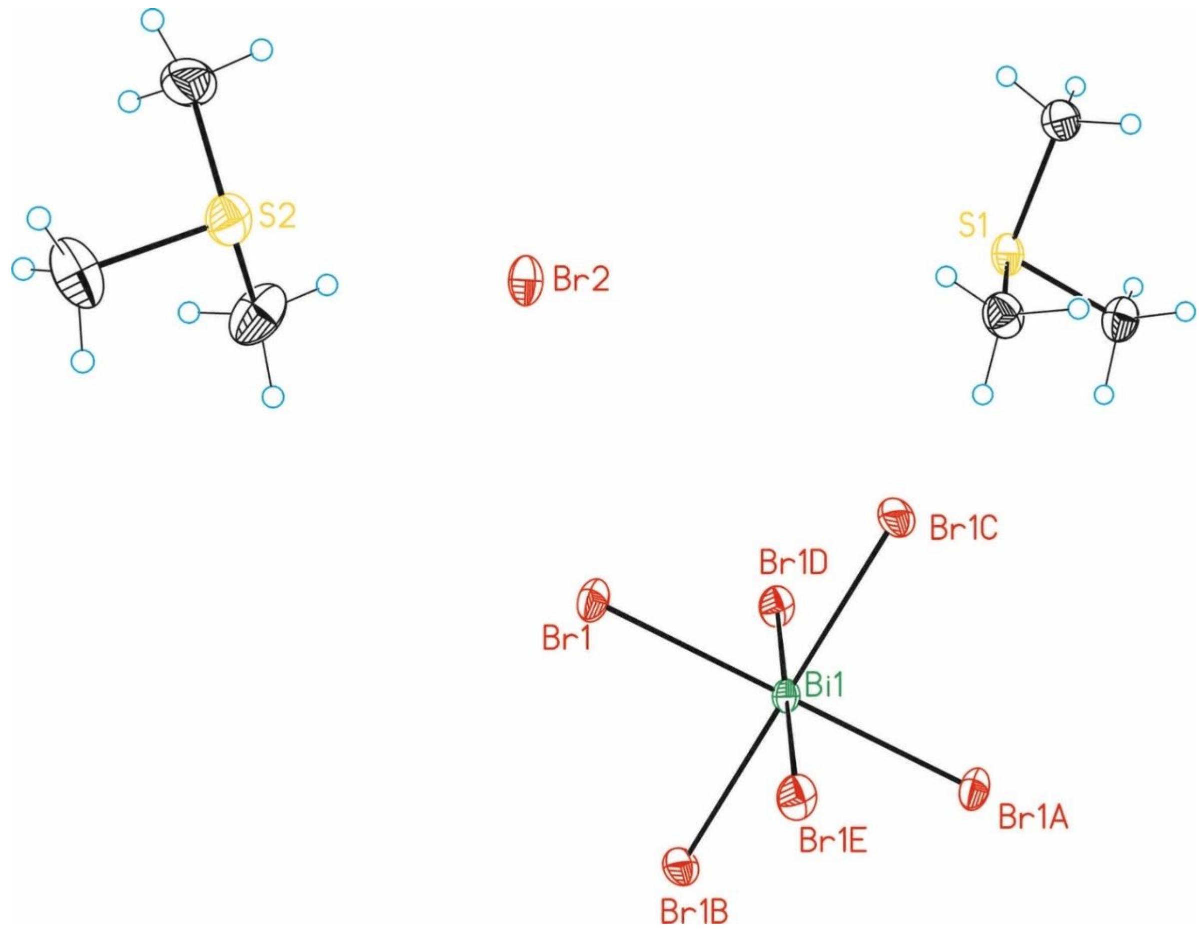

Supplementary Figure 7| ORTEP structure for C_{12} H_{36} Bi $Br_{6.88}$ $Cl_{0.12}$ S_4

Empirical formula C_{12} H_{36} Bi $Br_{6.88}$ $Cl_{0.12}$ S_4. Formula weight 1071.67

Temperature 100(2) K

Wavelength 0.71073 Å

Crystal system Trigonal

Space group R-3c

Unit cell dimensions a = 9.706 Å α= 90°, b = 9.706 Å β= 90°, c = 55.160 Å γ = 120°

Volume 4500.0 Å^3

Z 6

Density (calculated) 2.373 Mg/m^3

Absorption coefficient 15.301 mm^{-1}

F(000) 2987

Crystal size 0.400 x 0.300 x 0.250 mm^3

Theta range for data collection 2.215 to 30.530°.

Index ranges -11<=h<=10, -12<=k<=13, -64<=l<=78

Reflections collected 10611

Independent reflections 1536 [R(int) = 0.0533]

Completeness to theta = 25.242° 100.0 %

Refinement method Full-matrix least-squares on F^2

Data / restraints / parameters 1536 / 6 / 43

Goodness-of-fit on F^2 0.907

Final R indices [I>2sigma(I)] R1 = 0.0333, wR2 = 0.1114

R indices (all data) R1 = 0.0357, wR2 = 0.1139

Extinction coefficient n/a

Largest diff. peak and hole 2.539 and -2.257 e.$Å^{-3}$

5. Computational methods

The calculations were carried out using G09 D.01 program.[5] The structures were optimized at M06-L-D3/def2-SVP/SMD level (dimethyl sulfoxide, $\varepsilon = 46.826$); scalar relativistic small-core pseudopotential (ECP 60) designed for the basis sets of the def2-family has been employed for bismuth.[6-10] Local meta-GGA functional, M06-L, is a good choice for a blindfolded computational investigation since it has shown good-to-excellent overall accuracy in many parameters, including bond lengths and vibrational frequencies, noncovalent and "metallophilic" interactions, and thermodynamics for main-group elements of the periodic table. Single point calculations on optimized structures have been done at M06-L-D3/def2-TZVPP/SMD level. Vibrational analysis has been carried out to identify real minima and to obtain ZPE, enthalpic (H), and free energy corrections (G) to the potential energy via statistical thermodynamics. 298 K/1M has been chosen as a standard state. GoodVibes v. 2.0.3[11] has been used to introduce the quasi-harmonic corrections to vibrations ($\nu < 100\ cm^{-1}$ cut off).[12] Vertical excitations have been calculated at TD M06-L-D3/def2-TZVPP/SMD (DMSO) level. Both single-point energy corrections and TD DFT studies have been calculated using 99 radial shells by 590 angular points grid to avoid potential problems deriving from quadrature issues.[13] The stability of the wavefunctions has been tested to assign the correct electronic configuration to all the species. Spin contamination, arising from mixing of electronic states in UHF wavefunctions, has been considered negligible in all the pertinent cases (since $\langle\hat{S}^2\rangle_{UHF} \sim \langle\hat{S}^2\rangle_{exact}$). CHIMERA v.1.13.1 has been used to visualize, render, and generate all the graphical content included in the computational work.[14]

5.1. Scoring computation *vs.* experiment

To test the goodness of our level of theory in reproducing the thermodynamics of bismuth complexation we calculated the constant of equilibrium, log K_{eq}, (expressed as $\log K_{eq.} = -\frac{\Delta G^0}{2.303RT}$) for the titration of the [(κ^6-EDTA-H)Bi(OH_2)] complex (Supplementary Figure S8) with sodium hydroxide at 298 K and 1M standard state:

$$Bi(OH)_3 \;+\; EDTA\text{-}H_4 \rightleftharpoons [(\kappa^6\text{-}EDTA\text{-}H)Bi(OH_2)] \;+\; 2\; H_2O$$

Log K_{eq}		
Experiment<br><br>(titration at 298 K)	**Experiment**<br><br>**(polarography at 293 K)**	**Theory (this work)**<br><br>**at 298 K**
**26.47**	27.94	27.90 (-38 kcal·mol^{-1})

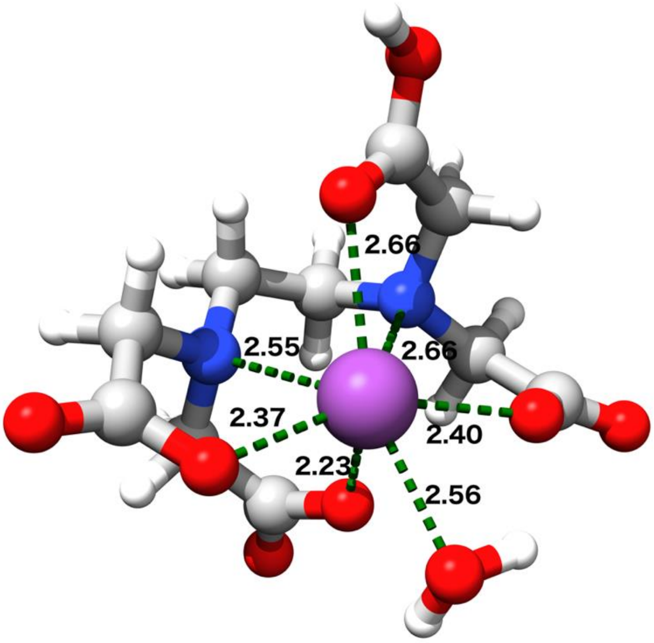

Supplementary Figure 8| Calculated structure of the heptacoordinated [(κ^6-EDTA-H)Bi(OH_2)] complex.

5.2. Chelation of diethyl bromomalonate onto Bi^{3+}: formation of Bi^{3+}•••O bonds

We used the $[Bi_9O_{13}(OH)]$ nanomer, derived from the X-ray characterized $[Bi_9O_8(OH)_6][CF_3SO_3]_5$ structure[15] by removal of five units of triflic acid as a reactant instead of a simpler Bi_2O_3 unit. In our opinion this approach is conceptually more accurate since $[Bi_9O_{13}(OH)]$ has been derived from an existing structure, it mimics more realistically the periodic surface of a solid (while retaining the computational efficiency of a fully homogeneous treatment) and avoids the unsaturation of the Bi coordination sphere (three-to-five coordination sites per bismuth are formally vacant in Bi_2O_3 unit).

Supplementary Table 1| General reaction scheme for Entries 1 to 48. The table includes values from Dataset 0, Dataset 1, Dataset 2, and Dataset 3 (SMD solvation is employed to model the properties of the implicit solvent (e.g. dielectric constant)).

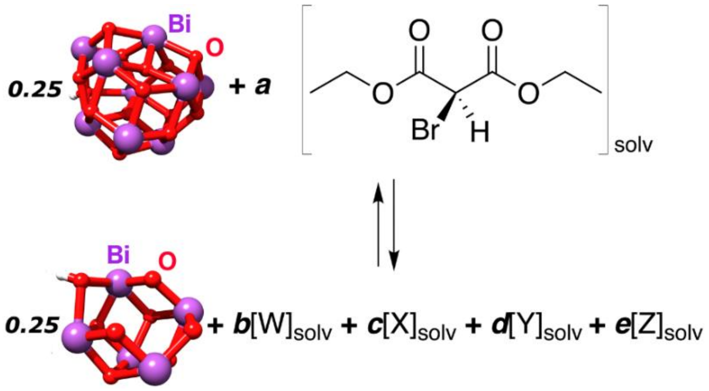

Bi ox. state	Dataset Entry Reaction	Coefficients[a]					Chemical species				ΔG^{0b}
		a	***b***	***c***	***d***	***e***	**W**	**X**	**Y**	**Z**	
III	***D-0*** E-1 (Redox)	1.50	0.50	0.50	0.75	0.00	P1	P2	O_2	N/A	**+23.6**
III	***D-0*** E-2 (Redox)	3.00	1.00	1.50	0.75	0.00	P1	Br_2	O_2	N/A	**+50.1**

III	***D-0*** E-3 (Redox)	1.50	0.50	0.25	0.50	0.00	P1	P3	O_2	N/A	**+47.2**
III	***D-0*** E-4 (Redox)	1.50	0.25	0.50	0.75	0.00	P4	P2	O_2	N/A	**+21.0**
III	***D-0*** E-5 (Redox)	3.00	0.50	1.50	0.75	0.00	P4	Br_2	O_2	N/A	**+45.0**
III	***D-0*** E-6 (Redox)	1.50	0.25	0.25	0.50	0.00	P4	P3	O_2	N/A	**+44.7**
III	***D-0*** E-7 (Redox)	1.50	0.50	0.50	0.75	0.00	P5	P6	O_2	N/A	**+25.0**
III	***D-0*** E-8 (Redox)	1.50	0.25	0.25	0.75	0.00	P7	P8	O_2	N/A	**+25.0**
III	***D-0*** E-9 (Redox)	1.00	0.50	0.50	0.25	0.00	P9	Br_2	O_2	N/A	**+31.3**
III	***D-0*** E-10 (Redox)	1.00	0.50	0.00	0.50	0.00	P10	N/A	O_2	N/A	**+21.4**
III	***D-0*** E-11 (Redox)	2.00	0.50	1.00	0.50	0.00	P11	Br_2	O_2	N/A	**+42.9**
III	***D-0*** E-12 (Acid-Base)	3.00	1.00	0.00	1.50	0.00	P12	N/A	H_2O	N/A	**+8.1**
III	***D-0*** E-13 (Acid-Base)	3.00	0.50	0.00	1.50	0.00	P13	N/A	H_2O	N/A	**+5.1**
III	***D-0*** E-14 (Acid-Base)	1.00	0.50	0.00	0.50	0.00	P14	N/A	H_2O	N/A	**+18.2**
III	***D-0*** E-15 (Acid-Base)	2.00	0.50	0.00	1.00	0.00	P15	N/A	H_2O	N/A	**+16.2**
III	***D-1*** E-16	3.00	1.00	1.50	0.75	0.00	P2	P16	O_2	N/A	**-5.4**

	(Redox)										
III	***D-2***<br>E-17<br>(Redox)	3.00	1.00	1.50	1.50	0.00	P2	P17	H_2O	N/A	**-46.7**
III	***D-3***<br>E-18<br>(Redox)	3.00	1.00	1.50	1.50	0.00	P2	P18	P19	N/A	**-53.4**
III	***D-1***<br>E-19<br>(Redox)	3.00	0.50	1.50	0.75	0.00	P20	P16	O_2	N/A	**-4.3**
III	***D-2***<br>E-20<br>(Redox)	3.00	0.50	1.50	1.50	0.00	P20	P17	H_2O	N/A	**-45.6**
III	***D-3***<br>E-21<br>(Redox)	3.00	0.50	1.50	1.50	0.00	P20	P18	P19	N/A	**-52.2**
III	***D-1***<br>E-22<br>(Redox)	3.00	0.50	1.50	0.50	0.00	P2	P16	P21	N/A	**+21.6**
IV	***D-1***<br>E-23<br>(Redox)	4.00	1.00	2.00	0.75	0.00	P22	P16	O_2	N/A	**+8.1**
IV	***D-2***<br>E-24<br>(Redox)	4.00	1.00	0.50	1.50	1.50	P22	P16	H_2O	P17	**-33.2**
IV	***D-3***<br>E-25<br>(Redox)	4.00	1.00	0.50	1.50	1.50	P22	P16	P18	P19	**-39.8**
IV	***D-1***<br>E-26<br>(Redox)	4.00	0.50	2.00	0.75	0.00	P23	P16	O_2	N/A	**+11.1**
IV	***D-2***<br>E-27<br>(Redox)	4.00	0.50	0.50	1.50	1.50	P23	P16	H_2O	P17	**-30.3**
IV	***D-3***<br>E-28<br>(Redox)	4.00	0.50	0.50	1.50	1.50	P23	P16	P18	P19	**-36.9**
IV	***D-0***<br>E-29<br>(Redox)	2.00	1.00	0.00	0.75	0.00	P24	N/A	O_2	N/A	**+35.5**
IV	***D-0***<br>E-30<br>(Redox)	2.00	1.00	0.00	0.75	0.00	P25	N/A	O_2	N/A	**+35.3**
V	***D-1***<br>E-31<br>(Redox)	5.00	1.00	2.50	0.75	0.00	P26	P16	O_2	N/A	**+24.7**
V	***D-2***<br>E-32<br>(Redox)	5.00	1.00	1.00	1.50	1.50	P26	P16	H_2O	P17	**-16.6**
V	***D-3***	5.00	1.00	1.00	1.50	1.50	P26	P16	P18	P19	**-23.2**

	E-33 (Redox)										
V	***D-1*** E-34 (Redox)	5.00	0.50	2.50	0.75	0.00	P27	P16	O_2	N/A	**+20.4**
V	***D-2*** E-35 (Redox)	5.00	0.50	1.00	1.50	1.50	P27	P16	H_2O	P17	**-20.9**
V	***D-3*** E-36 (Redox)	5.00	0.50	1.00	1.50	1.50	P27	P16	P18	P19	**-27.6**
II	***D-1*** E-37 (Redox)	2.00	1.00	1.00	0.75	0.00	P28	P16	O_2	N/A	**+32.7**
II	***D-2*** E-38 (Redox)	2.00	1.00	1.00	0.25	1.00	P28	P17	O_2	H_2O	**+5.2**
II	***D-3*** E-39 (Redox)	2.00	1.00	1.00	0.25	1.00	P28	P18	O_2	P19	**+0.7**
II	***D-1*** E-40 (Redox)	2.00	1.00	0.50	0.75	0.00	P29	P16	O_2	N/A	**+21.3**
II	***D-2*** E-41 (Redox)	2.00	0.50	1.00	0.25	1.00	P29	P17	O_2	H_2O	**-6.2**
II	***D-3*** E-42 (Redox)	2.00	0.50	1.00	0.25	1.00	P29	P18	O_2	P19	**-10.6**
II	***D-1*** E-43 (Redox)	3.00	1.00	1.50	0.75	0.50	P28	P16	O_2	Br_2	**+31.9**
II	***D-2*** E-44 (Redox)	3.00	1.00	1.50	1.50	0.50	P28	P17	H_2O	Br_2	**-9.4**
II	***D-3*** E-45 (Redox)	3.00	1.00	1.50	1.50	0.50	P28	P18	P19	Br_2	**-16.1**
II	***D-1*** E-46 (Redox)	3.00	0.50	1.50	0.75	0.50	P29	P16	O_2	Br_2	**+20.5**
II	***D-2*** E-47 (Redox)	3.00	0.50	1.50	1.50	0.50	P29	P17	H_2O	Br_2	**-20.8**
II	***D-3*** E-48 (Redox)	3.00	0.50	1.50	0.50	1.50	P29	P18	Br_2	P19	**-27.4**

[a] The coefficients are valid per Bi equivalent (since four bismuth atoms are involved in the reactivity reported herein, the coefficient should be multiplied by 4 to find the overall change).
[b]Expressed in kcal·mol^{-1} per Bi equivalent, 1M standard state at 298 K.

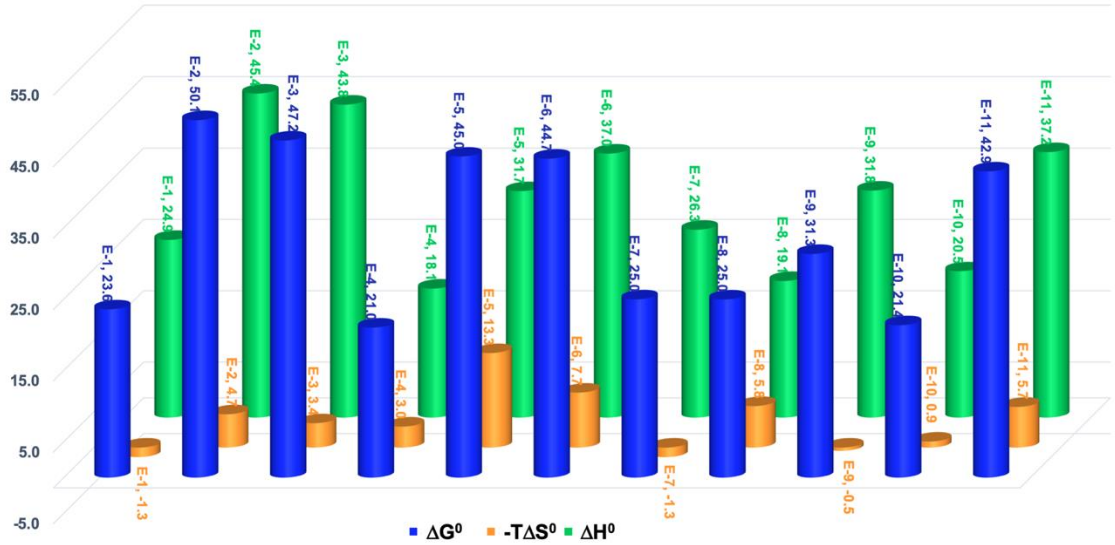

Supplementary Figure 9| Thermodynamics for the formation of Bi(III) products (kcal·mol^{-1} *vs* Entries) featuring chelated Bi(III)···O complexation between bismuth and malonate moiety (Dataset 0). ΔG^0 values are in blue, $-T\Delta S^0$ in orange, and ΔH^0 in green.

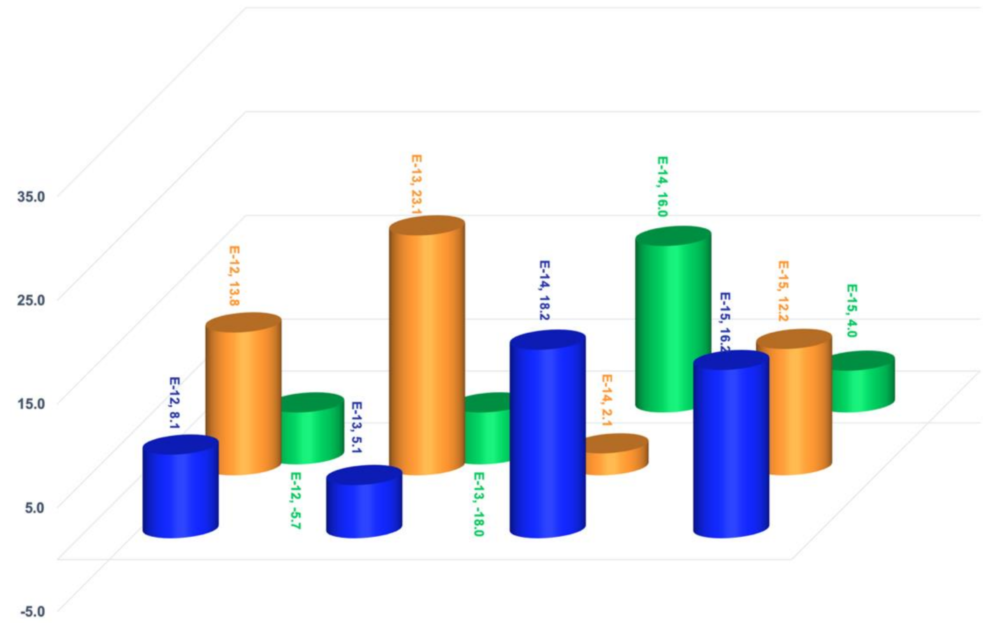

Supplementary Figure 10| Thermodynamics for the acid-base reactions (kcal·mol^{-1} *vs* Entries) featuring chelated Bi(III)···O complexation between bismuth and malonate moiety (Dataset 0). ΔG^0 values are in blue, $-T\Delta S^0$ in orange, and ΔH^0 in green.

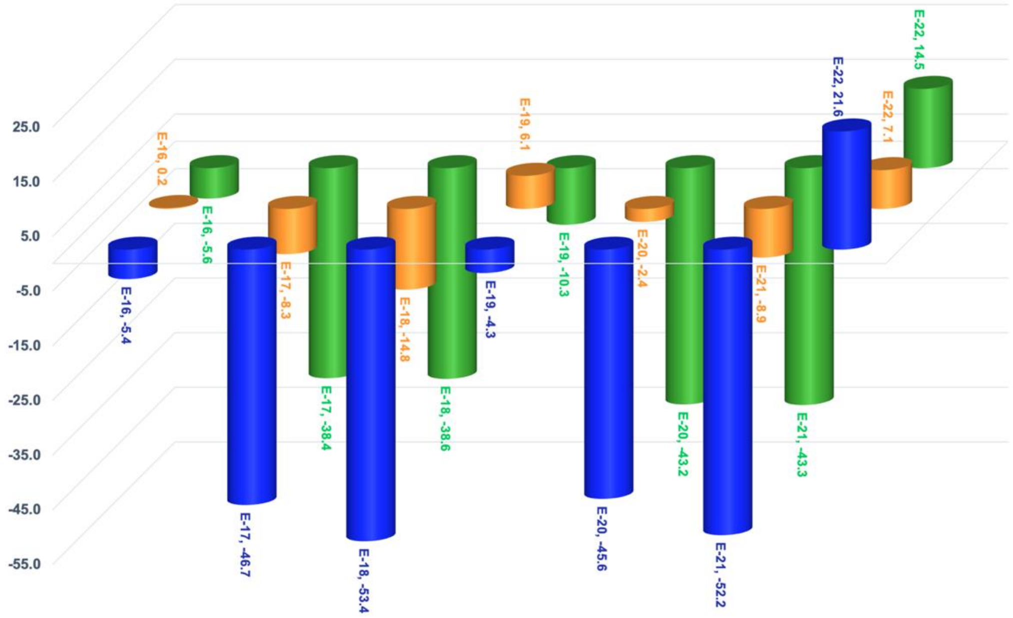

Supplementary Figure 11| Thermodynamics for the formation of Bi(III) products (kcal·mol^{-1} vs Entries) featuring Bi(III)-Br complexation. ΔG^0 values are in blue, $-T\Delta S^0$ in orange, and ΔH^0 in green.

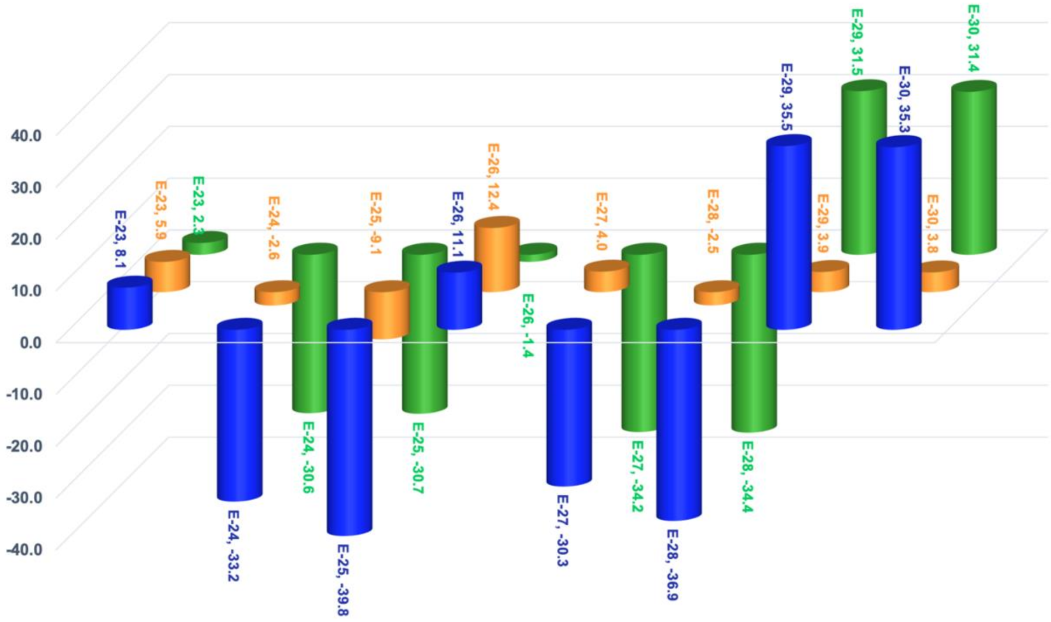

Supplementary Figure 12| Thermodynamics for the formation of Bi(IV) products ($kcal \cdot mol^{-1}$ vs Entries) featuring Bi(IV)-Br complexation. ΔG^0 values are in blue, $-T\Delta S^0$ in orange, and ΔH^0 in green.

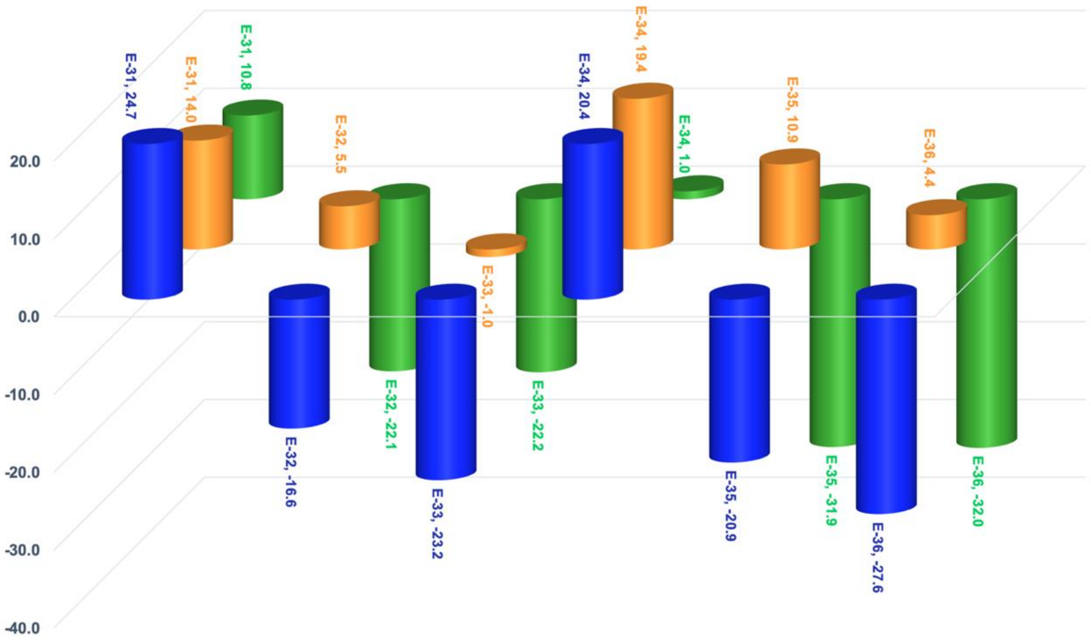

Supplementary Figure 13| Thermodynamics for the formation of Bi(V) products ($kcal \cdot mol^{-1}$ vs Entries) featuring Bi(V)-Br complexation. ΔG^0 values are in blue, $-T\Delta S^0$ in orange, and ΔH^0 in green.

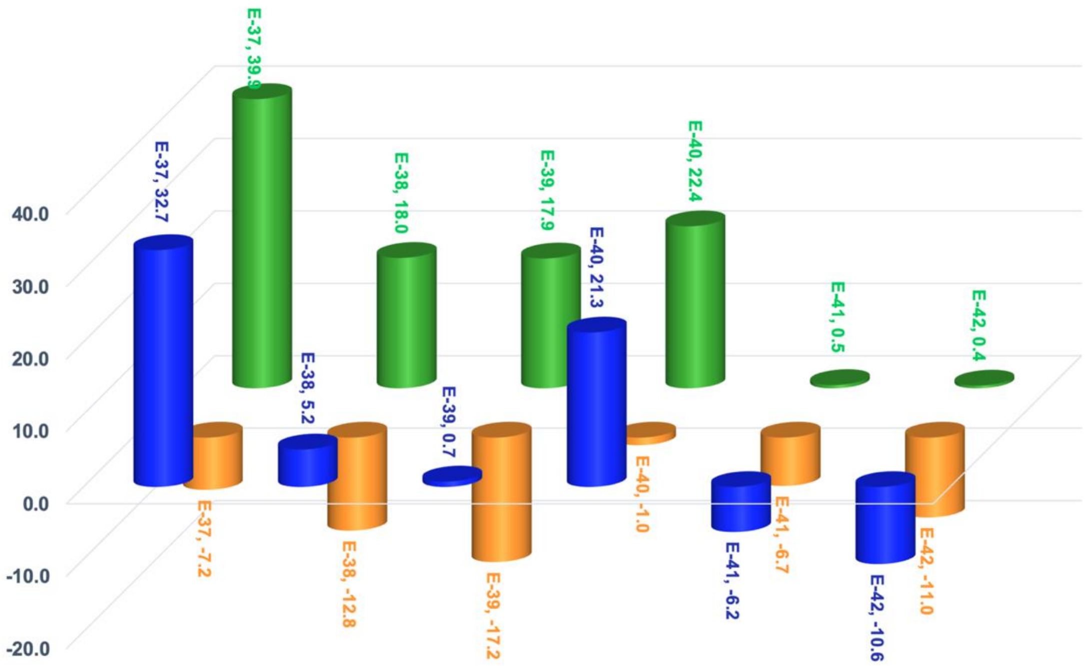

Supplementary Figure 14| Thermodynamics for the formation of Bi(II) products (kcal·mol^{-1} vs Entries) featuring Bi(II)-Br complexation. ΔG^0 values are in blue, -TΔS^0 in orange, and ΔH^0 in green.

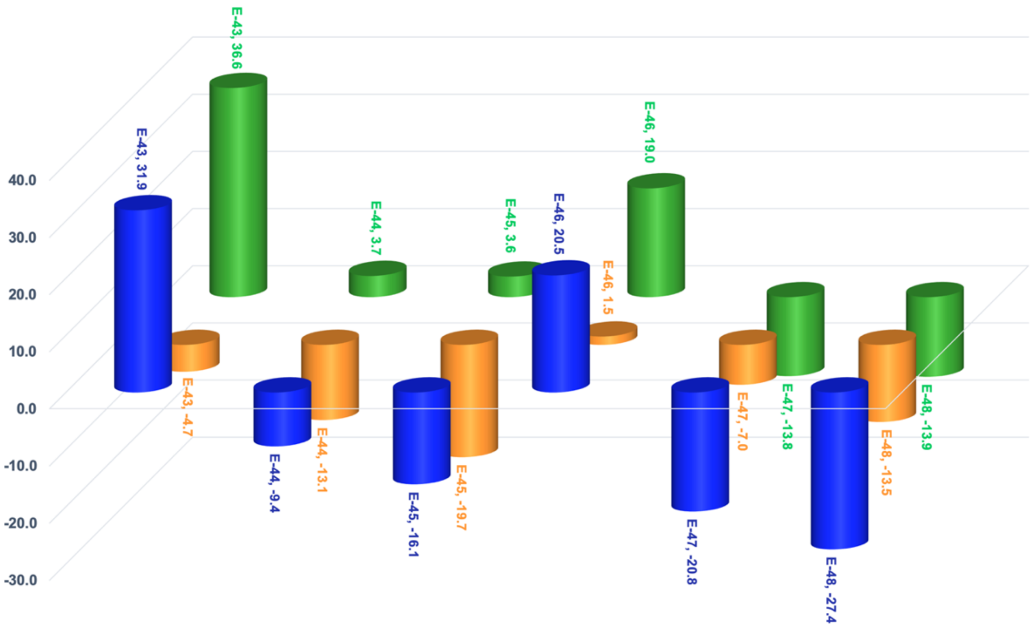

Supplementary Figure 15| Thermodynamics for the formation of Bi(II) products (kcal·mol^{-1} vs Entries) featuring Bi(II)-Br complexation with the concomitant formation of Br_2. ΔG^0 values are in blue, -TΔS^0 in orange, and ΔH^0 in green.

Entries for the Supplementary Table 2

Entry 1 – Dataset 0

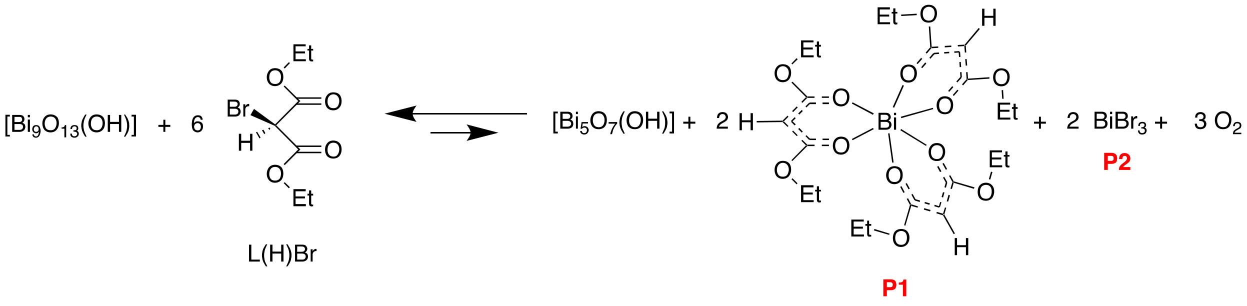

OX: $[Bi_9O_{13}(OH)] \rightleftharpoons [Bi_5O_7(OH)] + 4\ Bi^{3+} + 3\ O_2 + 12\ e^-$ | **1**

RED: $L(H)Br + 2\ e^- \rightleftharpoons L(H)^- + Br^-$ | **6**

Entry 2 – Dataset 0

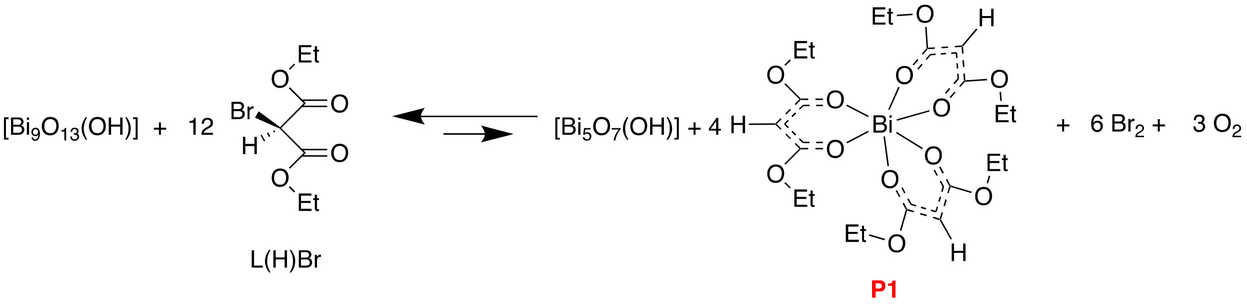

OX: $[Bi_9O_{13}(OH)] \rightleftharpoons [Bi_5O_7(OH)] + 4\ Bi^{3+} + 3\ O_2 + 12\ e^-$ | **1**

RED: $2\ L(H)Br + 2\ e^- \rightleftharpoons 2\ L(H)^- + Br_2$ | **6**

Entry 3 – Dataset 0

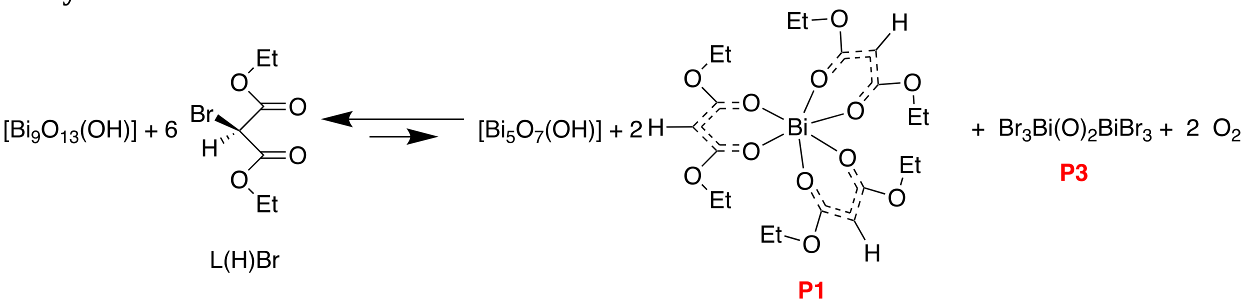

**OX:** $[Bi_9O_{13}(OH)]$	$\rightleftharpoons$	$[Bi_5O_7(OH)] + 2\ Bi^{3+} + 2\ Bi^{5+} + 2\ O^{2-} + 2\ O_2 + 12\ e^-$	**1**
**RED:** L(H)Br $+ 2\ e^-$	$\rightleftharpoons$	$L(H)^- + Br^-$	**6**

Entry 4 – Dataset 0

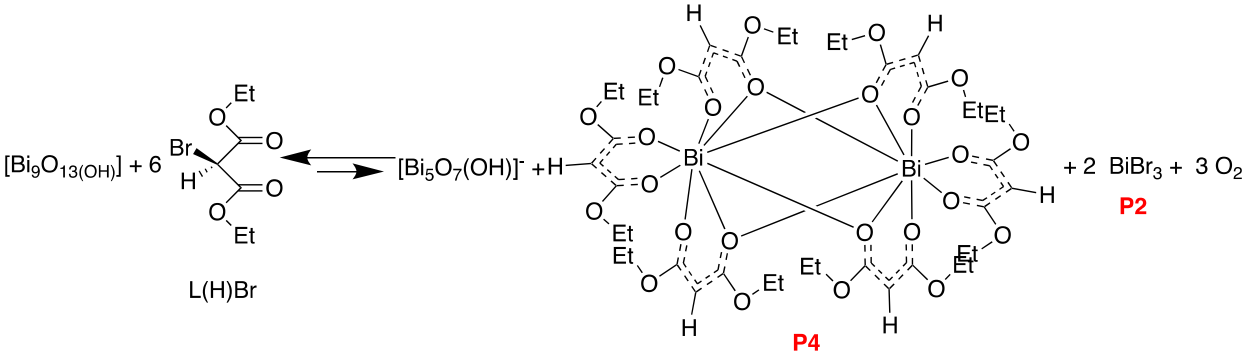

**OX:** $[Bi_9O_{13(OH)}]$	$\rightleftharpoons$	$[Bi_5O_{7(OH)}] + 4\ Bi^{3+} + 3\ O_2 + 12\ e^-$	**1**
**RED:** L(H)Br $+ 2\ e^-$	$\rightleftharpoons$	$L(H)^- + Br^-$	**6**

Entry 5 – Dataset 0

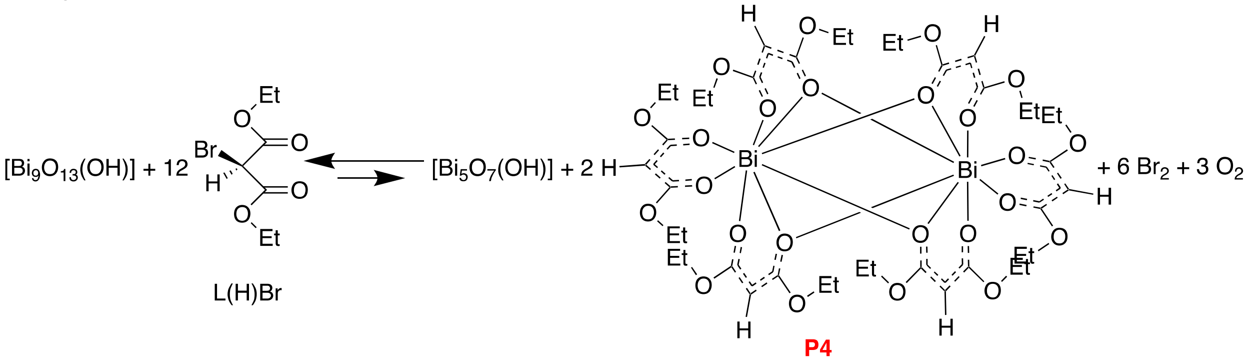

$$\textbf{OX:}\quad [Bi_9O_{13}(OH)] \rightleftharpoons [Bi_5O_7(OH)] + 4\,Bi^{3+} + 3\,O_2 + 12\,e^- \qquad |\ 1$$

$$\textbf{RED:}\quad 2\ L(H)Br + 2\,e^- \rightleftharpoons 2\ L(H)^- + Br_2 \qquad |\ 6$$

Entry 6 – Dataset 0

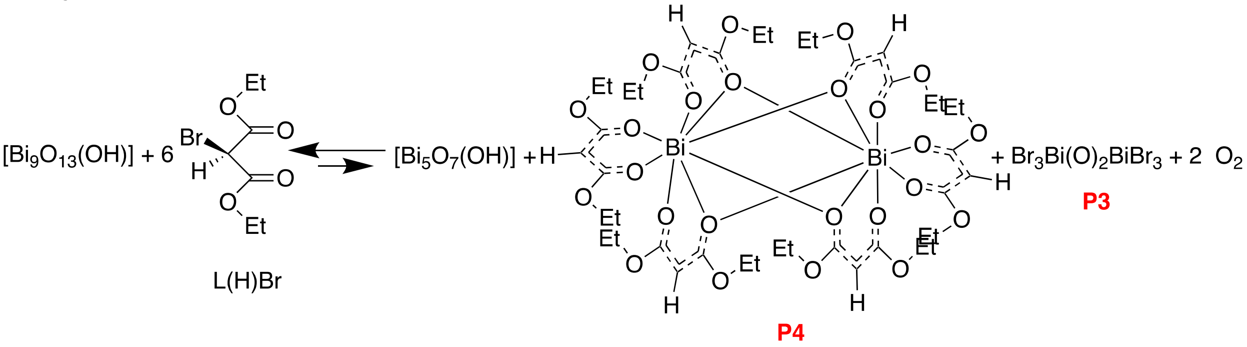

$$\textbf{OX:}\quad [Bi_9O_{13}(OH)] \rightleftharpoons [Bi_5O_7(OH)] + 2\,Bi^{3+} + 2\,Bi^{5+} + 2\,O^{2-} + 2\,O_2 + 12\,e^- \qquad |\ 1$$

$$\textbf{RED:}\quad L(H)Br + 2\,e^- \rightleftharpoons L(H)^- + Br^- \qquad |\ 6$$

Entry 7 – Dataset 0

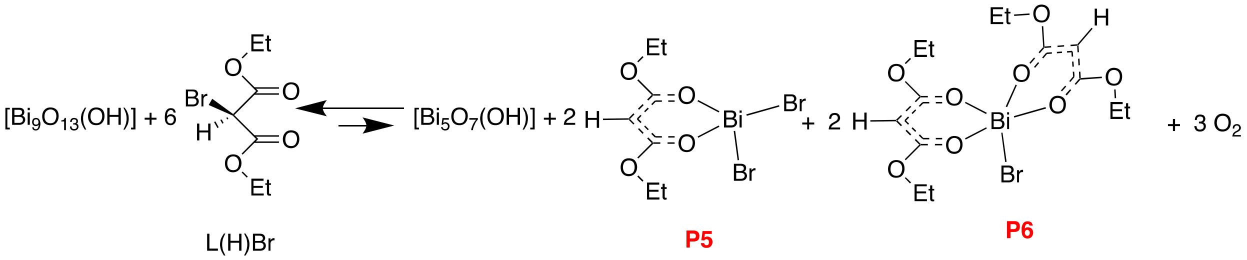

OX: $[Bi_9O_{13}(OH)] \rightleftharpoons [Bi_5O_7(OH)] + 4\ Bi^{3+} + 3\ O_2 + 12\ e^-$ | **1**

RED: $L(H)Br + 2\ e^- \rightleftharpoons L(H)^- + Br^-$ | **6**

Entry 8 – Dataset 0

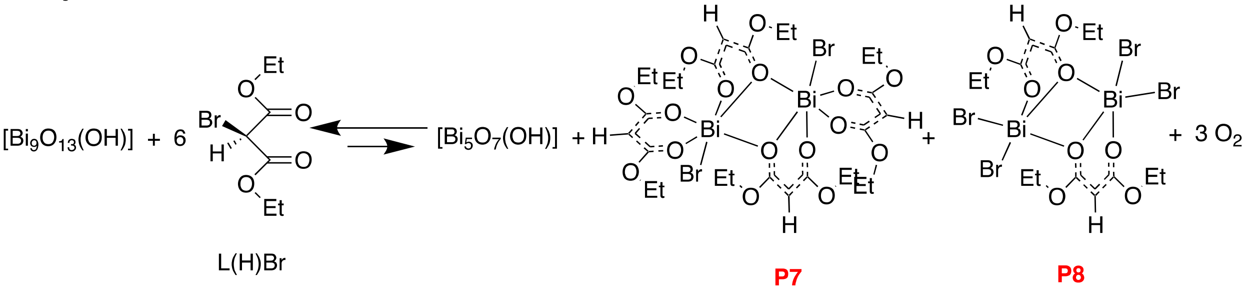

OX: $[Bi_9O_{13}(OH)] \rightleftharpoons [Bi_5O_7(OH)] + 4\ Bi^{3+} + 3\ O_2 + 12\ e^-$ | **1**

RED: $L(H)Br + 2\ e^- \rightleftharpoons L(H)^- + Br^-$ | **6**

Entry 9 – Dataset 0

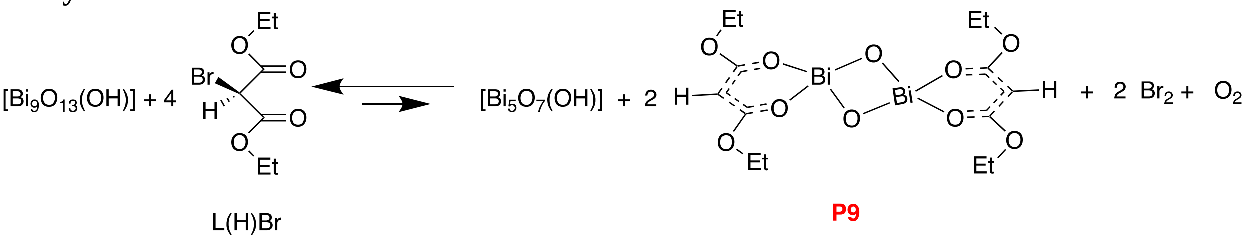

OX: $[Bi_9O_{13}(OH)] \rightleftharpoons [Bi_5O_7(OH)] + 2\ [Bi(O)_2Bi]^{2+} + O_2 + 4\ e^-$ | **1**

RED: $2\ L(H)Br + 2\ e^- \rightleftharpoons 2\ L(H)^- + Br_2$ | **2**

Entry 10 – Dataset 0

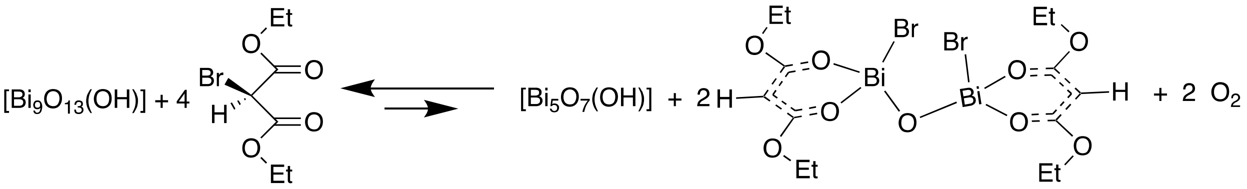

L(H)Br

P10

OX: $[Bi_9O_{13}(OH)] \rightleftharpoons [Bi_5O_7(OH)] + 2\,[Bi(O)Bi]^{4+} + 2\,O_2 + 8\,e^-$ | 1

RED: $L(H)Br + 2\,e^- \rightleftharpoons L(H)^- + Br^-$ | 4

Entry 11 – Dataset 0

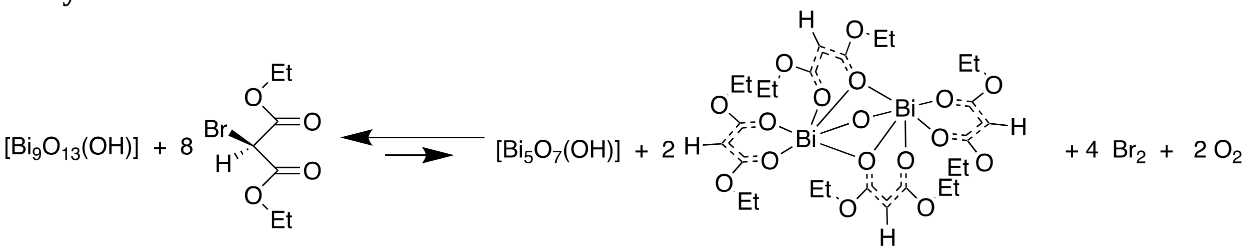

L(H)Br

P11

OX: $[Bi_9O_{13}(OH)] \rightleftharpoons [Bi_5O_7(OH)] + 2\,[Bi(O)Bi]^{4+} + 2\,O_2 + 8\,e^-$ | 1

RED: $2\ L(H)Br + 2\,e^- \rightleftharpoons 2\ L(H)^- + Br_2$ | 4

Entry 12 – Dataset 0

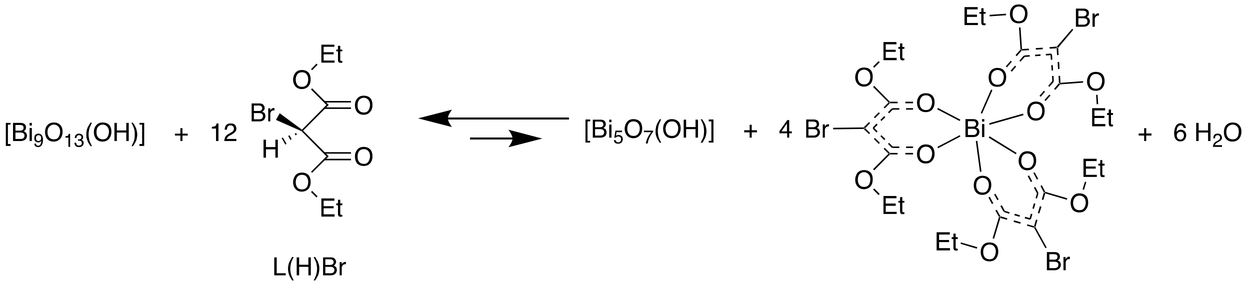

L(H)Br

P12

Entry 13 – Dataset 0

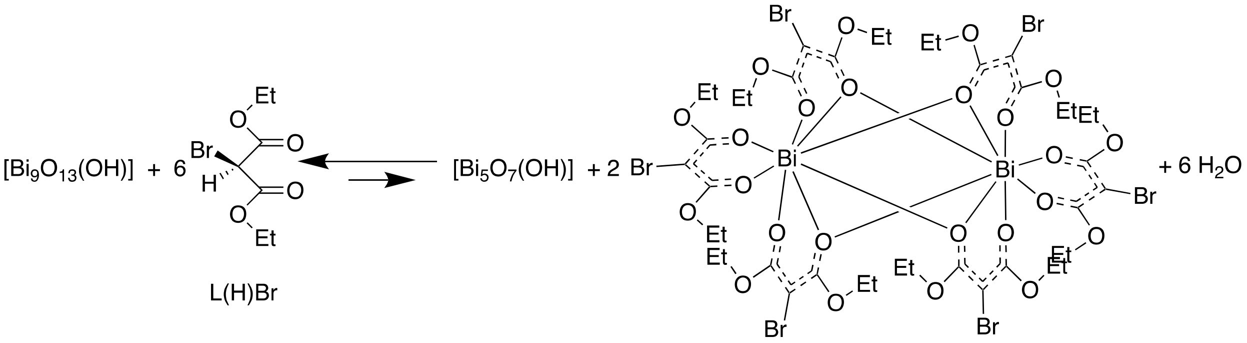

P13

Entry 14 – Dataset 0

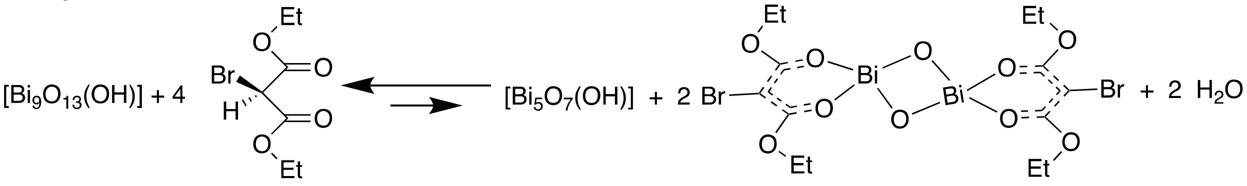

L(H)Br

P14

Entry 15 – Dataset 0

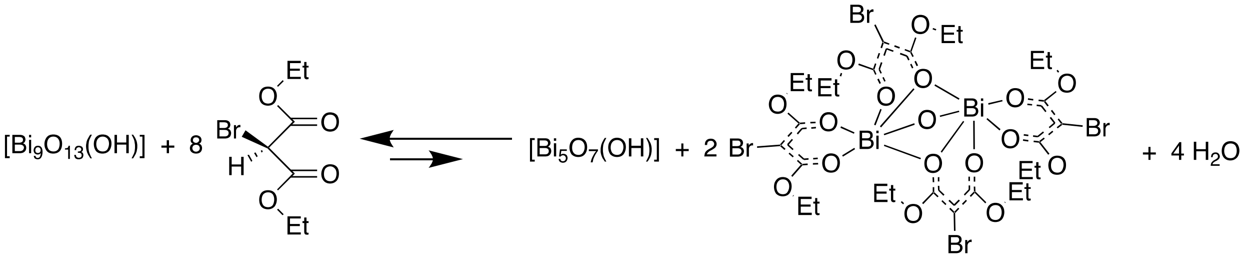

P15

Entry 16 – Dataset 1

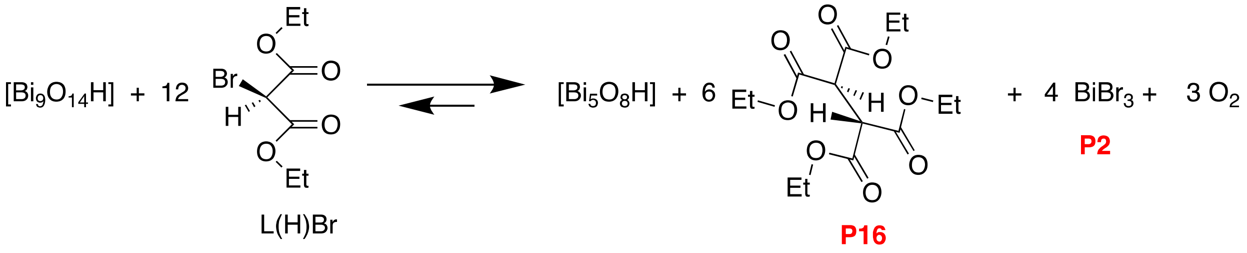

OX: $[Bi_9O_{13}(OH)] \rightleftharpoons [Bi_5O_7(OH)] + 4\ Bi^{3+} + 3\ O_2 + 12\ e^-$ | **1**

RED: $2\ L(H)Br + 2\ e^- \rightleftharpoons P16 + 2\ Br^-$ | **6**

Entry 17 – Dataset 2

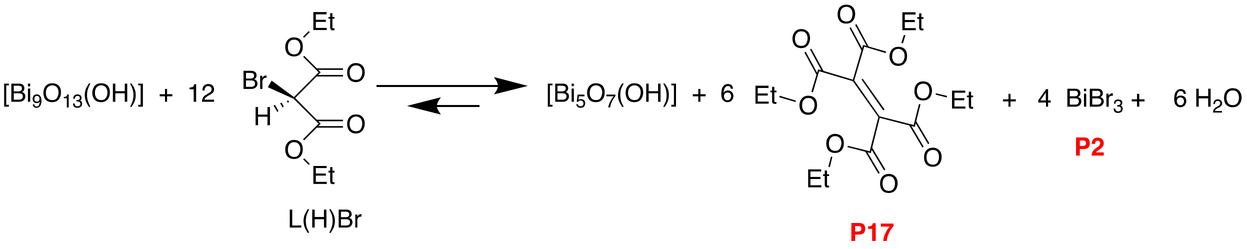

OX: $[Bi_9O_{13}(OH)] + 12\ L(H)^{\bullet} \rightleftharpoons [Bi_5O_7(OH)] + P17 + 4\ Bi^{3+} + 6\ H_2O + 12\ e^-$ | **1**

RED: $Br^{\bullet} + 1\ e^- \rightleftharpoons Br^-$ | **12**

Entry 18 – Dataset 3

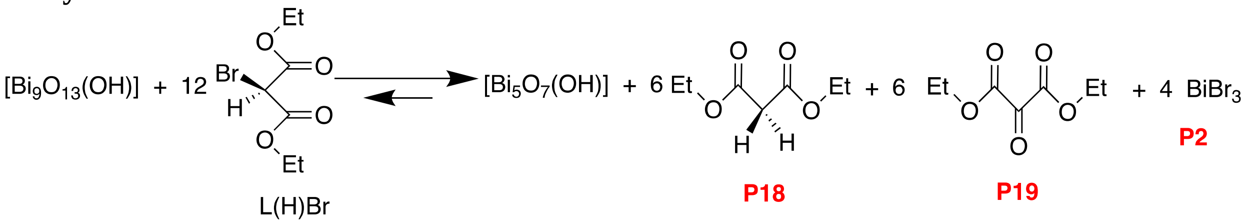

OX: $[Bi_9O_{13}(OH)] + 12\ L(H)^{\bullet} \rightleftharpoons [Bi_5O_7(OH)] + 6\ P18 + 6\ P19 + 4\ Bi^{3+} + 12\ e^-$ | **1**

RED: $Br^{\bullet} + 1\ e^- \rightleftharpoons Br^-$ | **12**

Entry 19 – Dataset 1

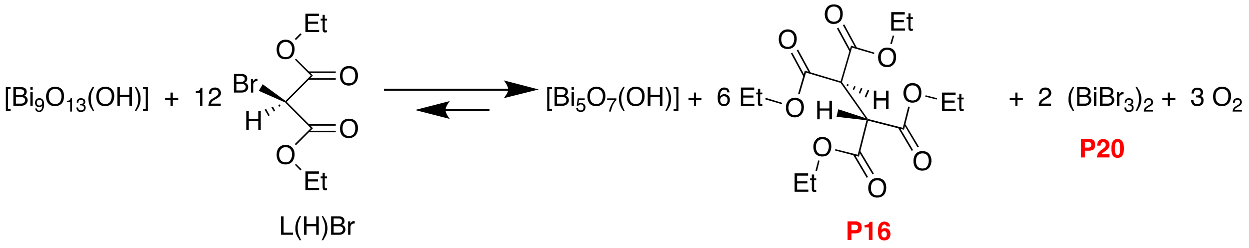

OX: $[Bi_9O_{13}(OH)] \rightleftharpoons [Bi_5O_7(OH)] + 4\ Bi^{3+} + 3\ O_2 + 12\ e^-$ | **1**

RED: $2\ L(H)Br + 2\ e^- \rightleftharpoons P16 + 2\ Br^-$ | **6**

Entry 20 – Dataset 2

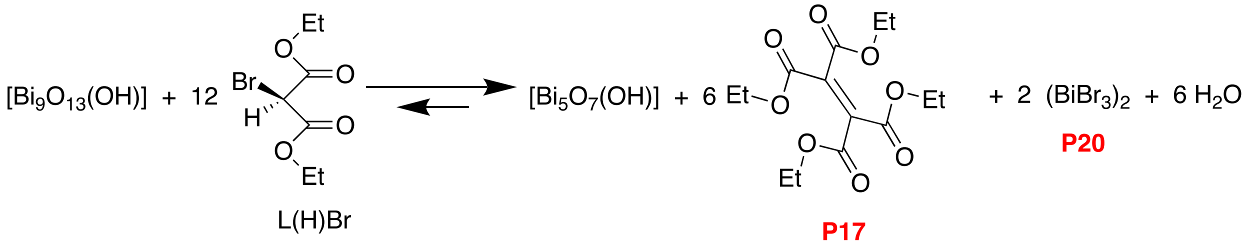

OX: $[Bi_9O_{13}(OH)] + 12\ L(H)^{\bullet} \rightleftharpoons [Bi_5O_7(OH)] + 6\ P17 + 4\ Bi^{3+} + 6\ H_2O + 12\ e^-$ | **1**

RED: $Br^{\bullet} + 1\ e^- \rightleftharpoons Br^-$ | **12**

Entry 21 – Dataset 3

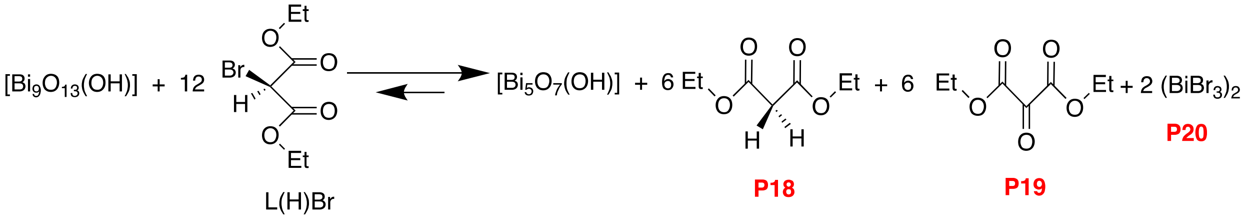

OX: $[Bi_9O_{13}(OH)] + 12\ L(H)^{\bullet} \rightleftharpoons [Bi_5O_7(OH)] + 6\ P18 + 6\ P19 + 4\ Bi^{3+} + 12\ e^-$ | **1**

RED: $Br^{\bullet} + 1\ e^- \rightleftharpoons Br^-$ | **12**

Entry 22 – Dataset 1

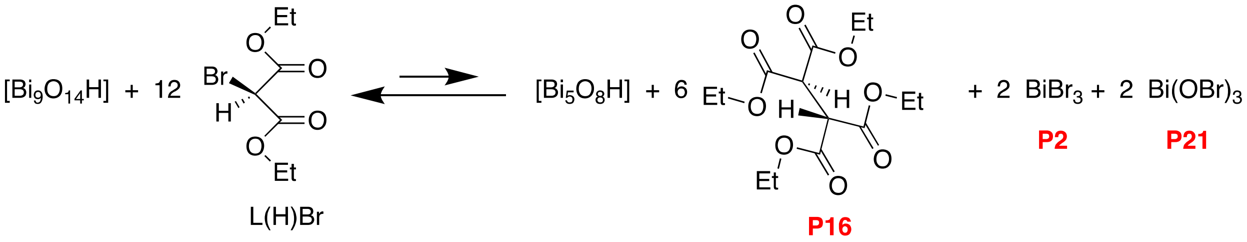

OX: $[Bi_9O_{14}H] + 6\ L(H)Br \rightleftharpoons [Bi_5O_8H] + 4\ Bi^{3+} + 6\ L(H)^{\bullet} + 6\ OBr^- + 6\ e^-$ | **1**

RED: $L(H)Br + 1\ e^- \rightleftharpoons L(H)^{\bullet} + Br^-$ | **6**

Entry 23 – Dataset 1

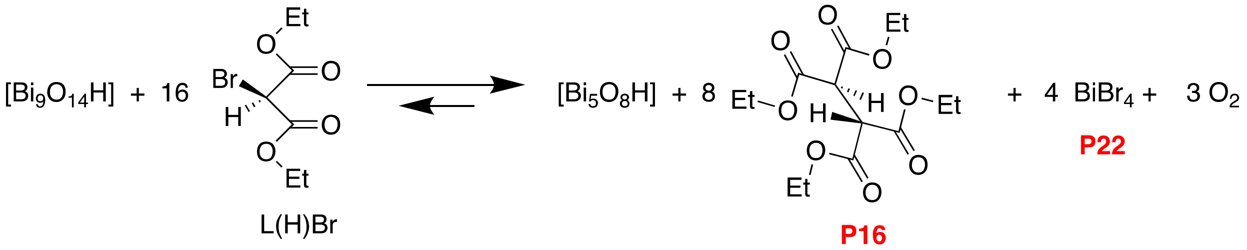

OX: $[Bi_9O_{14}H] \rightleftharpoons [Bi_5O_8H] + 4\ Bi^{4+} + 3\ O_2 + 16\ e^-$ | **1**

RED: $2\ L(H)Br + 2\ e^- \rightleftharpoons P16 + 2\ Br^-$ | **8**

Entry 24 – Dataset 2

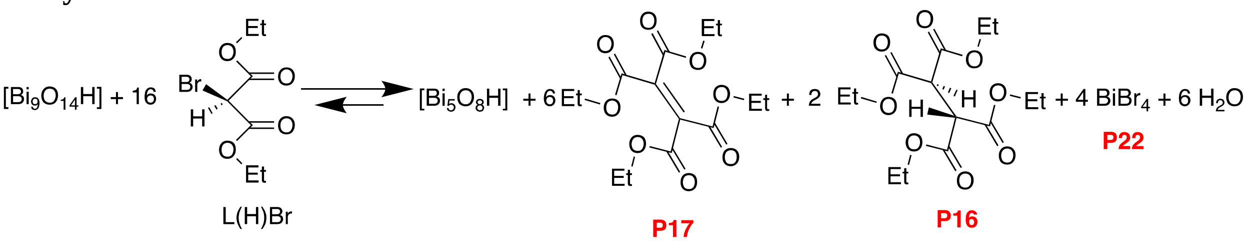

OX: $[Bi_9O_{14}H] + 16\ L(H)^{\bullet} \rightleftharpoons [Bi_5O_8H] + 6\ P17 + 2\ P16 + 4\ Bi^{4+} + 6\ H_2O + 16\ e^-$ | **1**

RED: $Br^{\bullet} + 1\ e^- \rightleftharpoons Br^-$ | **16**

Entry 25 – Dataset 3

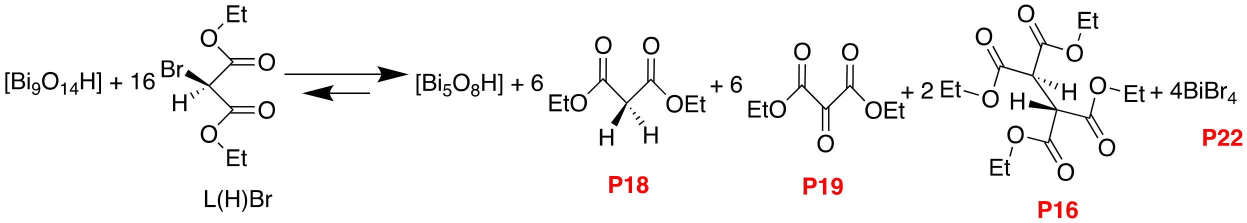

OX: $[Bi_9O_{14}H] + 16\ L(H)^{\bullet} \rightleftharpoons [Bi_5O_8H] + 6\ P18 + 6\ P19 + 2\ P16 + 4\ Bi^{4+} + 16\ e^-$ | **1**

RED: $Br^{\bullet} + 1\ e^- \rightleftharpoons Br^-$ | **16**

Entry 26 – Dataset 1

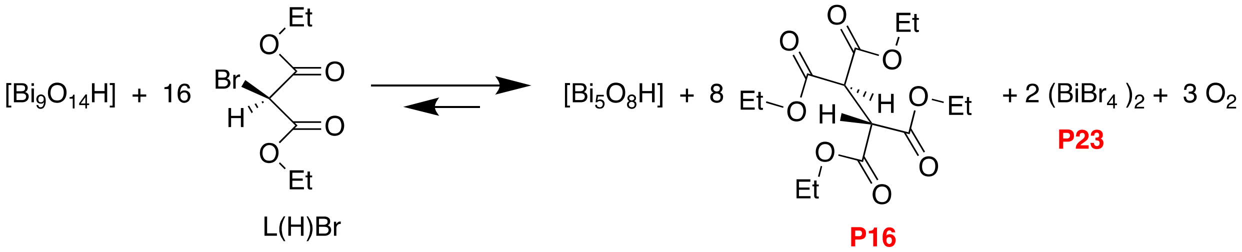

OX: $[Bi_9O_{14}H] \rightleftharpoons [Bi_5O_8H] + 4\ Bi^{4+} + 3\ O_2 + 16\ e^-$ | **1**

RED: $2\ L(H)Br + 2\ e^- \rightleftharpoons P16 + 2\ Br^-$ | **8**

Entry 27 – Dataset 2

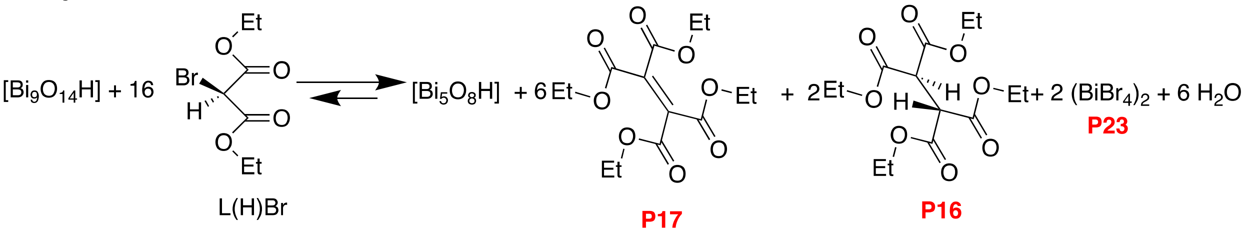

OX: $[Bi_9O_{14}H] + 16\ L(H)^{\bullet} \rightleftharpoons [Bi_5O_8H] + 6\ P17 + 2\ P16 + 4\ Bi^{4+} + 6\ H_2O + 16\ e^-$ | **1**

RED: $Br^{\bullet} + 1\ e^- \rightleftharpoons Br^-$ | **16**

Entry 28 – Dataset 3

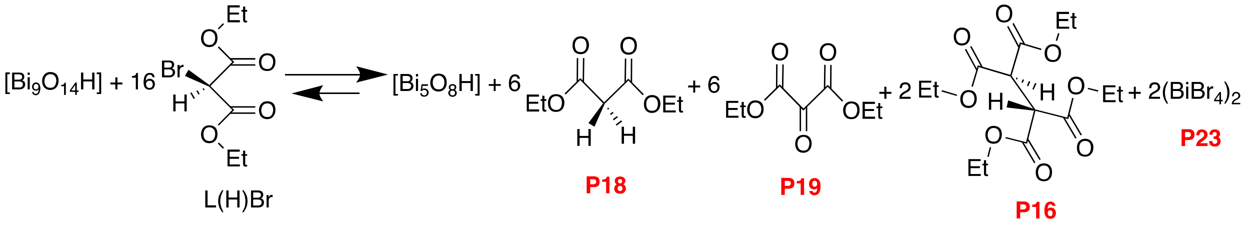

OX: $[Bi_9O_{14}H] + 16\,L(H)^{\bullet} \rightleftharpoons [Bi_5O_8H] + 6\ P18 + 6\ P19 + 2\ P16 + 4\,Bi^{4+} + 16\,e^-$ | **1**

RED: $Br^{\bullet} + 1\,e^- \rightleftharpoons Br^-$ | **16**

Entry 29 – Dataset 0

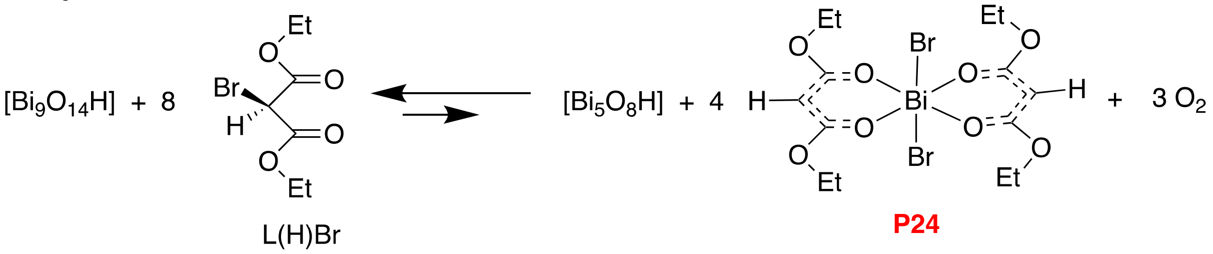

OX: $[Bi_9O_{14}H] \rightleftharpoons [Bi_5O_8H] + 4\,Bi^{4+} + 3\,O_2 + 16\,e^-$ | **1**

RED: $L(H)Br + 2\,e^- \rightleftharpoons L(H)^- + Br^-$ | **8**

Entry 30 – Dataset 0

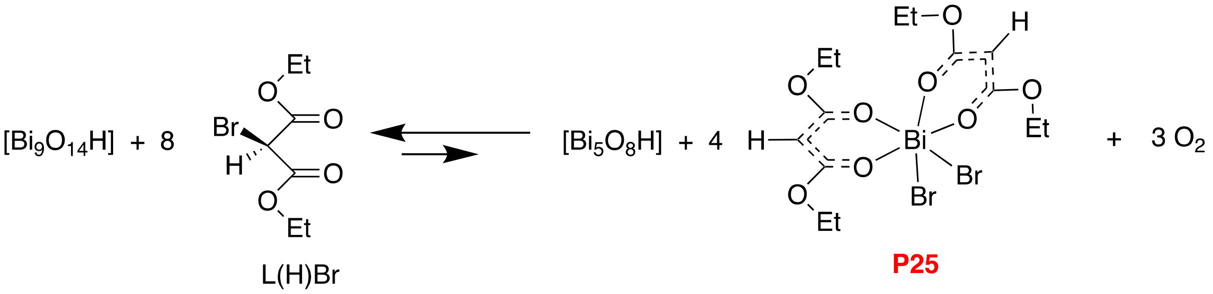

OX: $[Bi_9O_{14}H] \rightleftharpoons [Bi_5O_8H] + 4\,Bi^{4+} + 3\,O_2 + 16\,e^-$ | **1**

RED: $L(H)Br + 2\,e^- \rightleftharpoons L(H)^- + Br^-$ | **8**

Entry 31 – Dataset 1

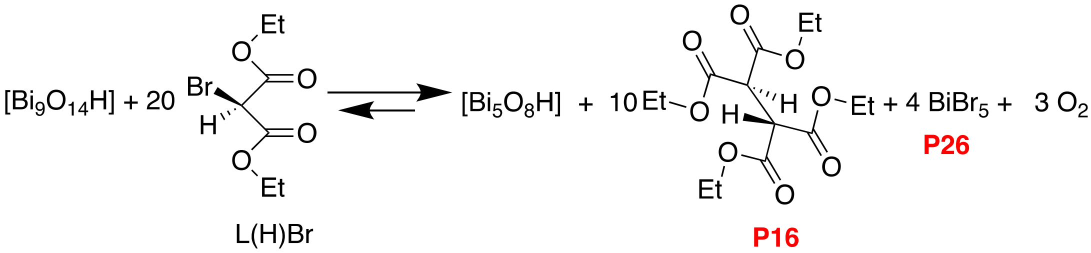

OX: $[Bi_9O_{14}H]$ + 20 L(H)$^{\bullet}$ ⇄ $[Bi_5O_8H]$ + 10 P16 + 4 Bi^{5+} + 3 O_2 + 20 e^- | 1

RED: $Br^{\bullet}$ + 1 e^- ⇄ Br^- | 20

Entry 32 – Dataset 2

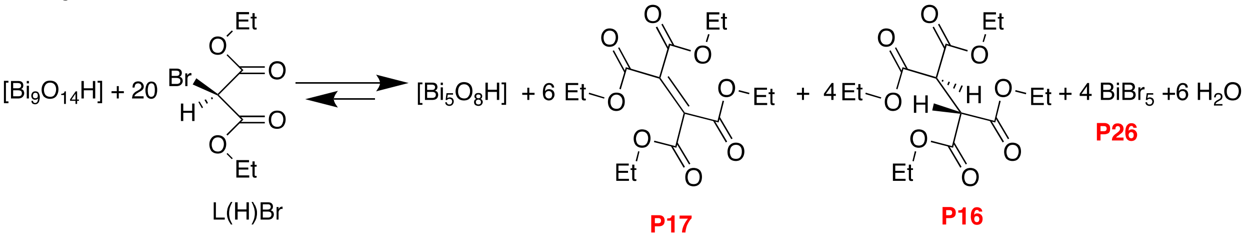

OX: $[Bi_9O_{14}H]$ + 20 L(H)$^{\bullet}$ ⇄ $[Bi_5O_8H]$ + 6 P17 + 4 P16 + 4 Bi^{5+} + 6 H_2O + 20 e^- | 1

RED: $Br^{\bullet}$ + 1 e^- ⇄ Br^- | 20

Entry 33 – Dataset 3

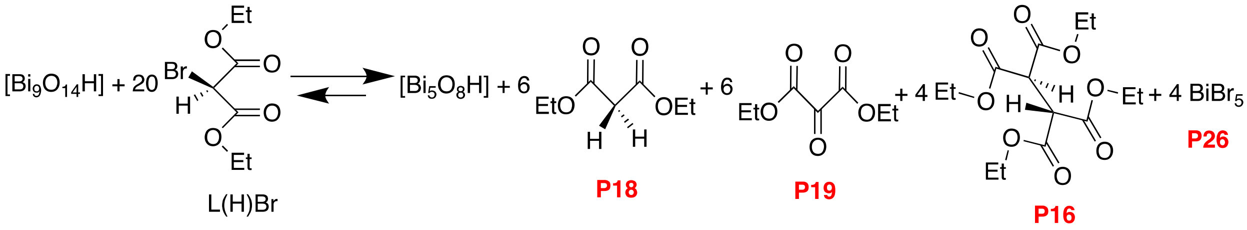

OX: $[Bi_9O_{14}H]$ + 20 L(H)$^{\bullet}$ ⇄ $[Bi_5O_8H]$ + 6 P18 + 6 P19 + 4 P16 + 4 Bi^{5+} + 20 e^- | 1

RED: $Br^{\bullet}$ + 1 e^- ⇄ Br^- | 20

Entry 34 – Dataset 1

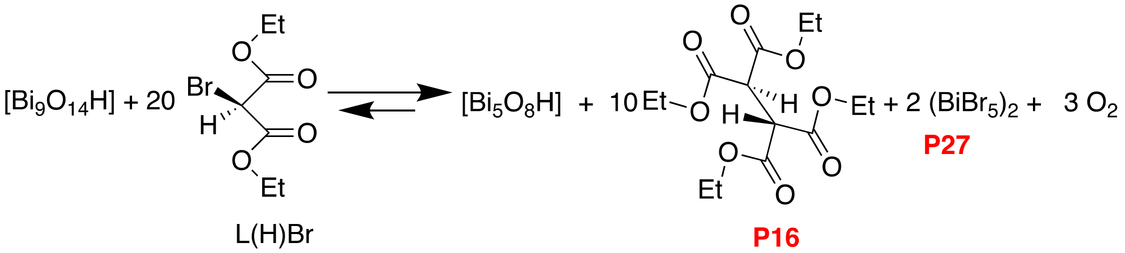

OX: $[Bi_9O_{14}H] + 20\ L(H)^{\bullet} \rightleftharpoons [Bi_5O_8H] + 10\ P16 + 4\ Bi^{5+} + 3\ O_2 + 20\ e^-$ | **1**

RED: $Br^{\bullet} + 1\ e^- \rightleftharpoons Br^-$ | **20**

Entry 35 – Dataset 2

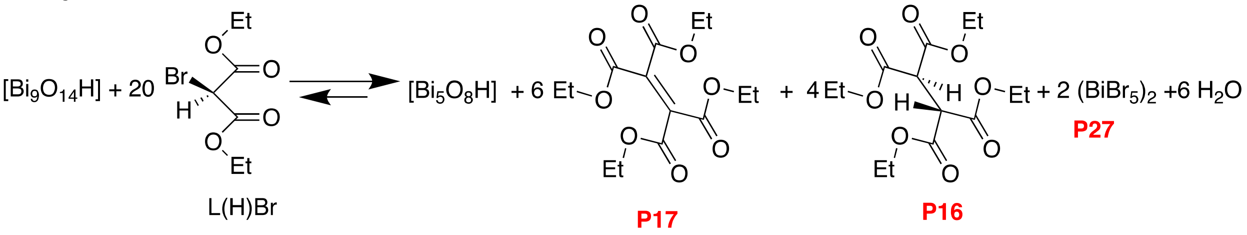

OX: $[Bi_9O_{14}H] + 20\ L(H)^{\bullet} \rightleftharpoons [Bi_5O_8H] + 6\ P17 + 4\ P16 + 4\ Bi^{5+} + 6\ H_2O + 20\ e^-$ | **1**

RED: $Br^{\bullet} + 1\ e^- \rightleftharpoons Br^-$ | **20**

Entry 36 – Dataset 3

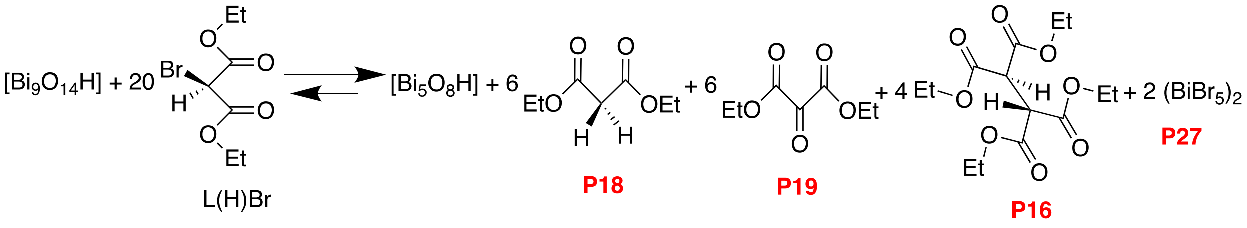

OX: $[Bi_9O_{14}H] + 20\ L(H)^{\bullet} \rightleftharpoons [Bi_5O_8H] + 6\ P18 + 6\ P19 + 4\ P16 + 4\ Bi^{5+} + 20\ e^-$ | **1**

RED: $Br^{\bullet} + 1\ e^- \rightleftharpoons Br^-$ | **20**

Entry 37 – Dataset 1

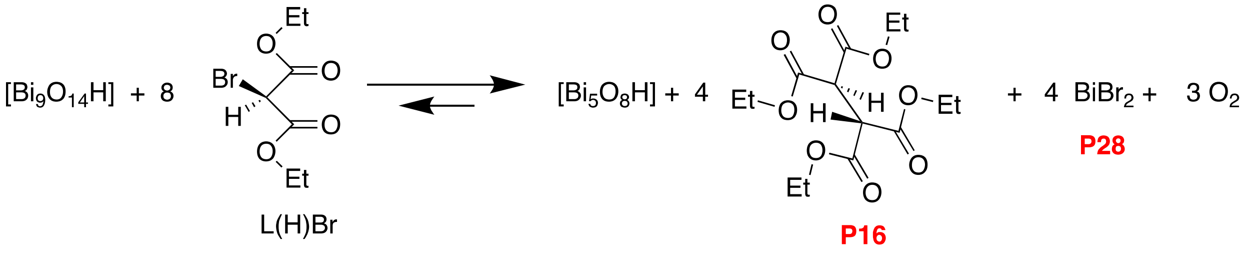

OX: $[Bi_9O_{14}H] \rightleftharpoons [Bi_5O_8H] + 4\ Bi^{2+} + 3\ O_2 + 8\ e^-$ | 1

RED: $2\ L(H)Br + 2\ e^- \rightleftharpoons P16 + 2\ Br^-$ | 4

Entry 38 – Dataset 2

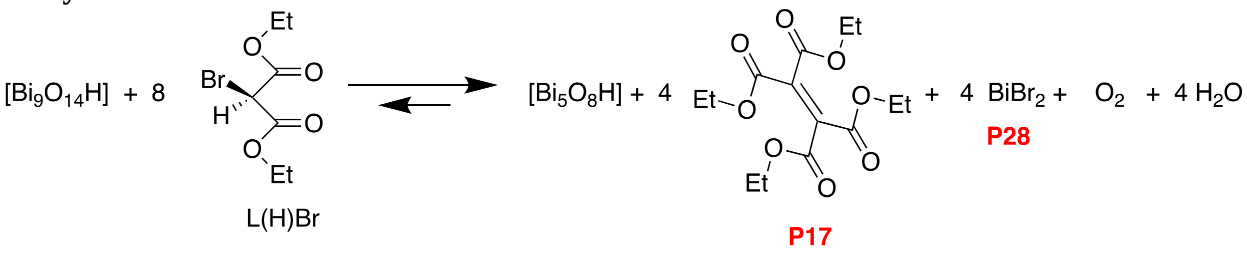

OX: $[Bi_9O_{14}H] + 8\ L(H)^{\bullet} \rightleftharpoons [Bi_5O_8H] + 4\ P17 + 4\ Bi^{2+} + 4\ H_2O + O_2 + 8\ e^-$ | 1

RED: $Br^{\bullet} + 1\ e^- \rightleftharpoons Br^-$ | 8

Entry 39 – Dataset 3

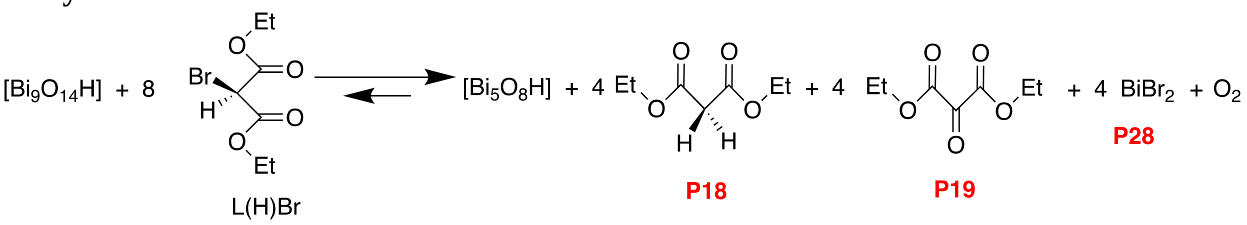

OX: $[Bi_9O_{14}H] + 8\ L(H)^{\bullet} \rightleftharpoons [Bi_5O_8H] + 6\ P18 + 6\ P19 + 4\ Bi^{2+} + O_2 + 8\ e^-$ | 1

RED: $Br^{\bullet} + 1\ e^- \rightleftharpoons Br^-$ | 8

Entry 40 – Dataset 1

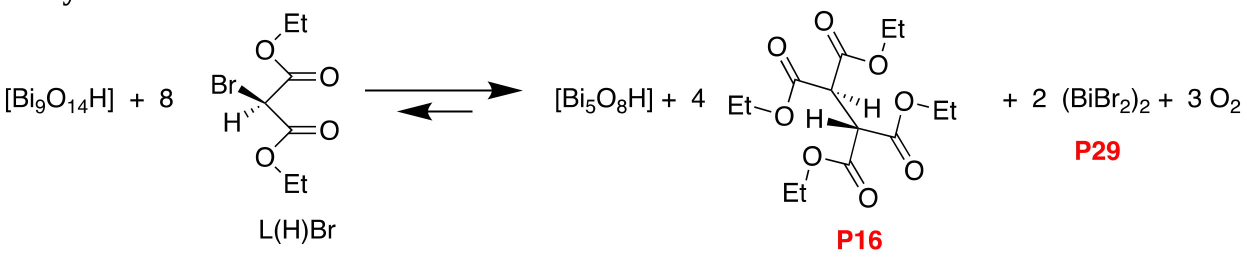

OX: $[Bi_9O_{14}H] \rightleftharpoons [Bi_5O_8H] + 4\ Bi^{2+} + 3\ O_2 + 8\ e^-$ | **1**

RED: $2\ L(H)Br + 2\ e^- \rightleftharpoons P16 + 2\ Br^-$ | **4**

Entry 41 – Dataset 2

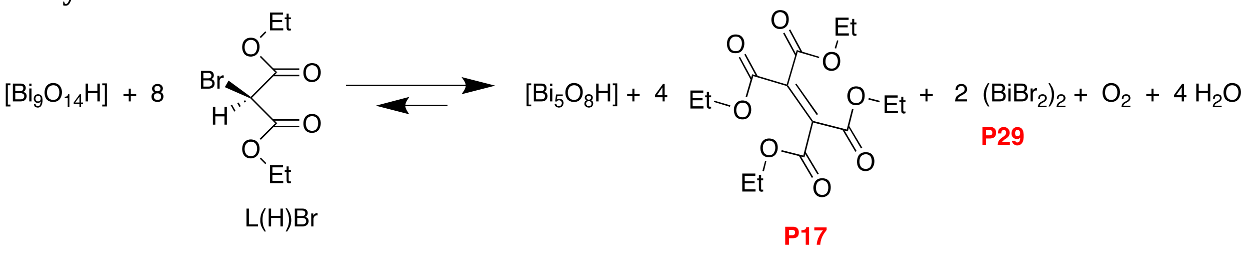

OX: $[Bi_9O_{14}H] + 8\ L(H)^{\bullet} \rightleftharpoons [Bi_5O_8H] + 4\ P17 + 4\ Bi^{2+} + 4\ H_2O + O_2 + 8\ e^-$ | **1**

RED: $Br^{\bullet} + 1\ e^- \rightleftharpoons Br^-$ | **8**

Entry 42 – Dataset 3

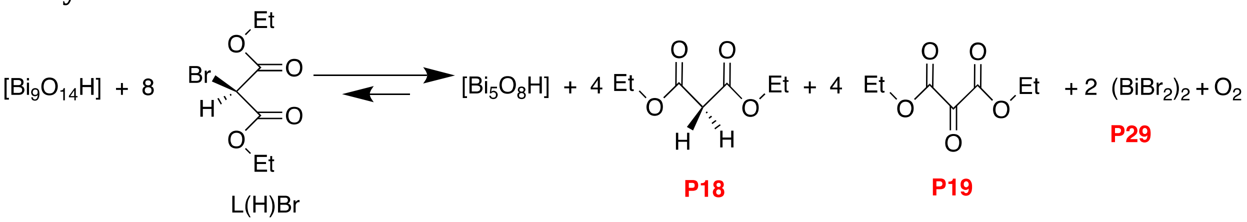

OX: $[Bi_9O_{14}H] + 8\ L(H)^{\bullet} \rightleftharpoons [Bi_5O_8H] + 6\ P18 + 6\ P19 + 4\ Bi^{2+} + O_2 + 8\ e^-$ | **1**

RED: $Br^{\bullet} + 1\ e^- \rightleftharpoons Br^-$ | **8**

Entry 43 – Dataset 1

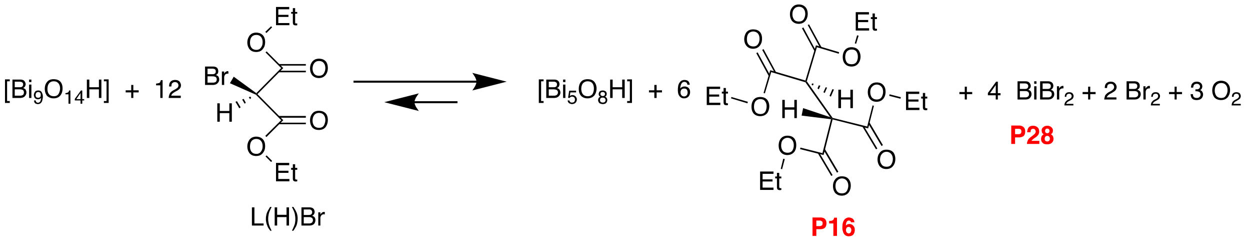

$$\textbf{OX:}\quad [Bi_9O_{14H}] \rightleftharpoons [Bi_5O_{8H}] + 4\,Bi^{2+} + 3\,O_2 + 8\,e^- \qquad \mathbf{1}$$

$$\textbf{RED:}\quad 12\ L(H)Br + 8\,e^- \rightleftharpoons 6\ P16 + 8\ Br^- + 2\,Br_2 \qquad \mathbf{1}$$

Entry 44 – Dataset 2

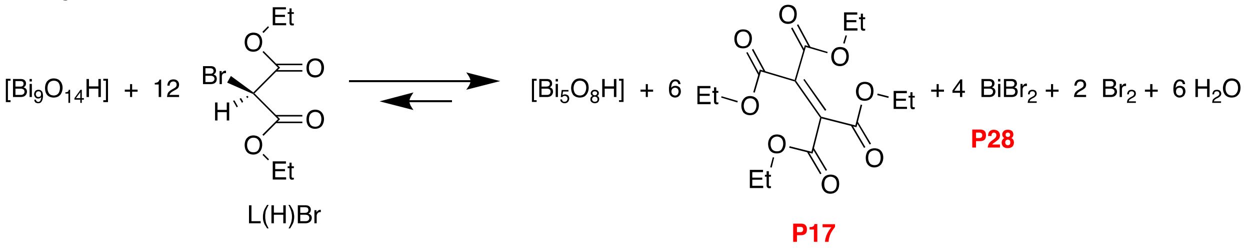

$$\textbf{OX:}\quad [Bi_9O_{14}H] + 12\ L(H)^{\bullet} \rightleftharpoons [Bi_5O_8H] + 6\ P17 + 4\,Bi^{2+} + 6\,H_2O + 8\,e^- \qquad \mathbf{1}$$

$$\textbf{RED:}\quad 6\ Br^{\bullet} + 4\,e^- \rightleftharpoons 4\ Br^- + Br_2 \qquad \mathbf{2}$$

Entry 45 – Dataset 3

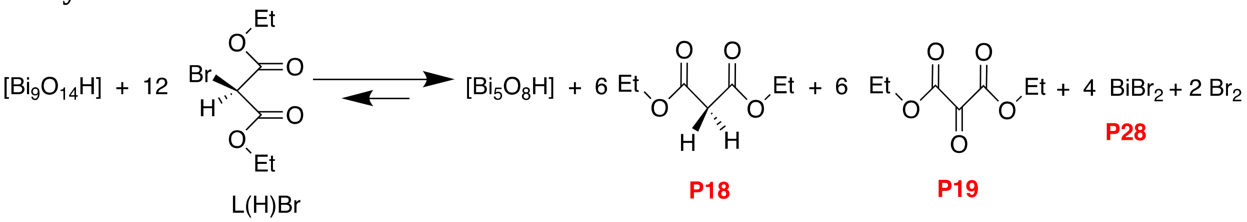

$$\textbf{OX:}\quad [Bi_9O_{14}H] + 12\ L(H)^{\bullet} \rightleftharpoons [Bi_5O_8H] + 6\ P18 + 6\ P19 + 4\,Bi^{2+} + 8\,e^- \qquad \mathbf{1}$$

$$\textbf{RED:}\quad 6\ Br^{\bullet} + 4\,e^- \rightleftharpoons 4\ Br^- + Br_2 \qquad \mathbf{2}$$

Entry 46 – Dataset 1

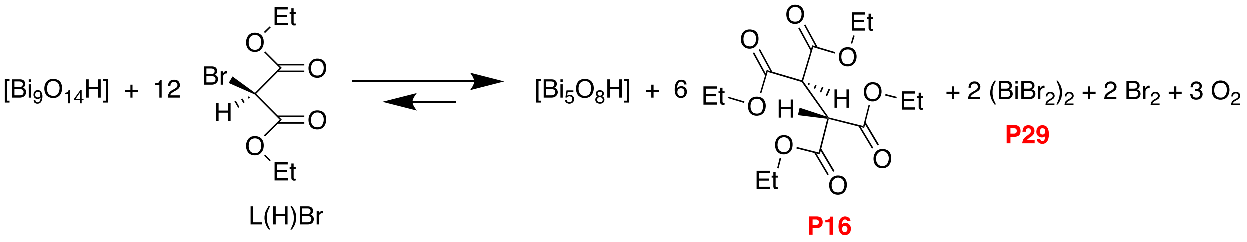

OX: $[Bi_9O_{14H}] \rightleftharpoons [Bi_5O_{8H}] + 4\ Bi^{2+} + 3\ O_2 + 8\ e^-$ **1**

RED: $12\ L(H)Br + 8\ e^- \rightleftharpoons 6\ P16 + 8\ Br^- + 2\ Br_2$ **1**

Entry 47 – Dataset 2

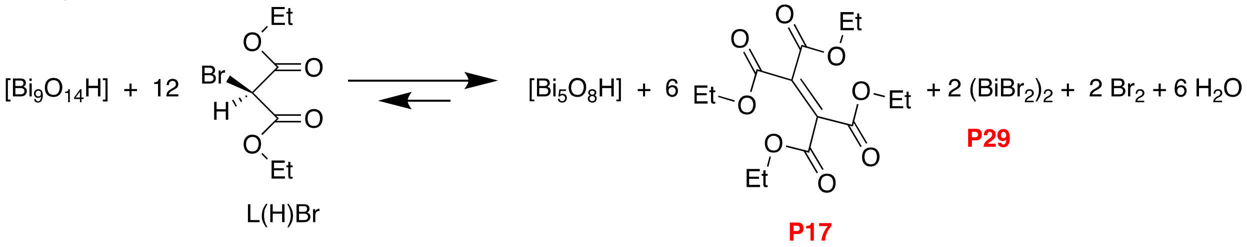

OX: $[Bi_9O_{14}H] + 12\ L(H)^\bullet \rightleftharpoons [Bi_5O_8H] + 6\ P17 + 4\ Bi^{2+} + 6\ H_2O + 8\ e^-$ **1**

RED: $6\ Br^\bullet + 4\ e^- \rightleftharpoons 4\ Br^- + Br_2$ **2**

Entry 48 – Dataset 3

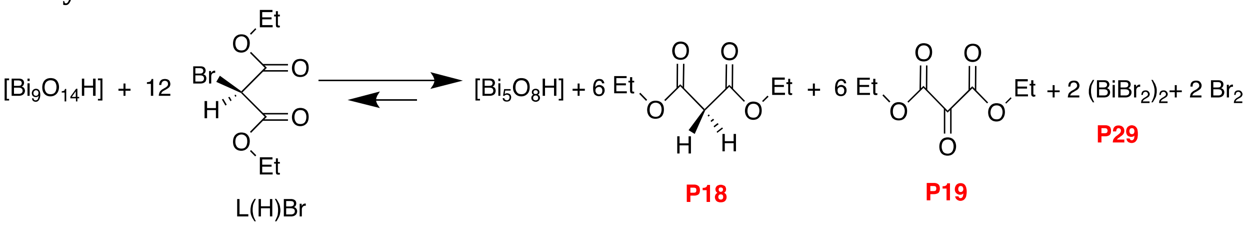

OX: $[Bi_9O_{14}H] + 12\ L(H)^\bullet \rightleftharpoons [Bi_5O_8H] + 6\ P18 + 6\ P19 + 4\ Bi^{2+} + 8\ e^-$ **1**

RED: $6\ Br^\bullet + 2\ e^- \rightleftharpoons 4\ Br^- + Br_2$ **2**

NOTE: The reaction schemes for **Entry 49** to **Entry 72** are equal to the reaction schemes for **Entries 16**, **17,** and **18** with the addition of *n* molecules of dimethyl sulfoxide (*n* varies by cases).

Supplementary Table 2| Entries 49 to 72 describe the Inclusion of the first layer of explicit solvation (dimethyl sulfoxide) around $BiBr_3$ moieties (SMD implicit solvation is still retained to model the dielectric of the continuum field).

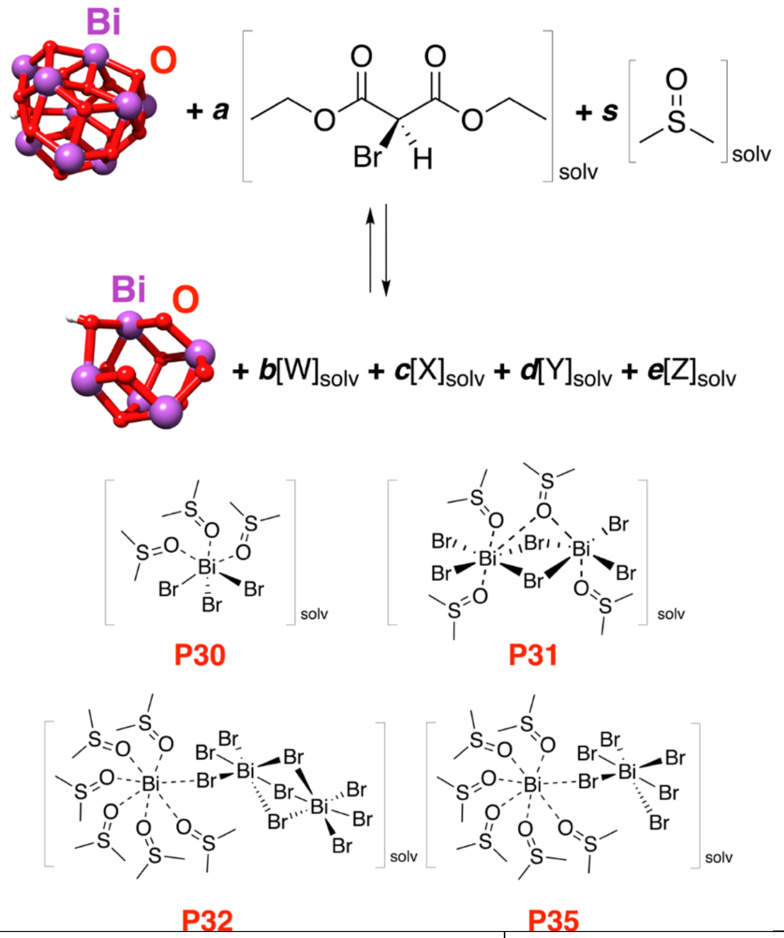

Entries	Coefficients[a]						Chemical species				ΔG^{0b}
	a	*s*	*b*	*c*	*d*	*e*	W	X	Y	Z	
E-49	3.00	3.00	1.00	0.00	1.5	0.75	P30	N/A	P16	O_2	**-11.7**
E-50	3.00	3.00	1.00	0.00	1.5	1.50	P30	N/A	P17	H_2O	**-53.0**
E-51	3.00	3.00	1.00	0.00	1.5	1.50	P30	N/A	P18	P19	**-59.6**
E-52	3.00	2.00	0.50	0.00	1.5	0.75	P31	N/A	P16	O_2	**-13.9**
E-53	3.00	2.00	0.50	0.00	1.5	1.50	P31	N/A	P17	H_2O	**-55.2**
E-54	3.00	2.00	0.50	0.00	1.5	1.50	P31	N/A	P18	P19	**-61.8**
E-55	3.00	2.25	0.25	0.25	1.5	0.75	P30	P32	P16	O_2	**-11.3**
E-56	3.00	2.25	0.25	0.25	1.5	1.50	P30	P32	P17	H_2O	**-52.6**
E-57	3.00	2.25	0.25	0.25	1.5	1.50	P30	P32	P18	P19	**-59.3**

E-58	3.00	2.50	0.25	0.25	1.5	0.75	P30	P33	P16	O_2	**-9.2**
E-59	3.00	2.50	0.25	0.25	1.5	1.50	P30	P33	P17	H_2O	**-50.5**
E-60	3.00	2.50	0.25	0.25	1.5	1.50	P30	P33	P18	P19	**-57.1**
E-61	3.00	2.75	0.25	0.25	1.5	0.75	P30	P34	P16	O_2	**-10.7**
E-62	3.00	2.75	0.25	0.25	1.5	1.50	P30	P34	P17	H_2O	**-52.0**
E-63	3.00	2.75	0.25	0.25	1.5	1.50	P30	P34	P18	P19	**-58.7**
E-64	3.00	3.00	0.50	0.00	1.5	0.75	P35	N/A	P16	O_2	**-6.4**
E-65	3.00	3.00	0.50	0.00	1.5	1.50	P35	N/A	P17	H_2O	**-47.8**
E-66	3.00	3.00	0.50	0.00	1.5	1.50	P35	N/A	P18	P19	**-54.4**
E-67	3.00	3.50	0.50	0.00	1.5	0.75	P36	N/A	P16	O_2	**+3.5**
E-68	3.00	3.50	0.50	0.00	1.5	1.50	P36	N/A	P17	H_2O	**-37.8**
E-69	3.00	3.50	0.50	0.00	1.5	1.50	P36	N/A	P18	P19	**-44.4**
E-70	3.00	4.00	0.50	0.00	1.5	0.75	P37	N/A	P16	O_2	**+0.6**
E-71	3.00	4.00	0.50	0.00	1.5	1.50	P37	N/A	P17	H_2O	**-40.7**
E-72	3.00	4.00	0.50	0.00	1.5	1.50	P37	N/A	P18	P19	**-47.3**

[a] The coefficients are valid per Bi equivalent (since four bismuth atoms are involved in the reactivity reported herein, the coefficient should be multiplied by 4 to find the overall change).

[b] Expressed in kcal·mol^{-1} per Bi equivalent, 1M standard state at 298 K.

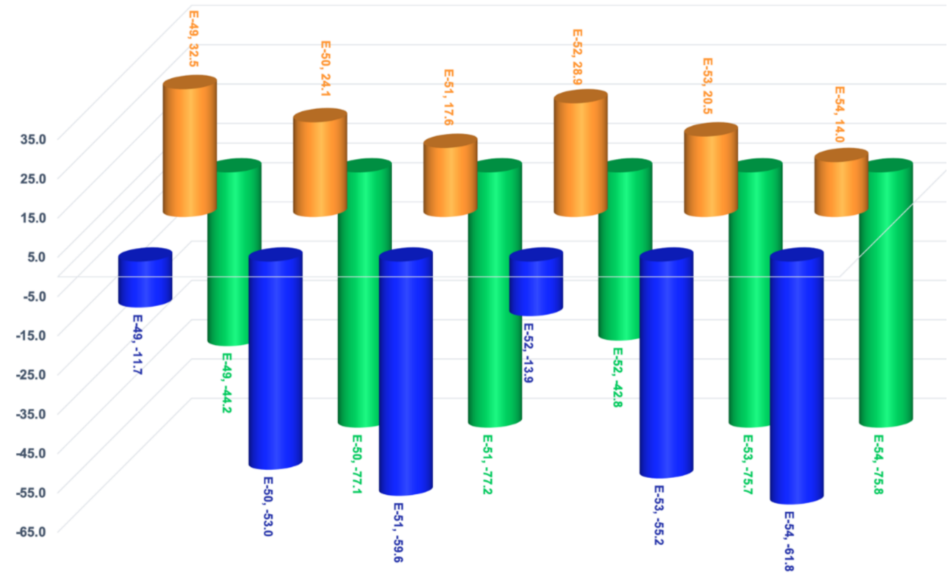

Supplementary Figure 16| Thermodynamics for the explicit solvation of $Bi^{III}Br_3$ with dimethyl sulfoxide (kcal·mol^{-1} vs Entries).

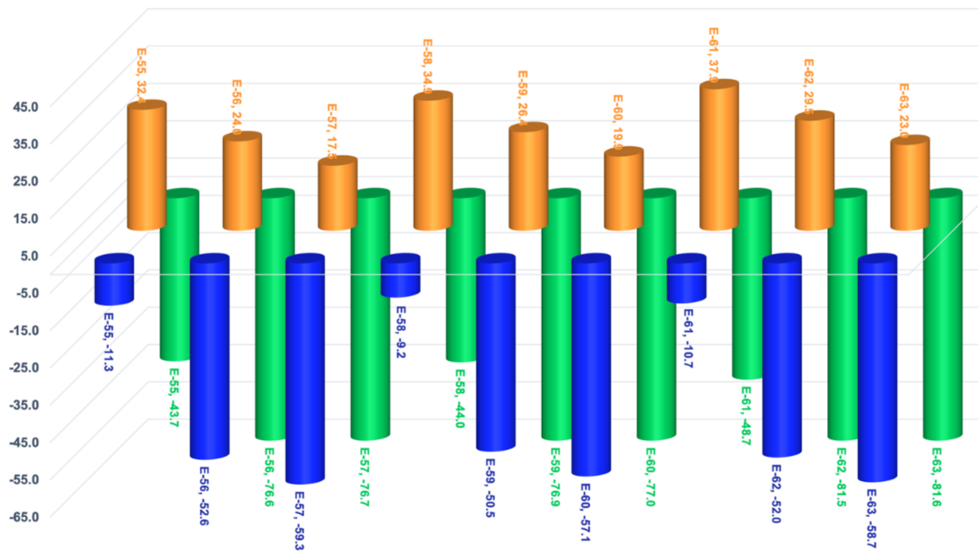

Supplementary Figure 17| Thermodynamics for the explicit solvation of complex $[Bi]^{3+}[Bi_2Br_9]^{3-}$ with dimethyl sulfoxide (kcal·mol^{-1} vs Entries).

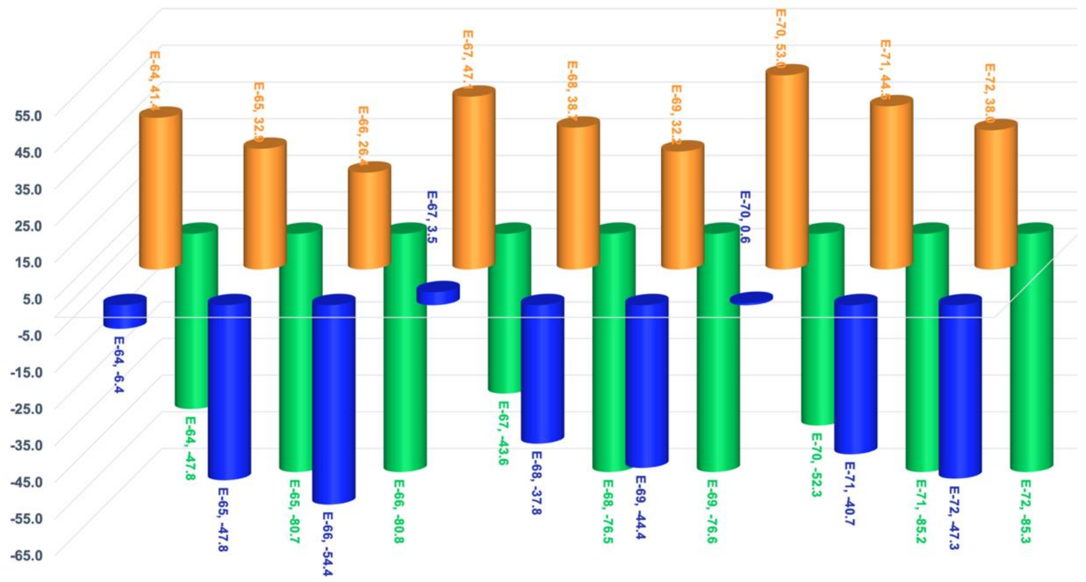

Supplementary Figure 18| Thermodynamics for the explicit solvation of complex $[Bi]^{3+}[BiBr_6]^{3-}$ with dimethyl sulfoxide (kcal·mol^{-1} vs Entries).

The introduction of explicit solvation in dimethyl sulfoxide (*e.g.*, discrete molecules of solvent saturating the coordination sphere of the metal) allows the isolation and stabilization of diverse monomeric and dimeric species of $BiBr_3$ (Supplementary Figures 21-24). Crystallographic evidence of the existence of these compounds has been reported by Bowmaker and co-workers.[16] Dimethyl sulfoxide acts as a ligand for the bismuth occupying three coordination positions to give the distorted *fac*-$[(Me_2S{=}O)_3BiBr_3]$ complex, that delivers an extra stabilization of ΔG^0 = -6.3 kcal·mol^{-1} to $BiBr_3$. Our computation affords an excellent reproduction of the experimental bond lengths with an identical value for the averaged Bi···O length, 2.47 Å, and slightly longer estimates for averaged S···O length (δ = +0.04 Å) and averaged Bi···Br length (δ = +0.11 Å). Dimeric $\{[(Me_2S{=}O)_3Bi_2Br_2](\mu^2\text{-}Br)_2(\mu^2\text{-}O{=}SMe_2)\}$ complex shows the highest stabilization due to solvation , ΔG^0 = -8.5 kcal·mol^{-1} per Bi atom. A very similar X-ray structure, $[(Me_2S{=}O)_4Bi_2I_2(\mu$□$\text{-}I)_2]$, has been already reported. The authors demonstrate that the coordinated molecules of DMSO are in *Trans* to each other and the Bi···Bi distance is 4.82 Å. The Bi···Bi distance in the calculated $\{[(Me_2S{=}O)_3Bi_2Br_2](\mu^2\text{-}Br)_2(\mu^2\text{-}O{=}SMe_2)\}$ complex is shorter, 4.12 Å, due to the shorter bridging capability provided by the

bromide (1.20 Å covalent radius) to the iodide (1.39 Å covalent radius). Unlike $[(Me_2S{=}O)_4Bi_2I_2(\mu^2\text{-}I)_2]$, the $X_2Bi(\mu^2\text{-}X)_2BiX_2$ unit in our calculated $\{[(Me_2S{=}O)_3Bi_2Br_2](\mu^2\text{-}Br)_2(\mu^2\text{-}O{=}SMe_2)\}$ structure strains away from planarity, bending at almost 120º, thus facilitating the formation of an asymmetric $\mu^2\text{-}O{=}SMe_2$ bridge between the Bi^{3+} ions. The computed averaged Bi···O length, 2.43 Å, is in line with that reported for $[(Me_2S{=}O)_4Bi_2I_2(\mu^2\text{-}I)_2]$, the former only marginally longer by $\delta = +0.04$ Å. We also calculated a planar $\{[(Me_2S{=}O)_4Bi_2Br_2](\mu^2\text{-}Br)_2\}$ arrangement, structurally very similar to $[(Me_2S{=}O)_4Bi_2I_2(\mu^2\text{-}I)_2]$, but the bent arrangement results to be thermodynamically more stable. The existence of closed-pair species formed by complex cations and anions, like $\{[(Me_2S{=}O)_6Bi](\mu^2\text{-}Br)[Bi_2Br_8]\}$, is also energetically very plausible, with a net stabilization of $\Delta G^0 = -5.9$ kcal·mol^{-1} per Bi atom. Bowmaker and co-workers also characterized crystallographically a similar iodide species, $\{[(Me_2S{=}O)_8Bi][Bi_2I_9]\}$, with eight molecules of dimethyl sulfoxide coordinated to the bismuth atom. Average Bi···Br bridging distances of the calculated $[Bi_2Br_9]^{3-}$ anion, 3.11 Å, agree well with the crystallographic average in $\{[(Me_4N)_3][Bi_2I_9]\}$, 3.06 Å. Our theory suggests that computed energetics of singlet-to-triplet electronic transitions for $\{[(Me_2S{=}O)_3Bi_2Br_2](\mu^2\text{-}Br)_2(\mu^2\text{-}O{=}SMe_2)\}$ complex agree qualitatively well with the experimental abs-max band: calculated transitions are at 323, 331, and 343 nm. Computed singlet-to-triplet transitions for *fac*-$[(Me_2S{=}O)_3BiBr_3]$ complex are 304 and 339 nm, also close to the experimental abs-max. Computed singlet-to-triplet transitions for $\{[(Me_2S{=}O)_6Bi](\mu^2\text{-}Br)[Bi_2Br_8]\}$ complex are 337, 339, 342, 344, 346, 353, 367, 372, 376 nm, which may also agree with the experimental abs-max. Computed singlet-to-triplet transitions for $\{[(CH_3)_3S]_3[(BiBr_6)]\}$ complex are 320, 317, and 314 nm: these values are also in line with the experimental abs-max.

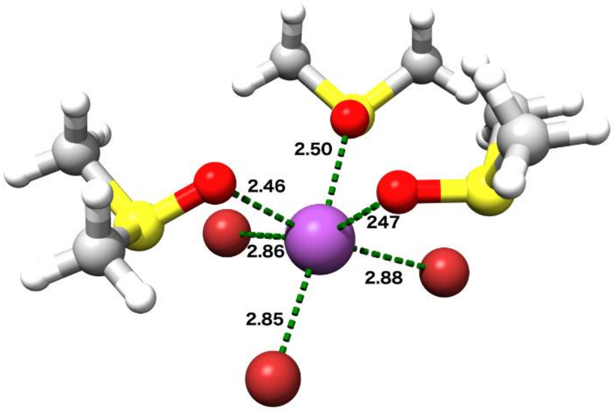

Supplementary Figure 19| Structure of *fac*-$(DMSO)_3BiBr_3$, P30 (calculated Bi•••X bond distances in Å).

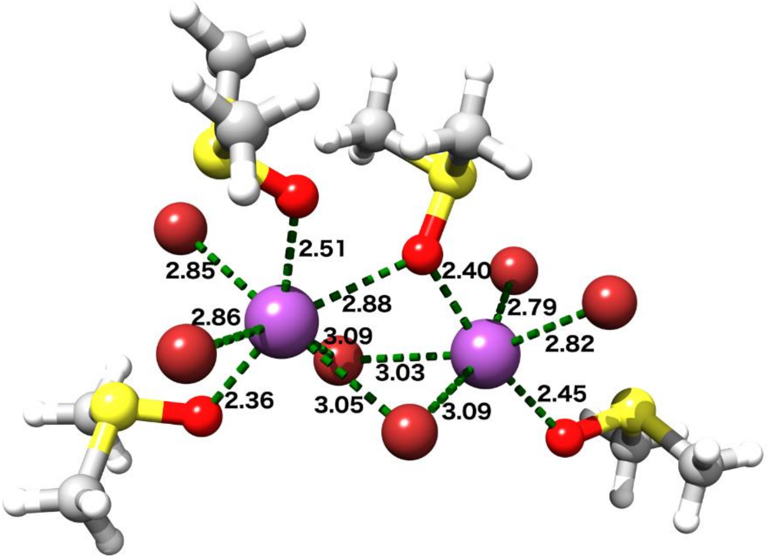

Supplementary Figure 20| Calculated structure of $[(DMSO)_3Bi_2Br_4(\mu^2\text{-}Br)_2(\mu^2\text{-}DMSO)]$,

P31 (calculated Bi•••X bond distances in Å).

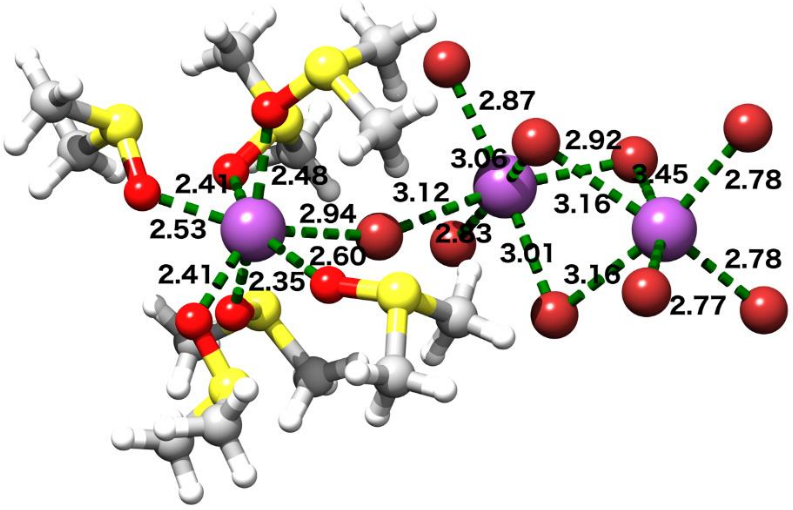

Supplementary Figure 21| Calculated structure of {[(DMSO)$_6$Bi)](μ^2-Br)[Bi$_2$Br$_5$(μ^2-Br)$_3$]}, P32 (calculated Bi•••X bond distances in Å).

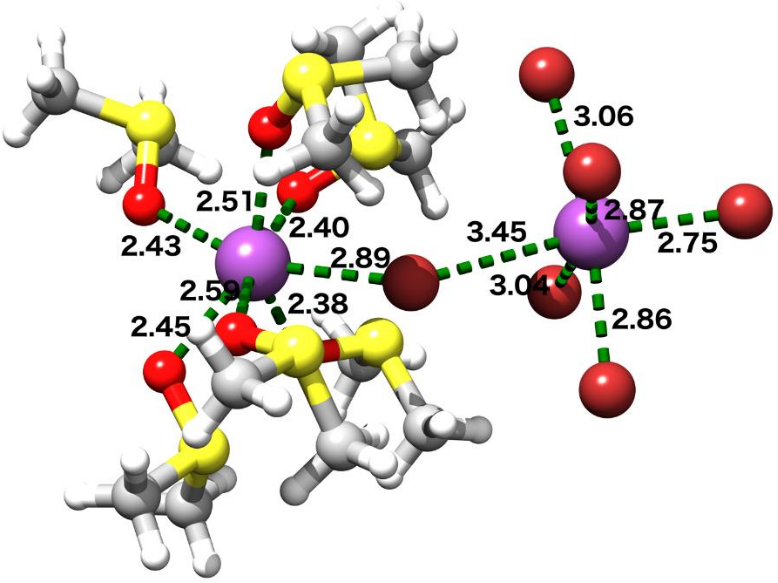

Supplementary Figure 22| Calculated structure of {[(DMSO)$_6$Bi](μ^2-Br)[BiBr$_5$]}, P35 (calculated Bi•••X bond distances in Å).

6. Supplementary references

1. Nguyen, J. D., Tucker, J. W., Konieczynska, M. D. & Stephenson, C. R. J. Intermolecular Atom Transfer Radical Addition to Olefins Mediated by Oxidative Quenching of Photoredox Catalysts. *J. Am. Chem. Soc.* **133**, 4160-4163 (2011).

2. Bruker, A. Bruker Advanced X-Ray Solutions Apex2 User Manual APEX2 v2014.9-0, Bruker AXS Inc., Madison, Wisconsin, USA (2014).

3. Sheldrick, G.M. SHELXT - Integrated Space-Group and Crystal-Structure Determination *Acta Cryst.* A**71**, 3-8 (2015).

4. Sheldrick, G. M. Crystal structure refinement with SHELXL. *Acta Cryst.* **C71**, 3-8 (2015).

5. Gaussian 09, Revision D.01, Frisch, M. J.; Trucks, G. W.; Schlegel, H. B.; Scuseria, G. E.; Robb, M. A.; Cheeseman, J. R.; Scalmani, G.; Barone, V.; Petersson, G. A.; Nakatsuji, H.; Li, X.; Caricato, M.; Marenich, A. V.; Bloino, J.; Janesko, B. G.; Gomperts, R.; Mennucci, B.; Hratchian, H. P.; Ortiz, J. V.; Izmaylov, A. F.; Sonnenberg, J. L.; Williams-Young, D.; Ding, F.; Lipparini, F.; Egidi, F.; Goings, J.; Peng, B.; Petrone, A.; Henderson, T.; Ranasinghe, D.; Zakrzewski, V. G.; Gao, J.; Rega, N.; Zheng, G.; Liang, W.; Hada, M.; Ehara, M.; Toyota, K.; Fukuda, R.; Hasegawa, J.; Ishida, M.; Nakajima, T.; Honda, Y.; Kitao, O.; Nakai, H.; Vreven, T.; Throssell, K.; Montgomery, J. A., Jr.; Peralta, J. E.; Ogliaro, F.; Bearpark, M. J.; Heyd, J. J.; Brothers, E. N.; Kudin, K. N.; Staroverov, V. N.; Keith, T. A.; Kobayashi, R.; Normand, J.; Raghavachari, K.; Rendell, A. P.; Burant, J. C.; Iyengar, S. S.; Tomasi, J.; Cossi, M.; Millam, J. M.; Klene, M.; Adamo, C.; Cammi, R.; Ochterski, J. W.; Martin, R. L.; Morokuma, K.; Farkas, O.; Foresman, J. B.; Fox, D. J. Gaussian, Inc., Wallingford CT.

6. Zhao, Y. & Truhlar, D. G. A New Local Density Functional for Main-Group Thermochemistry, Transition Metal Bonding, Thermochemical Kinetics, and Noncovalent Interactions. *J. Chem. Phys.* **125**, 194101, (2006).

7. Zhao, Y. & Truhlar, D. G. Applications and Validations of the Minnesota Density Functionals. *Chem. Phys. Lett.* **502**, 1-13 (2011).

8. Grimme, S., Antony, J., Ehrlich, S. & Krieg, H. A Consistent and Accurate Ab Initio Parametrization of Density Functional Dispersion Correction (DFT-D) for the 94 Elements H-Pu. *J. Chem. Phys.* **132**, 154104, (2010).

9. Weigend, F. & Ahlrichs, R. Balanced Basis Sets of Split Valence, Triple Zeta Valence and Quadruple Zeta Valence Quality for H to Rn: Design and Assessment of Accuracy. *Phys. Chem. Chem. Phys.* **7**, 3297-3305, (2005).

10. Marenich, A. V., Cramer, C. J. & Truhlar, D. G. Universal Solvation Model Based on Solute Electron Density and a Continuum Model of the Solvent Defined by the Bulk Dielectric Constant and Atomic Surface Tensions. *J. Phys. Chem. B*, **113**, 6378-6396 (2009).

11. Funes-Ardoiz, I. & Paton, R. GoodVibes, v.2.0.3. DOI: 10.5281/zenodo.1435820.

12. Grimme, S. Supramolecular Binding Thermodynamics by Dispersion-Corrected Density Functional Theory. *Chem. Eur. J.* **18**, 9955-9964, (2012).

13. Wheeler, S. E. & Houk, K. N. Integration Grid Errors for Meta-GGA-Predicted Reaction Energies: Origin of Grid Errors for the M06 Suite of Functionals. *J. Chem. Theory Comput.* **6**, 395-404, (2010).

14. Pettersen, E. F. et al. UCSF Chimera - A visualization system for exploratory research and analysis. *J. Comput. Chem.* **25**, 1605-1612, (2004).

15. Rogow, D. L. et al. Hydrothermal Synthesis of Two Cationic Bismuthate Clusters: An Alkylenedisulfonate Bridged Hexamer, $[Bi_6O_4(OH)_4(H_2O)_2][(CH_2)_2(SO_3)_2]_3$ and a Rare Nonamer Templated by Triflate, $[Bi_9O_8(OH)_6][CF_3SO_3]_5$. *Inorg. Chem.* **49**, 5619-5624, (2010).

16. Bowmaker, G. A., Harrowfield, J. M., Junk, P. C., Skelton, B. W. & Whiteet, A. H. Syntheses, Structures and Vibrational Spectra of Some Dimethyl Sulfoxide Solvates of Bismuth(III) Bromide and Iodide. *Austr. J. Chem.* **51**, 285-292, (1998).
