# Supplementary material for: Shedding light on the nature of the catalytically active species in photocatalytic reactions using Bi2O3 semiconductor
Source: Nat Commun. 2021 Jan 27;12:625. doi: 10.1038/s41467-020-20882-x (PMC7841156; doi:10.1038/s41467-020-20882-x)
Supplement: Supplementary file 3 — Description of Additional Supplementary Files [file 41467_2020_20882_MOESM3_ESM.pdf]

**Description of Additional Supplementary Files**

File Name: Supplementary Data 1
Description: Supplementary Data 1 contains atomic cartesian coordinates, harmonic frequencies, RRHO-corrected and noncorrected energies for all the stationary points reported in the present work.
