# Supplementary material for: Shedding light on the nature of the catalytically active species in photocatalytic reactions using Bi2O3 semiconductor
Source: Nat Commun. 2021 Jan 27;12:625. doi: 10.1038/s41467-020-20882-x (PMC7841156; doi:10.1038/s41467-020-20882-x)
Supplement: Supplementary file 4 — Supplementary Data 1 [file 41467_2020_20882_MOESM4_ESM.zip › Rev_Supplementary Data.docx]

**Supplementary Data 1**

**1. Cartesian coordinates, frequencies, and non-corrected absolute energies**

Unless otherwise stated, each stationary point has been calculated in DMSO (SMD**).**

**H_2_O (in H_2_O)**

Center Atomic Atomic Coordinates (Angstroms)

Number Number Type X Y Z

---------------------------------------------------------------------

1 8 0 0.077115 1.232839 2.786662

2 1 0 0.900897 1.733174 2.786662

3 1 0 -0.583333 1.934882 2.786662

---------------------------------------------------------------------

Frequencies -- 1675.3004 3841.0206 3934.9165

SCF Done: E(RM06L-D3/def2-SVP/SMD) = -76.3560356569

Sum of electronic and zero-point Energies= -76.334504

Sum of electronic and thermal Energies= -76.331669

Sum of electronic and thermal Free Energies= -76.352817

SCF Done: E(RM06L-D3/def2-TZVPP/SMD) = -76.4540265038

**Bi(OH)_3_ (in H_2_O)**

Center Atomic Atomic Coordinates (Angstroms)

Number Number Type X Y Z

---------------------------------------------------------------------

1 83 0 -1.819959 1.879934 -2.110927

2 8 0 -2.427015 0.111945 -1.098195

3 1 0 -2.237666 0.232969 -0.156703

4 8 0 -0.389563 2.405143 -0.639400

5 1 0 0.441354 1.934120 -0.788691

6 8 0 -3.141108 3.246111 -1.176477

7 1 0 -2.823337 3.369845 -0.270040

---------------------------------------------------------------------

Frequencies -- 102.8569 142.2732 161.5445

Frequencies -- 219.1582 258.1355 311.1817

Frequencies -- 498.1111 507.5698 537.0108

Frequencies -- 798.0123 834.9558 857.2884

Frequencies -- 3818.8392 3829.1207 3850.1719

SCF Done: E(RM06L-D3/def2-SVP/SMD) = -442.071582283

Sum of electronic and zero-point Energies= -442.033477

Sum of electronic and thermal Energies= -442.026369

Sum of electronic and thermal Free Energies= -442.065896

SCF Done: E(RM06L-D3/def2-TZVPP/SMD) = -442.374943873

**EDTA-H_4_ (in H_2_O)**

Center Atomic Atomic Coordinates (Angstroms)

Number Number Type X Y Z

---------------------------------------------------------------------

1 7 0 -0.201548 -2.071740 0.662944

2 6 0 -1.656597 -2.067679 0.708335

3 6 0 0.431115 -1.427728 1.796380

4 6 0 0.402423 -3.313414 0.253682

5 6 0 -2.255974 -1.910761 -0.676935

6 1 0 -1.964968 -1.227974 1.350191

7 1 0 -2.060728 -2.980030 1.192113

8 1 0 -0.085059 -1.598191 2.757449

9 1 0 1.449360 -1.823641 1.938197

10 1 0 -0.247645 -3.837177 -0.464529

11 1 0 0.553514 -4.040444 1.076090

12 1 0 -1.914522 -0.953700 -1.122502

13 1 0 -1.855278 -2.700667 -1.332580

14 6 0 -4.228784 -2.472961 -1.967913

15 6 0 -4.429111 -0.943233 -0.088023

16 1 0 -3.669377 -2.078677 -2.834394

17 1 0 -5.262138 -2.117171 -2.101592

18 1 0 -3.889622 -0.562876 0.792310

19 1 0 -4.549281 -0.062332 -0.749096

20 7 0 -3.705305 -2.037894 -0.686111

21 6 0 -5.785310 -1.389184 0.387312

22 8 0 -6.182814 -2.531775 0.362715

23 8 0 -6.501615 -0.357278 0.848206

24 6 0 -4.284937 -3.984459 -2.067577

25 8 0 -4.222926 -4.606738 -0.887828

26 8 0 -4.403421 -4.585192 -3.110213

27 6 0 0.586304 0.065742 1.585240

28 8 0 0.731380 0.862916 2.482607

29 8 0 0.583858 0.435987 0.301923

30 6 0 1.717123 -3.072680 -0.438502

31 8 0 2.341274 -4.222066 -0.715643

32 8 0 2.157325 -1.979542 -0.716310

33 1 0 -4.170774 -3.881996 -0.219118

34 1 0 0.494584 -0.397715 -0.215674

35 1 0 3.169693 -4.013304 -1.179711

36 1 0 -7.358476 -0.698905 1.155380

---------------------------------------------------------------------

Frequencies -- 10.8226 20.6211 34.1935

Frequencies -- 37.3408 45.3990 56.3435

Frequencies -- 69.0326 76.8810 81.4120

Frequencies -- 89.1631 91.4735 169.5536

Frequencies -- 176.9244 219.3679 231.5672

Frequencies -- 232.9812 243.0361 326.1078

Frequencies -- 344.5136 351.2374 380.6669

Frequencies -- 388.4612 430.3179 465.9078

Frequencies -- 509.8412 519.9396 526.0093

Frequencies -- 547.2085 565.3251 574.3944

Frequencies -- 599.8476 651.0052 667.9372

Frequencies -- 671.9373 678.7024 680.0398

Frequencies -- 682.0047 731.3956 779.7585

Frequencies -- 787.8925 888.3378 888.9778

Frequencies -- 905.7504 910.8382 937.2492

Frequencies -- 948.8378 988.2919 991.1020

Frequencies -- 993.3310 1004.6802 1084.3444

Frequencies -- 1101.6186 1147.2351 1151.2858

Frequencies -- 1168.2293 1172.6345 1183.7621

Frequencies -- 1224.0890 1227.3229 1237.4837

Frequencies -- 1262.8626 1288.2982 1293.6796

Frequencies -- 1296.3541 1299.5900 1330.7388

Frequencies -- 1343.2388 1352.1634 1359.3769

Frequencies -- 1378.3212 1384.7863 1394.7356

Frequencies -- 1402.0327 1408.9436 1410.4905

Frequencies -- 1418.2003 1429.7223 1443.9069

Frequencies -- 1446.9474 1451.4078 1469.1998

Frequencies -- 1472.7810 1824.9420 1828.7598

Frequencies -- 1853.2092 1855.2592 2989.3956

Frequencies -- 3003.0568 3016.6354 3021.1738

Frequencies -- 3049.3487 3058.0635 3098.6854

Frequencies -- 3113.0012 3126.3552 3128.3645

Frequencies -- 3131.7916 3134.9870 3477.0400

Frequencies -- 3508.7008 3753.7913 3758.3124

SCF Done: E(RM06L-D3/def2-SVP/SMD) = -1101.12628530

Sum of electronic and zero-point Energies= -1100.841581

Sum of electronic and thermal Energies= -1100.820932

Sum of electronic and thermal Free Energies= -1100.896082

SCF Done: E(RM06L-D3/def2-TZVPP/SMD) = -1102.35631729

**[(*κ*^6^-EDTA-H)Bi(OH_2_)] (in H_2_O)**

Center Atomic Atomic Coordinates (Angstroms)

Number Number Type X Y Z

---------------------------------------------------------------------

1 83 0 0.112185 0.538195 -0.107006

2 7 0 -0.004889 -2.094163 0.235648

3 6 0 -1.418984 -2.519366 0.277492

4 6 0 0.682991 -2.361863 1.508080

5 6 0 0.730891 -2.715905 -0.863692

6 6 0 -2.313309 -1.747748 -0.665378

7 1 0 -1.774326 -2.391063 1.310736

8 1 0 -1.509095 -3.599248 0.070110

9 1 0 -0.017243 -2.160486 2.331992

10 1 0 0.966589 -3.423720 1.596203

11 1 0 0.541603 -3.800584 -0.936007

12 1 0 1.814930 -2.618433 -0.680852

13 1 0 -2.035308 -1.900062 -1.716688

14 1 0 -3.341475 -2.137684 -0.570489

15 6 0 -2.946612 0.001943 0.902824

16 6 0 -2.827544 0.511653 -1.466759

17 1 0 -3.790037 -0.682872 1.083408

18 1 0 -3.384701 1.010221 0.848720

19 1 0 -2.392496 0.162422 -2.417320

20 1 0 -3.919839 0.394444 -1.558377

21 7 0 -2.284257 -0.305478 -0.376219

22 6 0 -2.483641 1.994815 -1.300972

23 8 0 -1.457687 2.257482 -0.560900

24 8 0 -3.167387 2.825258 -1.888916

25 6 0 -2.044434 0.015694 2.125987

26 8 0 -0.789592 0.312126 1.924958

27 8 0 -2.517147 -0.188913 3.231406

28 6 0 1.908627 -1.486391 1.763803

29 8 0 1.970809 -0.380770 1.101133

30 8 0 2.718269 -1.859331 2.605330

31 6 0 0.501848 -2.064163 -2.194386

32 8 0 0.114564 -0.912657 -2.331472

33 8 0 0.822872 -2.848393 -3.202034

34 1 0 0.728510 -2.354554 -4.037042

35 8 0 1.224724 2.194785 1.493496

36 1 0 0.619921 2.203208 2.252296

37 1 0 1.887664 1.523281 1.748367

---------------------------------------------------------------------

Frequencies -- 33.7078 50.6077 60.7440

Frequencies -- 69.0236 78.7052 94.7266

Frequencies -- 105.1978 118.2013 126.3724

Frequencies -- 144.6372 149.7425 159.0046

Frequencies -- 185.5612 189.4181 198.4170

Frequencies -- 207.4828 221.3307 238.8642

Frequencies -- 253.8031 266.5303 272.2363

Frequencies -- 283.5022 306.3526 362.7796

Frequencies -- 365.9508 373.3201 390.6217

Frequencies -- 426.9560 438.3825 472.1389

Frequencies -- 488.1292 512.6139 549.6082

Frequencies -- 560.7567 561.6051 575.0766

Frequencies -- 599.4577 623.5513 650.7688

Frequencies -- 660.4593 695.4557 704.0955

Frequencies -- 717.6973 724.1157 750.7776

Frequencies -- 849.7045 878.8926 925.1913

Frequencies -- 930.9518 936.7836 942.0893

Frequencies -- 971.9222 987.4488 990.2750

Frequencies -- 998.3995 1015.4210 1039.3815

Frequencies -- 1077.5919 1124.9406 1132.5758

Frequencies -- 1141.1183 1193.9596 1202.7832

Frequencies -- 1235.7477 1262.6338 1276.2396

Frequencies -- 1282.2151 1302.7825 1320.1188

Frequencies -- 1322.2916 1329.2648 1333.8849

Frequencies -- 1344.1158 1357.6219 1363.4437

Frequencies -- 1374.0156 1375.9634 1402.5314

Frequencies -- 1414.7113 1434.0358 1445.7740

Frequencies -- 1446.6645 1468.1285 1468.2639

Frequencies -- 1482.7408 1651.9932 1748.3615

Frequencies -- 1753.1947 1769.1525 1778.7656

Frequencies -- 3055.5841 3056.3027 3060.5840

Frequencies -- 3063.9074 3069.3517 3075.2030

Frequencies -- 3121.6159 3132.4870 3135.7142

Frequencies -- 3147.6471 3149.3083 3157.4722

Frequencies -- 3649.7624 3728.0947 3811.0334

SCF Done: E(RM06L-D3/def2-SVP/SMD) = -1390.55369779

Sum of electronic and zero-point Energies= -1390.276863

Sum of electronic and thermal Energies= -1390.254311

Sum of electronic and thermal Free Energies= -1390.328695

SCF Done: E(RM06L-D3/def2-TZVPP/SMD) = -1391.87887314

**[Bi_9_O_13_(OH)]**

Center Atomic Atomic Coordinates (Angstroms)

Number Number Type X Y Z

---------------------------------------------------------------------

1 83 0 1.816744 -2.013877 -0.961184

2 83 0 1.816062 2.014468 -0.961253

3 83 0 1.760492 0.000319 2.164023

4 83 0 -0.140826 -0.000061 -3.611453

5 83 0 -0.072730 -3.163341 1.839411

6 8 0 1.602673 0.000274 -0.076290

7 8 0 0.059994 -1.684434 -2.323562

8 8 0 2.136397 0.000330 -2.784718

9 8 0 -0.013453 -3.146583 -0.345568

10 8 0 1.895212 -2.232592 1.278375

11 8 0 1.894516 2.233361 1.278326

12 8 0 -0.029792 -1.221755 2.806847

13 83 0 -1.799596 1.886770 -1.048996

14 83 0 -1.798947 -1.887320 -1.048938

15 83 0 -1.824108 -0.000253 2.184039

16 83 0 -0.073819 3.163317 1.839328

17 8 0 -1.339838 -0.000180 0.028205

18 8 0 0.059408 1.684464 -2.323644

19 8 0 -2.044148 -0.000460 -2.548493

20 8 0 -0.014509 3.146554 -0.345632

21 8 0 -1.984948 2.222256 1.266050

22 8 0 -1.984296 -2.223188 1.266146

23 8 0 -0.030186 1.221804 2.806826

24 1 0 2.842684 0.000548 -3.443138

---------------------------------------------------------------------

Frequencies -- 58.4219 60.9391 71.8915

Frequencies -- 74.2552 79.2210 84.3222

Frequencies -- 86.8565 91.1993 91.6980

Frequencies -- 96.6886 97.4501 105.8948

Frequencies -- 114.8864 125.4313 134.5312

Frequencies -- 140.2307 146.9198 150.6949

Frequencies -- 156.9462 173.8835 174.9641

Frequencies -- 178.2165 178.4659 202.4138

Frequencies -- 224.7019 235.9418 243.6898

Frequencies -- 252.7183 256.8509 265.6483

Frequencies -- 277.2434 287.5364 291.9579

Frequencies -- 302.0822 306.9372 312.3165

Frequencies -- 315.4572 326.3019 328.3178

Frequencies -- 342.6487 350.9345 378.0702

Frequencies -- 382.5355 389.8545 411.3060

Frequencies -- 417.2754 441.6974 465.3313

Frequencies -- 471.4718 478.3529 488.6685

Frequencies -- 491.0228 505.0893 512.9745

Frequencies -- 529.2434 548.0217 559.6951

Frequencies -- 565.9736 586.0278 587.4006

Frequencies -- 587.4776 611.7257 659.1018

Frequencies -- 667.0795 683.9220 3848.7245

SCF Done: E(RM06L-D3/def2-SVP/SMD) = -2986.23029241

Sum of electronic and zero-point Energies= -2986.175467

Sum of electronic and thermal Energies= -2986.143155

Sum of electronic and thermal Free Energies= -2986.240723

SCF Done: E(RM06L-D3/def2-TZVPP/SMD) = -2987.60315425

**[Bi_5_O_7_(OH)]**

Center Atomic Atomic Coordinates (Angstroms)

Number Number Type X Y Z

---------------------------------------------------------------------

1 83 0 0.053732 -0.482810 0.113691

2 83 0 0.201540 3.002341 0.343879

3 83 0 0.272571 3.106151 -3.301627

4 83 0 0.145113 -0.386992 -3.301572

5 8 0 1.574467 -0.477285 -1.555446

6 8 0 1.733710 1.408741 -3.436182

7 8 0 1.499471 3.152057 -1.449408

8 8 0 1.646838 1.288628 0.443088

9 83 0 2.777262 1.331213 -1.472233

10 8 0 -1.021018 3.945794 -1.474493

11 8 0 -1.056392 1.357208 0.091430

12 8 0 -0.969341 -1.158890 -1.643577

13 8 0 -0.984352 1.441750 -3.245013

14 1 0 -0.914130 4.907615 -1.451333

---------------------------------------------------------------------

Frequencies -- 53.6333 81.5795 93.9441

Frequencies -- 95.5437 108.6244 115.3196

Frequencies -- 131.8808 145.1512 158.1314

Frequencies -- 163.1942 177.7157 190.0270

Frequencies -- 227.9034 249.7313 271.4451

Frequencies -- 280.7891 304.7143 326.6454

Frequencies -- 365.1755 368.7435 397.8556

Frequencies -- 423.6268 436.2531 445.5024

Frequencies -- 463.4610 475.6589 495.8102

Frequencies -- 498.9626 517.8032 528.0214

Frequencies -- 554.5712 600.8197 609.2147

Frequencies -- 611.6717 787.0111 3822.6671

SCF Done: E(RM06L-D3/def2-SVP/SMD) = -1675.92117988

Sum of electronic and zero-point Energies= -1675.885689

Sum of electronic and thermal Energies= -1675.867958

Sum of electronic and thermal Free Energies= -1675.934698

SCF Done: E(RM06L-D3/def2-TZVPP/SMD) = -1676.72306708

**Br_2_**

Center Atomic Atomic Coordinates (Angstroms)

Number Number Type X Y Z

---------------------------------------------------------------------

1 35 0 -0.324442 1.061469 3.115994

2 35 0 1.358413 1.914914 1.751749

---------------------------------------------------------------------

Frequencies -- 297.5765

SCF Done: E(RM06L-D3/def2-SVP/SMD) = -5147.49162484

Sum of electronic and zero-point Energies= -5147.490947

Sum of electronic and thermal Energies= -5147.488163

Sum of electronic and thermal Free Energies= -5147.515141

SCF Done: E(RM06L-D3/def2-TZVPP/SMD) = -5148.12089714

**O_2_ (triplet)**

Center Atomic Atomic Coordinates (Angstroms)

Number Number Type X Y Z

---------------------------------------------------------------------

1 8 0 0.951067 1.708332 2.081974

2 8 0 0.081804 1.267493 2.786662

---------------------------------------------------------------------

Frequencies -- 1685.4805

SCF Done: E(RM06L-D3/def2-SVP/SMD) = -150.200819370

Sum of electronic and zero-point Energies= -150.196980

Sum of electronic and thermal Energies= -150.194617

Sum of electronic and thermal Free Energies= -150.216940

SCF Done: E(RM06L-D3/def2-TZVPP/SMD) = -150.381417116

**Diethyl 2-bromomalonate (C-H tautomer)**

Center Atomic Atomic Coordinates (Angstroms)

Number Number Type X Y Z

---------------------------------------------------------------------

1 8 0 0.753303 2.056857 1.553818

2 8 0 0.519034 2.045496 -1.456906

3 6 0 1.172172 3.873520 0.001514

4 6 0 0.616723 3.227026 -1.255454

5 6 0 0.700328 3.228520 1.290681

6 8 0 0.223278 4.176017 2.101885

7 8 0 0.258963 4.184232 -2.113438

8 6 0 -0.246663 3.728642 3.389852

9 1 0 -1.060635 3.004161 3.232537

10 1 0 0.570092 3.184406 3.888636

11 6 0 -0.255524 3.735226 -3.383875

12 1 0 0.524703 3.139690 -3.882720

13 1 0 -1.102302 3.057104 -3.198434

14 6 0 -0.694326 4.927756 4.165095

15 1 0 0.130219 5.635306 4.324603

16 1 0 -1.506016 5.459759 3.651361

17 1 0 -1.066190 4.619727 5.150463

18 6 0 -0.653499 4.939798 -4.177306

19 1 0 -1.437144 5.515138 -3.666855

20 1 0 0.200486 5.607060 -4.354020

21 1 0 -1.045622 4.632045 -5.154866

22 1 0 0.945210 4.943793 0.005367

23 35 0 3.129375 3.753915 -0.068317

---------------------------------------------------------------------

Frequencies -- 17.2308 31.3264 77.7248

Frequencies -- 97.8596 110.3579 112.0746

Frequencies -- 126.2434 163.9445 187.4212

Frequencies -- 201.6891 250.9370 293.1440

Frequencies -- 297.5704 304.6885 349.7825

Frequencies -- 404.7189 421.8957 605.4045

Frequencies -- 676.9951 772.8651 784.6861

Frequencies -- 816.8317 819.3969 849.5317

Frequencies -- 883.9018 910.1757 965.5457

Frequencies -- 1023.2547 1062.5314 1071.2952

Frequencies -- 1144.2000 1148.3014 1164.0679

Frequencies -- 1165.4988 1167.6466 1175.7840

Frequencies -- 1290.8206 1299.9036 1304.3755

Frequencies -- 1352.3144 1377.2085 1387.7430

Frequencies -- 1421.6891 1425.5013 1441.8306

Frequencies -- 1442.4206 1455.2686 1456.2356

Frequencies -- 1482.8506 1483.6655 1851.7252

Frequencies -- 1884.3457 3062.3865 3065.1521

Frequencies -- 3066.3151 3075.9819 3130.2962

Frequencies -- 3141.9909 3178.5713 3179.1193

Frequencies -- 3183.9080 3185.8366 3188.5196

SCF Done: E(RM06L-D3/def2-SVP/SMD) = -3147.55047948

Sum of electronic and zero-point Energies= -3147.369428

Sum of electronic and thermal Energies= -3147.355592

Sum of electronic and thermal Free Energies= -3147.413213

SCF Done: E(RM06L-D3/def2-TZVPP/SMD) = -3148.50795392

**Diethyl 2-bromomalonate (O-H tautomer)**

Center Atomic Atomic Coordinates (Angstroms)

Number Number Type X Y Z

---------------------------------------------------------------------

1 8 0 -0.009942 2.372733 1.230517

2 8 0 -0.005874 2.387024 -1.285296

3 6 0 0.037144 4.376341 -0.005570

4 6 0 0.022277 3.694992 -1.215669

5 6 0 0.018047 3.614819 1.214290

6 8 0 0.031148 4.327802 2.336858

7 8 0 0.035682 4.346900 -2.354951

8 6 0 0.001255 3.586314 3.566848

9 1 0 -0.902416 2.957035 3.581997

10 1 0 0.861503 2.899443 3.592064

11 6 0 0.002670 3.591213 -3.584903

12 1 0 0.866176 2.909480 -3.606610

13 1 0 -0.904082 2.967535 -3.592781

14 6 0 0.026679 4.566793 4.698067

15 1 0 0.940951 5.174981 4.682589

16 1 0 -0.833927 5.247811 4.659684

17 1 0 -0.007523 4.035275 5.657623

18 6 0 0.025908 4.567934 -4.717579

19 1 0 -0.835680 5.247207 -4.680753

20 1 0 0.940724 5.174731 -4.706540

21 1 0 -0.009910 4.029098 -5.672606

22 35 0 0.061366 6.264078 0.010668

23 1 0 -0.014247 2.068094 -0.324608

---------------------------------------------------------------------

Frequencies -- 56.1317 82.3278 88.2750

Frequencies -- 94.1172 110.1760 116.9503

Frequencies -- 138.2140 184.4440 231.7194

Frequencies -- 240.7474 256.4796 261.2898

Frequencies -- 285.0010 319.4148 378.8592

Frequencies -- 426.7287 456.2916 479.7044

Frequencies -- 707.1810 709.6764 759.4641

Frequencies -- 820.3182 827.3157 829.7382

Frequencies -- 881.1066 920.9009 959.2544

Frequencies -- 1047.2149 1071.5064 1086.9182

Frequencies -- 1141.5073 1146.1968 1162.2817

Frequencies -- 1163.9317 1166.0249 1308.9289

Frequencies -- 1316.9651 1350.8494 1363.7437

Frequencies -- 1383.7739 1402.5741 1422.9456

Frequencies -- 1436.1022 1436.4378 1447.7781

Frequencies -- 1451.9806 1462.8784 1469.8490

Frequencies -- 1491.9092 1502.6366 1686.9551

Frequencies -- 1709.7818 2956.7587 3059.3620

Frequencies -- 3061.8012 3064.7028 3065.5362

Frequencies -- 3123.4947 3133.8248 3175.4897

Frequencies -- 3180.8542 3185.6419 3190.3154

SCF Done: E(RM06L-D3/def2-SVP/SMD) = -3147.54194933

Sum of electronic and zero-point Energies= -3147.360946

Sum of electronic and thermal Energies= -3147.347472

Sum of electronic and thermal Free Energies= -3147.402348

SCF Done: E(RM06L-D3/def2-TZVPP/SMD) = -3148.49848101

**Dimethyl sulfoxide**

Center Atomic Atomic Coordinates (Angstroms)

Number Number Type X Y Z

---------------------------------------------------------------------

1 8 0 -0.427908 -6.104051 2.879880

2 16 0 0.414804 -6.380323 1.660189

3 6 0 -0.574710 -5.890687 0.219451

4 6 0 0.316669 -8.166124 1.360790

5 1 0 -0.674664 -4.798756 0.249588

6 1 0 -1.569323 -6.351330 0.291000

7 1 0 -0.073689 -6.184225 -0.712463

8 1 0 0.831947 -8.662275 2.192338

9 1 0 0.811344 -8.432871 0.417457

10 1 0 -0.737668 -8.473587 1.348489

---------------------------------------------------------------------

Frequencies -- 202.7105 262.2761 291.2508

Frequencies -- 313.3213 368.8401 636.9850

Frequencies -- 662.3441 875.0988 920.4896

Frequencies -- 957.0597 1006.4774 1126.5084

Frequencies -- 1274.9923 1296.4077 1393.1555

Frequencies -- 1400.3541 1407.4036 1421.2619

Frequencies -- 3052.4531 3055.8108 3200.4537

Frequencies -- 3204.1247 3206.4262 3210.8635

SCF Done: E(RM06L-D3/def2-SVP/SMD) = -552.925119744

Sum of electronic and zero-point Energies= -552.845960

Sum of electronic and thermal Energies= -552.840392

Sum of electronic and thermal Free Energies= -552.874112

SCF Done: E(RM06L-D3/def2-TZVPP/SMD) = -553.265462160

**H_2_O (in DMSO)**

Center Atomic Atomic Coordinates (Angstroms)

Number Number Type X Y Z

---------------------------------------------------------------------

1 8 0 -0.612789 -2.951487 0.000000

2 1 0 0.348489 -2.890027 0.000000

3 1 0 -0.875816 -2.024736 0.000000

---------------------------------------------------------------------

Frequencies -- 1682.2805 3846.3229 3949.1164

SCF Done: E(RM06L-D3/def2-SVP/SMD) = -76.3544107699

Sum of electronic and zero-point Energies= -76.332819

Sum of electronic and thermal Energies= -76.329984

Sum of electronic and thermal Free Energies= -76.351130

SCF Done: E(RM06L-D3/def2-TZVPP/SMD) = -76.4524880699

**P1 (Bi^…^O-coordination, 1^st^ isomer)**

Center Atomic Atomic Coordinates (Angstroms)

Number Number Type X Y Z

---------------------------------------------------------------------

1 83 0 -0.331958 0.008811 1.605385

2 8 0 1.171541 1.824243 2.202283

3 8 0 1.670838 -0.513468 0.620339

4 8 0 0.184302 -2.132420 2.626238

5 8 0 -0.896246 -1.551642 0.029697

6 6 0 0.309324 -3.518406 0.680089

7 6 0 2.867211 1.562508 0.538572

8 1 0 3.682980 2.075927 0.033646

9 1 0 0.703295 -4.460808 0.304988

10 6 0 2.545046 0.266532 0.131050

11 6 0 2.179155 2.230629 1.568275

12 6 0 -0.344210 -2.668918 -0.215087

13 6 0 0.508922 -3.195127 2.035316

14 8 0 -0.400911 -3.116190 -1.479725

15 8 0 1.121856 -4.159639 2.740538

16 8 0 2.691323 3.433523 1.874400

17 8 0 3.278590 -0.200854 -0.891480

18 6 0 -1.012147 -2.268119 -2.460632

19 1 0 -2.098202 -2.225302 -2.276805

20 1 0 -0.638916 -1.238576 -2.344324

21 6 0 1.404970 -3.884236 4.116302

22 1 0 2.028114 -2.978080 4.185405

23 1 0 0.465462 -3.656486 4.644668

24 6 0 2.031355 4.189923 2.894609

25 1 0 1.002792 4.418519 2.571110

26 1 0 1.942053 3.576425 3.804692

27 6 0 2.970827 -1.508612 -1.391517

28 1 0 3.256994 -2.260717 -0.638307

29 1 0 1.881554 -1.604840 -1.527085

30 6 0 -0.693061 -2.819504 -3.816597

31 1 0 0.389891 -2.823086 -4.004298

32 1 0 -1.059495 -3.848301 -3.934441

33 1 0 -1.165377 -2.203760 -4.593141

34 6 0 2.095451 -5.080819 4.696305

35 1 0 1.464520 -5.978021 4.636022

36 1 0 3.037527 -5.294757 4.173517

37 1 0 2.330086 -4.905964 5.754245

38 6 0 2.831426 5.433784 3.135814

39 1 0 3.848069 5.195186 3.476628

40 1 0 2.914612 6.042085 2.224998

41 1 0 2.352850 6.049160 3.908457

42 6 0 3.712039 -1.693385 -2.680797

43 1 0 3.384529 -0.968355 -3.439477

44 1 0 4.795204 -1.573049 -2.544258

45 1 0 3.534323 -2.700432 -3.079637

46 8 0 -2.682857 0.516808 1.205723

47 6 0 -3.234589 0.737700 0.097489

48 6 0 -2.613015 1.076662 -1.119564

49 8 0 -4.572169 0.648148 0.013628

50 1 0 -3.228882 1.168823 -2.011852

51 6 0 -1.235145 1.264014 -1.242899

52 6 0 -5.281234 0.258725 1.193506

53 8 0 -0.810024 1.524614 -2.489277

54 1 0 -4.912350 -0.722084 1.534638

55 1 0 -5.064382 0.972274 2.004343

56 6 0 -6.742070 0.219802 0.860492

57 6 0 0.601094 1.664763 -2.696662

58 1 0 -7.106694 1.204370 0.537300

59 1 0 -6.950842 -0.498236 0.055852

60 1 0 -7.324959 -0.081723 1.740260

61 1 0 0.953007 2.580904 -2.195160

62 1 0 1.131875 0.826050 -2.218091

63 6 0 0.849436 1.700689 -4.173991

64 1 0 0.523944 0.768129 -4.656597

65 1 0 0.318396 2.533123 -4.655170

66 1 0 1.921687 1.825626 -4.373170

67 8 0 -0.356806 1.234941 -0.325700

---------------------------------------------------------------------

Frequencies -- 17.5364 23.1261 28.9815

Frequencies -- 39.9193 45.1456 57.3304

Frequencies -- 60.9390 63.3449 71.2202

Frequencies -- 73.5508 75.1112 80.3469

Frequencies -- 85.3961 85.8550 88.3965

Frequencies -- 98.2897 102.1427 107.0728

Frequencies -- 113.4234 122.3365 124.1331

Frequencies -- 137.1350 139.8358 142.5710

Frequencies -- 144.2178 148.7334 165.3033

Frequencies -- 167.8972 170.2498 177.3277

Frequencies -- 183.6147 187.2206 201.4481

Frequencies -- 204.5020 207.9853 210.9058

Frequencies -- 235.1310 238.3705 246.6760

Frequencies -- 273.4998 275.8425 277.5111

Frequencies -- 282.1472 282.6628 283.8511

Frequencies -- 287.9271 302.2350 315.8498

Frequencies -- 347.1967 350.6270 356.1724

Frequencies -- 394.5657 397.6149 401.2078

Frequencies -- 447.1432 449.2664 451.0444

Frequencies -- 457.1437 460.7626 469.8174

Frequencies -- 717.7052 720.0221 722.0025

Frequencies -- 722.1943 731.8596 732.5325

Frequencies -- 738.6425 739.0605 740.5770

Frequencies -- 780.9178 782.0572 782.9374

Frequencies -- 796.8479 797.4401 798.0821

Frequencies -- 815.2922 820.2905 821.5713

Frequencies -- 823.6636 835.4396 836.1033

Frequencies -- 885.0874 887.0233 889.4581

Frequencies -- 925.8888 928.5840 929.3896

Frequencies -- 1032.6125 1033.8382 1036.1756

Frequencies -- 1050.0946 1051.0713 1052.8186

Frequencies -- 1079.7315 1080.1227 1081.7761

Frequencies -- 1115.6188 1116.8625 1120.6429

Frequencies -- 1142.1300 1143.0452 1143.4561

Frequencies -- 1143.8344 1145.0751 1145.4914

Frequencies -- 1159.0327 1162.0022 1165.5505

Frequencies -- 1166.2640 1167.4171 1168.6044

Frequencies -- 1174.2819 1176.6946 1179.0980

Frequencies -- 1289.0482 1292.1106 1292.6376

Frequencies -- 1301.2281 1305.2785 1307.0633

Frequencies -- 1331.2621 1335.9451 1349.0016

Frequencies -- 1358.4100 1366.4570 1367.1846

Frequencies -- 1385.8292 1387.0488 1392.1740

Frequencies -- 1408.0434 1409.7186 1412.3625

Frequencies -- 1429.9341 1431.9910 1434.4450

Frequencies -- 1437.7175 1438.6079 1440.7134

Frequencies -- 1441.1610 1445.1205 1448.8804

Frequencies -- 1450.7685 1454.4904 1455.2642

Frequencies -- 1455.8353 1458.1370 1460.2082

Frequencies -- 1474.1893 1474.5533 1477.0163

Frequencies -- 1485.8820 1486.4880 1489.2722

Frequencies -- 1519.1246 1520.2772 1522.9837

Frequencies -- 1600.9818 1602.6012 1609.6515

Frequencies -- 1668.4561 1670.1763 1712.3653

Frequencies -- 3054.5652 3057.1933 3058.6288

Frequencies -- 3058.7345 3059.5381 3060.1824

Frequencies -- 3060.6947 3062.1227 3063.0316

Frequencies -- 3063.6914 3064.2349 3065.7088

Frequencies -- 3118.9314 3121.6728 3122.7127

Frequencies -- 3125.0035 3126.7823 3129.1418

Frequencies -- 3166.3645 3173.6192 3173.9797

Frequencies -- 3176.1906 3176.6777 3177.7905

Frequencies -- 3180.4062 3180.9599 3183.8447

Frequencies -- 3184.3704 3185.1818 3186.4580

Frequencies -- 3243.5623 3244.0625 3245.5027

SCF Done: E(RM06L-D3/def2-SVP/SMD) = -1936.26871827

Sum of electronic and zero-point Energies= -1935.728769

Sum of electronic and thermal Energies= -1935.688226

Sum of electronic and thermal Free Energies= -1935.804583

SCF Done: E(RM06L-D3/def2-TZVPP/SMD) = -1938.20156637

**P1 (Bi^…^O-coordination, 2^nd^ isomer)**

Center Atomic Atomic Coordinates (Angstroms)

Number Number Type X Y Z

---------------------------------------------------------------------

1 83 0 0.661137 15.158717 6.284932

2 8 0 1.049952 13.292677 7.766439

3 8 0 1.211284 13.262640 4.961799

4 6 0 1.936692 12.406711 7.656494

5 6 0 2.534193 11.956103 6.468632

6 6 0 2.131861 12.437378 5.215779

7 8 0 0.696317 15.835906 4.021539

8 8 0 1.170421 17.502412 6.209000

9 6 0 1.454367 16.662552 3.443848

10 6 0 2.101421 17.761287 4.025419

11 6 0 1.888333 18.106388 5.369659

12 8 0 1.319630 15.944081 8.429468

13 8 0 2.870549 15.149212 6.055003

14 6 0 2.484341 16.036846 8.883494

15 6 0 3.703332 15.763513 8.226439

16 6 0 3.807636 15.358664 6.897003

17 1 0 4.626126 15.890656 8.788515

18 1 0 2.744098 18.387934 3.410605

19 1 0 3.346377 11.233849 6.522466

20 8 0 2.643977 16.443806 10.152291

21 8 0 5.049188 15.171659 6.445761

22 8 0 2.528542 19.220548 5.767289

23 8 0 1.680076 16.510278 2.126370

24 8 0 2.839124 11.934610 4.184016

25 8 0 2.398471 11.816989 8.773768

26 6 0 0.994402 15.452981 1.447123

27 1 0 0.892496 14.589937 2.119227

28 1 0 -0.031111 15.788211 1.216006

29 6 0 1.758170 15.120180 0.200732

30 1 0 2.791702 14.827147 0.432420

31 1 0 1.278476 14.286154 -0.328965

32 1 0 1.803308 15.973827 -0.489392

33 6 0 2.377912 19.607523 7.136790

34 1 0 1.317001 19.825260 7.341920

35 1 0 2.651436 18.762651 7.790619

36 6 0 3.250275 20.803093 7.374967

37 1 0 3.163792 21.137630 8.416905

38 1 0 4.307042 20.572097 7.183363

39 1 0 2.966756 21.644011 6.727458

40 6 0 2.638988 12.532857 2.901990

41 1 0 1.616650 12.317267 2.549176

42 1 0 2.713860 13.629455 3.004825

43 6 0 3.674254 11.978241 1.969394

44 1 0 3.529471 12.371649 0.954932

45 1 0 4.691512 12.242611 2.291042

46 1 0 3.613235 10.882890 1.914011

47 6 0 1.904718 12.310930 10.021535

48 1 0 2.080141 13.397658 10.078653

49 1 0 0.812958 12.169612 10.067142

50 6 0 2.608418 11.578182 11.123184

51 1 0 3.694421 11.738630 11.080463

52 1 0 2.254696 11.932558 12.100277

53 1 0 2.423448 10.496536 11.070536

54 6 0 1.458678 16.757104 10.892767

55 1 0 0.784355 15.886039 10.887309

56 1 0 0.918942 17.571449 10.382584

57 6 0 1.867510 17.141986 12.281776

58 1 0 0.981994 17.389137 12.881247

59 1 0 2.396991 16.322933 12.787215

60 1 0 2.526715 18.020461 12.279430

61 6 0 5.207801 14.709824 5.093022

62 1 0 4.690831 13.742553 4.980627

63 1 0 4.716350 15.418259 4.406474

64 6 0 6.675913 14.593609 4.821605

65 1 0 7.183241 15.561713 4.931208

66 1 0 7.154393 13.878774 5.504458

67 1 0 6.842039 14.241344 3.795386

---------------------------------------------------------------------

Frequencies -- 11.8029 21.2716 22.6244

Frequencies -- 37.2216 39.4227 52.3326

Frequencies -- 56.6698 62.5278 64.8392

Frequencies -- 69.6932 75.3107 79.7112

Frequencies -- 85.0367 88.3191 98.0185

Frequencies -- 106.6345 106.9351 115.0162

Frequencies -- 118.7628 124.3142 129.0864

Frequencies -- 135.0595 141.2912 146.6946

Frequencies -- 158.3186 164.2890 169.1424

Frequencies -- 183.5045 191.7375 196.8306

Frequencies -- 199.7236 202.2153 207.7126

Frequencies -- 221.7401 225.6999 237.0392

Frequencies -- 242.0723 253.3144 260.7896

Frequencies -- 277.1542 278.7566 281.4151

Frequencies -- 285.6581 286.1038 288.9067

Frequencies -- 295.7341 304.1922 314.9987

Frequencies -- 354.0103 355.6575 367.5994

Frequencies -- 393.3581 398.5472 410.3760

Frequencies -- 452.8785 456.1068 456.5064

Frequencies -- 458.2059 465.1621 482.7574

Frequencies -- 715.2194 716.5363 722.0406

Frequencies -- 726.9654 729.6000 730.2259

Frequencies -- 733.9060 742.9804 745.9212

Frequencies -- 768.1638 783.4874 786.8682

Frequencies -- 796.9476 798.7351 803.2191

Frequencies -- 804.0832 818.7072 823.0665

Frequencies -- 826.3202 835.6519 838.3989

Frequencies -- 890.5639 892.5567 894.0955

Frequencies -- 929.4846 930.4925 933.6953

Frequencies -- 1031.1340 1037.3985 1040.8969

Frequencies -- 1050.6503 1051.5395 1054.7087

Frequencies -- 1076.3411 1082.6925 1086.6277

Frequencies -- 1113.7565 1117.5814 1122.2402

Frequencies -- 1138.9226 1140.8828 1144.6271

Frequencies -- 1145.4924 1146.8468 1148.1821

Frequencies -- 1159.9559 1163.7067 1165.5477

Frequencies -- 1167.7458 1170.2912 1172.9527

Frequencies -- 1175.3794 1183.1215 1184.3795

Frequencies -- 1278.5498 1287.0866 1298.1769

Frequencies -- 1300.3706 1304.4041 1307.2603

Frequencies -- 1331.0260 1337.1371 1351.2532

Frequencies -- 1361.5565 1366.3873 1371.5712

Frequencies -- 1385.7870 1392.4688 1395.7531

Frequencies -- 1410.3948 1411.0271 1415.5291

Frequencies -- 1429.7489 1434.0107 1438.3437

Frequencies -- 1439.0568 1439.2734 1440.2063

Frequencies -- 1440.9482 1441.9950 1443.1244

Frequencies -- 1453.5748 1454.5744 1454.9804

Frequencies -- 1456.1475 1460.9027 1472.3845

Frequencies -- 1474.7253 1481.5114 1486.3504

Frequencies -- 1488.1598 1494.4606 1511.4089

Frequencies -- 1517.0293 1530.2944 1535.2165

Frequencies -- 1598.1782 1601.8929 1613.8446

Frequencies -- 1672.2839 1677.5465 1720.6386

Frequencies -- 3043.7288 3047.5648 3050.6697

Frequencies -- 3051.9692 3056.6793 3057.4297

Frequencies -- 3058.5737 3058.7805 3059.9009

Frequencies -- 3060.6290 3062.8788 3065.0396

Frequencies -- 3106.1808 3108.8432 3109.4164

Frequencies -- 3114.4497 3130.3821 3150.1349

Frequencies -- 3170.3450 3170.6959 3171.7683

Frequencies -- 3175.3075 3175.9450 3176.9995

Frequencies -- 3178.7715 3179.8769 3179.8993

Frequencies -- 3180.7266 3181.0940 3188.6776

Frequencies -- 3242.7761 3244.3841 3248.1621

SCF Done: E(RM06L-D3/def2-SVP/SMD) = -1936.26811877

Sum of electronic and zero-point Energies= -1935.727224

Sum of electronic and thermal Energies= -1935.687145

Sum of electronic and thermal Free Energies= -1935.802957

SCF Done: E(RM06L-D3/def2-TZVPP/SMD) = -1938.20172596

**P1 (Bi^…^C-coordination, 3^nd^ isomer)**

Center Atomic Atomic Coordinates (Angstroms)

Number Number Type X Y Z

---------------------------------------------------------------------

1 83 0 1.601395 -0.293491 3.832769

2 6 0 1.488456 2.005586 4.443963

3 6 0 1.505454 2.099282 5.927377

4 6 0 2.485256 2.773702 3.677509

5 8 0 2.135954 2.858427 6.620538

6 8 0 0.631786 1.189612 6.427824

7 8 0 3.610754 3.057087 4.022102

8 8 0 2.006891 3.045418 2.438526

9 6 0 0.347953 1.260630 7.833679

10 6 0 2.954620 3.606849 1.515995

11 1 0 1.292077 1.257215 8.397985

12 1 0 -0.138627 2.227164 8.042669

13 6 0 -0.531747 0.102578 8.191238

14 1 0 3.357219 4.540199 1.939141

15 1 0 3.807253 2.913775 1.419624

16 6 0 2.268190 3.832112 0.204405

17 1 0 -1.469140 0.117268 7.619137

18 1 0 -0.033359 -0.857964 7.999312

19 1 0 -0.788637 0.136583 9.257690

20 1 0 1.875335 2.895491 -0.212546

21 1 0 1.430802 4.535777 0.307070

22 1 0 2.976398 4.256909 -0.519229

23 6 0 3.897210 -0.151972 3.205748

24 6 0 4.691941 -0.548183 4.405183

25 6 0 4.220580 -0.854325 1.941593

26 8 0 5.781984 -1.058271 4.430754

27 8 0 4.009060 -0.211252 5.530154

28 8 0 4.636600 -1.981942 1.819816

29 8 0 3.940305 -0.052925 0.886338

30 6 0 4.694763 -0.433486 6.777027

31 6 0 4.075831 -0.636064 -0.416521

32 1 0 5.006667 -1.487933 6.820064

33 1 0 5.615662 0.169709 6.777776

34 6 0 3.773731 -0.063364 7.896092

35 1 0 5.144023 -0.820116 -0.614024

36 1 0 3.583510 -1.621034 -0.421941

37 6 0 3.474804 0.305279 -1.416708

38 1 0 3.480186 0.993537 7.836303

39 1 0 2.867257 -0.685270 7.889816

40 1 0 4.274172 -0.219815 8.860489

41 1 0 2.397961 0.452877 -1.244683

42 1 0 3.963376 1.289343 -1.389181

43 1 0 3.592198 -0.097110 -2.431260

44 6 0 0.877477 0.164371 1.638884

45 6 0 0.566463 -1.172441 1.059518

46 6 0 -0.296464 1.084071 1.650885

47 8 0 -0.376778 -1.468711 0.368616

48 8 0 1.502313 -2.062367 1.477035

49 8 0 -1.078823 1.158928 2.578049

50 8 0 -0.391922 1.811733 0.539472

51 6 0 1.389116 -3.429596 1.034420

52 6 0 -1.458787 2.777150 0.495772

53 1 0 0.322214 -3.693139 0.992961

54 1 0 1.777196 -3.491349 0.005042

55 6 0 2.161867 -4.291102 1.984883

56 1 0 -2.393052 2.285696 0.805814

57 1 0 -1.247090 3.556580 1.245956

58 6 0 -1.543172 3.330143 -0.892810

59 1 0 3.208698 -3.966907 2.050282

60 1 0 1.727775 -4.260185 2.994423

61 1 0 2.142430 -5.335113 1.646562

62 1 0 -0.607729 3.818533 -1.196632

63 1 0 -1.767986 2.542357 -1.624238

64 1 0 -2.344602 4.077903 -0.948768

65 1 0 0.463991 2.168650 4.078119

66 1 0 3.999138 0.936985 3.081090

67 1 0 1.742865 0.596203 1.113834

---------------------------------------------------------------------

Frequencies -- 22.0894 26.9893 37.2899

Frequencies -- 39.2057 44.8618 48.0574

Frequencies -- 54.9940 58.2900 64.7311

Frequencies -- 71.9983 78.1340 80.8074

Frequencies -- 86.1985 87.7682 92.0548

Frequencies -- 92.3937 99.1754 106.8228

Frequencies -- 108.7377 121.0476 124.2861

Frequencies -- 127.5480 128.3408 138.3704

Frequencies -- 142.9570 153.6647 159.4906

Frequencies -- 165.0076 168.8990 175.7439

Frequencies -- 184.5550 187.1844 195.2475

Frequencies -- 203.4140 209.4727 224.7026

Frequencies -- 229.0287 242.7062 248.0626

Frequencies -- 287.1995 287.9553 291.0094

Frequencies -- 300.4621 307.2466 320.1461

Frequencies -- 325.5706 331.4270 337.2567

Frequencies -- 340.3208 344.3392 348.5024

Frequencies -- 393.2957 394.5357 397.7729

Frequencies -- 421.3076 424.4831 431.2130

Frequencies -- 469.5237 484.6542 574.3247

Frequencies -- 640.8916 676.5659 695.3191

Frequencies -- 725.3512 730.2891 740.8985

Frequencies -- 750.2267 761.2183 784.7003

Frequencies -- 809.0996 810.4359 812.3388

Frequencies -- 814.6312 819.9355 820.7706

Frequencies -- 823.2882 835.5347 836.0767

Frequencies -- 878.0062 880.7566 881.6457

Frequencies -- 899.7304 913.3150 916.9011

Frequencies -- 962.1016 973.6935 989.1900

Frequencies -- 991.0778 1007.6327 1023.2313

Frequencies -- 1029.8233 1037.1535 1040.9890

Frequencies -- 1066.0606 1071.5158 1075.4407

Frequencies -- 1079.3635 1092.6870 1099.4279

Frequencies -- 1139.5081 1140.7686 1141.9383

Frequencies -- 1143.0730 1145.3310 1146.2335

Frequencies -- 1148.4422 1149.6210 1151.9008

Frequencies -- 1162.4787 1164.1692 1165.5796

Frequencies -- 1170.2932 1173.4288 1176.6934

Frequencies -- 1227.9638 1237.2416 1257.2443

Frequencies -- 1283.9268 1290.3947 1291.7112

Frequencies -- 1293.8057 1300.5483 1307.0212

Frequencies -- 1342.2202 1350.3354 1360.4943

Frequencies -- 1368.1802 1369.0568 1374.7215

Frequencies -- 1384.3359 1388.4786 1402.3400

Frequencies -- 1413.5200 1415.9461 1419.1435

Frequencies -- 1422.9790 1424.7039 1438.3143

Frequencies -- 1440.7892 1441.5812 1441.9376

Frequencies -- 1442.1620 1443.3080 1444.2998

Frequencies -- 1450.3325 1451.1121 1456.9929

Frequencies -- 1458.4515 1463.0023 1469.8746

Frequencies -- 1478.5597 1480.0288 1480.9893

Frequencies -- 1483.9322 1490.1071 1496.8266

Frequencies -- 1800.9721 1814.4428 1822.8112

Frequencies -- 1842.3708 1857.4792 1866.7537

Frequencies -- 3053.8046 3054.8539 3058.2318

Frequencies -- 3060.3507 3061.0934 3063.5906

Frequencies -- 3064.3778 3067.2549 3068.5430

Frequencies -- 3068.8637 3069.5926 3070.2624

Frequencies -- 3113.5276 3123.8241 3131.4837

Frequencies -- 3131.9618 3138.1934 3139.5806

Frequencies -- 3139.6594 3140.4730 3141.5712

Frequencies -- 3165.9172 3169.3338 3171.3575

Frequencies -- 3174.7114 3176.0839 3180.4901

Frequencies -- 3182.2543 3183.5476 3184.2397

Frequencies -- 3188.6880 3189.2134 3191.8042

SCF Done: E(RM06L-D3/def2-SVP/SMD) = -1936.22550406

Sum of electronic and zero-point Energies= -1935.684298

Sum of electronic and thermal Energies= -1935.643986

Sum of electronic and thermal Free Energies= -1935.759621

SCF Done: E(RM06L-D3/def2-TZVPP/SMD) = -1938.15860520

**P2**

Center Atomic Atomic Coordinates (Angstroms)

Number Number Type X Y Z

---------------------------------------------------------------------

1 83 0 -1.797185 1.875505 -1.878115

2 35 0 -3.634485 3.299330 -0.586029

3 35 0 0.353033 2.753235 -0.586120

4 35 0 -2.112063 -0.430478 -0.593084

---------------------------------------------------------------------

Frequencies -- 59.3298 63.3642 75.6169

Frequencies -- 199.0212 200.1401 210.9638

SCF Done: E(RM06L-D3/def2-SVP/SMD) = -7936.15798263

Sum of electronic and zero-point Energies= -7936.156141

Sum of electronic and thermal Energies= -7936.149237

Sum of electronic and thermal Free Energies= -7936.193602

SCF Done: E(RM06L-D3/def2- TZVPP/SMD) = -7937.10822877

**P3**

Center Atomic Atomic Coordinates (Angstroms)

Number Number Type X Y Z

---------------------------------------------------------------------

1 83 0 -1.636625 1.353027 6.564569

2 35 0 -3.979004 2.935439 6.958198

3 8 0 -2.617295 0.378311 4.911599

4 83 0 -1.511193 -1.305857 3.941724

5 35 0 0.837309 -2.886112 3.570584

6 8 0 -0.530695 -0.329559 5.595308

7 35 0 -4.272380 -2.440718 2.932875

8 35 0 -2.819849 -3.276169 5.439421

9 35 0 -0.330440 3.327393 5.069124

10 35 0 1.119679 2.492920 7.577644

---------------------------------------------------------------------

Frequencies -- 12.4594 20.7994 24.3377

Frequencies -- 28.0510 32.0708 35.5239

Frequencies -- 39.6388 48.4614 51.8781

Frequencies -- 86.4119 89.3551 96.9836

Frequencies -- 104.2129 107.5988 120.3566

Frequencies -- 122.3897 139.2349 159.0328

Frequencies -- 172.6467 172.7831 265.8523

Frequencies -- 442.7185 445.4978 521.7695

SCF Done: E(RM06L-D3/def2-SVP/SMD) = -16022.4002807

Sum of electronic and zero-point Energies= -16022.392672

Sum of electronic and thermal Energies= -16022.373187

Sum of electronic and thermal Free Energies= -16022.455900

SCF Done: E(RM06L-D3/def2-TZVPP/SMD) = -16024.4787872

**P4**

Center Atomic Atomic Coordinates (Angstroms)

Number Number Type X Y Z

---------------------------------------------------------------------

1 83 0 6.396694 6.924075 5.733324

2 8 0 5.063612 6.845329 7.805486

3 8 0 4.545164 8.078788 5.141851

4 8 0 5.004813 5.014258 5.514367

5 8 0 6.562689 6.269049 3.458273

6 8 0 7.248836 8.806802 4.546865

7 8 0 6.748962 8.862315 7.356121

8 6 0 3.504349 6.936754 11.592114

9 1 0 3.963911 6.315731 12.371584

10 6 0 4.296364 6.846927 10.323257

11 1 0 4.382107 5.814812 9.952215

12 6 0 4.114831 7.628633 8.081439

13 6 0 3.438247 8.521281 7.230071

14 1 0 2.650898 9.132237 7.667223

15 6 0 3.692313 8.683745 5.865853

16 6 0 3.199634 9.883091 3.880183

17 1 0 3.147990 8.956313 3.288485

18 6 0 2.204236 10.900513 3.413727

19 1 0 2.393443 11.153759 2.362584

20 6 0 2.837999 1.669749 6.289727

21 1 0 2.462847 1.522394 7.310691

22 6 0 3.600682 2.956474 6.200855

23 1 0 4.465413 2.972079 6.884240

24 6 0 4.799309 4.194446 4.580197

25 6 0 5.265833 4.265244 3.256698

26 1 0 4.999918 3.466198 2.567564

27 6 0 6.116848 5.282386 2.804392

28 6 0 7.771893 5.747367 -0.389595

29 1 0 8.479398 6.471964 -0.813040

30 6 0 7.420693 6.130436 1.015658

31 1 0 6.962356 7.131095 1.064967

32 6 0 7.319425 10.161176 2.359175

33 1 0 6.512038 9.415989 2.273357

34 6 0 7.253280 11.193905 1.275496

35 1 0 7.344164 10.716726 0.290974

36 6 0 7.140568 10.046118 4.711092

37 6 0 6.949165 10.748924 5.915117

38 1 0 6.885065 11.834584 5.880430

39 6 0 6.791753 10.108725 7.149970

40 6 0 6.186408 10.390103 9.432798

41 1 0 6.876554 9.600059 9.770885

42 6 0 6.136250 11.489826 10.449945

43 1 0 5.844241 11.083313 11.428234

44 83 0 7.830664 7.048183 9.042572

45 8 0 9.156271 7.160133 6.967525

46 8 0 9.682648 5.887031 9.621505

47 8 0 9.266110 8.923701 9.249948

48 8 0 7.693497 7.706882 11.323627

49 8 0 7.015693 5.123630 10.207113

50 8 0 7.485963 5.123848 7.411587

51 6 0 10.763959 7.188677 3.200529

52 1 0 10.293026 7.809499 2.427343

53 6 0 9.971492 7.251552 4.470633

54 1 0 8.945654 6.872524 4.310495

55 6 0 10.140209 6.420721 6.694233

56 6 0 10.830603 5.531240 7.537266

57 1 0 11.649447 4.962492 7.101100

58 6 0 10.565753 5.330246 8.894813

59 6 0 11.095645 4.125893 10.867153

60 1 0 10.075468 3.716201 10.939621

61 6 0 12.126649 3.140709 11.325113

62 1 0 11.948480 2.874499 12.375066

63 6 0 11.587774 12.171020 8.495766

64 1 0 11.994009 12.295974 7.483782

65 6 0 10.793458 10.903182 8.581725

66 1 0 11.403169 10.017739 8.339819

67 6 0 9.512941 9.726908 10.188683

68 6 0 9.053396 9.668697 11.514813

69 1 0 9.358287 10.452720 12.204985

70 6 0 8.174748 8.679058 11.974233

71 6 0 6.545086 8.265378 15.188911

72 1 0 5.825871 7.559162 15.623425

73 6 0 6.875475 7.864634 13.783549

74 1 0 5.979975 7.844864 13.142098

75 6 0 7.326113 3.826365 12.422800

76 1 0 6.301956 4.219664 12.531594

77 6 0 7.667770 2.852538 13.509384

78 1 0 7.567374 3.332203 14.491822

79 6 0 7.343070 3.919826 10.061988

80 6 0 7.624431 3.241365 8.862190

81 1 0 7.868651 2.181680 8.902450

82 6 0 7.633897 3.885635 7.618632

83 6 0 8.177612 3.666258 5.311713

84 1 0 9.115299 4.240565 5.414846

85 6 0 8.303349 2.572992 4.294149

86 1 0 8.514550 3.001903 3.304709

87 8 0 7.208870 10.832108 3.619887

88 8 0 6.639240 10.942747 8.196563

89 8 0 2.919286 9.587573 5.255981

90 8 0 3.650423 7.663142 9.342650

91 8 0 6.501514 5.157076 1.523980

92 8 0 4.062426 3.106346 4.852129

93 8 0 7.852881 3.072092 6.568992

94 8 0 7.446372 3.152606 11.164861

95 8 0 10.631916 6.436181 5.442764

96 8 0 11.368490 4.447720 9.495275

97 8 0 10.296061 10.783164 9.920226

98 8 0 7.809447 8.814844 13.259204

99 1 0 9.870155 8.278095 4.855410

100 1 0 11.112298 5.047485 11.469408

101 1 0 12.093933 2.216246 10.733054

102 1 0 13.140893 3.555697 11.250947

103 1 0 10.830345 6.160647 2.817987

104 1 0 11.787166 7.559807 3.349017

105 1 0 7.387224 4.372120 5.010885

106 1 0 9.116973 1.879377 4.545993

107 1 0 7.370285 1.997502 4.217836

108 1 0 7.995016 4.702581 12.427257

109 1 0 7.001979 1.978818 13.493743

110 1 0 8.701419 2.490188 13.417439

111 1 0 8.309778 6.163225 1.665943

112 1 0 6.883632 5.728124 -1.035569

113 1 0 8.241617 4.755313 -0.431311

114 1 0 2.977905 3.824183 6.470457

115 1 0 3.471066 0.808498 6.036758

116 1 0 1.974134 1.665175 5.611134

117 1 0 9.947789 10.897773 7.875579

118 1 0 12.432067 12.166247 9.198569

119 1 0 10.966870 13.048992 8.719710

120 1 0 7.312720 6.854139 13.738944

121 1 0 6.097615 9.267790 15.226718

122 1 0 7.439056 8.270011 15.827218

123 1 0 8.264868 9.593884 2.329745

124 1 0 6.299397 11.739072 1.298117

125 1 0 8.064010 11.929377 1.367456

126 1 0 5.198328 9.919598 9.281812

127 1 0 7.121692 11.962544 10.564192

128 1 0 5.411488 12.267250 10.172417

129 1 0 5.327175 7.214155 10.482306

130 1 0 2.475436 6.581066 11.445438

131 1 0 3.454473 7.969573 11.964161

132 1 0 4.234378 10.254596 3.798121

133 1 0 1.176408 10.520617 3.488514

134 1 0 2.268025 11.826783 4.000288

---------------------------------------------------------------------

Frequencies -- 14.7377 23.0600 26.2217

Frequencies -- 27.0867 30.7548 31.3330

Frequencies -- 33.0613 34.5289 37.5152

Frequencies -- 40.7868 45.6103 48.0347

Frequencies -- 49.6151 55.1695 59.1023

Frequencies -- 60.4274 61.5888 62.8585

Frequencies -- 64.0682 67.2437 70.4907

Frequencies -- 73.9795 76.4297 79.4274

Frequencies -- 82.6957 85.7700 88.1178

Frequencies -- 89.5638 93.1398 94.2385

Frequencies -- 95.0694 96.0535 98.3016

Frequencies -- 99.0874 101.6682 102.9886

Frequencies -- 106.7883 108.9152 111.3589

Frequencies -- 114.0496 115.4126 116.7025

Frequencies -- 121.5249 124.5847 128.0058

Frequencies -- 130.3733 130.5818 133.8201

Frequencies -- 141.8202 144.9231 147.2699

Frequencies -- 149.3693 151.9595 158.1376

Frequencies -- 160.2081 164.4044 167.6843

Frequencies -- 172.1772 173.6428 174.0029

Frequencies -- 177.9965 180.8660 182.8049

Frequencies -- 185.6860 187.2245 189.7179

Frequencies -- 195.7355 202.3685 203.8731

Frequencies -- 206.7247 207.3786 214.3010

Frequencies -- 215.9737 217.9522 222.9016

Frequencies -- 226.3093 229.6113 231.5848

Frequencies -- 232.8661 233.1015 238.8532

Frequencies -- 241.7750 254.9446 259.9477

Frequencies -- 271.2589 271.6069 273.8245

Frequencies -- 277.2303 279.8312 281.8737

Frequencies -- 282.7293 286.5236 287.0941

Frequencies -- 288.4216 289.7831 294.1996

Frequencies -- 300.6078 309.6303 312.8482

Frequencies -- 316.9265 322.2738 327.9857

Frequencies -- 350.9002 352.0359 355.6098

Frequencies -- 356.4071 358.2848 359.6473

Frequencies -- 397.8517 399.6429 399.7090

Frequencies -- 400.7924 411.2406 412.2242

Frequencies -- 448.6942 449.7418 451.2664

Frequencies -- 452.0082 454.0903 455.7607

Frequencies -- 457.3338 459.1966 459.5881

Frequencies -- 461.9400 470.1567 471.1851

Frequencies -- 716.6963 721.2401 723.0065

Frequencies -- 723.0918 725.2819 725.6452

Frequencies -- 726.0933 727.9772 729.4680

Frequencies -- 730.6267 732.6923 734.2654

Frequencies -- 736.3857 739.0416 739.6919

Frequencies -- 742.1456 742.5473 742.8355

Frequencies -- 772.1041 775.3366 779.8655

Frequencies -- 781.1039 781.3265 782.8288

Frequencies -- 796.5041 798.2395 798.3693

Frequencies -- 798.6425 799.2250 800.0890

Frequencies -- 804.8869 809.9604 813.9291

Frequencies -- 816.7435 819.6603 822.3722

Frequencies -- 823.6641 825.4277 829.8989

Frequencies -- 837.5979 842.6237 843.9779

Frequencies -- 887.9077 888.3802 889.1214

Frequencies -- 889.9213 891.0269 892.4970

Frequencies -- 928.4519 929.1751 929.4874

Frequencies -- 932.4878 933.0260 933.2631

Frequencies -- 1023.5130 1024.4440 1034.6305

Frequencies -- 1034.9445 1036.6836 1037.6516

Frequencies -- 1054.2806 1054.4798 1054.8623

Frequencies -- 1055.3804 1056.0913 1056.9531

Frequencies -- 1078.9088 1078.9932 1080.6781

Frequencies -- 1081.9239 1086.7676 1087.0592

Frequencies -- 1114.0612 1115.7086 1116.8947

Frequencies -- 1118.2187 1123.9866 1125.3821

Frequencies -- 1142.2423 1142.3628 1143.3137

Frequencies -- 1143.6875 1144.2314 1144.6160

Frequencies -- 1144.7343 1144.9092 1146.5778

Frequencies -- 1146.7935 1149.3326 1149.4698

Frequencies -- 1161.0746 1161.4888 1162.0649

Frequencies -- 1163.7062 1164.3295 1165.0321

Frequencies -- 1166.1348 1166.3806 1167.0245

Frequencies -- 1167.6086 1170.8341 1172.6246

Frequencies -- 1172.7451 1173.3787 1175.7504

Frequencies -- 1180.8996 1181.2172 1181.8998

Frequencies -- 1293.7995 1294.5466 1300.1459

Frequencies -- 1300.9913 1301.9132 1302.3366

Frequencies -- 1303.9591 1305.0372 1305.4645

Frequencies -- 1310.0137 1312.9468 1320.9645

Frequencies -- 1328.7303 1332.3193 1337.6144

Frequencies -- 1339.5403 1347.8762 1353.4556

Frequencies -- 1364.9217 1366.5441 1366.9762

Frequencies -- 1369.1947 1370.3122 1371.0036

Frequencies -- 1382.3733 1384.5321 1386.6265

Frequencies -- 1388.2967 1389.4928 1390.0215

Frequencies -- 1408.3681 1411.5692 1411.8503

Frequencies -- 1411.9528 1412.5462 1413.0313

Frequencies -- 1429.8071 1430.5721 1432.9129

Frequencies -- 1434.5501 1435.3710 1436.4210

Frequencies -- 1436.9063 1437.0096 1437.6813

Frequencies -- 1437.7343 1437.9769 1439.0446

Frequencies -- 1439.1324 1439.4342 1440.8366

Frequencies -- 1441.1966 1442.4871 1446.1978

Frequencies -- 1451.8516 1452.0346 1452.7156

Frequencies -- 1453.5985 1453.6553 1454.4050

Frequencies -- 1455.1929 1456.1151 1456.3406

Frequencies -- 1457.5442 1458.8978 1460.8155

Frequencies -- 1470.8800 1471.7960 1473.7693

Frequencies -- 1475.6351 1478.0225 1483.0171

Frequencies -- 1484.7022 1484.9329 1488.2524

Frequencies -- 1489.3739 1491.5010 1499.5041

Frequencies -- 1511.8986 1515.7046 1524.8254

Frequencies -- 1527.2952 1529.1176 1531.9879

Frequencies -- 1593.6792 1599.0492 1602.4859

Frequencies -- 1605.0832 1610.4018 1617.8678

Frequencies -- 1659.9761 1662.2538 1672.0093

Frequencies -- 1674.2575 1697.8706 1728.6126

Frequencies -- 3034.6596 3035.3203 3040.7371

Frequencies -- 3047.6039 3052.6365 3052.9023

Frequencies -- 3053.2093 3054.5827 3055.2634

Frequencies -- 3055.3926 3055.8697 3056.4397

Frequencies -- 3057.5487 3057.6546 3057.8222

Frequencies -- 3057.8721 3058.7909 3058.9983

Frequencies -- 3060.5132 3060.6913 3060.9693

Frequencies -- 3061.4120 3069.0594 3070.1062

Frequencies -- 3112.2284 3112.3734 3115.0019

Frequencies -- 3117.1588 3118.3432 3121.0514

Frequencies -- 3123.2587 3124.2203 3127.9912

Frequencies -- 3128.1811 3133.3671 3142.1738

Frequencies -- 3169.6474 3170.6546 3171.4670

Frequencies -- 3172.3228 3172.7324 3172.9564

Frequencies -- 3173.1375 3173.9825 3174.0167

Frequencies -- 3174.3723 3174.9887 3176.8603

Frequencies -- 3177.7546 3178.3057 3180.1046

Frequencies -- 3180.3013 3180.5615 3181.2107

Frequencies -- 3181.2334 3181.4407 3181.9223

Frequencies -- 3185.3037 3186.1113 3188.4855

Frequencies -- 3242.5761 3242.8500 3243.0122

Frequencies -- 3243.9405 3244.0240 3244.7601

SCF Done: E(RM06L-D3/def2-SVP/SMD) = -3872.59096710

Sum of electronic and zero-point Energies= -3871.508193

Sum of electronic and thermal Energies= -3871.426469

Sum of electronic and thermal Free Energies= -3871.631061

SCF Done: E(RM06L-D3/def2-TZVPP/SMD) = -3876.44927315

**P5**

Center Atomic Atomic Coordinates (Angstroms)

Number Number Type X Y Z

---------------------------------------------------------------------

1 83 0 0.102322 -0.001891 0.581629

2 8 0 1.193783 1.728121 1.836685

3 8 0 2.245372 -0.605377 0.552617

4 6 0 3.429383 1.428482 1.055988

5 1 0 4.400253 1.911376 0.969958

6 6 0 3.300314 0.129295 0.574498

7 6 0 2.375093 2.133847 1.668913

8 8 0 2.706901 3.350013 2.105559

9 8 0 4.408431 -0.421935 0.087656

10 6 0 1.666636 4.147481 2.692963

11 1 0 0.825360 4.216605 1.985957

12 1 0 1.285127 3.635860 3.590573

13 6 0 4.316254 -1.733294 -0.499718

14 1 0 4.005653 -2.451895 0.274559

15 1 0 3.522566 -1.729970 -1.262700

16 6 0 2.242116 5.492287 3.014172

17 1 0 3.077344 5.414534 3.722879

18 1 0 2.607423 5.998356 2.110402

19 1 0 1.474794 6.131569 3.469002

20 6 0 5.656949 -2.071029 -1.075170

21 1 0 5.948041 -1.361559 -1.861388

22 1 0 6.439156 -2.064690 -0.304383

23 1 0 5.630756 -3.074214 -1.519202

24 35 0 -0.298292 -2.324995 -0.791745

25 35 0 0.294208 1.597726 -1.543359

---------------------------------------------------------------------

Frequencies -- 10.8306 33.1671 52.3954

Frequencies -- 54.6459 61.4644 75.8848

Frequencies -- 83.7543 104.8771 115.2056

Frequencies -- 139.3808 146.0786 176.6835

Frequencies -- 180.1949 192.3695 198.6658

Frequencies -- 203.7159 241.3367 274.7305

Frequencies -- 280.7021 287.6860 345.9836

Frequencies -- 397.6042 449.1664 474.5097

Frequencies -- 719.9900 738.3517 742.1798

Frequencies -- 781.6207 799.4740 813.0227

Frequencies -- 820.1895 885.7497 922.5522

Frequencies -- 1032.4691 1046.8878 1069.6503

Frequencies -- 1114.3693 1142.1914 1143.9878

Frequencies -- 1163.3785 1164.3509 1192.5101

Frequencies -- 1277.4829 1300.7729 1342.7405

Frequencies -- 1362.4529 1392.2858 1407.2332

Frequencies -- 1435.0177 1438.7909 1442.3512

Frequencies -- 1453.7161 1456.2782 1470.4153

Frequencies -- 1490.3211 1497.5996 1607.9003

Frequencies -- 1663.3743 3063.9723 3068.8291

Frequencies -- 3070.5033 3079.7475 3135.5644

Frequencies -- 3144.6606 3181.0118 3186.4640

Frequencies -- 3187.4838 3191.3265 3250.1949

SCF Done: E(RM06L-D3/def2-SVP/SMD) = -5936.18890332

Sum of electronic and zero-point Energies= -5936.007852

Sum of electronic and thermal Energies= -5935.989535

Sum of electronic and thermal Free Energies= -5936.060088

SCF Done: E(RM06L-D3/def2-TZVPP/SMD) = -5937.4681671

**P6**

Center Atomic Atomic Coordinates (Angstroms)

Number Number Type X Y Z

---------------------------------------------------------------------

1 83 0 -0.018977 -1.081302 1.107453

2 8 0 0.725701 1.011642 1.985245

3 8 0 2.139008 -0.883653 0.345296

4 6 0 2.482180 1.490280 0.435160

5 1 0 3.055772 2.302065 -0.008414

6 6 0 2.690290 0.192945 -0.046411

7 6 0 1.531499 1.804400 1.423703

8 8 0 1.498617 3.096674 1.769746

9 8 0 3.585707 0.099616 -1.037346

10 6 0 0.492066 3.503386 2.707118

11 1 0 -0.497890 3.193141 2.333577

12 1 0 0.649296 2.976485 3.661758

13 6 0 3.692049 -1.165348 -1.708605

14 1 0 4.095718 -1.914523 -1.009551

15 1 0 2.682123 -1.508828 -1.986866

16 6 0 0.587157 4.989756 2.866260

17 1 0 1.573520 5.292772 3.242968

18 1 0 0.415947 5.506272 1.912039

19 1 0 -0.168111 5.342571 3.580277

20 6 0 4.576940 -0.976637 -2.902272

21 1 0 4.156290 -0.242164 -3.603134

22 1 0 5.578249 -0.632074 -2.610685

23 1 0 4.692845 -1.925655 -3.441291

24 8 0 -2.032218 0.064976 1.430520

25 6 0 -2.275620 1.246695 1.045731

26 6 0 -1.698795 1.923758 -0.040554

27 8 0 -3.189533 1.953135 1.716102

28 1 0 -2.041915 2.930023 -0.270631

29 6 0 -0.745119 1.338958 -0.877307

30 6 0 -3.802321 1.336239 2.858886

31 8 0 -0.394464 2.057660 -1.941763

32 1 0 -4.250751 0.378160 2.554749

33 1 0 -3.022165 1.099826 3.599963

34 6 0 -4.821773 2.287857 3.403767

35 6 0 0.616478 1.522353 -2.813303

36 1 0 -4.362084 3.235363 3.715842

37 1 0 -5.596068 2.514479 2.658730

38 1 0 -5.316466 1.849356 4.279827

39 1 0 1.544474 1.381030 -2.236674

40 1 0 0.301039 0.527188 -3.163713

41 6 0 0.798019 2.483368 -3.947110

42 1 0 -0.133056 2.615241 -4.514608

43 1 0 1.122719 3.470499 -3.591391

44 1 0 1.562480 2.107623 -4.639322

45 8 0 -0.191977 0.198357 -0.741571

46 35 0 -0.611910 -3.125200 -0.645605

---------------------------------------------------------------------

Frequencies -- 24.9517 36.3715 41.0482

Frequencies -- 50.7208 50.8719 68.6669

Frequencies -- 80.3662 86.5545 87.8037

Frequencies -- 97.4102 109.4977 112.5225

Frequencies -- 124.1597 133.2571 139.7928

Frequencies -- 150.6515 159.9400 165.8408

Frequencies -- 170.2949 178.1871 183.1857

Frequencies -- 190.6549 206.7079 211.3787

Frequencies -- 216.0104 228.8145 241.9531

Frequencies -- 253.8321 277.4076 285.2780

Frequencies -- 289.6080 291.4439 297.4881

Frequencies -- 302.8569 353.4487 360.0164

Frequencies -- 399.2257 407.7187 449.9214

Frequencies -- 457.8225 464.0324 479.5081

Frequencies -- 717.6469 721.8484 727.8280

Frequencies -- 736.2909 736.7963 742.6113

Frequencies -- 773.4310 780.3621 797.0125

Frequencies -- 802.6956 818.5505 821.5508

Frequencies -- 829.6081 844.4098 888.5183

Frequencies -- 890.9628 928.0746 930.5938

Frequencies -- 1031.2513 1035.2264 1052.2878

Frequencies -- 1052.7965 1073.1902 1077.7646

Frequencies -- 1107.6190 1116.1520 1143.6161

Frequencies -- 1145.1113 1145.7636 1147.9030

Frequencies -- 1166.4219 1166.7293 1167.5430

Frequencies -- 1170.0659 1181.2475 1188.1111

Frequencies -- 1298.0355 1298.7564 1299.1072

Frequencies -- 1311.5674 1340.4668 1355.0800

Frequencies -- 1362.8239 1371.2008 1388.6741

Frequencies -- 1398.6070 1410.2777 1413.5268

Frequencies -- 1435.0512 1438.5901 1439.4005

Frequencies -- 1439.5409 1441.6050 1447.0255

Frequencies -- 1455.1254 1458.3641 1459.5279

Frequencies -- 1463.2184 1476.5640 1478.5369

Frequencies -- 1491.7155 1492.6372 1506.2405

Frequencies -- 1515.6304 1595.8088 1600.4951

Frequencies -- 1651.8083 1680.1007 3058.2295

Frequencies -- 3058.6212 3059.7275 3060.9551

Frequencies -- 3063.0933 3065.4341 3069.8082

Frequencies -- 3077.1702 3120.5766 3121.6139

Frequencies -- 3135.9712 3146.5800 3171.4739

Frequencies -- 3176.0950 3180.0875 3182.4583

Frequencies -- 3184.0107 3184.1419 3186.1438

Frequencies -- 3187.4617 3242.1804 3247.1631

SCF Done: E(RM06L-D3/def2-SVP/SMD) = -3936.23135888

Sum of electronic and zero-point Energies= -3935.869657

Sum of electronic and thermal Energies= -3935.840910

Sum of electronic and thermal Free Energies= -3935.931102

SCF Done: E(RM06L-D3/def2-TZVPP/SMD) = -3937.83769817

**P7**

Center Atomic Atomic Coordinates (Angstroms)

Number Number Type X Y Z

---------------------------------------------------------------------

1 83 0 0.195446 -0.293578 2.109460

2 83 0 0.674993 0.438692 -1.406498

3 8 0 0.154424 1.918201 0.460004

4 8 0 -0.566463 2.118349 -2.263021

5 8 0 -1.008124 -2.164157 2.573811

6 8 0 1.062867 -1.989242 0.605041

7 6 0 -0.700440 -3.601396 0.673343

8 6 0 -0.250522 3.937894 -0.723050

9 1 0 -0.275398 5.022056 -0.640236

10 1 0 -1.095493 -4.491178 0.187392

11 6 0 -0.560173 3.359360 -1.959098

12 6 0 0.066764 3.180894 0.413270

13 6 0 0.466451 -3.018009 0.163312

14 6 0 -1.326803 -3.157794 1.846481

15 8 0 0.987215 -3.628602 -0.908653

16 8 0 -2.361393 -3.896010 2.243983

17 8 0 0.292911 3.880756 1.528456

18 8 0 -0.901551 4.215378 -2.918510

19 6 0 2.262428 -3.169861 -1.381750

20 1 0 2.209462 -2.095681 -1.631163

21 1 0 3.004067 -3.263072 -0.571145

22 6 0 -3.067447 -3.487126 3.427948

23 1 0 -2.370380 -3.485634 4.280381

24 1 0 -3.418223 -2.451054 3.296962

25 6 0 0.664905 3.152047 2.706197

26 1 0 -0.184177 2.513165 3.011373

27 1 0 1.524468 2.498591 2.469232

28 6 0 -1.145794 3.689426 -4.236096

29 1 0 -0.290338 3.065593 -4.538327

30 1 0 -2.022648 3.024504 -4.200202

31 6 0 2.631812 -3.973070 -2.587819

32 1 0 2.721842 -5.041477 -2.349727

33 1 0 1.884431 -3.855979 -3.383579

34 1 0 3.597260 -3.629397 -2.981190

35 6 0 -4.205644 -4.437837 3.633793

36 1 0 -4.899521 -4.423811 2.782596

37 1 0 -3.850945 -5.468704 3.767511

38 1 0 -4.771207 -4.157392 4.531799

39 6 0 1.006821 4.130508 3.786890

40 1 0 1.847136 4.773258 3.493872

41 1 0 0.151535 4.773793 4.031023

42 1 0 1.294510 3.589525 4.697326

43 6 0 -1.355288 4.847801 -5.160604

44 1 0 -2.215033 5.459724 -4.856399

45 1 0 -0.470649 5.497531 -5.196328

46 1 0 -1.545804 4.483882 -6.178300

47 35 0 0.879043 -0.635273 -3.987605

48 35 0 1.145473 -0.365991 4.709063

49 8 0 -1.865448 0.514168 2.781781

50 6 0 -3.009017 0.075024 2.453080

51 6 0 -3.412302 -0.481387 1.227683

52 8 0 -3.983556 0.138941 3.365355

53 1 0 -4.453019 -0.783137 1.124811

54 6 0 -2.566258 -0.657627 0.126889

55 6 0 -3.626104 0.517928 4.704675

56 8 0 -1.316801 -0.428979 0.098271

57 8 0 -3.160394 -1.125216 -0.975748

58 1 0 -2.729543 -0.044460 5.010791

59 1 0 -3.350483 1.584199 4.716377

60 6 0 -4.801227 0.230453 5.588073

61 6 0 -2.349698 -1.330512 -2.137372

62 1 0 -5.691211 0.790982 5.272237

63 1 0 -5.052742 -0.839364 5.582049

64 1 0 -4.573613 0.515406 6.623102

65 1 0 -1.986779 -0.349458 -2.497461

66 1 0 -1.470543 -1.943342 -1.871292

67 6 0 -3.178111 -2.002798 -3.188170

68 1 0 -3.529749 -2.986236 -2.849379

69 1 0 -4.053956 -1.398706 -3.458695

70 1 0 -2.574045 -2.150373 -4.092072

71 8 0 2.379381 1.801933 -2.009061

72 6 0 3.539553 1.268536 -2.111823

73 6 0 4.123211 0.357283 -1.223994

74 8 0 4.284780 1.617951 -3.155841

75 1 0 5.124319 -0.005493 -1.449644

76 6 0 3.581388 0.072037 0.049952

77 6 0 3.681717 2.463605 -4.153532

78 8 0 2.436110 0.407614 0.457421

79 8 0 4.405823 -0.601218 0.857702

80 1 0 2.721618 2.020876 -4.460440

81 1 0 3.455408 3.442329 -3.702267

82 6 0 4.640270 2.582050 -5.297244

83 6 0 3.936167 -0.908518 2.177529

84 1 0 5.598642 3.014329 -4.979349

85 1 0 4.842301 1.604085 -5.754346

86 1 0 4.218234 3.235010 -6.071849

87 1 0 3.668281 0.027747 2.694083

88 1 0 3.022271 -1.521123 2.096805

89 6 0 5.018932 -1.644788 2.903743

90 1 0 5.278848 -2.582315 2.394225

91 1 0 5.930249 -1.037670 2.986752

92 1 0 4.683816 -1.892556 3.919183

---------------------------------------------------------------------

Frequencies -- 12.8019 23.6430 24.5554

Frequencies -- 27.6498 31.7746 33.1579

Frequencies -- 39.4147 42.0060 46.7523

Frequencies -- 48.4980 51.6889 52.1280

Frequencies -- 53.6756 57.3173 59.3281

Frequencies -- 63.6802 65.3060 72.0252

Frequencies -- 74.0405 78.6918 83.4629

Frequencies -- 86.1879 89.0786 90.0640

Frequencies -- 93.2676 98.6967 103.2964

Frequencies -- 108.7133 113.6068 115.0922

Frequencies -- 117.6605 119.9229 120.2829

Frequencies -- 133.1680 135.7551 143.2932

Frequencies -- 153.8563 156.0187 166.4039

Frequencies -- 169.2739 169.7561 173.4743

Frequencies -- 174.3527 175.7554 178.0945

Frequencies -- 183.0085 184.9275 188.4323

Frequencies -- 190.3394 198.0446 204.4530

Frequencies -- 204.5945 206.9637 212.1174

Frequencies -- 218.0545 223.4714 228.2658

Frequencies -- 234.7233 241.3881 249.1492

Frequencies -- 252.0939 260.0381 277.1793

Frequencies -- 277.7135 278.3105 285.1378

Frequencies -- 286.1763 291.6107 295.3083

Frequencies -- 298.3260 300.8269 303.1742

Frequencies -- 309.3812 310.1201 353.1996

Frequencies -- 356.0768 359.3382 360.3715

Frequencies -- 400.6265 406.2677 407.1588

Frequencies -- 409.9402 450.7336 452.0228

Frequencies -- 455.7988 457.5250 461.4533

Frequencies -- 466.4955 469.9712 474.7597

Frequencies -- 713.9958 716.1885 717.4411

Frequencies -- 719.2158 728.0103 734.1345

Frequencies -- 735.3040 736.1180 737.2514

Frequencies -- 738.3482 743.4561 752.2458

Frequencies -- 774.4673 776.4310 782.7837

Frequencies -- 786.3082 795.0986 797.5152

Frequencies -- 800.2895 801.5651 816.5096

Frequencies -- 820.5985 823.6304 836.6761

Frequencies -- 837.4777 847.7185 851.3788

Frequencies -- 859.0047 884.5242 887.3076

Frequencies -- 889.7472 893.9179 924.4476

Frequencies -- 925.1847 928.8398 933.9684

Frequencies -- 1026.1126 1029.3155 1034.3350

Frequencies -- 1035.6329 1048.8463 1051.3902

Frequencies -- 1052.4813 1055.0000 1070.1379

Frequencies -- 1072.2195 1074.5679 1080.3342

Frequencies -- 1106.9001 1109.4509 1111.6234

Frequencies -- 1121.7869 1145.0066 1145.4062

Frequencies -- 1145.7816 1147.0370 1147.6994

Frequencies -- 1149.7440 1152.3587 1152.9729

Frequencies -- 1159.6284 1161.8104 1165.1508

Frequencies -- 1166.3524 1167.3815 1171.6373

Frequencies -- 1180.4391 1183.6831 1184.9294

Frequencies -- 1186.0546 1187.7244 1190.1751

Frequencies -- 1276.8323 1283.4018 1295.6172

Frequencies -- 1297.0298 1297.9656 1310.1423

Frequencies -- 1314.2108 1317.2635 1337.1380

Frequencies -- 1340.2621 1347.7814 1354.9257

Frequencies -- 1358.2494 1359.9862 1367.4992

Frequencies -- 1376.6826 1390.5667 1391.4005

Frequencies -- 1392.7921 1395.2352 1406.7429

Frequencies -- 1408.6083 1411.9718 1416.3579

Frequencies -- 1433.0182 1436.1715 1437.0095

Frequencies -- 1439.0805 1439.6979 1440.7087

Frequencies -- 1441.1632 1441.4334 1441.9615

Frequencies -- 1442.2048 1443.1821 1444.1893

Frequencies -- 1445.5969 1455.4908 1455.6968

Frequencies -- 1456.5690 1458.9424 1459.4154

Frequencies -- 1460.9523 1462.4076 1467.6460

Frequencies -- 1473.6552 1476.3271 1479.3263

Frequencies -- 1483.4115 1488.5890 1493.5821

Frequencies -- 1494.9430 1500.9390 1505.5010

Frequencies -- 1506.0323 1513.2485 1588.7188

Frequencies -- 1594.0484 1598.8517 1603.9205

Frequencies -- 1639.8130 1644.0844 1653.8923

Frequencies -- 1682.3720 3011.6404 3012.0013

Frequencies -- 3043.9404 3052.3617 3054.7382

Frequencies -- 3059.9215 3060.5218 3061.3046

Frequencies -- 3062.5803 3063.9024 3064.0641

Frequencies -- 3064.5195 3065.3107 3065.8742

Frequencies -- 3079.1899 3079.2069 3079.8382

Frequencies -- 3084.4030 3105.8578 3119.9502

Frequencies -- 3122.3016 3129.8801 3145.4370

Frequencies -- 3149.4150 3174.1447 3175.6216

Frequencies -- 3178.1195 3178.6903 3180.6737

Frequencies -- 3180.8698 3182.3515 3182.6156

Frequencies -- 3183.0651 3183.8214 3184.2899

Frequencies -- 3186.1900 3187.3632 3188.8973

Frequencies -- 3189.2617 3194.1711 3241.4418

Frequencies -- 3245.9443 3246.9110 3250.2739

SCF Done: E(RM06L-D3/def2-SVP/SMD) = -7872.49686413

Sum of electronic and zero-point Energies= -7871.773306

Sum of electronic and thermal Energies= -7871.713657

Sum of electronic and thermal Free Energies= -7871.874194

SCF Done: E(RM06L-D3/def2-TZVPP/SMD) = -7875.70437783

**P8**

Center Atomic Atomic Coordinates (Angstroms)

Number Number Type X Y Z

---------------------------------------------------------------------

1 83 0 0.274087 0.078102 2.160976

2 83 0 0.414610 0.580629 -1.861787

3 35 0 1.320801 -1.771874 -2.752635

4 35 0 1.355809 0.570033 4.613991

5 8 0 -1.774949 0.576101 2.957239

6 6 0 -2.922529 0.117232 2.631772

7 6 0 -3.292197 -0.523754 1.445827

8 8 0 -3.899914 0.256739 3.519615

9 1 0 -4.308840 -0.905169 1.379618

10 6 0 -2.465994 -0.698022 0.327389

11 6 0 -3.585123 0.826368 4.804247

12 8 0 -1.262327 -0.307813 0.192062

13 8 0 -3.061958 -1.340693 -0.680773

14 1 0 -2.751238 0.262766 5.250217

15 1 0 -3.231436 1.859022 4.662005

16 6 0 -4.825150 0.764665 5.640725

17 6 0 -2.279135 -1.686410 -1.824137

18 1 0 -5.645281 1.335173 5.184749

19 1 0 -5.165221 -0.270370 5.778516

20 1 0 -4.630908 1.190520 6.633169

21 1 0 -1.990271 -0.759252 -2.358071

22 1 0 -1.359550 -2.195946 -1.493592

23 6 0 -3.102843 -2.552064 -2.726528

24 1 0 -3.402479 -3.479687 -2.221753

25 1 0 -4.010020 -2.032170 -3.060736

26 1 0 -2.518450 -2.822446 -3.615355

27 8 0 2.254525 1.737063 -2.448068

28 6 0 3.490352 1.517218 -2.205552

29 6 0 4.041146 0.704327 -1.210609

30 8 0 4.371278 2.125188 -2.991970

31 1 0 5.120699 0.570699 -1.214473

32 6 0 3.313918 0.060392 -0.199450

33 6 0 3.869598 2.916048 -4.087208

34 8 0 2.059399 0.129572 0.004876

35 8 0 4.069399 -0.681278 0.615215

36 1 0 3.203391 2.292013 -4.702441

37 1 0 3.253606 3.733245 -3.682490

38 6 0 5.044564 3.427632 -4.860354

39 6 0 3.417966 -1.476486 1.608164

40 1 0 5.695221 4.055213 -4.237046

41 1 0 5.649470 2.606082 -5.266203

42 1 0 4.697576 4.037948 -5.703627

43 1 0 2.946308 -0.808289 2.355507

44 1 0 2.624123 -2.072662 1.130020

45 6 0 4.436191 -2.344356 2.280086

46 1 0 4.917868 -3.022189 1.563588

47 1 0 5.216178 -1.746053 2.768387

48 1 0 3.946249 -2.955877 3.048565

49 35 0 -0.025803 -2.558164 2.488997

50 35 0 -0.762367 1.326627 -4.205970

---------------------------------------------------------------------

Frequencies -- 19.3389 22.5093 28.5025

Frequencies -- 30.7409 33.7747 37.0777

Frequencies -- 39.9440 44.7662 51.8750

Frequencies -- 53.9493 57.3247 60.4471

Frequencies -- 64.6526 70.1715 74.8247

Frequencies -- 81.8060 92.3320 97.5801

Frequencies -- 98.8937 100.7720 107.9528

Frequencies -- 117.3472 120.5983 138.3500

Frequencies -- 152.7813 159.7970 171.3134

Frequencies -- 174.9939 177.5587 180.5109

Frequencies -- 185.4902 186.1614 192.3115

Frequencies -- 197.1728 201.1955 204.1339

Frequencies -- 213.9696 222.3325 239.2660

Frequencies -- 242.0544 276.3342 282.2820

Frequencies -- 286.5595 292.6796 301.8173

Frequencies -- 309.9184 351.8429 356.4482

Frequencies -- 402.4816 404.8706 450.8113

Frequencies -- 456.0363 465.0339 471.4493

Frequencies -- 718.5231 721.2707 735.9087

Frequencies -- 740.0803 740.6203 744.2493

Frequencies -- 782.0107 786.3983 793.9289

Frequencies -- 797.1618 820.5502 832.8657

Frequencies -- 850.6765 867.4107 884.6541

Frequencies -- 889.6420 928.4613 933.2882

Frequencies -- 1022.7904 1024.1443 1050.4174

Frequencies -- 1051.0979 1075.9914 1079.5341

Frequencies -- 1122.7001 1127.0366 1144.5619

Frequencies -- 1145.9811 1150.8793 1152.1349

Frequencies -- 1160.0545 1164.3449 1183.8362

Frequencies -- 1188.3532 1197.7800 1201.6531

Frequencies -- 1297.5607 1302.0543 1306.4997

Frequencies -- 1316.0602 1345.3875 1354.0716

Frequencies -- 1362.2825 1371.5956 1392.0685

Frequencies -- 1394.8081 1407.2515 1412.3465

Frequencies -- 1437.1002 1440.1388 1440.2188

Frequencies -- 1440.5064 1441.1656 1442.5889

Frequencies -- 1453.5123 1460.0129 1460.1658

Frequencies -- 1463.5545 1470.6775 1475.2577

Frequencies -- 1492.7430 1497.3833 1503.6389

Frequencies -- 1514.4838 1607.6433 1611.1974

Frequencies -- 1630.7657 1644.5128 3002.6919

Frequencies -- 3009.4745 3064.1850 3066.0680

Frequencies -- 3066.1644 3069.8878 3072.4015

Frequencies -- 3073.2872 3114.1477 3114.8869

Frequencies -- 3140.1895 3140.8290 3181.6051

Frequencies -- 3182.3000 3187.1502 3187.9928

Frequencies -- 3189.8071 3190.2411 3190.8721

Frequencies -- 3192.8325 3245.3965 3248.3184

SCF Done: E(RM06L-D3/def2-SVP/SMD) = -11872.4024873

Sum of electronic and zero-point Energies= -11872.038468

Sum of electronic and thermal Energies= -11872.000361

Sum of electronic and thermal Free Energies= -11872.117877

SCF Done: E(RM06L-D3/def2-TZVPP/SMD) = -11874.9569347

**P9**

Center Atomic Atomic Coordinates (Angstroms)

Number Number Type X Y Z

---------------------------------------------------------------------

1 83 0 7.738710 0.266791 2.833440

2 8 0 8.819095 1.127225 1.244304

3 83 0 9.272553 -0.609199 0.051747

4 8 0 8.135974 -1.463641 1.610539

5 8 0 6.652645 2.420430 2.642566

6 6 0 5.931897 2.699946 1.651039

7 6 0 5.043134 1.842879 0.977566

8 8 0 5.964262 3.953860 1.160224

9 6 0 4.874704 0.504989 1.348915

10 6 0 6.965595 4.826409 1.691137

11 8 0 5.563576 -0.150548 2.191266

12 8 0 3.865410 -0.135416 0.746956

13 1 0 6.780361 4.990422 2.764983

14 1 0 7.947907 4.331803 1.614177

15 6 0 6.917006 6.109233 0.917702

16 6 0 3.744764 -1.544710 0.983219

17 1 0 7.126118 5.944591 -0.148156

18 1 0 5.932278 6.590103 0.998092

19 1 0 7.665447 6.814746 1.301344

20 1 0 4.725889 -2.023183 0.835082

21 1 0 3.466634 -1.709054 2.037214

22 6 0 2.707482 -2.084081 0.045620

23 1 0 1.749339 -1.558995 0.162892

24 1 0 3.023849 -1.992574 -1.002260

25 1 0 2.528552 -3.148186 0.247760

26 1 0 4.449794 2.229792 0.152113

27 8 0 7.904718 -2.154086 -1.186708

28 6 0 6.679820 -1.924006 -1.364557

29 6 0 6.077796 -0.678046 -1.612581

30 8 0 5.820055 -2.959196 -1.337889

31 6 0 6.832218 0.495968 -1.730119

32 6 0 6.341960 -4.222982 -0.915403

33 8 0 8.074262 0.625223 -1.508547

34 8 0 6.154451 1.578149 -2.130960

35 1 0 7.054604 -4.594712 -1.669629

36 1 0 6.915172 -4.082475 0.015235

37 6 0 5.187252 -5.160266 -0.729748

38 6 0 6.841010 2.835561 -2.065898

39 1 0 4.512744 -4.817795 0.067131

40 1 0 4.598379 -5.260645 -1.651987

41 1 0 5.549077 -6.159132 -0.452615

42 1 0 7.265915 2.960944 -1.056021

43 1 0 7.690881 2.818358 -2.767208

44 6 0 5.862770 3.917904 -2.407780

45 1 0 5.407241 3.748669 -3.393448

46 1 0 5.057336 3.986120 -1.664069

47 1 0 6.371702 4.889974 -2.441674

48 1 0 5.000207 -0.620471 -1.747611

---------------------------------------------------------------------

Frequencies -- 34.0996 42.2607 47.2162

Frequencies -- 52.2822 64.2843 67.2255

Frequencies -- 78.8683 81.6459 85.3587

Frequencies -- 98.2457 103.6857 108.9056

Frequencies -- 113.3843 132.1493 137.2740

Frequencies -- 140.1460 151.1665 168.0428

Frequencies -- 172.7059 176.4163 183.3297

Frequencies -- 186.0098 191.4875 202.2922

Frequencies -- 208.2379 219.1357 229.9552

Frequencies -- 239.7849 240.6355 249.2903

Frequencies -- 274.1122 283.3300 292.5601

Frequencies -- 304.8735 310.1060 323.0307

Frequencies -- 351.0246 356.3894 386.9880

Frequencies -- 395.7655 447.6807 449.7349

Frequencies -- 454.6329 463.6683 473.2924

Frequencies -- 476.6461 551.0911 576.6862

Frequencies -- 717.8419 719.1720 737.4158

Frequencies -- 741.9303 755.7132 767.1108

Frequencies -- 776.7168 785.8878 798.4481

Frequencies -- 799.6772 803.8765 813.4401

Frequencies -- 814.6238 820.6461 885.0462

Frequencies -- 890.8538 925.4209 930.8705

Frequencies -- 1032.7228 1036.3955 1049.9746

Frequencies -- 1053.7191 1080.1950 1082.8614

Frequencies -- 1113.3355 1115.9107 1140.3529

Frequencies -- 1141.9854 1142.9168 1144.0722

Frequencies -- 1165.3328 1166.1062 1169.0703

Frequencies -- 1172.1111 1174.1661 1176.5116

Frequencies -- 1285.3637 1287.9623 1294.9943

Frequencies -- 1311.0174 1335.2989 1345.9873

Frequencies -- 1354.8915 1369.4780 1386.9266

Frequencies -- 1391.0859 1407.3492 1412.9578

Frequencies -- 1430.0696 1436.0472 1439.6485

Frequencies -- 1441.1494 1448.9931 1450.2091

Frequencies -- 1455.4074 1455.8149 1460.2075

Frequencies -- 1463.3024 1477.4353 1480.0051

Frequencies -- 1492.1287 1493.6463 1518.0747

Frequencies -- 1520.5077 1591.9064 1595.7787

Frequencies -- 1671.4227 1691.8699 3055.0794

Frequencies -- 3055.6790 3057.4906 3058.5013

Frequencies -- 3058.6274 3060.6400 3061.9670

Frequencies -- 3062.5293 3118.6086 3120.1306

Frequencies -- 3121.0751 3123.4740 3170.8121

Frequencies -- 3172.7851 3175.6948 3175.7110

Frequencies -- 3179.8086 3181.9551 3183.7364

Frequencies -- 3185.9089 3251.9748 3262.4113

SCF Done: E(RM06L-D3/def2-SVP/SMD) = -1727.54464738

Sum of electronic and zero-point Energies= -1727.177609

Sum of electronic and thermal Energies= -1727.146899

Sum of electronic and thermal Free Energies= -1727.240423

SCF Done: E(RM06L-D3/def2-TZVPP/SMD) = -1729.04103393

**P10**

Center Atomic Atomic Coordinates (Angstroms)

Number Number Type X Y Z

---------------------------------------------------------------------

1 83 0 5.325089 5.222900 8.052679

2 8 0 4.696247 3.715112 6.217892

3 8 0 5.573783 6.388327 6.135340

4 8 0 6.682933 6.764953 8.660676

5 6 0 4.568007 0.285351 4.017429

6 1 0 4.105330 -0.604488 4.463468

7 6 0 4.557556 1.420029 4.995486

8 1 0 5.110772 1.177759 5.917820

9 6 0 5.234053 3.683443 5.083701

10 6 0 5.928067 4.732480 4.445171

11 1 0 6.396918 4.547198 3.481104

12 6 0 6.045830 5.998932 5.015035

13 6 0 7.957459 8.920119 4.070861

14 1 0 8.166850 9.902487 4.514091

15 6 0 6.977217 8.174710 4.923390

16 1 0 6.026141 8.721615 5.024606

17 83 0 8.076226 6.162443 10.056521

18 8 0 9.514303 7.684485 9.001355

19 8 0 7.673322 8.051517 11.232600

20 6 0 11.466840 9.786449 6.113185

21 1 0 12.125311 9.237546 5.427691

22 6 0 10.782061 8.829932 7.040145

23 1 0 11.503428 8.264476 7.649307

24 6 0 9.290705 8.923503 8.880103

25 6 0 8.451304 9.719239 9.677708

26 1 0 8.373547 10.778979 9.444174

27 6 0 7.742019 9.246177 10.785497

28 6 0 5.527874 10.965420 13.078777

29 1 0 4.874748 10.685278 13.914982

30 6 0 6.243474 9.757797 12.556016

31 1 0 5.546319 8.980446 12.205770

32 8 0 6.724453 6.903550 4.302575

33 8 0 5.171539 2.551716 4.367089

34 8 0 9.921446 9.585845 7.905634

35 8 0 7.066541 10.179501 11.456951

36 1 0 7.349923 8.008632 5.947225

37 1 0 7.564900 9.087608 3.058585

38 1 0 8.907621 8.376137 3.978205

39 1 0 3.534597 1.685122 5.305807

40 1 0 5.591412 0.018853 3.720607

41 1 0 4.005575 0.535570 3.107756

42 1 0 10.179744 8.083137 6.497289

43 1 0 12.084115 10.506511 6.667120

44 1 0 10.748615 10.353390 5.506520

45 1 0 6.877566 9.288881 13.324162

46 1 0 4.901858 11.427994 12.304072

47 1 0 6.232443 11.724739 13.443865

48 35 0 7.605383 3.642438 7.933636

49 35 0 5.779009 4.816502 11.243098

---------------------------------------------------------------------

Frequencies -- 7.2312 23.2802 26.2247

Frequencies -- 31.8865 35.5723 51.1456

Frequencies -- 57.2294 62.6092 63.6477

Frequencies -- 69.0236 75.3046 80.9967

Frequencies -- 86.4594 96.6331 99.7995

Frequencies -- 112.9500 117.6469 123.1271

Frequencies -- 128.2835 131.2965 145.5917

Frequencies -- 148.2544 151.8522 158.7201

Frequencies -- 165.0165 169.9446 174.5396

Frequencies -- 182.6684 190.5721 198.5815

Frequencies -- 208.7675 213.4503 219.3214

Frequencies -- 244.9504 247.2311 276.9028

Frequencies -- 280.2823 283.3662 284.9355

Frequencies -- 289.6648 328.4271 349.3617

Frequencies -- 355.0683 395.4325 404.2048

Frequencies -- 438.4734 449.5833 452.4014

Frequencies -- 467.5945 472.3498 616.8360

Frequencies -- 717.2481 722.9463 724.2215

Frequencies -- 734.4122 743.5052 744.5749

Frequencies -- 783.6697 786.4490 798.7678

Frequencies -- 799.6477 812.5737 819.7148

Frequencies -- 828.8598 834.9806 887.3025

Frequencies -- 887.4353 924.2841 926.1450

Frequencies -- 1029.5054 1038.9738 1048.2929

Frequencies -- 1053.0408 1071.5476 1079.4251

Frequencies -- 1110.8234 1116.5488 1141.7899

Frequencies -- 1142.1539 1144.0039 1146.4038

Frequencies -- 1161.4734 1165.4519 1168.8667

Frequencies -- 1170.1409 1180.3898 1188.1990

Frequencies -- 1279.2032 1293.2082 1303.7166

Frequencies -- 1310.9543 1339.8105 1351.5898

Frequencies -- 1360.5521 1370.1656 1388.4745

Frequencies -- 1394.3564 1409.1084 1412.3980

Frequencies -- 1436.5425 1437.1732 1440.1582

Frequencies -- 1441.3506 1442.2687 1443.8905

Frequencies -- 1452.5330 1456.9755 1459.1263

Frequencies -- 1469.1249 1477.0820 1481.0483

Frequencies -- 1492.5600 1496.4449 1507.4642

Frequencies -- 1512.8659 1604.7913 1605.7827

Frequencies -- 1660.6027 1683.8471 3057.9713

Frequencies -- 3061.2903 3061.5446 3062.8854

Frequencies -- 3067.7108 3068.5262 3069.9125

Frequencies -- 3070.2486 3124.3336 3130.9015

Frequencies -- 3133.4701 3134.2432 3175.3388

Frequencies -- 3177.1589 3178.5023 3181.1165

Frequencies -- 3182.6751 3184.4908 3185.2215

Frequencies -- 3189.7912 3247.0884 3247.9093

SCF Done: E(RM06L-D3/def2-SVP/SMD) = -6799.98339577

Sum of electronic and zero-point Energies= -6799.618237

Sum of electronic and thermal Energies= -6799.583481

Sum of electronic and thermal Free Energies= -6799.691584

SCF Done: E(RM06L-D3/def2-TZVPP/SMD) = -6802.00856369

**P11**

Center Atomic Atomic Coordinates (Angstroms)

Number Number Type X Y Z

---------------------------------------------------------------------

1 83 0 6.236268 8.264096 6.572350

2 8 0 5.222249 7.019293 8.273325

3 8 0 3.912702 8.668100 6.403234

4 8 0 5.958299 5.979852 5.835937

5 8 0 6.182583 8.237715 3.959232

6 8 0 6.869966 9.294138 8.379620

7 6 0 5.159251 3.256010 9.799484

8 1 0 6.065143 2.899585 10.307845

9 6 0 5.313967 4.700705 9.435230

10 1 0 6.181416 4.870994 8.776983

11 6 0 4.187492 6.292914 8.105091

12 6 0 3.083913 6.610631 7.312723

13 1 0 2.252651 5.910346 7.266453

14 6 0 3.012399 7.792072 6.552588

15 6 0 1.723902 9.101591 5.050190

16 1 0 2.543747 9.081153 4.314783

17 6 0 0.378356 9.029695 4.395487

18 1 0 0.239495 9.883614 3.720138

19 6 0 5.821035 1.913846 5.803714

20 1 0 5.767696 1.375684 6.758936

21 6 0 5.837943 3.396268 6.031745

22 1 0 6.695188 3.702469 6.657379

23 6 0 5.999537 5.374316 4.726925

24 6 0 6.115698 5.905350 3.436090

25 1 0 6.136332 5.205146 2.602790

26 6 0 6.182854 7.286847 3.150618

27 6 0 6.569555 8.977228 -0.039534

28 1 0 6.634212 10.020418 -0.375353

29 6 0 6.359998 8.918392 1.443812

30 1 0 5.440540 9.446792 1.746748

31 83 0 7.347776 7.983674 9.913216

32 8 0 7.972679 6.870308 7.752632

33 8 0 8.140086 5.580176 10.358681

34 8 0 9.605873 8.153198 9.668877

35 8 0 7.910718 9.864875 11.233621

36 6 0 9.992781 6.639613 4.159708

37 1 0 9.924130 7.477237 3.452962

38 6 0 9.082493 6.876939 5.326733

39 1 0 8.048930 6.998368 4.965378

40 6 0 8.676781 5.848374 7.448173

41 6 0 9.044586 4.799606 8.284800

42 1 0 9.675637 4.020638 7.860575

43 6 0 8.761736 4.748274 9.671262

44 6 0 9.097952 3.530130 11.676808

45 1 0 8.026251 3.576702 11.928854

46 6 0 9.717375 2.235672 12.109508

47 1 0 9.611601 2.104227 13.194041

48 6 0 13.163106 8.105977 7.721911

49 1 0 13.460839 7.184082 7.205812

50 6 0 11.858460 7.902305 8.428690

51 1 0 11.914797 7.094228 9.175216

52 6 0 10.324080 9.202330 9.687575

53 6 0 10.030146 10.433941 10.279640

54 1 0 10.766655 11.230900 10.203064

55 6 0 8.867560 10.663970 11.036015

56 6 0 7.834628 13.564269 12.949513

57 1 0 6.965087 13.841230 13.559165

58 6 0 7.651991 12.188705 12.383999

59 1 0 6.757196 12.122721 11.744852

60 8 0 1.846633 7.969177 5.922421

61 8 0 4.124427 5.127978 8.754355

62 8 0 6.269383 7.546371 1.824652

63 8 0 5.928739 4.028098 4.753647

64 8 0 9.157933 5.747691 6.195105

65 8 0 9.263885 3.643922 10.261099

66 8 0 11.517114 9.130925 9.086721

67 8 0 8.813214 11.874493 11.605505

68 1 0 9.363102 7.790499 5.880070

69 1 0 9.568736 4.396136 12.170148

70 1 0 9.235940 1.378972 11.618241

71 1 0 10.789021 2.204587 11.870361

72 1 0 9.713182 5.723608 3.621956

73 1 0 11.040422 6.547144 4.477803

74 1 0 7.184393 9.401087 1.993620

75 1 0 5.741598 8.500056 -0.581625

76 1 0 7.498983 8.469917 -0.332745

77 1 0 4.928379 3.745568 6.549335

78 1 0 6.729811 1.581851 5.283256

79 1 0 4.956468 1.610104 5.197790

80 1 0 11.051598 7.625919 7.732046

81 1 0 13.964384 8.371551 8.424529

82 1 0 13.092008 8.904172 6.970419

83 1 0 7.528418 11.431943 13.174358

84 1 0 7.941231 14.315403 12.155311

85 1 0 8.725327 13.619606 13.589873

86 1 0 5.460549 5.326090 10.331931

87 1 0 5.006010 2.629630 8.910272

88 1 0 4.309648 3.093790 10.476340

89 1 0 1.847467 10.026396 5.635114

90 1 0 0.269747 8.110923 3.803505

91 1 0 -0.430278 9.052452 5.138290

---------------------------------------------------------------------

Frequencies -- 19.4788 21.7928 23.5021

Frequencies -- 24.0881 28.7804 31.9993

Frequencies -- 39.8282 46.7121 49.4099

Frequencies -- 59.9566 60.9065 65.5280

Frequencies -- 70.2440 71.7736 73.9845

Frequencies -- 77.7783 84.5641 86.2373

Frequencies -- 88.9075 92.2252 93.8898

Frequencies -- 100.9207 102.5781 104.8337

Frequencies -- 106.5509 111.1214 117.5141

Frequencies -- 118.3656 124.3921 124.6111

Frequencies -- 132.0309 134.4037 138.9622

Frequencies -- 142.0299 142.6859 149.7877

Frequencies -- 152.0712 154.6029 161.2620

Frequencies -- 165.2514 169.5311 174.4372

Frequencies -- 176.2857 185.1072 192.9501

Frequencies -- 194.4618 197.1235 205.2903

Frequencies -- 208.1209 210.0266 221.1738

Frequencies -- 223.6459 227.9231 231.8569

Frequencies -- 244.5436 250.7326 259.0412

Frequencies -- 265.2781 268.2199 272.4511

Frequencies -- 277.8714 283.8599 284.1555

Frequencies -- 287.9563 292.3067 294.4265

Frequencies -- 296.8689 304.6545 312.6036

Frequencies -- 344.6138 347.1004 355.9846

Frequencies -- 359.1494 389.3115 398.9037

Frequencies -- 400.0067 408.7156 435.4404

Frequencies -- 440.9351 449.3329 453.3020

Frequencies -- 458.2186 458.8492 464.0348

Frequencies -- 470.0861 475.0057 574.8775

Frequencies -- 717.0294 721.3341 722.7129

Frequencies -- 723.7578 724.7830 729.2157

Frequencies -- 732.5016 735.1288 739.1721

Frequencies -- 745.5744 746.6440 749.3546

Frequencies -- 782.1085 783.9962 786.4106

Frequencies -- 788.5578 795.2806 795.7290

Frequencies -- 800.4944 803.4479 806.0740

Frequencies -- 811.0857 820.3482 821.5097

Frequencies -- 828.3419 834.1729 838.3780

Frequencies -- 843.9777 889.4764 889.9005

Frequencies -- 891.3876 894.9003 930.1968

Frequencies -- 933.3546 933.4168 936.4028

Frequencies -- 1024.7825 1027.1773 1033.5692

Frequencies -- 1034.7019 1052.0258 1052.2553

Frequencies -- 1053.8775 1053.9909 1077.6392

Frequencies -- 1079.5049 1086.2263 1090.2262

Frequencies -- 1115.9177 1116.3175 1127.0088

Frequencies -- 1129.2249 1143.1834 1144.3350

Frequencies -- 1144.4382 1145.5665 1145.8716

Frequencies -- 1146.6014 1146.8834 1147.5307

Frequencies -- 1157.8422 1160.1342 1163.7306

Frequencies -- 1165.4610 1170.1512 1171.0186

Frequencies -- 1172.3599 1173.1023 1179.4595

Frequencies -- 1181.4059 1182.8666 1188.5334

Frequencies -- 1278.4667 1291.9888 1294.7354

Frequencies -- 1295.9837 1297.2586 1299.0623

Frequencies -- 1306.5323 1320.5618 1321.5996

Frequencies -- 1326.3578 1341.3870 1346.5584

Frequencies -- 1364.6638 1365.0590 1366.1912

Frequencies -- 1370.5023 1380.8609 1384.6901

Frequencies -- 1395.9079 1397.2356 1407.0263

Frequencies -- 1411.9112 1414.0830 1419.0904

Frequencies -- 1425.3557 1428.1241 1435.1137

Frequencies -- 1435.9030 1437.6910 1438.0906

Frequencies -- 1439.9948 1440.7502 1441.9254

Frequencies -- 1442.3458 1442.6194 1444.1337

Frequencies -- 1452.2879 1452.3735 1452.6269

Frequencies -- 1457.4276 1457.7864 1457.9833

Frequencies -- 1458.6028 1461.2948 1472.0458

Frequencies -- 1478.9667 1480.2556 1483.8617

Frequencies -- 1486.9046 1493.1521 1494.5918

Frequencies -- 1496.1861 1504.0651 1510.6185

Frequencies -- 1516.5420 1535.1601 1600.8132

Frequencies -- 1602.9606 1612.6447 1619.5016

Frequencies -- 1655.2176 1669.2502 1702.1721

Frequencies -- 1715.1288 3035.3120 3038.1847

Frequencies -- 3047.2356 3055.5766 3056.9496

Frequencies -- 3058.1352 3058.2563 3058.4896

Frequencies -- 3058.9666 3059.1365 3059.6876

Frequencies -- 3063.5575 3067.1920 3068.7135

Frequencies -- 3075.8136 3078.4644 3105.5359

Frequencies -- 3106.4568 3115.0950 3119.0553

Frequencies -- 3126.7239 3132.9694 3140.1285

Frequencies -- 3141.5317 3171.8468 3172.1170

Frequencies -- 3173.1539 3173.4631 3178.1230

Frequencies -- 3180.0765 3180.3382 3180.3728

Frequencies -- 3181.3099 3182.3884 3182.5299

Frequencies -- 3183.7122 3184.7251 3184.7334

Frequencies -- 3185.4685 3187.6943 3237.9680

Frequencies -- 3240.7939 3245.4873 3251.1236

SCF Done: E(RM06L-D3/def2-SVP/SMD) = -2800.05160385

Sum of electronic and zero-point Energies= -2799.327105

Sum of electronic and thermal Energies= -2799.270349

Sum of electronic and thermal Free Energies= -2799.422524

SCF Done: E(RM06L-D3/def2-TZVPP/SMD) = -2802.72798326

**P12**

Center Atomic Atomic Coordinates (Angstroms)

Number Number Type X Y Z

---------------------------------------------------------------------

1 83 0 -0.325769 -0.038722 1.741326

2 8 0 1.236392 1.731253 2.214378

3 8 0 1.692059 -0.632972 0.839241

4 8 0 0.152803 -2.277388 2.515949

5 8 0 -1.018755 -1.495056 0.122354

6 6 0 0.248331 -3.489486 0.469631

7 6 0 2.902768 1.401578 0.544151

8 6 0 2.618256 0.045673 0.296950

9 6 0 2.154473 2.167770 1.475482

10 6 0 -0.551757 -2.606301 -0.278783

11 6 0 0.561433 -3.250154 1.833572

12 8 0 -0.850807 -2.978866 -1.521856

13 8 0 1.342123 -4.163770 2.412117

14 8 0 2.487402 3.455988 1.552321

15 8 0 3.397002 -0.581333 -0.584520

16 6 0 -1.608214 -2.058944 -2.326136

17 1 0 -2.531812 -1.783653 -1.793264

18 1 0 -1.026756 -1.130169 -2.449989

19 6 0 1.697462 -3.948102 3.785033

20 1 0 2.178754 -2.962343 3.881408

21 1 0 0.780780 -3.912790 4.394173

22 6 0 1.720912 4.281872 2.439639

23 1 0 0.670172 4.281508 2.108016

24 1 0 1.733561 3.841542 3.448336

25 6 0 3.053474 -1.936028 -0.922286

26 1 0 3.025118 -2.545930 -0.005512

27 1 0 2.032431 -1.953703 -1.340657

28 6 0 -1.890323 -2.719486 -3.639994

29 1 0 -0.963988 -2.981694 -4.168378

30 1 0 -2.477638 -3.638112 -3.508078

31 1 0 -2.465897 -2.040050 -4.281667

32 6 0 2.606672 -5.061640 4.204336

33 1 0 2.115720 -6.039012 4.105322

34 1 0 3.524144 -5.080528 3.601030

35 1 0 2.896997 -4.935558 5.255212

36 6 0 2.316946 5.655771 2.416520

37 1 0 3.357219 5.646927 2.768437

38 1 0 2.305127 6.081367 1.404199

39 1 0 1.744240 6.325746 3.070359

40 6 0 4.068905 -2.434963 -1.903239

41 1 0 4.073093 -1.833820 -2.822507

42 1 0 5.081696 -2.411342 -1.479161

43 1 0 3.842552 -3.472785 -2.179129

44 8 0 -2.587470 0.613176 1.190129

45 6 0 -3.103198 0.771452 0.054999

46 6 0 -2.406299 1.156167 -1.120093

47 8 0 -4.414768 0.579671 -0.090834

48 6 0 -1.018559 1.381818 -1.123835

49 6 0 -5.151181 0.173357 1.071183

50 8 0 -0.482733 1.762869 -2.282165

51 1 0 -4.703515 -0.746624 1.478760

52 1 0 -5.050619 0.945433 1.850066

53 6 0 -6.578036 -0.026881 0.662781

54 6 0 0.945058 1.924438 -2.327884

55 1 0 -7.011067 0.896400 0.255081

56 1 0 -6.671459 -0.811578 -0.099691

57 1 0 -7.179388 -0.326054 1.530713

58 1 0 1.254920 2.644496 -1.554329

59 1 0 1.421742 0.962205 -2.075136

60 6 0 1.314972 2.380006 -3.705172

61 1 0 1.012576 1.648917 -4.466958

62 1 0 0.843649 3.341203 -3.949988

63 1 0 2.402869 2.509006 -3.772539

64 8 0 -0.236456 1.266715 -0.130715

65 35 0 -3.353213 1.325639 -2.757203

66 35 0 4.260930 2.263345 -0.465938

67 35 0 0.964399 -5.022362 -0.393171

---------------------------------------------------------------------

Frequencies -- 17.8188 21.8801 27.3483

Frequencies -- 34.4978 37.5908 40.1223

Frequencies -- 45.3878 46.0644 52.6393

Frequencies -- 63.1558 65.1510 68.7197

Frequencies -- 76.3722 89.8240 94.5106

Frequencies -- 96.9081 105.4011 107.9586

Frequencies -- 113.3224 115.4394 118.3494

Frequencies -- 120.6463 122.3741 123.7398

Frequencies -- 130.0764 132.1958 132.6683

Frequencies -- 138.4560 140.0014 146.6421

Frequencies -- 153.3959 168.2697 168.5378

Frequencies -- 171.6233 175.5241 179.3420

Frequencies -- 183.4015 195.3933 215.7732

Frequencies -- 257.4591 258.4613 264.0664

Frequencies -- 265.6249 270.5319 273.1697

Frequencies -- 276.0319 277.2285 279.0161

Frequencies -- 280.4296 287.0755 288.7740

Frequencies -- 307.0046 308.2695 318.2418

Frequencies -- 331.7458 334.5464 337.6185

Frequencies -- 397.1225 398.1526 401.3275

Frequencies -- 427.2713 430.3344 431.4511

Frequencies -- 464.0822 466.3035 470.5018

Frequencies -- 479.0551 480.3814 489.3155

Frequencies -- 723.7822 728.1788 729.3385

Frequencies -- 740.3960 741.7647 743.0428

Frequencies -- 768.9207 772.5440 774.4058

Frequencies -- 806.3449 813.1262 813.2139

Frequencies -- 834.3521 836.2593 836.6570

Frequencies -- 838.1145 842.6010 853.6621

Frequencies -- 890.3178 892.3759 893.8159

Frequencies -- 926.4293 927.6363 929.1078

Frequencies -- 1053.2224 1054.7821 1055.6132

Frequencies -- 1072.1658 1072.7917 1075.3549

Frequencies -- 1082.5345 1084.4403 1085.3539

Frequencies -- 1138.1906 1138.8926 1140.4040

Frequencies -- 1146.7511 1147.6968 1149.0712

Frequencies -- 1155.2568 1157.5333 1158.8942

Frequencies -- 1162.5876 1163.2570 1164.6440

Frequencies -- 1167.2841 1168.3849 1179.8707

Frequencies -- 1284.5859 1293.0959 1301.1313

Frequencies -- 1302.2912 1302.5463 1309.2241

Frequencies -- 1338.4701 1343.5410 1349.6142

Frequencies -- 1353.2121 1354.7111 1357.6596

Frequencies -- 1391.5235 1392.5836 1396.1782

Frequencies -- 1403.7793 1405.7844 1408.0297

Frequencies -- 1435.9484 1436.9283 1437.0508

Frequencies -- 1437.8994 1439.0447 1439.7498

Frequencies -- 1441.0465 1441.5887 1445.9727

Frequencies -- 1452.2225 1452.6105 1453.0381

Frequencies -- 1454.4721 1457.7274 1459.6836

Frequencies -- 1467.5602 1468.4660 1474.9778

Frequencies -- 1488.5377 1490.1769 1490.9833

Frequencies -- 1505.5192 1506.1055 1509.2268

Frequencies -- 1542.1614 1543.9798 1545.1253

Frequencies -- 1660.1991 1661.6269 1699.1573

Frequencies -- 3053.3453 3057.9597 3059.9649

Frequencies -- 3060.8731 3061.2394 3061.3170

Frequencies -- 3062.0165 3062.9324 3063.5607

Frequencies -- 3065.1208 3065.1910 3065.5212

Frequencies -- 3117.7920 3121.6749 3125.8032

Frequencies -- 3127.2560 3132.6105 3133.3801

Frequencies -- 3174.8583 3175.1656 3178.3923

Frequencies -- 3181.1736 3181.7446 3182.2294

Frequencies -- 3184.3364 3186.1737 3186.7028

Frequencies -- 3186.9248 3187.6183 3187.6831

SCF Done: E(RM06L-D3/def2-SVP/SMD) = -9655.70906205

Sum of electronic and zero-point Energies= -9655.198443

Sum of electronic and thermal Energies= -9655.153675

Sum of electronic and thermal Free Energies= -9655.281047

SCF Done: E(RM06L-D3/def2-TZVPP/SMD) = -9658.57267756

**P13**

Center Atomic Atomic Coordinates (Angstroms)

Number Number Type X Y Z

---------------------------------------------------------------------

1 83 0 6.345018 6.901521 5.743174

2 8 0 4.955745 6.866045 7.724429

3 8 0 4.545737 8.148166 5.194845

4 8 0 4.966340 5.018508 5.404225

5 8 0 6.449942 6.331063 3.459720

6 8 0 7.320673 8.797747 4.667109

7 8 0 6.789063 8.831607 7.369360

8 6 0 3.602479 6.911331 11.585349

9 1 0 4.076126 6.252836 12.324517

10 6 0 4.340919 6.839573 10.284652

11 1 0 4.379813 5.821737 9.871587

12 6 0 4.113472 7.730152 8.091871

13 6 0 3.558924 8.763229 7.292821

14 6 0 3.794025 8.890431 5.906975

15 6 0 3.491721 10.164195 3.927746

16 1 0 3.331017 9.269627 3.306980

17 6 0 2.649146 11.320982 3.488314

18 1 0 2.895965 11.590408 2.453423

19 6 0 2.998237 1.573057 6.251653

20 1 0 2.592151 1.440331 7.262539

21 6 0 3.646904 2.917142 6.132565

22 1 0 4.479443 3.038811 6.843750

23 6 0 4.844728 4.143028 4.505442

24 6 0 5.398245 4.206237 3.202186

25 6 0 6.170910 5.307661 2.770761

26 6 0 8.205879 5.876174 -0.149047

27 1 0 8.803917 6.702796 -0.554596

28 6 0 7.437167 6.340738 1.049285

29 1 0 6.754712 7.170342 0.802238

30 6 0 7.219115 10.021607 2.392406

31 1 0 6.562705 9.136813 2.398222

32 6 0 6.910154 10.948069 1.257490

33 1 0 7.049944 10.427387 0.301046

34 6 0 7.023729 10.014770 4.742499

35 6 0 6.665535 10.710271 5.925485

36 6 0 6.640519 10.074986 7.188199

37 6 0 6.098063 10.278711 9.498486

38 1 0 6.879890 9.559937 9.792622

39 6 0 5.989983 11.372280 10.516459

40 1 0 5.806987 10.941613 11.511048

41 83 0 7.914499 7.068649 9.031376

42 8 0 9.278516 7.077682 7.025989

43 8 0 9.741107 5.863160 9.581912

44 8 0 9.237838 9.005171 9.217085

45 8 0 7.916496 7.710726 11.299260

46 8 0 6.969585 5.177930 10.159271

47 8 0 7.434249 5.114696 7.440788

48 6 0 10.740685 7.052425 3.197266

49 1 0 10.332969 7.753561 2.458067

50 6 0 9.967096 7.134507 4.477886

51 1 0 8.916023 6.826087 4.325807

52 6 0 10.146408 6.232024 6.674862

53 6 0 10.722139 5.222569 7.489723

54 6 0 10.503765 5.121938 8.880173

55 6 0 10.856643 3.899544 10.884591

56 1 0 9.793604 3.615558 10.935809

57 6 0 11.744597 2.785367 11.344470

58 1 0 11.514676 2.531727 12.387308

59 6 0 11.146844 12.428982 8.174129

60 1 0 11.392310 12.587469 7.116269

61 6 0 10.387971 11.148587 8.337413

62 1 0 10.965048 10.275297 7.997566

63 6 0 9.440699 9.884757 10.096062

64 6 0 9.038477 9.812616 11.452619

65 6 0 8.292956 8.723383 11.958349

66 6 0 6.777259 8.086838 15.181707

67 1 0 6.194436 7.274825 15.635226

68 6 0 7.176485 7.712924 13.787670

69 1 0 6.303285 7.525319 13.143261

70 6 0 7.225390 4.010776 12.453967

71 1 0 6.191007 4.382825 12.535469

72 6 0 7.587868 3.107833 13.591916

73 1 0 7.485715 3.645652 14.543746

74 6 0 7.319498 3.973793 10.098908

75 6 0 7.682749 3.274115 8.920435

76 6 0 7.639510 3.882853 7.644468

77 6 0 8.170772 3.653135 5.337601

78 1 0 9.114915 4.213039 5.434879

79 6 0 8.289127 2.547409 4.334836

80 1 0 8.505858 2.962040 3.340470

81 8 0 7.036091 10.743073 3.621134

82 8 0 6.430182 10.871776 8.239281

83 8 0 3.144169 9.865558 5.291562

84 8 0 3.690076 7.717231 9.358250

85 8 0 6.653927 5.236678 1.530717

86 8 0 4.151812 3.043338 4.792892

87 8 0 7.848243 3.071840 6.604677

88 8 0 7.366793 3.266901 11.233331

89 8 0 10.583387 6.241923 5.413990

90 8 0 11.186059 4.180725 9.512026

91 8 0 10.074489 10.997819 9.732844

92 8 0 7.944381 8.797247 13.242434

93 1 0 9.941378 8.151128 4.896228

94 1 0 10.987352 4.813020 11.484686

95 1 0 11.597190 1.883820 10.735171

96 1 0 12.805227 3.065632 11.292985

97 1 0 10.704509 6.041851 2.766960

98 1 0 11.795260 7.313704 3.356800

99 1 0 7.383975 4.364735 5.037855

100 1 0 9.101971 1.859221 4.603866

101 1 0 7.355440 1.972721 4.261583

102 1 0 7.875247 4.899030 12.402561

103 1 0 6.936066 2.224971 13.632853

104 1 0 8.626994 2.758951 13.512804

105 1 0 8.096556 6.700133 1.853176

106 1 0 7.538395 5.520601 -0.945304

107 1 0 8.891478 5.057911 0.110260

108 1 0 2.942658 3.740200 6.330098

109 1 0 3.717823 0.763963 6.068118

110 1 0 2.170667 1.460174 5.538711

111 1 0 9.447278 11.149665 7.762798

112 1 0 12.087393 12.414118 8.740837

113 1 0 10.558703 13.291492 8.515137

114 1 0 7.789650 6.798165 13.770163

115 1 0 6.157903 8.993666 15.193207

116 1 0 7.654921 8.266382 15.816630

117 1 0 8.254427 9.646194 2.347782

118 1 0 5.870389 11.301092 1.297437

119 1 0 7.566692 11.828399 1.260222

120 1 0 5.149875 9.726793 9.393140

121 1 0 6.918778 11.956335 10.573244

122 1 0 5.163451 12.055295 10.278404

123 1 0 5.388200 7.171528 10.412637

124 1 0 2.559464 6.588103 11.468752

125 1 0 3.598062 7.932580 11.990523

126 1 0 4.565309 10.406914 3.887088

127 1 0 1.578573 11.079135 3.526702

128 1 0 2.825772 12.201477 4.120138

129 35 0 6.195440 12.545515 5.806024

130 35 0 9.472369 11.252844 12.610977

131 35 0 11.860613 3.934037 6.677103

132 35 0 8.262234 1.471138 9.063852

133 35 0 5.126842 2.748120 2.016738

134 35 0 2.456267 10.069611 8.125598

---------------------------------------------------------------------

Frequencies -- 15.4278 16.2736 20.2897

Frequencies -- 25.6940 30.9299 33.1549

Frequencies -- 34.6792 35.7416 38.3162

Frequencies -- 40.4229 41.8230 43.2765

Frequencies -- 45.6349 48.3362 51.9837

Frequencies -- 54.5208 55.8136 57.1703

Frequencies -- 65.1893 68.2059 70.8083

Frequencies -- 73.4776 78.8916 79.9525

Frequencies -- 82.1483 83.4764 88.2747

Frequencies -- 90.1338 92.0209 93.3757

Frequencies -- 95.6312 96.3677 98.9899

Frequencies -- 99.3441 102.0972 102.6784

Frequencies -- 104.7218 107.1650 109.6535

Frequencies -- 110.2753 112.5942 115.1856

Frequencies -- 115.7852 118.9716 120.4083

Frequencies -- 121.7598 123.8351 125.9681

Frequencies -- 128.7855 130.2733 132.7662

Frequencies -- 135.1853 136.1949 139.6270

Frequencies -- 140.8632 142.2224 143.3715

Frequencies -- 145.1501 147.9427 149.0541

Frequencies -- 151.6838 156.4345 159.7570

Frequencies -- 161.0024 162.6462 165.0027

Frequencies -- 166.7324 169.1236 173.9470

Frequencies -- 178.5087 180.3177 181.3018

Frequencies -- 182.9492 186.6228 188.5966

Frequencies -- 193.2417 196.8557 199.6651

Frequencies -- 204.4310 210.1521 215.9098

Frequencies -- 220.7141 241.8073 247.3419

Frequencies -- 249.5553 251.6474 256.1164

Frequencies -- 257.0528 258.4117 262.1793

Frequencies -- 264.0618 265.9862 270.0767

Frequencies -- 271.7637 272.8944 274.4082

Frequencies -- 277.4647 279.3222 282.9313

Frequencies -- 284.3705 287.8206 292.5132

Frequencies -- 293.6273 297.0450 301.4021

Frequencies -- 302.7808 303.3836 304.9527

Frequencies -- 308.8928 310.1250 312.2105

Frequencies -- 312.5439 315.3351 333.2736

Frequencies -- 334.5464 337.7914 339.9577

Frequencies -- 340.8618 343.2416 344.1787

Frequencies -- 393.8903 399.9263 401.5809

Frequencies -- 404.2257 407.6513 411.7991

Frequencies -- 412.3800 415.8022 420.5757

Frequencies -- 422.3168 427.1723 432.8800

Frequencies -- 462.1266 463.4337 466.7826

Frequencies -- 468.0107 469.7317 470.7248

Frequencies -- 474.6983 476.5195 477.6418

Frequencies -- 481.2141 485.1301 487.8141

Frequencies -- 721.4073 721.6838 726.0934

Frequencies -- 726.3663 730.1433 731.6851

Frequencies -- 735.2899 736.1682 739.7113

Frequencies -- 739.9011 744.5549 744.8193

Frequencies -- 757.6736 759.4452 767.5644

Frequencies -- 767.7457 769.8667 770.9003

Frequencies -- 811.0739 813.1203 813.5423

Frequencies -- 820.7768 822.4271 823.4749

Frequencies -- 823.7973 826.2522 830.3874

Frequencies -- 832.2635 832.3665 835.4098

Frequencies -- 836.8021 837.1020 839.3199

Frequencies -- 840.1360 847.6507 851.7253

Frequencies -- 889.9543 890.8672 891.1483

Frequencies -- 891.7925 892.3960 893.2884

Frequencies -- 923.8575 924.7295 928.7148

Frequencies -- 930.4355 931.0585 931.7561

Frequencies -- 1050.6303 1051.3175 1052.1671

Frequencies -- 1055.1210 1056.9750 1057.4595

Frequencies -- 1067.8606 1068.4698 1070.7987

Frequencies -- 1072.8672 1076.3863 1076.6998

Frequencies -- 1077.0938 1078.3163 1083.0171

Frequencies -- 1083.8588 1084.2449 1085.3070

Frequencies -- 1137.7321 1138.0119 1140.9594

Frequencies -- 1141.4183 1141.9313 1142.8293

Frequencies -- 1145.8283 1146.7132 1147.8322

Frequencies -- 1149.0214 1149.5204 1150.2542

Frequencies -- 1150.9481 1151.4756 1155.0930

Frequencies -- 1157.1336 1157.5722 1159.4402

Frequencies -- 1162.3731 1164.7738 1165.3332

Frequencies -- 1166.9126 1167.4726 1167.6510

Frequencies -- 1168.3283 1168.8034 1171.1019

Frequencies -- 1172.1241 1172.5376 1175.6908

Frequencies -- 1287.3330 1289.3629 1294.0591

Frequencies -- 1299.1127 1301.7811 1302.0666

Frequencies -- 1304.6934 1307.4423 1308.2260

Frequencies -- 1309.5337 1314.5565 1317.4321

Frequencies -- 1336.8400 1342.0699 1344.8701

Frequencies -- 1347.7616 1350.0642 1351.1531

Frequencies -- 1354.8494 1357.3414 1359.5784

Frequencies -- 1361.3689 1362.7198 1363.5146

Frequencies -- 1390.4158 1391.7376 1393.4323

Frequencies -- 1394.1229 1394.7321 1398.0210

Frequencies -- 1403.4732 1403.8094 1404.5250

Frequencies -- 1404.8321 1406.0156 1406.8580

Frequencies -- 1432.0765 1432.9625 1434.7924

Frequencies -- 1435.4735 1437.1355 1437.4567

Frequencies -- 1437.9707 1438.9445 1439.9055

Frequencies -- 1440.0250 1440.5820 1441.4915

Frequencies -- 1442.2474 1442.7290 1443.7835

Frequencies -- 1444.3197 1445.8558 1446.2601

Frequencies -- 1448.2116 1449.5100 1449.9594

Frequencies -- 1451.7598 1452.9809 1453.9802

Frequencies -- 1455.5115 1456.4551 1457.7497

Frequencies -- 1461.0218 1462.1751 1462.3889

Frequencies -- 1463.0607 1463.9390 1465.6310

Frequencies -- 1467.5221 1469.1146 1471.6964

Frequencies -- 1480.4685 1481.6817 1483.2193

Frequencies -- 1487.9603 1492.1038 1494.4593

Frequencies -- 1500.4664 1501.1581 1502.7042

Frequencies -- 1503.6541 1510.9181 1512.0801

Frequencies -- 1522.0025 1523.0835 1534.9262

Frequencies -- 1535.9290 1540.8817 1551.4107

Frequencies -- 1643.5160 1643.6739 1655.3793

Frequencies -- 1658.6071 1681.7019 1707.0057

Frequencies -- 3035.7995 3043.5146 3054.1708

Frequencies -- 3054.6266 3056.2403 3056.6779

Frequencies -- 3058.3793 3058.7372 3060.4978

Frequencies -- 3061.9315 3062.1835 3062.7837

Frequencies -- 3063.1465 3063.4682 3064.4368

Frequencies -- 3064.9708 3065.7750 3066.9770

Frequencies -- 3070.4629 3072.5358 3073.6806

Frequencies -- 3074.1727 3080.0352 3082.9469

Frequencies -- 3119.2453 3124.4917 3134.1359

Frequencies -- 3135.1442 3135.4240 3137.1529

Frequencies -- 3138.1032 3139.4551 3140.4591

Frequencies -- 3145.8446 3150.9297 3151.5671

Frequencies -- 3170.5718 3173.8511 3174.4705

Frequencies -- 3175.1942 3177.4080 3178.3288

Frequencies -- 3178.8718 3178.9120 3179.2342

Frequencies -- 3180.4641 3181.2872 3181.4208

Frequencies -- 3181.6875 3182.2199 3182.8314

Frequencies -- 3183.8253 3183.8991 3184.9191

Frequencies -- 3185.1953 3186.8321 3187.0991

Frequencies -- 3188.8720 3189.3638 3190.9482

SCF Done: E(RM06L-D3/def2-SVP/SMD) = -19311.4650869

Sum of electronic and zero-point Energies= -19310.440552

Sum of electronic and thermal Energies= -19310.350858

Sum of electronic and thermal Free Energies= -19310.572980

SCF Done: E(RM06L-D3/def2-TZVPP/SMD) = -19317.1870145

**P14**

Center Atomic Atomic Coordinates (Angstroms)

Number Number Type X Y Z

---------------------------------------------------------------------

1 83 0 7.798630 0.301016 2.781620

2 8 0 8.694692 1.133371 1.075074

3 83 0 9.298387 -0.638402 0.016270

4 8 0 8.304048 -1.474378 1.679550

5 8 0 6.610205 2.402995 2.593149

6 6 0 5.801990 2.710875 1.685852

7 6 0 4.828777 1.843961 1.122568

8 8 0 5.811879 3.958770 1.203537

9 6 0 4.792971 0.471150 1.437189

10 6 0 6.826904 4.833408 1.710756

11 8 0 5.663634 -0.168266 2.108311

12 8 0 3.753050 -0.221835 0.982598

13 1 0 6.666825 4.987524 2.789888

14 1 0 7.807462 4.342357 1.604658

15 6 0 6.753951 6.119352 0.945814

16 6 0 3.751816 -1.637390 1.217609

17 1 0 6.948105 5.961508 -0.123344

18 1 0 5.767513 6.592611 1.044352

19 1 0 7.504014 6.826838 1.322276

20 1 0 4.708653 -2.061012 0.874133

21 1 0 3.701061 -1.816052 2.303771

22 6 0 2.578344 -2.227444 0.497035

23 1 0 1.641600 -1.735339 0.792413

24 1 0 2.692717 -2.131535 -0.591075

25 1 0 2.482864 -3.293416 0.741186

26 8 0 7.841633 -2.107386 -1.137205

27 6 0 6.653102 -1.891935 -1.498206

28 6 0 6.134313 -0.647371 -1.923276

29 8 0 5.793914 -2.917451 -1.486688

30 6 0 6.948931 0.508555 -1.965466

31 6 0 6.271609 -4.142533 -0.912560

32 8 0 8.172591 0.549101 -1.658675

33 8 0 6.367813 1.632635 -2.376951

34 1 0 7.140290 -4.499469 -1.487319

35 1 0 6.635061 -3.938650 0.107933

36 6 0 5.148213 -5.132932 -0.930976

37 6 0 7.122087 2.843541 -2.206049

38 1 0 4.289635 -4.781008 -0.343715

39 1 0 4.800703 -5.326953 -1.954771

40 1 0 5.480710 -6.086925 -0.501656

41 1 0 7.489499 2.887794 -1.167063

42 1 0 8.012627 2.805548 -2.853963

43 6 0 6.230061 3.997487 -2.546913

44 1 0 5.825056 3.900435 -3.563781

45 1 0 5.387789 4.072854 -1.845456

46 1 0 6.800288 4.935012 -2.506886

47 35 0 3.458928 2.578651 0.031728

48 35 0 4.334121 -0.526943 -2.506016

---------------------------------------------------------------------

Frequencies -- 11.8221 19.3038 31.7897

Frequencies -- 39.7095 43.9838 54.9684

Frequencies -- 56.6124 82.1058 83.4189

Frequencies -- 87.4717 94.0428 96.8687

Frequencies -- 106.8395 110.9793 122.7210

Frequencies -- 128.4430 131.9673 135.2994

Frequencies -- 139.4808 147.6971 154.2687

Frequencies -- 168.5370 171.4707 176.6837

Frequencies -- 184.9457 190.7425 198.8127

Frequencies -- 210.5678 225.3105 238.6193

Frequencies -- 258.2700 262.2628 265.6227

Frequencies -- 270.7810 272.5873 282.0295

Frequencies -- 289.0181 297.8002 307.6691

Frequencies -- 329.2450 331.9801 337.9972

Frequencies -- 392.6977 398.9176 425.4193

Frequencies -- 426.5261 460.8300 466.3496

Frequencies -- 467.3408 471.0592 474.8330

Frequencies -- 479.5504 550.8944 573.6181

Frequencies -- 721.9010 728.5902 736.7971

Frequencies -- 738.8139 765.8641 769.9108

Frequencies -- 815.3903 819.3279 820.4536

Frequencies -- 834.0809 834.5741 839.9601

Frequencies -- 886.4870 893.9487 925.5734

Frequencies -- 927.7540 1054.3917 1058.3311

Frequencies -- 1075.6619 1078.2703 1082.7228

Frequencies -- 1085.3856 1136.3070 1137.2440

Frequencies -- 1145.3642 1148.2519 1152.5194

Frequencies -- 1156.4495 1165.8883 1167.5853

Frequencies -- 1170.0738 1171.7348 1290.8077

Frequencies -- 1300.4884 1307.3034 1318.5517

Frequencies -- 1347.9719 1353.1266 1361.0757

Frequencies -- 1362.0704 1389.8434 1395.9362

Frequencies -- 1402.6429 1406.1197 1435.2135

Frequencies -- 1438.0453 1439.6012 1442.9822

Frequencies -- 1443.4154 1444.9035 1448.3656

Frequencies -- 1452.8933 1458.0278 1463.9843

Frequencies -- 1473.5150 1483.5485 1495.6948

Frequencies -- 1502.2794 1509.3585 1515.7306

Frequencies -- 1536.6928 1542.9744 1666.7431

Frequencies -- 1686.1445 3052.5979 3058.9689

Frequencies -- 3059.9837 3061.2038 3061.5635

Frequencies -- 3063.0088 3063.3316 3065.9432

Frequencies -- 3123.6234 3124.9129 3130.1988

Frequencies -- 3132.0421 3170.2098 3176.5723

Frequencies -- 3176.8759 3178.1947 3178.7489

Frequencies -- 3182.6153 3183.9223 3184.1885

SCF Done: E(RM06L-D3/def2-SVP/SMD) = -6873.83281529

Sum of electronic and zero-point Energies= -6873.485784

Sum of electronic and thermal Energies= -6873.451905

Sum of electronic and thermal Free Energies= -6873.555457

SCF Done: E(RM06L-D3/def2-TZVPP/SMD) = -6875.95091588

**P15**

Center Atomic Atomic Coordinates (Angstroms)

Number Number Type X Y Z

---------------------------------------------------------------------

1 83 0 6.216116 8.299812 6.611211

2 8 0 5.137093 7.032973 8.256196

3 8 0 3.905512 8.706132 6.485125

4 8 0 5.960684 6.015831 5.841525

5 8 0 5.945245 8.169326 4.033982

6 8 0 6.816507 9.286883 8.448334

7 6 0 5.131462 3.253639 9.744921

8 1 0 6.043904 2.910983 10.251685

9 6 0 5.265618 4.700533 9.382697

10 1 0 6.141180 4.890947 8.742269

11 6 0 4.143806 6.266138 8.028278

12 6 0 3.086308 6.568046 7.150403

13 6 0 3.029743 7.795243 6.442333

14 6 0 1.913815 9.178231 4.885220

15 1 0 2.831733 9.234938 4.279968

16 6 0 0.679933 9.123737 4.039605

17 1 0 0.609981 10.029038 3.423238

18 6 0 6.392840 1.972110 6.007588

19 1 0 6.445220 1.488926 6.992275

20 6 0 6.108277 3.435693 6.161965

21 1 0 6.869933 3.925987 6.789836

22 6 0 6.057070 5.355394 4.768277

23 6 0 6.127395 5.873982 3.455733

24 6 0 6.044410 7.266931 3.177821

25 6 0 6.219343 9.127524 0.076812

26 1 0 6.182354 10.186155 -0.211083

27 6 0 6.040532 8.985669 1.558071

28 1 0 5.077995 9.402678 1.896733

29 83 0 7.248568 7.974380 9.987639

30 8 0 7.915172 6.870471 7.790178

31 8 0 8.051707 5.609210 10.290960

32 8 0 9.508591 8.067983 9.781227

33 8 0 7.966932 9.808184 11.278718

34 6 0 9.930310 6.714957 4.168551

35 1 0 9.904282 7.578961 3.491903

36 6 0 9.022131 6.952663 5.336971

37 1 0 7.993302 7.108874 4.972270

38 6 0 8.616387 5.866276 7.434527

39 6 0 9.005546 4.802877 8.265628

40 6 0 8.732943 4.774707 9.666463

41 6 0 9.077533 3.703881 11.743319

42 1 0 7.997683 3.703707 11.961182

43 6 0 9.757358 2.476389 12.267531

44 1 0 9.638860 2.415586 13.356831

45 6 0 12.861205 7.688217 7.555660

46 1 0 13.044681 6.712105 7.087295

47 6 0 11.617216 7.618775 8.384545

48 1 0 11.686351 6.857746 9.177925

49 6 0 10.270186 9.078390 9.665258

50 6 0 10.035637 10.351953 10.219593

51 6 0 8.914376 10.609898 11.050509

52 6 0 7.959302 13.475988 13.039387

53 1 0 7.115090 13.740204 13.688823

54 6 0 7.759780 12.103187 12.474865

55 1 0 6.839727 12.033515 11.874180

56 8 0 1.956598 7.982552 5.683224

57 8 0 4.085810 5.104102 8.668657

58 8 0 6.092571 7.592905 1.875442

59 8 0 6.117143 4.021739 4.857839

60 8 0 9.063413 5.796873 6.174818

61 8 0 9.275697 3.744879 10.323581

62 8 0 11.400720 8.908710 8.982898

63 8 0 8.889305 11.801222 11.640421

64 1 0 9.326027 7.841267 5.918612

65 1 0 9.485533 4.625085 12.188907

66 1 0 9.331058 1.563126 11.830956

67 1 0 10.832890 2.488122 12.046060

68 1 0 9.613191 5.830749 3.599388

69 1 0 10.968885 6.565351 4.493163

70 1 0 6.823691 9.518984 2.120362

71 1 0 5.429848 8.600082 -0.475317

72 1 0 7.187025 8.723464 -0.250146

73 1 0 5.127450 3.622317 6.632638

74 1 0 7.358357 1.812836 5.509638

75 1 0 5.616003 1.465178 5.419156

76 1 0 10.738701 7.353476 7.776931

77 1 0 13.740156 7.943511 8.162268

78 1 0 12.769490 8.435360 6.755659

79 1 0 7.681342 11.338401 13.262653

80 1 0 8.023371 14.232244 12.245782

81 1 0 8.876919 13.534489 13.639779

82 1 0 5.375151 5.330425 10.281110

83 1 0 4.984350 2.625940 8.855710

84 1 0 4.285433 3.079058 10.422778

85 1 0 1.919683 10.053440 5.552976

86 1 0 0.691896 8.257195 3.365253

87 1 0 -0.228042 9.064470 4.654412

88 35 0 10.092302 3.434385 7.516065

89 35 0 11.289217 11.742646 9.911182

90 35 0 6.321357 4.652781 2.007570

91 35 0 1.736582 5.271047 6.854477

---------------------------------------------------------------------

Frequencies -- -0.8661 13.8109 17.8637

Frequencies -- 19.9906 22.2479 25.9716

Frequencies -- 29.6687 31.1869 38.4652

Frequencies -- 45.9177 47.6153 49.5826

Frequencies -- 55.9966 67.6860 70.7711

Frequencies -- 71.6542 74.3010 75.4383

Frequencies -- 79.9216 84.7644 87.6225

Frequencies -- 91.2412 92.6813 95.2698

Frequencies -- 96.9496 97.3827 101.9983

Frequencies -- 107.7836 108.2158 110.9486

Frequencies -- 113.2009 116.4265 122.1968

Frequencies -- 125.0315 127.5333 129.4186

Frequencies -- 132.2791 133.1793 136.0877

Frequencies -- 136.3749 139.6862 142.8806

Frequencies -- 145.0045 149.7205 152.7157

Frequencies -- 156.4400 164.8056 169.3379

Frequencies -- 177.4652 179.9461 185.3511

Frequencies -- 186.9720 202.2305 212.7629

Frequencies -- 228.3284 230.4419 232.7668

Frequencies -- 253.7900 254.1171 257.4593

Frequencies -- 259.6977 261.4361 264.8702

Frequencies -- 265.5535 268.6583 271.6948

Frequencies -- 277.3497 285.2874 285.8309

Frequencies -- 286.8482 294.3944 297.8280

Frequencies -- 299.3561 300.7260 314.2281

Frequencies -- 322.0565 329.8425 330.1937

Frequencies -- 333.6080 339.0000 343.1531

Frequencies -- 389.1926 390.1420 401.0661

Frequencies -- 403.1101 407.5843 420.7763

Frequencies -- 428.2029 434.3976 447.9427

Frequencies -- 456.1473 461.7262 466.7332

Frequencies -- 472.0784 473.0734 479.5334

Frequencies -- 482.6252 490.4931 587.0603

Frequencies -- 720.9196 721.9463 725.6476

Frequencies -- 731.0560 737.9161 738.4184

Frequencies -- 739.0646 744.0429 766.9910

Frequencies -- 769.1820 772.9852 773.4639

Frequencies -- 807.3302 814.1050 814.1535

Frequencies -- 819.7981 822.2003 822.6831

Frequencies -- 827.7789 831.4448 832.1594

Frequencies -- 835.6745 839.8709 842.2566

Frequencies -- 888.2609 890.8705 893.3639

Frequencies -- 895.3776 928.6870 928.7763

Frequencies -- 930.3093 933.5693 1051.5301

Frequencies -- 1052.7911 1054.4982 1056.6990

Frequencies -- 1068.0787 1069.7376 1072.2032

Frequencies -- 1074.8059 1081.2904 1082.6938

Frequencies -- 1084.5613 1085.8601 1137.8687

Frequencies -- 1140.0776 1140.5918 1141.5122

Frequencies -- 1144.5474 1146.1377 1146.6624

Frequencies -- 1148.6484 1150.1312 1152.7794

Frequencies -- 1157.2919 1158.1705 1164.5655

Frequencies -- 1165.9200 1166.1037 1166.5677

Frequencies -- 1169.3505 1170.3765 1173.7836

Frequencies -- 1173.9866 1284.6287 1285.6339

Frequencies -- 1292.5986 1295.3336 1296.1212

Frequencies -- 1296.5997 1298.9536 1318.4655

Frequencies -- 1331.2328 1334.4805 1342.9987

Frequencies -- 1344.7963 1345.8363 1348.6188

Frequencies -- 1350.6576 1366.9420 1385.8233

Frequencies -- 1387.0388 1398.3635 1399.8532

Frequencies -- 1400.9996 1402.3913 1407.8898

Frequencies -- 1413.9585 1427.6826 1429.6250

Frequencies -- 1431.5495 1434.1437 1438.0675

Frequencies -- 1438.5631 1438.8323 1439.9845

Frequencies -- 1440.3752 1441.0839 1444.5337

Frequencies -- 1446.4433 1451.4923 1452.1033

Frequencies -- 1453.8858 1454.3335 1454.9894

Frequencies -- 1457.5508 1457.9479 1461.2385

Frequencies -- 1463.2644 1464.6638 1467.1114

Frequencies -- 1467.5053 1484.9621 1487.1255

Frequencies -- 1488.3190 1492.3045 1497.6806

Frequencies -- 1502.2522 1505.4327 1506.1061

Frequencies -- 1524.4825 1534.7283 1546.7377

Frequencies -- 1561.9702 1643.2645 1658.6926

Frequencies -- 1687.5396 1699.9742 3030.2068

Frequencies -- 3050.1532 3053.4951 3056.0517

Frequencies -- 3056.6576 3057.8301 3059.8046

Frequencies -- 3059.9137 3061.2240 3061.8360

Frequencies -- 3063.1627 3063.7521 3066.0852

Frequencies -- 3067.6713 3067.6998 3069.6681

Frequencies -- 3114.8615 3116.5758 3120.6250

Frequencies -- 3129.9411 3130.4195 3130.7548

Frequencies -- 3131.5383 3134.6749 3174.1537

Frequencies -- 3174.4424 3177.0708 3177.4864

Frequencies -- 3178.4355 3178.6972 3179.3978

Frequencies -- 3180.7944 3180.8349 3181.7898

Frequencies -- 3182.1452 3184.0178 3185.3168

Frequencies -- 3185.4017 3186.0873 3187.0088

SCF Done: E(RM06L-D3/def2-SVP/SMD) = -13092.6325059

Sum of electronic and zero-point Energies= -13091.948652

Sum of electronic and thermal Energies= -13091.886799

Sum of electronic and thermal Free Energies= -13092.052320

SCF Done: E(RM06L-D3/def2-TZVPP/SMD) = -13096.5511749

**P16 (Tetraethyl 1,1,2,2-ethanetetracarboxylate)**

Center Atomic Atomic Coordinates (Angstroms)

Number Number Type X Y Z

---------------------------------------------------------------------

1 8 0 0.688014 2.314924 1.792535

2 8 0 0.521934 1.754252 -1.071785

3 6 0 1.192696 3.844371 -0.029072

4 6 0 0.618119 2.952222 -1.117384

5 6 0 0.749584 3.437958 1.366561

6 8 0 0.482505 4.537046 2.084085

7 8 0 0.279820 3.700309 -2.175847

8 6 0 0.135644 4.317846 3.464746

9 1 0 -0.731088 3.640468 3.505263

10 1 0 0.968025 3.787409 3.954417

11 6 0 -0.192110 2.978910 -3.330220

12 1 0 0.595594 2.278155 -3.650291

13 1 0 -1.053368 2.362358 -3.030849

14 6 0 -0.150432 5.645557 4.093980

15 1 0 0.718726 6.315196 4.042980

16 1 0 -0.994354 6.149333 3.604021

17 1 0 -0.407387 5.511958 5.152474

18 6 0 -0.545908 3.969774 -4.394608

19 1 0 -1.344109 4.647836 -4.064152

20 1 0 0.318603 4.581928 -4.684497

21 1 0 -0.900878 3.445673 -5.291063

22 1 0 0.890870 4.884505 -0.215593

23 6 0 2.744216 3.790419 -0.099354

24 6 0 3.187428 4.196666 -1.495009

25 6 0 3.318726 4.682699 0.988870

26 1 0 3.046054 2.750312 0.087284

27 8 0 3.249245 5.319659 -1.921051

28 8 0 3.454363 3.097474 -2.212432

29 8 0 3.414693 5.880686 0.943174

30 8 0 3.657205 3.934724 2.047344

31 6 0 3.801234 3.316541 -3.593111

32 6 0 4.128949 4.656264 3.201702

33 1 0 2.969048 3.847334 -4.082729

34 1 0 4.668257 3.993543 -3.633667

35 6 0 4.086717 1.988705 -4.222358

36 1 0 4.990175 5.272882 2.902352

37 1 0 3.341146 5.356957 3.521644

38 6 0 4.482846 3.665503 4.266158

39 1 0 4.930316 1.484490 -3.732295

40 1 0 3.217214 1.319502 -4.171477

41 1 0 4.343878 2.122191 -5.280813

42 1 0 3.618531 3.052906 4.555700

43 1 0 5.281486 2.987845 3.935938

44 1 0 4.837236 4.189721 5.162774

---------------------------------------------------------------------

Frequencies -- 20.8833 45.8210 55.3068

Frequencies -- 61.1802 70.0376 81.0641

Frequencies -- 85.8519 94.4246 114.0059

Frequencies -- 122.7145 125.7657 143.4891

Frequencies -- 147.3547 163.9008 174.3372

Frequencies -- 189.2072 192.0044 197.8989

Frequencies -- 202.8064 216.7079 219.1670

Frequencies -- 259.2044 261.6230 292.5310

Frequencies -- 297.7948 309.0226 316.0429

Frequencies -- 324.1723 353.8868 355.9136

Frequencies -- 373.9740 407.1025 422.8561

Frequencies -- 425.5468 444.8586 672.8900

Frequencies -- 701.8669 732.7814 754.4957

Frequencies -- 754.9132 804.8458 804.9175

Frequencies -- 824.2150 825.3710 828.3983

Frequencies -- 838.3702 844.2317 881.8729

Frequencies -- 884.2179 904.0079 907.4538

Frequencies -- 973.0495 973.1338 1014.4303

Frequencies -- 1045.0286 1053.6548 1061.9624

Frequencies -- 1075.2865 1076.7506 1088.6038

Frequencies -- 1142.3675 1143.0184 1146.2563

Frequencies -- 1147.1785 1165.5467 1167.8631

Frequencies -- 1168.4711 1169.0128 1171.4818

Frequencies -- 1172.4202 1181.1457 1272.5588

Frequencies -- 1285.9214 1299.3010 1299.5779

Frequencies -- 1302.8232 1303.4353 1344.1979

Frequencies -- 1356.1577 1362.2837 1373.4262

Frequencies -- 1377.5726 1389.1418 1395.9952

Frequencies -- 1419.8680 1420.7188 1424.1255

Frequencies -- 1425.4406 1438.8329 1438.8690

Frequencies -- 1441.7159 1441.7315 1453.6680

Frequencies -- 1453.7429 1457.3117 1457.5851

Frequencies -- 1500.9889 1501.5110 1509.3349

Frequencies -- 1509.7255 1846.6070 1855.7443

Frequencies -- 1885.2332 1885.3368 3049.4609

Frequencies -- 3049.5232 3061.0793 3061.1161

Frequencies -- 3061.9463 3061.9606 3063.1579

Frequencies -- 3063.3713 3119.3301 3119.4182

Frequencies -- 3124.1694 3124.6223 3124.6555

Frequencies -- 3137.5153 3172.8400 3172.8786

Frequencies -- 3174.8938 3174.9042 3187.5359

Frequencies -- 3187.5505 3188.8903 3188.9225

SCF Done: E(RM06L-D3/def2-SVP/SMD) = -1147.62550754

Sum of electronic and zero-point Energies= -1147.262208

Sum of electronic and thermal Energies= -1147.237815

Sum of electronic and thermal Free Energies= -1147.316740

SCF Done: E(RM06L-D3/def2-TZVPP/SMD) = -1148.90699991

**P17 (Tetraethyl 1,1,2,2-ethenetetracarboxylate)**

Center Atomic Atomic Coordinates (Angstroms)

Number Number Type X Y Z

---------------------------------------------------------------------

1 8 0 0.720582 2.116381 1.922176

2 8 0 0.410911 1.557989 -1.046680

3 6 0 1.429413 3.427256 0.047547

4 6 0 0.718860 2.722249 -1.062218

5 6 0 0.908917 3.199701 1.431891

6 8 0 0.661033 4.369577 2.030429

7 8 0 0.456221 3.573229 -2.059949

8 6 0 0.209374 4.299788 3.396541

9 1 0 -0.651878 3.616522 3.446061

10 1 0 1.005943 3.842831 4.005509

11 6 0 -0.149406 3.004902 -3.236811

12 1 0 0.539717 2.251800 -3.652272

13 1 0 -1.060400 2.464835 -2.937625

14 6 0 -0.133620 5.685418 3.845507

15 1 0 0.733186 6.357707 3.788698

16 1 0 -0.937300 6.115170 3.233057

17 1 0 -0.477122 5.668592 4.887504

18 6 0 -0.434799 4.116287 -4.197770

19 1 0 -1.136192 4.845081 -3.770470

20 1 0 0.480000 4.654239 -4.481727

21 1 0 -0.885656 3.713670 -5.113679

22 6 0 2.507991 4.205731 -0.183805

23 6 0 3.028208 4.433105 -1.568257

24 6 0 3.218506 4.911012 0.925822

25 8 0 3.216857 5.516307 -2.058688

26 8 0 3.275517 3.263080 -2.166798

27 8 0 3.526908 6.075145 0.909767

28 8 0 3.480650 4.060440 1.924024

29 6 0 3.726649 3.332583 -3.533081

30 6 0 4.085782 4.629248 3.100904

31 1 0 2.929820 3.789232 -4.141938

32 1 0 4.587758 4.015999 -3.583063

33 6 0 4.069754 1.946885 -3.981795

34 1 0 4.996888 5.169218 2.801907

35 1 0 3.396451 5.382493 3.515779

36 6 0 4.370717 3.518260 4.062466

37 1 0 4.873798 1.517486 -3.369584

38 1 0 3.203095 1.274456 -3.924380

39 1 0 4.412754 1.963479 -5.023960

40 1 0 3.455834 2.980003 4.345589

41 1 0 5.072874 2.789627 3.636148

42 1 0 4.820373 3.921345 4.978757

---------------------------------------------------------------------

Frequencies -- 23.8962 34.9380 43.7302

Frequencies -- 62.4711 72.0308 73.3781

Frequencies -- 81.7285 82.3265 105.7185

Frequencies -- 108.4123 125.9391 135.6336

Frequencies -- 153.9833 161.6144 179.7179

Frequencies -- 188.4343 194.8310 206.9684

Frequencies -- 210.0001 214.0692 239.0658

Frequencies -- 252.7824 283.6096 286.2047

Frequencies -- 286.6094 308.0761 309.7773

Frequencies -- 341.1097 352.9711 355.2340

Frequencies -- 376.4294 395.2125 415.5161

Frequencies -- 439.2428 467.9378 660.7204

Frequencies -- 677.5191 705.5467 737.0471

Frequencies -- 753.1742 802.2554 803.7510

Frequencies -- 810.0572 812.5219 827.1883

Frequencies -- 841.3584 854.9060 878.4400

Frequencies -- 883.8645 886.1771 905.0034

Frequencies -- 924.6733 1000.3770 1039.5303

Frequencies -- 1048.5741 1060.2382 1067.9796

Frequencies -- 1096.7980 1125.4976 1143.3125

Frequencies -- 1145.9399 1146.6198 1151.6350

Frequencies -- 1164.9803 1165.1490 1169.3983

Frequencies -- 1169.6007 1248.5342 1282.2362

Frequencies -- 1284.4649 1293.2519 1306.1729

Frequencies -- 1308.1750 1308.3107 1344.4790

Frequencies -- 1371.2490 1375.4801 1378.7354

Frequencies -- 1383.9373 1416.2636 1417.0794

Frequencies -- 1424.5952 1425.7031 1434.3924

Frequencies -- 1434.4220 1437.9776 1438.0438

Frequencies -- 1451.9159 1451.9790 1458.5648

Frequencies -- 1458.7646 1490.0335 1490.4695

Frequencies -- 1505.6851 1506.0864 1688.0514

Frequencies -- 1833.7831 1838.8171 1874.6585

Frequencies -- 1875.3395 3056.0799 3056.0989

Frequencies -- 3062.3452 3062.4237 3064.5959

Frequencies -- 3064.6657 3070.7792 3070.9802

Frequencies -- 3126.6373 3126.7020 3133.7140

Frequencies -- 3133.8378 3176.7998 3176.9002

Frequencies -- 3179.2226 3179.2924 3185.6670

Frequencies -- 3185.7339 3190.3100 3190.3424

SCF Done: E(RM06L-D3/def2-SVP/SMD) = -1146.39712859

Sum of electronic and zero-point Energies= -1146.058228

Sum of electronic and thermal Energies= -1146.033839

Sum of electronic and thermal Free Energies= -1146.113221

SCF Done: E(RM06L-D3/def2-TZVPP/SMD) = -1147.6775383

**P18 (Diethyl malonate)**

Center Atomic Atomic Coordinates (Angstroms)

Number Number Type X Y Z

---------------------------------------------------------------------

1 8 0 1.889133 2.507586 1.876829

2 8 0 -0.440892 2.655156 -0.729307

3 6 0 1.491768 3.918529 -0.038282

4 6 0 0.359281 3.530823 -0.953518

5 6 0 1.275915 3.407529 1.358778

6 8 0 0.297849 4.094879 1.960448

7 8 0 0.376608 4.269514 -2.065946

8 6 0 -0.046351 3.670656 3.289712

9 1 0 -0.293164 2.597972 3.265071

10 1 0 0.839868 3.774097 3.935395

11 6 0 -0.631008 3.960401 -3.046843

12 1 0 -0.548297 2.895545 -3.314212

13 1 0 -1.622195 4.092009 -2.585610

14 6 0 -1.194337 4.505711 3.765543

15 1 0 -0.935126 5.572855 3.784038

16 1 0 -2.074170 4.380812 3.120172

17 1 0 -1.479847 4.212282 4.783854

18 6 0 -0.428953 4.861843 -4.225052

19 1 0 -0.518697 5.919505 -3.943067

20 1 0 0.559719 4.715356 -4.680393

21 1 0 -1.185368 4.654921 -4.992712

22 1 0 2.422367 3.493359 -0.438816

23 1 0 1.609069 5.010633 -0.042074

---------------------------------------------------------------------

Frequencies -- 36.5714 71.5971 76.9526

Frequencies -- 111.5772 115.0483 145.1976

Frequencies -- 160.7985 204.3484 221.2100

Frequencies -- 269.4383 293.0946 338.7887

Frequencies -- 377.4970 413.3452 433.4033

Frequencies -- 588.2760 621.4766 699.4195

Frequencies -- 800.8265 808.9763 826.8821

Frequencies -- 872.8398 897.0716 967.7862

Frequencies -- 981.7962 1002.8586 1067.2646

Frequencies -- 1079.7050 1142.9988 1146.8212

Frequencies -- 1163.5362 1166.5045 1168.5607

Frequencies -- 1230.8392 1281.7732 1294.6940

Frequencies -- 1312.0902 1345.1475 1375.2130

Frequencies -- 1380.9409 1400.7784 1417.5339

Frequencies -- 1429.8619 1435.9991 1436.6491

Frequencies -- 1455.1929 1457.8491 1485.1577

Frequencies -- 1486.2341 1847.5631 1866.7933

Frequencies -- 3062.4174 3063.8258 3065.0074

Frequencies -- 3069.4287 3098.4421 3130.3515

Frequencies -- 3133.8019 3173.8512 3177.0153

Frequencies -- 3181.0522 3185.5640 3185.7772

SCF Done: E(RM06L-D3/def2-SVP/SMD) = -574.411640924

Sum of electronic and zero-point Energies= -574.220869

Sum of electronic and thermal Energies= -574.208484

Sum of electronic and thermal Free Energies= -574.260515

SCF Done: E(RM06L-D3/def2-TZVPP/SMD) = -575.058040563

**P19 (Diethyl ketomalonate)**

Center Atomic Atomic Coordinates (Angstroms)

Number Number Type X Y Z

---------------------------------------------------------------------

1 8 0 1.397170 2.842636 1.313089

2 8 0 -0.908680 3.005863 -0.872385

3 6 0 0.596641 4.638222 -0.009632

4 6 0 -0.129895 3.895054 -1.126222

5 6 0 0.705210 3.830713 1.274338

6 8 0 0.047828 4.378285 2.280100

7 8 0 0.151455 4.378661 -2.320766

8 6 0 0.146538 3.698394 3.556454

9 1 0 -0.037389 2.626423 3.391434

10 1 0 1.182872 3.795970 3.913990

11 6 0 -0.556091 3.782445 -3.432887

12 1 0 -0.353370 2.700693 -3.431765

13 1 0 -1.635502 3.904785 -3.256211

14 6 0 -0.842299 4.314763 4.493371

15 1 0 -0.642504 5.383303 4.647446

16 1 0 -1.869240 4.207686 4.119694

17 1 0 -0.786783 3.818425 5.470444

18 6 0 -0.101566 4.450613 -4.691045

19 1 0 -0.308837 5.528584 -4.672007

20 1 0 0.974987 4.311238 -4.855163

21 1 0 -0.631021 4.021601 -5.551043

22 8 0 1.077381 5.735531 -0.128115

---------------------------------------------------------------------

Frequencies -- 16.0051 50.5572 77.8490

Frequencies -- 104.1654 116.6580 129.1734

Frequencies -- 149.5246 155.7384 209.0348

Frequencies -- 263.3978 274.2044 287.6516

Frequencies -- 307.8536 344.0286 387.9866

Frequencies -- 408.5439 506.3633 555.1327

Frequencies -- 740.0121 796.9215 812.6540

Frequencies -- 830.5803 846.2542 887.5327

Frequencies -- 894.3427 915.5245 1044.4705

Frequencies -- 1052.5132 1092.1718 1147.0209

Frequencies -- 1152.5348 1165.6802 1168.9410

Frequencies -- 1286.1476 1320.8299 1326.9718

Frequencies -- 1355.2806 1382.9872 1393.2049

Frequencies -- 1424.6274 1433.0381 1438.5806

Frequencies -- 1443.2322 1457.8129 1460.7956

Frequencies -- 1482.3551 1486.7958 1828.8897

Frequencies -- 1845.6943 1858.1578 3067.1893

Frequencies -- 3067.5719 3078.5516 3081.3650

Frequencies -- 3145.1731 3148.0875 3184.1741

Frequencies -- 3185.6075 3191.1221 3192.6070

SCF Done: E(RM06L-D3/def2-SVP/SMD) = -648.340963493

Sum of electronic and zero-point Energies= -648.169058

Sum of electronic and thermal Energies= -648.155783

Sum of electronic and thermal Free Energies= -648.210751

SCF Done: E(RM06L-D3/def2-TZVPP/SMD) = -649.07272945

**P20**

Center Atomic Atomic Coordinates (Angstroms)

Number Number Type X Y Z

---------------------------------------------------------------------

1 83 0 -1.455096 1.438241 -2.205299

2 35 0 1.437782 3.155359 -1.289247

3 35 0 -1.896574 3.963726 -3.171523

4 83 0 1.409521 4.051412 -3.840770

5 35 0 -2.740936 1.754687 0.105838

6 35 0 -3.367365 0.230103 -3.612790

7 35 0 0.772927 1.639017 -4.884401

8 35 0 4.031824 3.904058 -4.253096

---------------------------------------------------------------------

Frequencies -- 16.9724 27.2609 30.9332

Frequencies -- 43.2201 45.9055 49.7175

Frequencies -- 65.8797 66.7336 74.1767

Frequencies -- 78.6771 85.3386 93.0571

Frequencies -- 176.5662 180.8774 191.4418

Frequencies -- 200.5842 205.6488 206.1069

SCF Done: E(RM06L-D3/def2-SVP/SMD) = -15872.3364034

Sum of electronic and zero-point Energies= -15872.332214

Sum of electronic and thermal Energies= -15872.316086

Sum of electronic and thermal Free Energies= -15872.388053

SCF Done: E(RM06L-D3/def2-TZVPP/SMD) = -15874.2335655

**P21**

Center Atomic Atomic Coordinates (Angstroms)

Number Number Type X Y Z

---------------------------------------------------------------------

1 83 0 -1.787404 1.927808 -2.266405

2 8 0 -0.175676 3.120272 -1.536226

3 8 0 -3.136288 2.856272 -0.878141

4 8 0 -1.545322 0.089573 -1.199292

5 35 0 -4.730384 1.942536 -0.650740

6 35 0 -0.075215 3.692706 0.217271

7 35 0 -1.553747 0.049973 0.646814

---------------------------------------------------------------------

Frequencies -- 33.4452 46.2010 54.7435

Frequencies -- 68.6167 89.0891 114.4720

Frequencies -- 140.3159 163.0189 178.1236

Frequencies -- 381.1473 391.6095 444.8034

Frequencies -- 642.6418 659.6073 671.8802

SCF Done: E(RM06L-D3/def2-SVP/SMD) = -8161.37830661

Sum of electronic and zero-point Energies= -8161.369012

Sum of electronic and thermal Energies= -8161.358199

Sum of electronic and thermal Free Energies= -8161.412246

SCF Done: E(RM06L-D3/def2-TZVPP/SMD) = -8162.61699982

**P22**

Center Atomic Atomic Coordinates (Angstroms)

Number Number Type X Y Z

---------------------------------------------------------------------

1 83 0 -1.797940 1.875561 -1.878473

2 35 0 -3.804017 3.427822 -0.978254

3 35 0 0.549083 2.828113 -0.965634

4 35 0 -2.143650 -0.643887 -0.997081

5 35 0 -1.789401 1.889916 -4.571132

---------------------------------------------------------------------

Frequencies -- 28.8045 33.0276 34.0778

Frequencies -- 39.0705 40.9626 174.6832

Frequencies -- 185.3041 185.8034 186.5190

SCF Done: E(RM06L-D3/def2-SVP/SMD) = -10509.8898230

Sum of electronic and zero-point Energies= -10509.887754

Sum of electronic and thermal Energies= -10509.878239

Sum of electronic and thermal Free Energies= -10509.932560

SCF Done: E(RM06L-D3/def2-TZVPP/SMD) = -10511.1515209

**P23**

Center Atomic Atomic Coordinates (Angstroms)

Number Number Type X Y Z

---------------------------------------------------------------------

1 83 0 -1.690385 1.487019 -2.202764

2 35 0 1.273057 3.317395 -1.289783

3 35 0 -1.868483 4.067405 -3.398245

4 83 0 1.456485 3.866845 -3.921361

5 35 0 -2.940351 1.988512 0.083432

6 35 0 -3.523713 0.174726 -3.600593

7 35 0 0.503400 1.456939 -4.960394

8 35 0 4.072320 3.599366 -4.283105

9 35 0 -0.002805 -0.529486 -1.447824

10 35 0 1.084935 6.543857 -4.332046

---------------------------------------------------------------------

Frequencies -- 14.8095 19.9008 25.0675

Frequencies -- 30.5151 31.5608 38.6508

Frequencies -- 40.5027 47.9728 51.4105

Frequencies -- 54.2225 56.4616 57.3136

Frequencies -- 62.5252 71.0807 76.7311

Frequencies -- 83.1722 136.6654 148.0968

Frequencies -- 153.1812 157.4211 187.6443

Frequencies -- 194.9618 200.4306 200.7755

SCF Done: E(RM06L-D3/def2-SVP/SMD) = -21019.7942778

Sum of electronic and zero-point Energies= -21019.789400

Sum of electronic and thermal Energies= -21019.768268

Sum of electronic and thermal Free Energies= -21019.856566

SCF Done: E(RM06L-D3/def2-TZVPP/SMD) = -21022.3165365

**P24**

Center Atomic Atomic Coordinates (Angstroms)

Number Number Type X Y Z

---------------------------------------------------------------------

1 83 0 -1.141203 -0.089741 0.798677

2 8 0 -1.225152 2.348786 1.736250

3 8 0 -1.112378 1.488005 -1.026011

4 8 0 -0.599387 -2.159531 1.822472

5 8 0 -0.562676 -1.337308 -1.006523

6 6 0 0.238932 -3.397903 -0.049183

7 6 0 0.242020 3.159592 0.031444

8 1 0 1.010717 3.903704 -0.172403

9 1 0 0.709396 -4.340621 -0.319985

10 6 0 -0.304402 2.433193 -1.084004

11 6 0 -0.360109 3.138922 1.345856

12 6 0 -0.033450 -2.493510 -1.079560

13 6 0 -0.060664 -3.178776 1.305522

14 8 0 0.312467 -2.922386 -2.295979

15 8 0 0.279069 -4.191440 2.109054

16 8 0 0.121458 4.096987 2.129152

17 8 0 0.133430 2.871293 -2.251101

18 6 0 0.147555 -2.028066 -3.406101

19 1 0 -0.882104 -1.639531 -3.414022

20 1 0 0.819306 -1.165266 -3.267938

21 6 0 0.036936 -4.038070 3.514693

22 1 0 0.575891 -3.148903 3.878316

23 1 0 -1.035020 -3.846893 3.679071

24 6 0 -0.374594 4.137578 3.481893

25 1 0 -1.468278 4.256989 3.451069

26 1 0 -0.175431 3.162997 3.954371

27 6 0 -0.308158 2.152558 -3.425146

28 1 0 -0.140783 1.079145 -3.251485

29 1 0 -1.395300 2.291531 -3.523755

30 6 0 0.469716 -2.787075 -4.656755

31 1 0 1.499451 -3.168743 -4.642523

32 1 0 -0.208399 -3.640275 -4.791652

33 1 0 0.365559 -2.132931 -5.531856

34 6 0 0.495244 -5.290569 4.197205

35 1 0 -0.052006 -6.169700 3.831156

36 1 0 1.567158 -5.466790 4.035136

37 1 0 0.326943 -5.216425 5.279075

38 6 0 0.301734 5.266646 4.193486

39 1 0 1.391058 5.131839 4.218194

40 1 0 0.086927 6.231277 3.714805

41 1 0 -0.054403 5.319595 5.229923

42 6 0 0.446293 2.675725 -4.605593

43 1 0 0.264560 3.747366 -4.760378

44 1 0 1.527705 2.523706 -4.492371

45 1 0 0.125889 2.147240 -5.512218

46 35 0 1.809279 0.806849 0.700917

47 35 0 -3.810805 -0.521070 0.367709

---------------------------------------------------------------------

Frequencies -- 16.2540 20.3360 29.7952

Frequencies -- 37.8833 43.1883 48.5408

Frequencies -- 50.2136 55.2200 70.5314

Frequencies -- 75.5320 78.8720 85.5307

Frequencies -- 93.4476 95.6000 109.4981

Frequencies -- 117.0036 117.9608 125.9943

Frequencies -- 133.7889 139.4129 154.6850

Frequencies -- 159.5419 163.5611 169.7013

Frequencies -- 173.5613 180.2375 192.6395

Frequencies -- 203.3042 214.6729 230.6927

Frequencies -- 238.6836 270.3878 280.6669

Frequencies -- 286.1733 286.5366 297.8249

Frequencies -- 305.9863 340.1023 352.9887

Frequencies -- 384.7917 404.0436 433.7443

Frequencies -- 437.1913 455.5673 470.0484

Frequencies -- 669.9382 714.5337 720.3862

Frequencies -- 727.6908 733.2384 740.4449

Frequencies -- 780.7193 782.9757 800.0553

Frequencies -- 804.7453 811.6020 820.2541

Frequencies -- 822.8774 844.4521 883.9762

Frequencies -- 890.1028 917.0130 929.2431

Frequencies -- 1017.8743 1030.2763 1044.8713

Frequencies -- 1051.7291 1060.0190 1076.4257

Frequencies -- 1105.6655 1120.2361 1144.1091

Frequencies -- 1145.7230 1147.3972 1149.0018

Frequencies -- 1157.7140 1164.9909 1170.2486

Frequencies -- 1171.3131 1186.0261 1200.6258

Frequencies -- 1293.3332 1297.9296 1307.5371

Frequencies -- 1307.8525 1332.8577 1351.4343

Frequencies -- 1363.3466 1368.8656 1386.2694

Frequencies -- 1393.1271 1411.5337 1412.7002

Frequencies -- 1429.5986 1437.7737 1439.9736

Frequencies -- 1441.9233 1442.1909 1443.0208

Frequencies -- 1450.8479 1456.3594 1459.1363

Frequencies -- 1461.4848 1469.8621 1479.0269

Frequencies -- 1482.2323 1492.8505 1514.5147

Frequencies -- 1525.9691 1607.5867 1644.6375

Frequencies -- 1667.5412 1718.4317 3062.9365

Frequencies -- 3065.2449 3065.6784 3065.9966

Frequencies -- 3066.9602 3068.4637 3071.8715

Frequencies -- 3077.6109 3131.5731 3134.5018

Frequencies -- 3136.4624 3142.2134 3180.9051

Frequencies -- 3182.6150 3183.4825 3185.4472

Frequencies -- 3187.7043 3188.6500 3188.6866

Frequencies -- 3191.6293 3248.5447 3252.7632

SCF Done: E(RM06L-D3/def2-SVP/SMD) = -6509.98061406

Sum of electronic and zero-point Energies= -6509.619429

Sum of electronic and thermal Energies= -6509.587461

Sum of electronic and thermal Free Energies= -6509.689141

SCF Done: E(RM06L-D3/def2-TZVPP/SMD) = -6511.89890468

**P25**

Center Atomic Atomic Coordinates (Angstroms)

Number Number Type X Y Z

---------------------------------------------------------------------

1 83 0 -0.615999 0.292503 -0.203410

2 8 0 0.617518 1.357915 1.388508

3 8 0 1.483305 -0.996426 -0.038950

4 8 0 -0.922135 -1.895156 1.342452

5 8 0 -0.841993 -2.029107 -1.579066

6 6 0 0.048496 -3.603823 -0.009774

7 6 0 2.840808 0.445901 1.296236

8 1 0 3.855377 0.602111 1.657211

9 1 0 0.516064 -4.587785 0.064509

10 6 0 2.578124 -0.681406 0.498003

11 6 0 1.860469 1.370104 1.667997

12 6 0 -0.237073 -3.066558 -1.334245

13 6 0 -0.109471 -2.799589 1.197628

14 8 0 0.281078 -3.843040 -2.277460

15 8 0 0.770842 -3.155817 2.122791

16 8 0 2.294495 2.373377 2.434565

17 8 0 3.635294 -1.487995 0.316541

18 6 0 0.194401 -3.357506 -3.635878

19 1 0 -0.867108 -3.333360 -3.926331

20 1 0 0.552203 -2.315902 -3.649853

21 6 0 0.889575 -2.294745 3.279158

22 1 0 0.806335 -1.250384 2.939035

23 1 0 0.032633 -2.487132 3.942756

24 6 0 1.342294 3.367398 2.846660

25 1 0 0.795153 3.733940 1.965241

26 1 0 0.595177 2.898606 3.506449

27 6 0 3.447063 -2.638797 -0.516193

28 1 0 2.701324 -3.303657 -0.047012

29 1 0 3.028196 -2.321451 -1.485368

30 6 0 1.009209 -4.262299 -4.503720

31 1 0 2.064305 -4.267753 -4.198518

32 1 0 0.637077 -5.294719 -4.472302

33 1 0 0.963108 -3.919704 -5.544856

34 6 0 2.205133 -2.577169 3.931096

35 1 0 2.270348 -3.615257 4.282434

36 1 0 3.037896 -2.393545 3.238149

37 1 0 2.336631 -1.917871 4.798303

38 6 0 2.096070 4.461113 3.538934

39 1 0 2.629807 4.086270 4.422622

40 1 0 2.832101 4.924902 2.868560

41 1 0 1.402950 5.244396 3.871457

42 6 0 4.768430 -3.324982 -0.675000

43 1 0 5.509311 -2.663634 -1.143663

44 1 0 5.169809 -3.655613 0.292397

45 1 0 4.655193 -4.210690 -1.313055

46 35 0 -2.767088 1.874934 0.536611

47 35 0 0.396843 1.749279 -2.261805

---------------------------------------------------------------------

Frequencies -- 17.0525 18.4290 32.5409

Frequencies -- 36.5785 40.9393 43.3637

Frequencies -- 52.4285 53.3634 59.9181

Frequencies -- 66.5822 71.3497 90.8461

Frequencies -- 92.0039 95.4905 107.7430

Frequencies -- 115.5572 124.8814 136.0441

Frequencies -- 143.5124 153.0145 155.5696

Frequencies -- 161.3492 167.1772 175.5446

Frequencies -- 177.7678 184.7798 201.1547

Frequencies -- 203.9418 208.3465 224.2376

Frequencies -- 236.2439 250.7324 266.6079

Frequencies -- 279.4871 296.2060 301.5282

Frequencies -- 309.7749 339.1516 351.7502

Frequencies -- 361.4511 402.7838 416.4194

Frequencies -- 423.2370 451.3860 464.2648

Frequencies -- 553.6294 691.2262 719.1541

Frequencies -- 723.1257 731.2995 739.9200

Frequencies -- 768.4793 779.7253 798.3713

Frequencies -- 804.8138 814.6908 815.3230

Frequencies -- 830.3132 834.3069 877.8622

Frequencies -- 889.7061 909.5235 928.2905

Frequencies -- 999.9520 1033.9747 1037.9438

Frequencies -- 1052.6910 1053.1628 1077.0902

Frequencies -- 1087.9243 1116.8057 1144.6472

Frequencies -- 1144.7732 1146.7638 1148.5669

Frequencies -- 1162.6497 1166.8247 1167.0919

Frequencies -- 1169.3103 1182.2351 1189.1514

Frequencies -- 1286.8014 1291.0415 1299.5909

Frequencies -- 1308.4318 1320.6759 1343.6119

Frequencies -- 1360.3716 1363.9533 1374.2899

Frequencies -- 1389.4941 1408.8133 1409.4907

Frequencies -- 1419.7238 1434.8392 1437.4624

Frequencies -- 1438.4909 1438.6004 1438.9474

Frequencies -- 1451.4862 1456.7140 1457.4010

Frequencies -- 1462.6168 1464.5268 1480.3691

Frequencies -- 1486.7451 1493.3865 1499.2201

Frequencies -- 1511.8445 1601.5794 1672.3091

Frequencies -- 1711.0533 1753.4302 3046.0610

Frequencies -- 3063.3325 3064.6791 3065.0681

Frequencies -- 3066.1954 3068.0192 3072.1972

Frequencies -- 3074.9785 3109.6340 3137.2033

Frequencies -- 3138.0481 3140.1432 3177.7885

Frequencies -- 3181.3023 3181.8055 3183.0723

Frequencies -- 3187.1361 3189.2949 3190.0043

Frequencies -- 3194.2228 3222.9881 3244.5451

SCF Done: E(RM06L-D3/def2-SVP/SMD) = -6509.98056326

Sum of electronic and zero-point Energies= -6509.620342

Sum of electronic and thermal Energies= -6509.588215

Sum of electronic and thermal Free Energies= -6509.690275

SCF Done: E(RM06L-D3/def2-TZVPP/SMD) = -6511.89829094

**P26**

Center Atomic Atomic Coordinates (Angstroms)

Number Number Type X Y Z

---------------------------------------------------------------------

1 83 0 -1.796954 1.876636 -1.878443

2 35 0 -3.987142 2.856276 -0.734291

3 35 0 0.572220 2.232444 -0.726961

4 35 0 -2.120997 -0.429387 -0.511723

5 35 0 -1.972383 0.537678 -4.168949

6 35 0 -1.477854 4.179385 -3.248321

---------------------------------------------------------------------

Frequencies -- 23.8443 25.5056 77.6183

Frequencies -- 78.7578 81.1271 82.1185

Frequencies -- 87.3383 158.4687 170.3679

Frequencies -- 184.0488 199.3487 200.5245

SCF Done: E(RM06L-D3/def2-SVP/SMD) = -13083.6158599

Sum of electronic and zero-point Energies= -13083.612741

Sum of electronic and thermal Energies= -13083.601331

Sum of electronic and thermal Free Energies= -13083.658554

SCF Done: E(RM06L-D3/def2-TZVPP/SMD) = -13085.1938841

**P27**

Center Atomic Atomic Coordinates (Angstroms)

Number Number Type X Y Z

---------------------------------------------------------------------

1 83 0 -1.335570 1.544992 -2.600612

2 35 0 1.046703 2.892351 -1.029266

3 35 0 -1.623913 4.231051 -3.676451

4 83 0 1.403077 4.271703 -3.989154

5 35 0 -0.727497 1.362406 0.801668

6 35 0 -3.158138 0.270299 -4.344750

7 35 0 0.702362 1.292647 -4.584735

8 35 0 3.998561 3.567687 -3.750256

9 35 0 -0.566184 -0.922827 -1.122129

10 35 0 1.281013 4.933092 -6.555902

11 35 0 -4.200955 1.456855 -1.768568

12 35 0 1.533252 6.700534 -2.845527

---------------------------------------------------------------------

Frequencies -- 17.6194 18.7814 23.7136

Frequencies -- 26.1990 28.9630 29.2898

Frequencies -- 35.1164 35.9965 40.4319

Frequencies -- 47.0153 53.6811 56.6114

Frequencies -- 58.0178 59.8340 65.4677

Frequencies -- 71.3056 72.2971 93.3583

Frequencies -- 100.3413 106.6550 112.2330

Frequencies -- 120.3674 123.1031 139.3163

Frequencies -- 142.1291 155.3616 169.0127

Frequencies -- 170.0002 176.6854 193.0725

SCF Done: E(RM06L-D3/def2-SVP/SMD) = -26167.2792147

Sum of electronic and zero-point Energies= -26167.273424

Sum of electronic and thermal Energies= -26167.247512

Sum of electronic and thermal Free Energies= -26167.348643

SCF Done: E(RM06L-D3/def2-TZVPP/SMD) = -26170.4204993

**P28**

Center Atomic Atomic Coordinates (Angstroms)

Number Number Type X Y Z

---------------------------------------------------------------------

1 83 0 -1.715374 1.909494 -1.941015

2 35 0 -3.783150 3.258379 -0.947670

3 35 0 -2.247083 -0.508377 -0.965123

---------------------------------------------------------------------

Frequencies -- 65.1127 199.8020 208.3602

SCF Done: E(RM06L-D3/def2-SVP/SMD) = -5362.34400535

Sum of electronic and zero-point Energies= -5362.342927

Sum of electronic and thermal Energies= -5362.338182

Sum of electronic and thermal Free Energies= -5362.377062

SCF Done: E(RM06L-D3/def2-TZVPP/SMD) = -5362.97986829

**P29**

Center Atomic Atomic Coordinates (Angstroms)

Number Number Type X Y Z

---------------------------------------------------------------------

1 83 0 -0.632980 -0.554615 -0.065089

2 83 0 0.557314 0.519051 2.554130

3 35 0 2.646000 -0.069544 0.912612

4 35 0 -0.202732 -2.703786 1.546795

5 35 0 0.568104 3.180792 2.262052

6 35 0 -3.285041 -0.353296 0.245148

---------------------------------------------------------------------

Frequencies -- 23.7732 32.6063 35.4387

Frequencies -- 58.8660 61.3365 62.7984

Frequencies -- 73.4312 131.9571 183.3897

Frequencies -- 192.0655 197.4906 199.3410

SCF Done: E(RM06L-D3/def2-SVP/SMD) = -10724.7473510

Sum of electronic and zero-point Energies= -10724.744498

Sum of electronic and thermal Energies= -10724.732851

Sum of electronic and thermal Free Energies= -10724.792304

SCF Done: E(RM06L-D3/def2-TZVPP/SMD) = -10726.0175765

**P30**

Center Atomic Atomic Coordinates (Angstroms)

Number Number Type X Y Z

---------------------------------------------------------------------

1 83 0 8.053093 2.896786 3.397559

2 35 0 9.902271 1.135231 2.075119

3 35 0 7.418612 1.433929 5.774621

4 35 0 9.685985 4.947225 4.518927

5 8 0 7.654483 4.015836 1.235083

6 16 0 8.301569 3.600650 -0.106058

7 6 0 7.569537 4.758960 -1.264711

8 6 0 7.429192 2.118925 -0.620325

9 8 0 6.369182 1.375606 2.357949

10 16 0 6.438169 -0.115700 2.771803

11 6 0 4.863160 -0.408000 3.577068

12 6 0 6.126541 -0.990977 1.236298

13 8 0 6.179173 4.315134 4.115609

14 16 0 6.266445 4.805234 5.584789

15 6 0 6.207983 6.589457 5.434814

16 6 0 4.612039 4.510935 6.203841

17 1 0 6.476145 4.728668 -1.179191

18 1 0 7.938052 5.757412 -1.001780

19 1 0 7.888726 4.510567 -2.285228

20 1 0 7.774650 1.325660 0.052185

21 1 0 6.348134 2.272990 -0.512817

22 1 0 7.698657 1.878822 -1.657410

23 1 0 4.897566 0.151920 4.519667

24 1 0 4.047528 -0.041965 2.940412

25 1 0 4.745117 -1.478762 3.788302

26 1 0 5.278380 -0.533803 0.710157

27 1 0 7.036426 -0.920901 0.628819

28 1 0 5.924471 -2.048196 1.452208

29 1 0 7.159226 6.883579 4.974652

30 1 0 5.364982 6.886703 4.798292

31 1 0 6.124257 7.043052 6.431126

32 1 0 3.872515 4.938787 5.515301

33 1 0 4.493923 3.421997 6.255731

34 1 0 4.505783 4.938663 7.209257

---------------------------------------------------------------------

Frequencies -- 24.4758 31.9590 34.5929

Frequencies -- 40.0491 46.1920 54.4319

Frequencies -- 57.9748 63.9137 67.9584

Frequencies -- 78.8479 84.6471 87.3109

Frequencies -- 94.7613 105.3865 109.2607

Frequencies -- 117.0565 120.2803 123.6710

Frequencies -- 145.4516 152.1034 153.7259

Frequencies -- 157.5296 161.1692 170.5853

Frequencies -- 183.6004 223.1691 226.3998

Frequencies -- 230.3587 272.4385 275.6063

Frequencies -- 304.8232 308.0919 309.3838

Frequencies -- 339.5688 340.8126 342.0722

Frequencies -- 390.7208 411.8223 419.9537

Frequencies -- 678.5872 685.3218 690.3591

Frequencies -- 709.2383 716.7291 723.0550

Frequencies -- 891.7757 903.7015 907.4806

Frequencies -- 929.6137 937.3128 940.1290

Frequencies -- 957.8464 960.4000 961.7181

Frequencies -- 995.7422 998.1438 1010.9532

Frequencies -- 1028.9513 1035.8866 1039.1642

Frequencies -- 1284.9100 1288.2308 1292.2916

Frequencies -- 1303.3753 1311.4560 1313.0752

Frequencies -- 1380.9482 1389.1364 1390.3850

Frequencies -- 1392.4857 1396.2206 1401.2777

Frequencies -- 1402.9909 1407.5496 1410.5950

Frequencies -- 1413.1065 1424.4188 1429.9710

Frequencies -- 3057.1236 3058.8232 3060.1409

Frequencies -- 3060.6753 3063.6466 3063.8832

Frequencies -- 3207.5174 3208.7700 3209.4912

Frequencies -- 3210.4342 3211.0287 3212.4655

Frequencies -- 3213.7613 3215.0130 3216.2392

Frequencies -- 3220.4400 3221.4518 3226.9873

SCF Done: E(RM06L-D3/def2-SVP/SMD) = -9595.02900762

Sum of electronic and zero-point Energies= -9594.784937

Sum of electronic and thermal Energies= -9594.756383

Sum of electronic and thermal Free Energies= -9594.849910

SCF Done: E(RM06L-D3/def2-TZVPP/SMD) = -9596.97308354

**P31**

Center Atomic Atomic Coordinates (Angstroms)

Number Number Type X Y Z

---------------------------------------------------------------------

1 83 0 -1.306327 1.616467 -2.371095

2 35 0 0.717378 3.214634 -0.672296

3 35 0 -2.150307 4.444801 -3.149572

4 83 0 0.883789 5.035645 -3.091274

5 35 0 -3.909548 0.560197 -2.892340

6 35 0 3.661314 4.815648 -2.929916

7 8 0 -2.296302 1.711945 -0.227233

8 16 0 -2.888156 0.415111 0.396567

9 6 0 -4.480470 0.969636 0.991778

10 6 0 -1.997879 0.306008 1.945784

11 1 0 -5.086976 1.156550 0.097419

12 1 0 -4.361018 1.893233 1.571881

13 1 0 -4.946450 0.182445 1.598389

14 1 0 -0.954758 0.095541 1.679455

15 1 0 -2.400223 -0.517916 2.549389

16 1 0 -2.068228 1.259235 2.484271

17 8 0 -0.947456 0.297656 -4.482213

18 16 0 -1.472776 -1.163647 -4.544210

19 6 0 -0.053182 -2.091511 -5.130187

20 6 0 -2.450695 -1.173670 -6.044180

21 1 0 0.655415 -2.150411 -4.294335

22 1 0 0.406477 -1.592674 -5.994271

23 1 0 -0.370278 -3.107887 -5.399338

24 1 0 -3.312911 -0.524928 -5.848516

25 1 0 -2.799396 -2.193933 -6.250511

26 1 0 -1.863143 -0.785815 -6.886166

27 8 0 1.170824 7.417904 -2.580075

28 16 0 2.289911 8.198759 -3.320956

29 6 0 3.123783 9.070014 -1.997507

30 6 0 1.417867 9.566110 -4.078141

31 1 0 3.637008 8.308628 -1.398083

32 1 0 2.390527 9.606190 -1.382098

33 1 0 3.863349 9.763030 -2.418841

34 1 0 0.805531 9.132040 -4.877376

35 1 0 2.140805 10.268822 -4.512358

36 1 0 0.781860 10.065325 -3.336779

37 8 0 0.764731 2.806086 -3.983863

38 16 0 1.883228 2.296128 -4.954026

39 6 0 0.984375 1.830504 -6.424725

40 6 0 2.261753 0.691812 -4.263758

41 1 0 0.659213 2.770064 -6.888494

42 1 0 0.124423 1.215519 -6.130406

43 1 0 1.660960 1.296063 -7.104781

44 1 0 2.765552 0.874387 -3.305847

45 1 0 2.931314 0.142889 -4.939175

46 1 0 1.317304 0.157634 -4.100628

47 35 0 0.970503 5.783695 -5.807598

48 35 0 -0.121819 -0.783266 -1.386614

---------------------------------------------------------------------

Frequencies -- 16.9288 19.7781 24.4681

Frequencies -- 32.1154 36.5520 40.5261

Frequencies -- 46.0124 46.4085 47.6506

Frequencies -- 53.4772 57.1695 59.4952

Frequencies -- 60.9743 68.8580 71.5362

Frequencies -- 72.7675 74.8016 75.8013

Frequencies -- 76.5942 80.0167 89.4906

Frequencies -- 91.0354 96.9375 103.6720

Frequencies -- 113.2957 117.4093 121.2154

Frequencies -- 123.9113 129.8780 133.3860

Frequencies -- 133.4341 138.5552 144.0614

Frequencies -- 148.0526 148.9931 160.8994

Frequencies -- 162.6083 169.0997 173.9541

Frequencies -- 182.0044 190.5499 203.1077

Frequencies -- 225.9284 228.9250 231.4297

Frequencies -- 238.5393 270.6963 273.0496

Frequencies -- 278.4342 280.3939 310.8219

Frequencies -- 311.7792 315.0726 318.5958

Frequencies -- 332.7572 337.8970 340.8991

Frequencies -- 348.5028 407.2977 412.2661

Frequencies -- 434.8935 438.7780 682.2230

Frequencies -- 684.3332 694.3984 699.0525

Frequencies -- 713.4757 718.7739 730.0412

Frequencies -- 731.8737 896.2340 901.1908

Frequencies -- 903.1616 907.8875 935.1098

Frequencies -- 936.1647 942.5780 945.7787

Frequencies -- 946.1742 949.8415 960.1884

Frequencies -- 963.3121 987.1406 989.0979

Frequencies -- 996.3609 1001.7594 1025.3743

Frequencies -- 1030.4251 1034.6756 1044.3167

Frequencies -- 1277.4247 1286.4410 1290.4345

Frequencies -- 1294.3177 1301.3962 1307.7227

Frequencies -- 1310.3540 1314.3191 1380.7203

Frequencies -- 1390.0405 1390.6005 1393.0584

Frequencies -- 1396.5839 1399.9055 1400.9129

Frequencies -- 1401.5813 1402.6792 1405.5662

Frequencies -- 1408.3692 1412.2147 1421.1274

Frequencies -- 1421.5737 1426.3900 1430.1406

Frequencies -- 3053.3957 3057.5300 3061.1102

Frequencies -- 3063.1093 3063.6468 3064.1314

Frequencies -- 3064.5675 3065.7678 3203.3377

Frequencies -- 3209.3373 3211.1554 3212.0096

Frequencies -- 3212.6492 3213.7831 3214.1063

Frequencies -- 3216.0918 3217.6718 3218.7701

Frequencies -- 3218.8688 3219.8677 3221.0243

Frequencies -- 3221.2228 3221.8262 3222.6405

SCF Done: E(RM06L-D3/def2-SVP/SMD) = -18084.1926425

Sum of electronic and zero-point Energies= -18083.864047

Sum of electronic and thermal Energies= -18083.819991

Sum of electronic and thermal Free Energies= -18083.951633

SCF Done: E(RM06L-D3/def2-TZVPP/SMD) = -18087.4085915

**P32**

Center Atomic Atomic Coordinates (Angstroms)

Number Number Type X Y Z

---------------------------------------------------------------------

1 83 0 11.557780 1.547272 -0.308483

2 83 0 11.120125 0.978020 3.772197

3 83 0 7.716252 -3.792470 4.776934

4 16 0 11.123488 -4.964381 3.760556

5 8 0 9.933846 -4.901604 4.754520

6 6 0 12.177926 -3.579783 4.183356

7 1 0 12.610179 -3.782060 5.171247

8 1 0 11.596695 -2.647856 4.189653

9 1 0 12.986721 -3.507890 3.444161

10 6 0 10.465849 -4.334454 2.216437

11 1 0 9.693080 -5.039603 1.886991

12 1 0 11.265765 -4.287243 1.466203

13 1 0 10.057897 -3.328272 2.396846

14 16 0 7.705585 -3.147531 0.814445

15 8 0 7.285962 -3.631800 2.221972

16 6 0 7.939979 -1.371975 0.888570

17 1 0 8.908358 -1.167147 1.361239

18 1 0 7.125287 -0.921360 1.470330

19 1 0 7.972177 -0.978484 -0.137155

20 6 0 6.175588 -3.154018 -0.125192

21 1 0 5.848615 -4.193204 -0.246469

22 1 0 6.364079 -2.726618 -1.118532

23 1 0 5.414713 -2.562640 0.401259

24 16 0 10.167483 -2.558905 6.890597

25 8 0 8.721224 -3.172046 6.881499

26 6 0 11.013077 -3.532969 8.130054

27 1 0 11.113373 -4.550403 7.731519

28 1 0 10.458021 -3.539514 9.077552

29 1 0 12.014001 -3.104332 8.275976

30 6 0 9.964701 -1.030039 7.790626

31 1 0 9.508761 -0.300365 7.107652

32 1 0 10.954711 -0.659348 8.083856

33 1 0 9.332329 -1.211165 8.669998

34 16 0 4.603444 -3.447747 3.544580

35 8 0 5.465166 -4.501273 4.293246

36 6 0 3.205341 -3.196439 4.630851

37 1 0 3.590490 -2.676645 5.515024

38 1 0 2.777761 -4.165303 4.917228

39 1 0 2.455199 -2.572877 4.127006

40 6 0 3.808863 -4.428890 2.277667

41 1 0 4.612573 -4.837849 1.656048

42 1 0 3.145637 -3.800018 1.669381

43 1 0 3.248632 -5.250515 2.740832

44 16 0 6.554263 -0.997644 6.536315

45 8 0 6.146380 -2.305013 5.778449

46 6 0 5.408654 -0.983245 7.909955

47 1 0 5.667178 -1.828043 8.559132

48 1 0 4.375486 -1.080046 7.553871

49 1 0 5.537158 -0.046903 8.468109

50 6 0 5.841920 0.309306 5.546293

51 1 0 6.454083 0.394125 4.639383

52 1 0 5.902050 1.250380 6.108491

53 1 0 4.799246 0.066824 5.302275

54 16 0 8.375202 -5.956434 7.370206

55 8 0 7.333404 -5.562324 6.278846

56 6 0 8.068019 -7.706882 7.557179

57 1 0 8.404724 -8.197522 6.636767

58 1 0 6.996708 -7.884456 7.711595

59 1 0 8.652961 -8.086597 8.404579

60 6 0 7.641111 -5.366737 8.891056

61 1 0 7.664271 -4.272395 8.840053

62 1 0 8.237436 -5.715219 9.744660

63 1 0 6.607876 -5.728090 8.966786

64 35 0 12.911812 2.815914 2.600830

65 35 0 9.143987 1.897140 1.694990

66 35 0 11.882844 -0.988246 1.557074

67 35 0 10.016488 0.074856 -2.079784

68 35 0 14.010650 1.088580 -1.533257

69 35 0 11.129488 4.033793 -1.463921

70 35 0 9.803758 2.403796 5.829338

71 35 0 12.869445 -0.358373 5.612849

72 35 0 8.886336 -1.168767 4.175339

---------------------------------------------------------------------

Frequencies -- 18.7501 20.6884 22.5859

Frequencies -- 24.2929 30.8422 34.4467

Frequencies -- 34.8060 38.8043 40.0441

Frequencies -- 44.3567 45.9659 47.3389

Frequencies -- 48.5675 52.2917 53.7446

Frequencies -- 56.1663 58.3472 58.9706

Frequencies -- 60.5661 61.9099 64.4768

Frequencies -- 65.2172 68.0793 70.4406

Frequencies -- 72.9015 77.1943 79.9412

Frequencies -- 81.8152 83.5426 87.3507

Frequencies -- 89.5371 91.7892 94.4191

Frequencies -- 98.0872 103.1039 107.1736

Frequencies -- 111.1794 113.9255 117.6585

Frequencies -- 120.8485 122.7936 127.3237

Frequencies -- 127.6776 131.9362 136.8976

Frequencies -- 138.9875 141.4193 144.8968

Frequencies -- 146.8832 150.6414 152.7988

Frequencies -- 153.4508 155.4125 158.5705

Frequencies -- 160.9913 166.0452 168.1666

Frequencies -- 172.1854 173.4501 176.3981

Frequencies -- 183.3329 186.3408 194.0351

Frequencies -- 205.3796 214.7759 225.3286

Frequencies -- 232.0327 240.7031 252.2237

Frequencies -- 257.4553 278.8206 283.8530

Frequencies -- 294.5544 296.4878 308.4382

Frequencies -- 308.9353 323.6888 326.8604

Frequencies -- 327.4994 332.7687 333.6221

Frequencies -- 336.2666 343.3936 345.6513

Frequencies -- 346.5292 349.6318 353.9526

Frequencies -- 361.0739 366.0076 374.6761

Frequencies -- 381.6419 423.3101 435.9612

Frequencies -- 439.0622 446.8592 457.7364

Frequencies -- 679.3692 687.1865 696.4506

Frequencies -- 700.8337 702.3794 706.5434

Frequencies -- 709.7145 716.5618 730.7954

Frequencies -- 731.3503 736.2873 740.9524

Frequencies -- 892.4006 904.0490 910.9260

Frequencies -- 913.1072 918.5776 921.9799

Frequencies -- 927.1718 929.4140 933.5258

Frequencies -- 943.1980 946.0672 948.2473

Frequencies -- 955.3766 956.8108 957.4799

Frequencies -- 960.4728 966.8233 972.4460

Frequencies -- 989.8716 995.3740 999.2320

Frequencies -- 1002.5089 1008.7829 1018.5273

Frequencies -- 1026.5533 1035.4022 1039.2275

Frequencies -- 1044.4996 1045.1512 1047.6387

Frequencies -- 1271.1160 1284.2431 1288.8200

Frequencies -- 1291.8772 1302.9794 1304.5543

Frequencies -- 1313.0224 1314.4768 1314.7162

Frequencies -- 1325.2027 1331.8227 1332.7998

Frequencies -- 1380.2487 1381.0718 1383.9996

Frequencies -- 1387.1187 1390.6989 1393.1627

Frequencies -- 1396.8951 1397.9297 1402.0173

Frequencies -- 1403.7913 1404.6096 1405.6198

Frequencies -- 1408.2901 1408.7093 1411.3785

Frequencies -- 1412.6964 1417.7993 1419.2615

Frequencies -- 1419.9865 1426.1597 1435.0524

Frequencies -- 1439.5935 1442.1836 1451.2616

Frequencies -- 3049.8065 3053.6883 3058.8549

Frequencies -- 3059.4591 3059.5962 3060.3934

Frequencies -- 3062.2866 3064.8609 3065.5756

Frequencies -- 3068.2446 3070.4259 3070.7768

Frequencies -- 3196.3374 3201.4488 3204.4086

Frequencies -- 3207.0257 3207.1489 3207.7454

Frequencies -- 3208.4408 3209.5129 3212.0712

Frequencies -- 3212.2308 3213.3407 3213.8603

Frequencies -- 3216.8238 3218.9063 3219.0933

Frequencies -- 3220.4256 3220.9074 3221.3885

Frequencies -- 3221.6781 3224.9763 3225.8740

Frequencies -- 3229.5496 3230.3127 3231.1785

SCF Done: E(RM06L-D3/def2-SVP/SMD) = -27126.2950267

Sum of electronic and zero-point Energies= -27125.798179

Sum of electronic and thermal Energies= -27125.732796

Sum of electronic and thermal Free Energies= -27125.912566

SCF Done: E(RM06L-D3/def2-TZVPP/SMD) = -27131.1191617

**P33**

Center Atomic Atomic Coordinates (Angstroms)

Number Number Type X Y Z

---------------------------------------------------------------------

1 83 0 16.530698 0.213786 1.006991

2 83 0 12.779991 0.914187 2.619543

3 83 0 6.842431 -3.186632 4.650632

4 16 0 6.482640 -6.078773 2.340211

5 8 0 7.237392 -4.753350 2.608078

6 6 0 7.560178 -7.349111 2.996404

7 1 0 7.546457 -7.232562 4.085597

8 1 0 8.575374 -7.209013 2.601293

9 1 0 7.171072 -8.338169 2.721876

10 6 0 6.724904 -6.378440 0.590838

11 1 0 6.183407 -5.598694 0.042857

12 1 0 6.298630 -7.356062 0.331009

13 1 0 7.794597 -6.349805 0.346741

14 16 0 9.977500 -3.913139 3.405352

15 8 0 8.885268 -2.803744 3.624569

16 6 0 11.460550 -3.038853 3.868431

17 1 0 11.457296 -2.887149 4.953005

18 1 0 11.451961 -2.070005 3.349534

19 1 0 12.338665 -3.630345 3.579758

20 6 0 10.251751 -3.972304 1.642125

21 1 0 9.369472 -4.432535 1.184171

22 1 0 11.129857 -4.609195 1.469154

23 1 0 10.434056 -2.949452 1.273310

24 16 0 5.638322 -2.739430 1.268484

25 8 0 5.643154 -2.165412 2.711824

26 6 0 7.238003 -2.281490 0.593387

27 1 0 7.977479 -2.583652 1.343651

28 1 0 7.296118 -1.197952 0.431449

29 1 0 7.405180 -2.818025 -0.351065

30 6 0 4.608358 -1.587210 0.367819

31 1 0 3.587934 -1.673143 0.759340

32 1 0 4.613780 -1.868490 -0.693577

33 1 0 4.981728 -0.562541 0.488799

34 16 0 7.504688 -0.085906 3.388890

35 8 0 7.092296 -0.855975 4.705532

36 6 0 8.690780 1.095064 3.990564

37 1 0 9.583842 0.541022 4.318697

38 1 0 8.255313 1.683655 4.809360

39 1 0 8.990915 1.744768 3.156409

40 6 0 6.110247 1.003881 3.125344

41 1 0 5.944702 1.636853 4.006293

42 1 0 5.240476 0.365602 2.932290

43 1 0 6.313235 1.630006 2.245962

44 16 0 9.296767 -1.575416 6.432191

45 8 0 8.115459 -2.607509 6.583243

46 6 0 10.461894 -2.179305 7.654400

47 1 0 10.915752 -3.104123 7.280631

48 1 0 9.936650 -2.367768 8.599907

49 1 0 11.248499 -1.424138 7.778551

50 6 0 8.744573 -0.121210 7.316444

51 1 0 7.868891 0.258775 6.781282

52 1 0 9.560012 0.613985 7.282420

53 1 0 8.484692 -0.379216 8.350836

54 16 0 5.273215 -1.336577 7.183422

55 8 0 5.140382 -2.585426 6.272655

56 6 0 4.492686 -1.860528 8.703969

57 1 0 5.142655 -2.619607 9.154807

58 1 0 3.505871 -2.287516 8.486601

59 1 0 4.408292 -1.004530 9.385705

60 6 0 4.008322 -0.231551 6.568628

61 1 0 4.324862 0.079337 5.565935

62 1 0 3.937061 0.650432 7.218126

63 1 0 3.047648 -0.758794 6.515764

64 16 0 9.035778 -5.323610 6.476368

65 8 0 7.784254 -5.278712 5.560315

66 6 0 9.619472 -7.006633 6.320786

67 1 0 10.007318 -7.133067 5.302537

68 1 0 8.803604 -7.714027 6.515433

69 1 0 10.439813 -7.161331 7.033918

70 6 0 8.388821 -5.392364 8.142307

71 1 0 7.886645 -4.434591 8.321309

72 1 0 9.218281 -5.514425 8.851194

73 1 0 7.678147 -6.223688 8.229359

74 35 0 15.457334 1.723490 3.582122

75 35 0 13.869009 1.679810 0.006282

76 35 0 14.226307 -1.719747 2.005786

77 35 0 16.875241 -1.137705 -1.420506

78 35 0 18.500282 -1.192953 2.404356

79 35 0 18.164247 2.390604 0.340759

80 35 0 11.449269 3.392380 2.996779

81 35 0 12.079716 0.123491 5.437668

82 35 0 10.396603 -0.261583 1.330114

---------------------------------------------------------------------

Frequencies -- 6.1970 14.2752 16.1043

Frequencies -- 19.9662 26.2912 28.7341

Frequencies -- 29.3469 32.3489 36.4902

Frequencies -- 37.7005 42.2244 44.2783

Frequencies -- 45.2803 47.8972 49.0099

Frequencies -- 52.2649 53.5355 55.5544

Frequencies -- 57.4070 58.6961 59.2965

Frequencies -- 61.0008 61.5661 65.2266

Frequencies -- 66.6841 68.1052 68.3885

Frequencies -- 71.4624 72.7382 74.8492

Frequencies -- 76.0577 77.2709 82.3999

Frequencies -- 87.0898 88.3512 95.8914

Frequencies -- 99.6699 103.8496 105.1488

Frequencies -- 108.7163 111.7941 114.4573

Frequencies -- 115.7149 117.1293 121.8511

Frequencies -- 125.9220 127.0205 130.4286

Frequencies -- 132.5315 139.3702 140.0630

Frequencies -- 142.2365 143.6008 144.7917

Frequencies -- 145.5136 150.1347 150.4095

Frequencies -- 156.2240 159.9950 160.9972

Frequencies -- 163.2281 164.1746 166.3496

Frequencies -- 170.5974 175.6992 176.2388

Frequencies -- 181.3602 184.5082 190.5555

Frequencies -- 195.6131 202.7337 207.6638

Frequencies -- 219.8898 225.0206 228.5898

Frequencies -- 234.6714 243.8963 245.5244

Frequencies -- 269.0679 276.2766 277.1541

Frequencies -- 284.2894 302.1611 304.2457

Frequencies -- 310.1797 313.1288 314.8500

Frequencies -- 315.5114 323.4459 328.1724

Frequencies -- 330.5922 333.9567 341.6681

Frequencies -- 343.6387 344.2831 353.6063

Frequencies -- 358.2678 363.3992 371.8861

Frequencies -- 384.8313 393.0910 398.2887

Frequencies -- 403.1244 412.3371 435.4187

Frequencies -- 450.1328 452.1245 689.8007

Frequencies -- 691.8619 693.1316 694.8514

Frequencies -- 697.4718 702.0131 709.5358

Frequencies -- 720.6822 725.8575 727.7816

Frequencies -- 728.2056 732.3611 736.9236

Frequencies -- 740.9336 892.8439 896.0810

Frequencies -- 900.0572 902.7506 903.6910

Frequencies -- 904.0382 906.1977 907.6409

Frequencies -- 911.2326 920.8709 937.1817

Frequencies -- 939.9224 943.2097 945.5509

Frequencies -- 945.7613 946.9615 951.2944

Frequencies -- 956.4030 961.4871 961.9953

Frequencies -- 968.8647 983.3784 984.1052

Frequencies -- 988.8251 996.1617 998.6208

Frequencies -- 1007.1295 1017.2838 1023.5761

Frequencies -- 1024.9462 1026.2236 1030.1589

Frequencies -- 1032.7802 1039.3523 1040.6853

Frequencies -- 1277.2570 1283.1878 1284.9982

Frequencies -- 1290.6877 1292.7358 1297.2809

Frequencies -- 1298.1605 1300.6804 1302.5844

Frequencies -- 1309.7598 1314.9321 1315.0905

Frequencies -- 1316.6866 1317.6703 1365.9635

Frequencies -- 1371.3477 1374.1660 1376.1587

Frequencies -- 1386.4919 1388.4284 1388.5197

Frequencies -- 1390.3851 1394.3502 1396.0320

Frequencies -- 1397.4222 1399.6328 1400.1098

Frequencies -- 1400.6040 1402.9415 1403.4408

Frequencies -- 1404.8272 1406.7510 1409.7915

Frequencies -- 1411.3395 1416.5334 1418.0333

Frequencies -- 1418.8558 1423.0017 1425.9537

Frequencies -- 1431.3925 1436.1476 1437.0942

Frequencies -- 3031.4476 3034.0458 3059.7959

Frequencies -- 3060.0900 3060.1946 3063.0884

Frequencies -- 3063.5673 3064.7342 3066.1078

Frequencies -- 3068.8158 3069.2769 3069.6718

Frequencies -- 3070.4177 3071.1446 3166.3058

Frequencies -- 3182.7528 3199.1128 3206.4394

Frequencies -- 3206.6636 3206.7121 3209.5832

Frequencies -- 3209.9130 3210.3733 3211.4945

Frequencies -- 3211.6539 3213.7611 3215.3152

Frequencies -- 3215.5632 3216.1664 3216.7442

Frequencies -- 3217.3766 3220.6569 3221.2401

Frequencies -- 3221.9609 3222.9041 3224.1038

Frequencies -- 3225.6919 3229.2824 3232.0416

Frequencies -- 3233.6299 3240.2293 3250.9242

SCF Done: E(RM06L-D3/def2-SVP/SMD) = -27679.2368683

Sum of electronic and zero-point Energies= -27678.660947

Sum of electronic and thermal Energies= -27678.587865

Sum of electronic and thermal Free Energies= -27678.787294

SCF Done: E(RM06L-D3/def2-TZVPP/SMD) = -27684.3876309

**P34**

Center Atomic Atomic Coordinates (Angstroms)

Number Number Type X Y Z

---------------------------------------------------------------------

1 83 0 16.814757 0.390732 1.286847

2 83 0 12.992024 0.977883 2.778839

3 83 0 6.917123 -3.282251 4.757418

4 16 0 6.327315 -5.926894 2.234669

5 8 0 7.178311 -4.698319 2.649297

6 6 0 6.760497 -7.235562 3.376458

7 1 0 6.361675 -6.951215 4.355975

8 1 0 7.851277 -7.343692 3.421966

9 1 0 6.289282 -8.166567 3.034011

10 6 0 7.206033 -6.532474 0.793625

11 1 0 7.114708 -5.772464 0.007123

12 1 0 6.739129 -7.463357 0.445866

13 1 0 8.262914 -6.700962 1.039710

14 16 0 10.022228 -3.921097 3.252459

15 8 0 8.979244 -2.839902 3.668109

16 6 0 11.561334 -3.108445 3.647202

17 1 0 11.635652 -3.034612 4.738606

18 1 0 11.532178 -2.102116 3.205380

19 1 0 12.404564 -3.686192 3.247664

20 6 0 10.112818 -3.833688 1.469851

21 1 0 9.165091 -4.217370 1.078588

22 1 0 10.935013 -4.481159 1.136641

23 1 0 10.289579 -2.787896 1.170355

24 16 0 5.173463 -2.819357 1.691382

25 8 0 5.518493 -2.133337 3.038552

26 6 0 6.482543 -2.289146 0.590873

27 1 0 7.410564 -2.688463 1.017183

28 1 0 6.526557 -1.192513 0.557366

29 1 0 6.321566 -2.701349 -0.414177

30 6 0 3.817552 -1.835812 1.064461

31 1 0 2.972837 -1.981234 1.749434

32 1 0 3.537684 -2.198252 0.066528

33 1 0 4.090480 -0.774132 1.025341

34 16 0 7.699548 -0.131291 3.519059

35 8 0 7.204251 -0.849968 4.822843

36 6 0 8.912413 1.016946 4.141539

37 1 0 9.790089 0.438922 4.468570

38 1 0 8.474333 1.585822 4.973388

39 1 0 9.231729 1.692896 3.336353

40 6 0 6.392368 1.039159 3.148991

41 1 0 6.153255 1.643472 4.034327

42 1 0 5.518051 0.464876 2.821435

43 1 0 6.728307 1.688568 2.329289

44 16 0 9.429958 -1.696998 6.552089

45 8 0 8.252615 -2.707523 6.753509

46 6 0 10.645159 -2.274842 7.743000

47 1 0 11.122966 -3.178264 7.345999

48 1 0 10.145463 -2.492438 8.696356

49 1 0 11.411993 -1.499148 7.863309

50 6 0 8.906468 -0.227796 7.433390

51 1 0 8.020784 0.142486 6.906795

52 1 0 9.718534 0.510094 7.383498

53 1 0 8.658503 -0.480123 8.472538

54 16 0 3.530535 -4.310663 4.597022

55 8 0 4.977732 -4.853863 4.530312

56 6 0 2.895238 -4.920971 6.157163

57 1 0 3.521522 -4.470861 6.935052

58 1 0 2.969936 -6.014973 6.190113

59 1 0 1.853803 -4.596898 6.282333

60 6 0 2.629458 -5.396379 3.489953

61 1 0 2.932211 -5.159565 2.462660

62 1 0 1.552560 -5.211362 3.596988

63 1 0 2.867135 -6.443455 3.719297

64 16 0 5.041223 -1.206718 6.929751

65 8 0 5.142300 -2.641343 6.347357

66 6 0 3.795124 -1.378363 8.203740

67 1 0 4.218422 -2.008376 8.994794

68 1 0 2.893202 -1.845629 7.787375

69 1 0 3.560971 -0.388554 8.616593

70 6 0 4.073665 -0.319914 5.710613

71 1 0 4.667088 -0.333943 4.790369

72 1 0 3.913310 0.714591 6.041924

73 1 0 3.116237 -0.832333 5.549584

74 16 0 9.337873 -5.335713 6.255587

75 8 0 7.943706 -5.347696 5.582544

76 6 0 10.001657 -6.944797 5.843867

77 1 0 10.228119 -6.938795 4.770966

78 1 0 9.265166 -7.724446 6.075574

79 1 0 10.930389 -7.108662 6.405759

80 6 0 8.997960 -5.590838 7.993385

81 1 0 8.463196 -4.696488 8.333232

82 1 0 9.940029 -5.699585 8.546843

83 1 0 8.370801 -6.482850 8.114894

84 35 0 15.638296 1.839641 3.834992

85 35 0 14.178046 1.833583 0.214284

86 35 0 14.530721 -1.593021 2.174210

87 35 0 17.264213 -0.958874 -1.134085

88 35 0 18.771430 -0.984015 2.745245

89 35 0 18.447193 2.592377 0.670325

90 35 0 11.622258 3.422243 3.257961

91 35 0 12.270487 -0.027452 5.495463

92 35 0 10.650669 -0.120747 1.360310

---------------------------------------------------------------------

Frequencies -- 11.0053 16.5052 20.8155

Frequencies -- 25.3844 27.8937 29.8681

Frequencies -- 30.3710 36.5061 39.4151

Frequencies -- 40.8484 42.0739 44.4502

Frequencies -- 45.3322 48.7971 50.8481

Frequencies -- 52.9649 53.7457 55.8434

Frequencies -- 57.4343 58.2745 58.5308

Frequencies -- 59.7941 61.3026 63.3655

Frequencies -- 66.1007 68.3114 69.1916

Frequencies -- 70.0835 72.5264 73.1719

Frequencies -- 75.1024 78.5786 81.8519

Frequencies -- 84.0999 87.6429 93.2203

Frequencies -- 94.5428 95.3778 99.6299

Frequencies -- 103.5060 105.8057 108.8978

Frequencies -- 109.6530 112.2501 113.0480

Frequencies -- 114.5946 116.9812 119.2859

Frequencies -- 120.7241 124.3648 125.2922

Frequencies -- 125.9622 128.4443 129.7995

Frequencies -- 131.7622 136.0994 137.6144

Frequencies -- 140.7782 143.0738 144.1436

Frequencies -- 148.9196 149.3454 152.2816

Frequencies -- 154.3656 158.7659 158.9525

Frequencies -- 161.2338 162.8931 166.2312

Frequencies -- 169.0867 169.8206 174.9599

Frequencies -- 176.9252 177.5854 186.7668

Frequencies -- 199.0302 205.5938 209.4033

Frequencies -- 215.2913 227.6620 232.0489

Frequencies -- 235.1656 239.1625 247.5101

Frequencies -- 263.0166 264.8348 265.9822

Frequencies -- 283.4370 285.5618 299.9309

Frequencies -- 302.2109 304.3442 307.2774

Frequencies -- 309.5582 311.1848 314.5011

Frequencies -- 316.1802 320.6329 326.8281

Frequencies -- 328.5159 335.0996 344.2630

Frequencies -- 346.2866 349.9439 352.2834

Frequencies -- 352.7884 359.6896 366.6159

Frequencies -- 373.4833 387.9568 400.8140

Frequencies -- 404.0175 407.1863 409.8887

Frequencies -- 415.7523 421.6176 430.0255

Frequencies -- 443.2774 680.9142 683.7649

Frequencies -- 689.0799 692.5132 694.4520

Frequencies -- 695.3597 695.4921 701.2249

Frequencies -- 714.2000 717.4217 720.9638

Frequencies -- 726.2800 728.3882 731.9170

Frequencies -- 732.0465 734.3016 892.4868

Frequencies -- 899.0847 900.5433 901.4113

Frequencies -- 902.0544 903.2722 907.7167

Frequencies -- 910.9706 924.9582 931.0807

Frequencies -- 936.4218 936.9842 937.7447

Frequencies -- 939.4634 940.6590 941.4559

Frequencies -- 942.5184 947.4512 955.2546

Frequencies -- 957.3147 962.3315 964.9727

Frequencies -- 966.6125 971.4654 985.2706

Frequencies -- 986.0252 988.1229 994.1985

Frequencies -- 997.6922 1000.7404 1006.7894

Frequencies -- 1021.9459 1023.3528 1025.5364

Frequencies -- 1025.9804 1028.9417 1031.6178

Frequencies -- 1034.4443 1037.1305 1044.7533

Frequencies -- 1276.1556 1282.4407 1284.3318

Frequencies -- 1286.6935 1287.4431 1289.7885

Frequencies -- 1290.1031 1298.7572 1302.5695

Frequencies -- 1303.8239 1308.1432 1310.5785

Frequencies -- 1311.4147 1312.2337 1314.5066

Frequencies -- 1316.1661 1376.0421 1383.0761

Frequencies -- 1384.9536 1387.0054 1390.7732

Frequencies -- 1392.6205 1392.9970 1394.8743

Frequencies -- 1395.1130 1395.6720 1396.3567

Frequencies -- 1397.1916 1400.4929 1401.9359

Frequencies -- 1403.2374 1403.9812 1404.5070

Frequencies -- 1405.2196 1407.1140 1408.7266

Frequencies -- 1409.2894 1410.5232 1413.1650

Frequencies -- 1415.4666 1418.0591 1421.3329

Frequencies -- 1423.3870 1428.1631 1429.4365

Frequencies -- 1432.4736 1434.8283 1438.8064

Frequencies -- 3023.3637 3036.2838 3056.1633

Frequencies -- 3056.7444 3060.0746 3060.1805

Frequencies -- 3060.9670 3062.1220 3063.8981

Frequencies -- 3066.3841 3066.4191 3067.9523

Frequencies -- 3068.4137 3069.1642 3069.2692

Frequencies -- 3072.4072 3173.8980 3176.6592

Frequencies -- 3197.1856 3198.9729 3204.2870

Frequencies -- 3207.3768 3208.1295 3208.7607

Frequencies -- 3208.8140 3209.4653 3209.5782

Frequencies -- 3209.9113 3209.9488 3211.7577

Frequencies -- 3212.5862 3213.6848 3215.2232

Frequencies -- 3215.4548 3215.6938 3216.0751

Frequencies -- 3216.7852 3217.3957 3218.5919

Frequencies -- 3220.8757 3223.1698 3223.2728

Frequencies -- 3223.5062 3229.8742 3232.0790

Frequencies -- 3240.9581 3242.3048 3254.6258

SCF Done: E(RM06L-D3/def2-SVP/SMD) = -28232.2036436

Sum of electronic and zero-point Energies= -28231.546254

Sum of electronic and thermal Energies= -28231.466419

Sum of electronic and thermal Free Energies= -28231.677840

SCF Done: E(RM06L-D3/def2-TZVPP/SMD) = -28237.6851334

**P35**

Center Atomic Atomic Coordinates (Angstroms)

Number Number Type X Y Z

---------------------------------------------------------------------

1 83 0 10.901676 1.133061 4.464561

2 83 0 7.597498 -3.775809 4.752263

3 16 0 11.186927 -4.804830 3.774424

4 8 0 9.912820 -4.749863 4.651880

5 6 0 12.117184 -3.317294 4.152154

6 1 0 12.569485 -3.430753 5.144088

7 1 0 11.455257 -2.440954 4.116537

8 1 0 12.917700 -3.201636 3.409771

9 6 0 10.677564 -4.372335 2.107248

10 1 0 9.895057 -5.086002 1.819924

11 1 0 11.539045 -4.501013 1.438519

12 1 0 10.316510 -3.334929 2.078574

13 16 0 7.560766 -3.289527 1.001735

14 8 0 7.011440 -4.040048 2.238220

15 6 0 6.487425 -1.870721 0.768904

16 1 0 6.649805 -1.200986 1.623431

17 1 0 5.438926 -2.188079 0.702306

18 1 0 6.786857 -1.352528 -0.151875

19 6 0 6.988534 -4.284869 -0.378166

20 1 0 7.497319 -5.254044 -0.312108

21 1 0 7.272069 -3.793647 -1.318154

22 1 0 5.901783 -4.428787 -0.331443

23 16 0 9.973759 -2.426952 6.875911

24 8 0 8.594051 -3.192567 6.861628

25 6 0 11.107930 -3.651061 7.539866

26 1 0 11.137117 -4.480493 6.825006

27 1 0 10.752417 -4.003974 8.517469

28 1 0 12.093554 -3.175875 7.633014

29 6 0 9.834437 -1.406567 8.337991

30 1 0 9.212548 -0.533963 8.094579

31 1 0 10.842516 -1.040245 8.570150

32 1 0 9.412510 -2.009403 9.153582

33 16 0 4.522176 -3.149404 3.602186

34 8 0 5.217667 -4.247452 4.446112

35 6 0 3.165277 -2.611366 4.635781

36 1 0 3.615735 -2.080244 5.482294

37 1 0 2.601007 -3.483192 4.989769

38 1 0 2.516033 -1.930734 4.069790

39 6 0 3.608425 -4.090974 2.386380

40 1 0 4.358508 -4.579625 1.753974

41 1 0 2.981119 -3.423528 1.780687

42 1 0 2.994118 -4.848653 2.889217

43 16 0 6.985959 -0.707067 6.085818

44 8 0 6.245691 -2.004442 5.577550

45 6 0 6.153342 -0.401363 7.639432

46 1 0 6.423100 -1.226021 8.311103

47 1 0 5.065916 -0.377107 7.492757

48 1 0 6.520452 0.548439 8.049453

49 6 0 6.251952 0.574608 5.080658

50 1 0 6.702482 0.496608 4.082086

51 1 0 6.521790 1.548493 5.507233

52 1 0 5.164949 0.419647 5.046283

53 16 0 8.351489 -6.014976 7.324386

54 8 0 7.267132 -5.636677 6.281279

55 6 0 8.068026 -7.767658 7.555943

56 1 0 8.369276 -8.272934 6.631006

57 1 0 7.004681 -7.949883 7.754769

58 1 0 8.687887 -8.132361 8.385046

59 6 0 7.705802 -5.423792 8.889183

60 1 0 7.719305 -4.329425 8.832341

61 1 0 8.357609 -5.765832 9.704151

62 1 0 6.681604 -5.791126 9.031868

63 35 0 12.462122 3.394112 4.624091

64 35 0 8.938165 2.300521 2.736090

65 35 0 12.324427 -0.094132 2.290188

66 35 0 9.100693 1.848436 6.811991

67 35 0 12.648037 -0.469967 6.392188

68 35 0 8.870824 -1.488702 3.520112

---------------------------------------------------------------------

Frequencies -- 15.0851 23.7184 28.5378

Frequencies -- 31.1894 40.1161 40.6210

Frequencies -- 44.2350 48.6009 50.9340

Frequencies -- 52.6956 56.1305 58.4179

Frequencies -- 60.4660 62.9586 66.0576

Frequencies -- 69.2578 69.9482 71.7824

Frequencies -- 76.3864 82.2977 88.2504

Frequencies -- 89.8877 90.7002 95.7620

Frequencies -- 96.4052 100.1815 107.0112

Frequencies -- 108.2372 111.7156 113.3716

Frequencies -- 113.9781 115.9921 120.1951

Frequencies -- 123.4238 127.2268 129.0330

Frequencies -- 133.3749 133.9420 138.1359

Frequencies -- 143.6865 144.4602 145.7949

Frequencies -- 148.0427 150.7952 159.5776

Frequencies -- 162.8107 169.4265 173.9966

Frequencies -- 177.8902 181.6825 186.2356

Frequencies -- 199.1811 211.2783 222.0247

Frequencies -- 227.7912 231.7796 233.2278

Frequencies -- 254.9110 264.7799 267.5529

Frequencies -- 268.1317 274.7828 289.9862

Frequencies -- 296.8385 299.4541 302.7930

Frequencies -- 304.1402 310.9587 319.7670

Frequencies -- 323.1481 324.4066 333.8111

Frequencies -- 335.1731 338.7620 343.4709

Frequencies -- 344.4008 349.7769 360.9256

Frequencies -- 371.6152 399.7078 413.7226

Frequencies -- 417.5475 426.7668 436.6222

Frequencies -- 673.3215 680.4030 683.5663

Frequencies -- 686.6857 697.8880 701.0138

Frequencies -- 701.7947 714.7566 721.1117

Frequencies -- 722.3297 731.1551 738.0578

Frequencies -- 893.9588 895.3390 898.6591

Frequencies -- 898.8959 900.0740 904.4235

Frequencies -- 911.3596 915.2225 928.6299

Frequencies -- 929.1514 940.0324 941.4129

Frequencies -- 943.7431 945.6418 949.3456

Frequencies -- 960.1652 961.8289 964.6450

Frequencies -- 975.1598 979.0457 996.2527

Frequencies -- 1003.2061 1005.4637 1019.3835

Frequencies -- 1022.7308 1024.1782 1029.4356

Frequencies -- 1034.3302 1037.5462 1043.0993

Frequencies -- 1258.8108 1274.4910 1281.8965

Frequencies -- 1282.5712 1284.4689 1290.7470

Frequencies -- 1292.1330 1295.9620 1303.7506

Frequencies -- 1308.9644 1311.1136 1314.4561

Frequencies -- 1378.7881 1379.2464 1384.3667

Frequencies -- 1386.5111 1386.6857 1390.9642

Frequencies -- 1394.8122 1395.8547 1396.8928

Frequencies -- 1397.9364 1403.3927 1404.1938

Frequencies -- 1405.0320 1406.6351 1408.0466

Frequencies -- 1411.9298 1412.4624 1415.0808

Frequencies -- 1420.9447 1421.7560 1427.6303

Frequencies -- 1428.4747 1435.3973 1435.5143

Frequencies -- 3047.5058 3051.2655 3051.3712

Frequencies -- 3052.8477 3057.6626 3060.7828

Frequencies -- 3061.8829 3062.4686 3065.6385

Frequencies -- 3065.7935 3066.1418 3068.0077

Frequencies -- 3194.3077 3196.7518 3200.1254

Frequencies -- 3201.5112 3203.9705 3208.4830

Frequencies -- 3208.9839 3209.2136 3210.4393

Frequencies -- 3210.8133 3210.8701 3213.8756

Frequencies -- 3214.0521 3215.0674 3215.2355

Frequencies -- 3217.5480 3218.8405 3221.0560

Frequencies -- 3222.0161 3222.1813 3222.5450

Frequencies -- 3223.1676 3225.6855 3227.8617

SCF Done: E(RM06L-D3/def2-SVP/SMD) = -19190.0813456

Sum of electronic and zero-point Energies= -19189.589744

Sum of electronic and thermal Energies= -19189.532796

Sum of electronic and thermal Free Energies= -19189.689072

SCF Done: E(RM06L-D3/def2-TZVPP/SMD) = -19193.9599502

**P36**

Center Atomic Atomic Coordinates (Angstroms)

Number Number Type X Y Z

---------------------------------------------------------------------

1 83 0 12.832301 0.947271 2.478702

2 83 0 6.797990 -3.232538 4.705156

3 16 0 6.420020 -6.142188 2.386280

4 8 0 7.175099 -4.819972 2.648385

5 6 0 7.499867 -7.415553 3.036897

6 1 0 7.491182 -7.300875 4.126390

7 1 0 8.513166 -7.269723 2.638973

8 1 0 7.112731 -8.404896 2.760549

9 6 0 6.656624 -6.447596 0.635707

10 1 0 6.128734 -5.656325 0.090779

11 1 0 6.216166 -7.418106 0.372956

12 1 0 7.726835 -6.432933 0.392316

13 16 0 9.923892 -3.922314 3.378519

14 8 0 8.793752 -2.856488 3.635558

15 6 0 11.377572 -2.991188 3.814739

16 1 0 11.433380 -2.903599 4.905103

17 1 0 11.284037 -1.995556 3.355163

18 1 0 12.273739 -3.491287 3.425118

19 6 0 10.145680 -3.947819 1.608151

20 1 0 9.264874 -4.432943 1.173127

21 1 0 11.040801 -4.549955 1.401361

22 1 0 10.284378 -2.908707 1.255589

23 16 0 5.595120 -2.793782 1.307885

24 8 0 5.569200 -2.241888 2.758940

25 6 0 7.157429 -2.219169 0.633144

26 1 0 7.925455 -2.469245 1.374755

27 1 0 7.147078 -1.131490 0.486030

28 1 0 7.357942 -2.730793 -0.318541

29 6 0 4.481551 -1.703459 0.427489

30 1 0 3.471169 -1.870857 0.818748

31 1 0 4.504961 -1.964145 -0.638961

32 1 0 4.775694 -0.655655 0.566655

33 16 0 7.530243 -0.195052 3.407982

34 8 0 7.049503 -0.909703 4.741538

35 6 0 8.721588 0.984798 3.990696

36 1 0 9.616960 0.441788 4.335860

37 1 0 8.283281 1.599684 4.788844

38 1 0 9.040811 1.603431 3.137871

39 6 0 6.164054 0.909382 3.061368

40 1 0 5.996806 1.598765 3.899065

41 1 0 5.283434 0.278738 2.895002

42 1 0 6.395339 1.478686 2.150607

43 16 0 9.310719 -1.659961 6.408688

44 8 0 8.105572 -2.666966 6.612881

45 6 0 10.489267 -2.275161 7.613761

46 1 0 10.911475 -3.214619 7.238953

47 1 0 9.981124 -2.439658 8.573061

48 1 0 11.294174 -1.533475 7.695286

49 6 0 8.808252 -0.183990 7.286491

50 1 0 7.931886 0.207799 6.760563

51 1 0 9.646900 0.523285 7.222358

52 1 0 8.559397 -0.427932 8.327171

53 16 0 5.337129 -1.270297 7.201827

54 8 0 5.131631 -2.551624 6.353797

55 6 0 4.578840 -1.690337 8.765956

56 1 0 5.212801 -2.451742 9.235411

57 1 0 3.570429 -2.088744 8.598940

58 1 0 4.546366 -0.800532 9.407819

59 6 0 4.097767 -0.142308 6.573396

60 1 0 4.385445 0.091325 5.541880

61 1 0 4.095619 0.778775 7.170640

62 1 0 3.111343 -0.622019 6.591879

63 16 0 9.067971 -5.362922 6.432523

64 8 0 7.773294 -5.343195 5.581450

65 6 0 9.678713 -7.033513 6.242942

66 1 0 10.026103 -7.144411 5.208319

67 1 0 8.885238 -7.758125 6.465283

68 1 0 10.529509 -7.177539 6.921746

69 6 0 8.507150 -5.449508 8.129658

70 1 0 7.986941 -4.505279 8.328482

71 1 0 9.373522 -5.544656 8.797395

72 1 0 7.825854 -6.300541 8.254342

73 35 0 15.188596 2.076699 3.598347

74 35 0 13.542080 1.819545 -0.113219

75 35 0 14.089404 -1.717379 2.049370

76 35 0 11.222977 3.351336 3.009150

77 35 0 12.023940 0.058045 5.485530

78 35 0 10.219569 -0.310423 1.242439

---------------------------------------------------------------------

Frequencies -- 11.9752 22.5768 26.0462

Frequencies -- 30.4020 34.2559 35.8561

Frequencies -- 38.4600 41.3306 43.0191

Frequencies -- 44.1245 47.3741 50.3626

Frequencies -- 52.0246 53.8687 56.6746

Frequencies -- 58.8540 61.7305 66.3493

Frequencies -- 67.5159 68.8610 71.2470

Frequencies -- 73.9414 74.3269 75.2749

Frequencies -- 80.5663 87.0558 89.4370

Frequencies -- 93.2820 94.7310 97.4068

Frequencies -- 100.8418 107.4029 108.4458

Frequencies -- 113.3751 117.6472 121.4087

Frequencies -- 122.3094 124.6118 126.8023

Frequencies -- 127.9375 136.7503 139.2355

Frequencies -- 142.2733 144.7777 147.2719

Frequencies -- 152.1314 154.5738 157.8739

Frequencies -- 158.4644 160.4181 164.2304

Frequencies -- 169.1987 175.9385 179.2672

Frequencies -- 181.0144 184.6189 191.1176

Frequencies -- 197.2175 219.5666 222.2139

Frequencies -- 229.9011 233.6392 237.5769

Frequencies -- 246.1100 251.8656 252.2959

Frequencies -- 270.7876 273.5726 280.8587

Frequencies -- 289.5011 302.1713 306.3367

Frequencies -- 307.7042 309.7950 311.7046

Frequencies -- 317.5533 320.6563 323.7125

Frequencies -- 324.4887 329.6402 335.3699

Frequencies -- 338.8682 345.5809 349.4393

Frequencies -- 353.1580 358.2076 359.7836

Frequencies -- 381.9539 393.5048 396.7409

Frequencies -- 401.7652 412.3106 431.7951

Frequencies -- 447.7461 462.4875 683.0390

Frequencies -- 687.0126 689.4316 690.8907

Frequencies -- 691.7560 698.4709 706.7835

Frequencies -- 720.0145 721.6159 722.8270

Frequencies -- 725.0162 728.0609 732.1779

Frequencies -- 748.2207 875.6465 883.8774

Frequencies -- 887.4864 888.3607 888.9445

Frequencies -- 899.1064 903.0922 906.5938

Frequencies -- 907.9719 921.8025 932.3803

Frequencies -- 933.8335 939.2853 940.1118

Frequencies -- 941.2008 943.5163 944.8489

Frequencies -- 957.1627 963.4231 965.0034

Frequencies -- 967.5128 974.2496 976.3253

Frequencies -- 979.9616 999.2869 1000.9917

Frequencies -- 1009.8019 1010.8440 1013.6110

Frequencies -- 1019.4118 1021.5374 1028.1955

Frequencies -- 1033.0834 1039.3260 1043.7013

Frequencies -- 1262.4761 1266.6923 1275.4853

Frequencies -- 1276.8962 1283.1480 1289.3712

Frequencies -- 1291.0608 1292.8537 1294.5920

Frequencies -- 1301.4475 1308.5980 1309.8804

Frequencies -- 1311.9552 1313.3762 1359.3911

Frequencies -- 1373.8471 1379.3260 1383.1181

Frequencies -- 1384.3034 1386.3822 1387.6764

Frequencies -- 1389.9730 1392.3131 1393.2777

Frequencies -- 1393.6076 1399.2234 1399.6973

Frequencies -- 1400.4380 1401.2363 1403.3858

Frequencies -- 1403.6286 1404.0415 1406.4528

Frequencies -- 1408.2053 1413.3646 1414.0965

Frequencies -- 1418.7320 1423.3466 1426.9234

Frequencies -- 1429.0763 1433.1926 1438.5567

Frequencies -- 2984.5487 3019.8378 3052.1909

Frequencies -- 3056.5245 3057.3733 3060.0959

Frequencies -- 3060.8330 3062.7843 3063.8121

Frequencies -- 3065.4353 3065.7018 3065.7290

Frequencies -- 3066.8556 3069.7035 3145.9715

Frequencies -- 3160.9314 3189.7660 3195.0311

Frequencies -- 3196.7826 3200.0490 3206.3367

Frequencies -- 3206.7852 3210.4579 3210.5969

Frequencies -- 3211.0885 3211.4495 3213.1057

Frequencies -- 3213.9524 3214.3072 3216.5022

Frequencies -- 3217.4552 3218.6905 3221.0117

Frequencies -- 3221.5102 3225.4352 3225.5767

Frequencies -- 3226.3798 3229.6098 3229.7362

Frequencies -- 3233.3597 3234.3388 3254.9253

SCF Done: E(RM06L-D3/def2-SVP/SMD) = -19743.0037551

Sum of electronic and zero-point Energies= -19742.431273

Sum of electronic and thermal Energies= -19742.367491

Sum of electronic and thermal Free Energies= -19742.540070

SCF Done: E(RM06L-D3/def2-TZVPP/SMD) = -19747.2140429

**P37**

Center Atomic Atomic Coordinates (Angstroms)

Number Number Type X Y Z

---------------------------------------------------------------------

1 83 0 12.833623 1.040103 2.462794

2 83 0 6.872054 -3.311548 4.784145

3 16 0 6.217168 -5.932199 2.315934

4 8 0 7.167820 -4.776166 2.714115

5 6 0 6.621997 -7.289413 3.412132

6 1 0 6.304116 -6.991338 4.417182

7 1 0 7.703599 -7.473757 3.390266

8 1 0 6.068384 -8.181285 3.089153

9 6 0 6.980799 -6.563481 0.821611

10 1 0 6.905494 -5.780367 0.056856

11 1 0 6.437410 -7.453587 0.478488

12 1 0 8.034265 -6.804240 1.013856

13 16 0 10.006512 -3.865606 3.293696

14 8 0 8.881005 -2.839665 3.646518

15 6 0 11.471150 -2.942925 3.710851

16 1 0 11.525720 -2.850201 4.801738

17 1 0 11.381111 -1.946758 3.253306

18 1 0 12.364631 -3.445893 3.320037

19 6 0 10.142868 -3.823670 1.513091

20 1 0 9.245338 -4.306375 1.108888

21 1 0 11.032717 -4.403594 1.232819

22 1 0 10.245330 -2.771388 1.188336

23 16 0 5.546471 -2.740704 1.484678

24 8 0 5.524175 -2.216315 2.945476

25 6 0 7.058816 -2.071095 0.783839

26 1 0 7.868119 -2.341724 1.473711

27 1 0 7.007308 -0.976944 0.709838

28 1 0 7.232698 -2.513746 -0.206749

29 6 0 4.351115 -1.701014 0.650180

30 1 0 3.359172 -1.948924 1.046296

31 1 0 4.377139 -1.922567 -0.424967

32 1 0 4.572066 -0.640890 0.824904

33 16 0 7.651666 -0.197153 3.590829

34 8 0 7.072759 -0.880296 4.889019

35 6 0 8.930374 0.861957 4.214801

36 1 0 9.771046 0.229914 4.542820

37 1 0 8.537836 1.473772 5.038874

38 1 0 9.298902 1.492301 3.393022

39 6 0 6.434662 1.058208 3.193846

40 1 0 6.223906 1.687750 4.068786

41 1 0 5.522917 0.553152 2.853935

42 1 0 6.840940 1.668332 2.375462

43 16 0 9.436793 -1.772504 6.542785

44 8 0 8.262128 -2.786226 6.769611

45 6 0 10.689344 -2.340793 7.695523

46 1 0 11.090979 -3.291120 7.324298

47 1 0 10.250113 -2.476738 8.692680

48 1 0 11.493912 -1.593064 7.692837

49 6 0 8.926249 -0.301349 7.427888

50 1 0 8.033216 0.065414 6.909254

51 1 0 9.745383 0.428297 7.360783

52 1 0 8.690061 -0.554773 8.469704

53 16 0 3.506530 -4.407792 4.791356

54 8 0 4.957356 -4.936553 4.700375

55 6 0 2.921810 -4.988094 6.383068

56 1 0 3.548828 -4.498184 7.135931

57 1 0 3.031159 -6.077950 6.446380

58 1 0 1.873194 -4.693330 6.520824

59 6 0 2.591843 -5.535591 3.740021

60 1 0 2.866180 -5.320626 2.700264

61 1 0 1.515431 -5.363174 3.869482

62 1 0 2.850722 -6.572853 3.990480

63 16 0 4.992300 -1.182637 6.910019

64 8 0 5.119445 -2.641424 6.398732

65 6 0 3.736462 -1.323073 8.180421

66 1 0 4.166272 -1.901649 9.006642

67 1 0 2.852841 -1.834910 7.776951

68 1 0 3.468558 -0.321551 8.541776

69 6 0 4.011314 -0.374912 5.646112

70 1 0 4.590654 -0.467053 4.721227

71 1 0 3.867386 0.683560 5.900387

72 1 0 3.045568 -0.885454 5.540332

73 16 0 9.326636 -5.389371 6.227955

74 8 0 7.921748 -5.388942 5.582018

75 6 0 9.976917 -6.994329 5.776406

76 1 0 10.167365 -6.972916 4.696605

77 1 0 9.246681 -7.776568 6.018796

78 1 0 10.922922 -7.168441 6.305505

79 6 0 9.018924 -5.674023 7.968624

80 1 0 8.498900 -4.779953 8.331717

81 1 0 9.969877 -5.799252 8.503084

82 1 0 8.385726 -6.562136 8.087679

83 35 0 15.205770 2.169532 3.547436

84 35 0 13.512723 1.951040 -0.122472

85 35 0 14.107596 -1.594373 1.919243

86 35 0 11.233715 3.446845 3.030399

87 35 0 12.185004 0.053847 5.506249

88 35 0 10.169833 -0.157807 1.216098

---------------------------------------------------------------------

Frequencies -- 17.7844 19.7340 22.2600

Frequencies -- 26.6190 30.1961 33.9483

Frequencies -- 35.6089 38.3382 42.4663

Frequencies -- 44.4848 48.0023 50.6620

Frequencies -- 52.8777 53.1251 55.1467

Frequencies -- 57.7576 59.0270 61.1418

Frequencies -- 65.1650 65.9828 68.5734

Frequencies -- 69.4913 70.3558 72.5178

Frequencies -- 73.3520 81.1060 84.0054

Frequencies -- 86.6506 88.6806 90.8695

Frequencies -- 96.4535 99.8249 101.6934

Frequencies -- 104.8547 108.1669 111.1805

Frequencies -- 111.4361 115.0824 116.6158

Frequencies -- 120.0211 122.5464 125.7257

Frequencies -- 126.3360 127.8747 131.5395

Frequencies -- 133.4944 136.2877 142.2980

Frequencies -- 143.4919 146.2068 147.5277

Frequencies -- 150.9705 151.8243 155.3393

Frequencies -- 156.0507 157.6082 161.5673

Frequencies -- 167.4806 169.8351 171.4500

Frequencies -- 175.3025 182.8025 185.9761

Frequencies -- 198.7637 204.4818 213.6887

Frequencies -- 219.7018 228.5369 229.1080

Frequencies -- 236.6120 241.2538 258.4340

Frequencies -- 274.0173 274.2054 282.8208

Frequencies -- 284.9076 294.1979 300.5011

Frequencies -- 307.5418 309.5919 312.3806

Frequencies -- 313.8480 316.0280 318.5059

Frequencies -- 321.1815 321.8909 322.0491

Frequencies -- 324.4822 327.6025 344.6488

Frequencies -- 345.7854 348.9367 351.4962

Frequencies -- 355.1943 359.0662 361.9434

Frequencies -- 370.2779 392.6168 399.2012

Frequencies -- 400.7643 404.6101 409.4065

Frequencies -- 413.1233 421.9703 427.1579

Frequencies -- 438.6613 678.8605 685.9363

Frequencies -- 686.7286 687.7820 690.1883

Frequencies -- 694.7161 702.3397 703.6777

Frequencies -- 718.4456 719.4265 719.8140

Frequencies -- 724.1463 725.7531 731.2822

Frequencies -- 731.7995 764.6763 870.7640

Frequencies -- 889.1912 896.4408 897.6141

Frequencies -- 897.9013 899.5355 901.5626

Frequencies -- 904.8814 908.8543 918.8915

Frequencies -- 926.6820 932.2379 936.2516

Frequencies -- 938.5012 939.7297 942.1877

Frequencies -- 944.2352 947.2233 948.4088

Frequencies -- 957.3186 960.2587 963.5019

Frequencies -- 967.0650 971.5910 979.6765

Frequencies -- 982.0282 984.0738 997.2421

Frequencies -- 1001.4861 1004.5701 1006.0834

Frequencies -- 1010.1388 1013.7423 1022.9919

Frequencies -- 1024.1387 1024.7304 1027.7541

Frequencies -- 1030.0354 1034.8439 1044.2384

Frequencies -- 1266.7401 1269.0168 1270.3843

Frequencies -- 1275.9503 1284.2017 1285.3379

Frequencies -- 1286.9752 1289.6213 1292.0245

Frequencies -- 1294.3097 1301.5590 1307.7955

Frequencies -- 1309.3259 1309.4076 1314.7999

Frequencies -- 1318.5245 1370.8329 1378.2302

Frequencies -- 1381.5187 1386.1837 1388.5201

Frequencies -- 1389.3822 1389.8581 1390.9134

Frequencies -- 1392.8905 1396.1672 1398.2925

Frequencies -- 1398.5652 1400.2289 1402.4981

Frequencies -- 1402.5805 1402.8318 1404.3380

Frequencies -- 1404.6713 1406.1651 1407.3666

Frequencies -- 1409.0532 1409.9089 1411.3605

Frequencies -- 1415.6041 1419.5362 1420.4807

Frequencies -- 1424.4062 1425.0191 1431.5236

Frequencies -- 1432.0625 1433.9969 1443.2981

Frequencies -- 2989.7790 3029.1845 3048.7322

Frequencies -- 3054.5847 3057.2808 3059.5128

Frequencies -- 3059.7091 3060.6105 3061.9817

Frequencies -- 3062.9628 3063.5828 3065.6861

Frequencies -- 3066.8368 3066.8795 3067.5142

Frequencies -- 3068.4946 3157.2360 3165.1984

Frequencies -- 3192.5637 3201.0418 3201.5947

Frequencies -- 3201.7765 3202.2792 3202.7190

Frequencies -- 3206.2803 3207.8993 3209.2106

Frequencies -- 3209.2790 3210.2264 3211.6037

Frequencies -- 3211.9352 3211.9580 3213.5946

Frequencies -- 3213.9638 3214.8779 3216.2681

Frequencies -- 3217.2278 3217.9060 3221.3451

Frequencies -- 3222.4195 3222.5425 3222.5644

Frequencies -- 3223.3526 3227.0957 3230.6316

Frequencies -- 3231.1052 3243.6608 3260.9050

SCF Done: E(RM06L-D3/def2-SVP/SMD) = -20295.9683338

Sum of electronic and zero-point Energies= -20295.314167

Sum of electronic and thermal Energies= -20295.243499

Sum of electronic and thermal Free Energies= -20295.430495

SCF Done: E(RM06L-D3/def2-TZVPP/SMD) = -20300.5102006

**P38 {(Me_3_S)_3_[BiBr_6_]}**

Center Atomic Atomic Coordinates (Angstroms)

Number Number Type X Y Z

---------------------------------------------------------------------

1 83 0 4.814113 2.917321 9.286334

2 35 0 6.950358 3.790096 11.033653

3 35 0 2.804195 4.393562 10.922596

4 35 0 4.329121 0.548590 11.080513

5 35 0 2.450888 1.942953 7.620074

6 35 0 6.591695 1.318817 7.610421

7 35 0 5.135616 5.163778 7.476045

8 16 0 4.656378 2.666956 3.832735

9 6 0 3.432978 3.744157 4.570826

10 1 0 3.535512 3.764734 5.665909

11 1 0 2.448678 3.364473 4.271569

12 1 0 3.583798 4.742968 4.142950

13 6 0 4.389151 1.112191 4.676191

14 1 0 4.387051 1.252916 5.767127

15 1 0 5.191980 0.432663 4.365380

16 1 0 3.424834 0.721300 4.329723

17 6 0 6.195437 3.253248 4.532611

18 1 0 6.177883 3.199482 5.631503

19 1 0 6.331400 4.284713 4.186425

20 1 0 6.992589 2.622319 4.120825

21 16 0 4.608867 3.020743 14.751446

22 6 0 5.882195 1.971198 14.058364

23 1 0 5.845272 1.983095 12.958970

24 1 0 6.843852 2.349869 14.425611

25 1 0 5.715760 0.960017 14.449178

26 6 0 4.896941 4.586239 13.934845

27 1 0 4.922755 4.456215 12.843017

28 1 0 4.087355 5.262994 14.233792

29 1 0 5.853601 4.972282 14.306539

30 6 0 3.120350 2.427893 13.952859

31 1 0 3.229306 2.486120 12.859835

32 1 0 2.963051 1.396395 14.290906

33 1 0 2.294093 3.058824 14.303303

34 16 0 0.258473 0.238034 9.562774

35 6 0 0.824424 1.339959 10.852068

36 1 0 -0.028219 1.977009 11.118645

37 1 0 1.625455 1.967126 10.434556

38 1 0 1.177398 0.764457 11.716612

39 6 0 -0.996367 -0.704887 10.440390

40 1 0 -1.323244 -1.525281 9.790553

41 1 0 -1.848110 -0.040299 10.627865

42 1 0 -0.606803 -1.101389 11.386082

43 6 0 1.562245 -0.980805 9.437098

44 1 0 2.479914 -0.447541 9.155833

45 1 0 1.267049 -1.684743 8.649475

46 1 0 1.692831 -1.495363 10.396575

---------------------------------------------------------------------

Frequencies -- 18.6064 25.8498 30.9686

Frequencies -- 43.0544 44.3381 46.8186

Frequencies -- 48.0617 52.9728 56.7162

Frequencies -- 58.9128 60.8216 65.1552

Frequencies -- 69.3230 73.4672 77.7228

Frequencies -- 89.2734 92.2983 99.6617

Frequencies -- 103.5028 105.6783 107.8537

Frequencies -- 108.8273 113.3636 120.2812

Frequencies -- 124.3972 124.4903 127.9048

Frequencies -- 132.5932 133.9648 135.6437

Frequencies -- 138.2332 142.3780 145.1258

Frequencies -- 169.8269 188.8434 221.9281

Frequencies -- 237.9313 240.6627 248.9555

Frequencies -- 256.4627 263.3175 265.4062

Frequencies -- 276.7343 295.2767 299.2808

Frequencies -- 303.0108 312.2070 314.2854

Frequencies -- 332.5759 344.4376 352.3029

Frequencies -- 666.1825 670.0939 671.9671

Frequencies -- 734.9482 741.5039 746.1948

Frequencies -- 749.1507 749.3966 753.4271

Frequencies -- 885.3936 889.5858 891.5423

Frequencies -- 937.9141 938.6888 943.0940

Frequencies -- 946.2265 946.8271 959.1089

Frequencies -- 1031.6986 1046.0514 1048.0810

Frequencies -- 1049.3216 1051.7452 1052.6367

Frequencies -- 1062.5743 1064.2036 1070.0007

Frequencies -- 1306.3799 1313.0113 1313.5574

Frequencies -- 1316.7049 1318.3703 1319.7280

Frequencies -- 1340.4335 1348.7533 1351.6538

Frequencies -- 1361.3802 1373.1351 1379.4615

Frequencies -- 1384.2163 1391.6451 1392.0841

Frequencies -- 1393.8312 1394.9113 1397.4478

Frequencies -- 1397.8379 1398.5831 1404.4717

Frequencies -- 1408.6166 1410.5543 1411.1780

Frequencies -- 1415.4506 1416.0583 1424.5401

Frequencies -- 3043.9254 3047.2613 3047.4754

Frequencies -- 3047.9883 3049.0262 3049.5998

Frequencies -- 3057.3521 3059.2732 3068.8252

Frequencies -- 3198.2709 3198.7287 3200.3414

Frequencies -- 3200.4559 3200.5735 3201.3786

Frequencies -- 3204.5148 3210.4758 3216.7541

Frequencies -- 3216.9565 3218.2473 3218.7797

Frequencies -- 3219.8965 3220.1484 3221.8902

Frequencies -- 3222.5431 3223.3357 3224.4161

SCF Done: E(RM06L-D3/def2-SVP/SMD) = -17210.5492657

Sum of electronic and zero-point Energies= -17210.197262

Sum of electronic and thermal Energies= -17210.159230

Sum of electronic and thermal Free Energies= -17210.276081

SCF Done: E(RM06L-D3/def2-TZVPP/SMD) = -17213.2736517

**(Me_3_S)Br**

Center Atomic Atomic Coordinates (Angstroms)

Number Number Type X Y Z

---------------------------------------------------------------------

1 6 0 4.113827 3.269356 5.468579

2 1 0 4.859407 3.267924 6.272799

3 1 0 3.588482 4.232011 5.464855

4 1 0 4.560674 3.031048 4.481737

5 16 0 2.906282 2.004296 5.861054

6 6 0 1.896465 1.995829 4.379012

7 1 0 1.345167 2.943493 4.351615

8 1 0 2.565216 1.874637 3.502680

9 6 0 3.873799 0.510657 5.645620

10 1 0 4.625022 0.492682 6.444248

11 1 0 3.194881 -0.341403 5.770415

12 1 0 4.335849 0.537250 4.638433

13 1 0 1.181927 1.169306 4.473635

14 35 0 4.826182 1.642527 2.368380

---------------------------------------------------------------------

Frequencies -- 108.6006 122.7229 123.8035

Frequencies -- 237.5145 259.7646 264.8325

Frequencies -- 284.1611 304.8534 325.0340

Frequencies -- 664.3657 727.4044 734.6758

Frequencies -- 901.6271 956.2084 968.6845

Frequencies -- 1056.1568 1062.9626 1078.9868

Frequencies -- 1295.0761 1298.9675 1341.0576

Frequencies -- 1389.2481 1392.1222 1409.0793

Frequencies -- 1414.9186 1424.5564 1431.4761

Frequencies -- 2931.1998 2938.0128 2962.5827

Frequencies -- 3159.5894 3163.4936 3167.7548

Frequencies -- 3220.0418 3226.0011 3227.9616

SCF Done: E(RM06L-D3/def2-SVP/SMD) = -3091.42482427

Sum of electronic and zero-point Energies= -3091.309605

Sum of electronic and thermal Energies= -3091.301356

Sum of electronic and thermal Free Energies= -3091.343371

SCF Done: E(RM06L-D3/def2-TZVPP/SMD) = -3092.02101112

**2. RRHO-corrected energies**

**Supplementary Data Table 1| Absolute RRHO-corrected energies (in Hartrees), relative RRHO-corrected energies (in kcal·mol^-1^), and the logarithm of the binding constant of Bi^3+^ with [EDTA-H]^3-^ at 298 K (1M standard state).**

| **Structures** | **H** | **T.qh-S** | **qh-G(T)** | **ΔH** | **-TΔS** | **ΔG** | **Log_10_ K_eq_** |
| --- | --- | --- | --- | --- | --- | --- | --- |
| **Bi(OH)_3_** | -442.32879 | 0.03730 | -442.36608 |  | | | |
| **EDTA-H_4_** | -1102.05002 | 0.06679 | -1102.11681 |  |  |  |  |
| **[(*κ*^6^-EDTA-H)**  **Bi(OH_2_)]** | -1391.57854 | 0.06943 | -1391.64797 |  |  |  |  |
| **H_2_O** | -76.42872 | 0.01907 | -76.44779 | **-35.9** | **-2.2** | **-38.1** | **27.90** |

**Supplementary Data Table 2| Absolute RRHO-corrected energies (in Hartrees) and relative RRHO-corrected energies (in kcal·mol^-1^) at 298 K (1M standard state) for Entries 1 to 48.**

| **Entry** | **Structures** | **H** | **T.qh-S** | **qh-G(T)** | **ΔH** | **-TΔS** | **ΔG** |
| --- | --- | --- | --- | --- | --- | --- | --- |
| **Bi(III)··· O-Malonate chelated products** | | | | | | | |
| **1** | **Bi_9_O_13_(OH)** | -2987.51507 | 0.09213 | -2987.60720 |  | | |
|  | **Diethyl bromomalonate** | -3148.31212 | 0.05295 | -3148.36507 |  |  |  |
|  | **Bi_5_O_7_(OH)** | -1676.66890 | 0.06329 | -1676.73219 |  |  |  |
|  | **P1** | -1937.62013 | 0.10604 | -1937.72617 |  |  |  |
|  | **P2** | -7937.09854 | 0.04109 | -7937.13963 |  |  |  |
|  | **O_2_** | -150.37427 | 0.02025 | -150.39452 | **24.9** | **-1.3** | **23.6** |
| **2** | **Bi_9_O_13_(OH)** | -2987.51507 | 0.09213 | -2987.60720 |  | | |
|  | **Diethyl bromomalonate** | -3148.31212 | 0.05295 | -3148.36507 |  |  |  |
|  | **Bi_5_O_7_(OH)** | -1676.66890 | 0.06329 | -1676.73219 |  |  |  |
|  | **P1** | -1937.62013 | 0.10604 | -1937.72617 |  |  |  |
|  | **Br_2_** | -5148.11649 | 0.02491 | -5148.14140 |  |  |  |
|  | **O_2_** | -150.37427 | 0.02025 | -150.39452 | **45.4** | **4.7** | **50.1** |
| **3** | **Bi_9_O_13_(OH)** | -2987.51507 | 0.09213 | -2987.60720 |  | | |
|  | **Diethyl Bromomalonate** | -3148.31212 | 0.05295 | -3148.36507 |  |  |  |
|  | **Bi_5_O_7_(OH)** | -1676.66890 | 0.06329 | -1676.73219 |  |  |  |
|  | **P1** | -1937.62013 | 0.10604 | -1937.72617 |  |  |  |
|  | **P3** | -16024.45075 | 0.07217 | -16024.52292 |  |  |  |
|  | **O_2_** | -150.37427 | 0.02025 | -150.39452 | **43.8** | **3.4** | **47.2** |
| **4** | **Bi_9_O_13_(OH)** | -2987.51507 | 0.09213 | -2987.60720 |  | | |
|  | **Diethyl Bromomalonate** | -3148.31212 | 0.05295 | -3148.36507 |  |  |  |
|  | **Bi_5_O_7_(OH)** | -1676.66890 | 0.06329 | -1676.73219 |  |  |  |
|  | **P4** | -3875.28383 | 0.18476 | -3875.46859 |  |  |  |
|  | **P2** | -7937.09854 | 0.04109 | -7937.13963 |  |  |  |
|  | **O_2_** | -150.37427 | 0.02025 | -150.39452 | **18.1** | **3.0** | **21.0** |
| **5** | **Bi_9_O_13_(OH)** | -2987.51507 | 0.09213 | -2987.60720 |  | | |
|  | **Diethyl Bromomalonate** | -3148.31212 | 0.05295 | -3148.36507 |  |  |  |
|  | **Bi_5_O_7_(OH)** | -1676.66890 | 0.06329 | -1676.73219 |  |  |  |
|  | **P4** | -3875.28383 | 0.18476 | -3875.46859 |  |  |  |
|  | **Br_2_** | -5148.11649 | 0.02491 | -5148.14140 |  |  |  |
|  | **O_2_** | -150.37427 | 0.02025 | -150.39452 | **31.7** | **13.3** | **45.0** |
| **6** | **Bi_9_O_13_(OH)** | -2987.51507 | 0.09213 | -2987.60720 |  | | |
|  | **Diethyl Bromomalonate** | -3148.31212 | 0.05295 | -3148.36507 |  |  |  |
|  | **Bi_5_O_7_(OH)** | -1676.66890 | 0.06329 | -1676.73219 |  |  |  |
|  | **P4** | -3875.28383 | 0.18476 | -3875.46859 |  |  |  |
|  | **P3** | -16024.45075 | 0.07217 | -16024.52292 |  |  |  |
|  | **O_2_** | -150.37427 | 0.02025 | -150.39452 | **37.0** | **7.7** | **44.7** |
| **7** | **Bi_9_O_13_(OH)** | -2987.51507 | 0.09213 | -2987.60720 |  | | |
|  | **Diethyl Bromomalonate** | -3148.31212 | 0.05295 | -3148.36507 |  |  |  |
|  | **Bi_5_O_7_(OH)** | -1676.66890 | 0.06329 | -1676.73219 |  |  |  |
|  | **P5** | -5937.26786 | 0.06407 | -5937.33192 |  |  |  |
|  | **P6** | -3937.44631 | 0.08309 | -3937.52939 |  |  |  |
|  | **O_2_** | -150.37427 | 0.02025 | -150.39452 | **26.3** | **-1.3** | **25.0** |
| **8** | **Bi_9_O_13_(OH)** | -2987.51507 | 0.09213 | -2987.60720 |  | | |
|  | **Diethyl Bromomalonate** | -3148.31212 | 0.05295 | -3148.36507 |  |  |  |
|  | **Bi_5_O_7_(OH)** | -1676.66890 | 0.06329 | -1676.73219 |  |  |  |
|  | **P7** | -7874.92023 | 0.14403 | -7875.06425 |  |  |  |
|  | **P8** | -11874.55386 | 0.10461 | -11874.65847 |  |  |  |
|  | **O_2_** | -150.37427 | 0.02025 | -150.39452 | **19.1** | **5.8** | **25.0** |
| **9** | **Bi_9_O_13_(OH)** | -2987.51507 | 0.09213 | -2987.60720 |  | | |
|  | **Diethyl Bromomalonate** | -3148.31212 | 0.05295 | -3148.36507 |  |  |  |
|  | **Bi_5_O_7_(OH)** | -1676.66890 | 0.06329 | -1676.73219 |  |  |  |
|  | **P9** | -1728.64234 | 0.08673 | -1728.72907 |  |  |  |
|  | **Br_2_** | -5148.11649 | 0.02491 | -5148.14140 |  |  |  |
|  | **O_2_** | -150.37427 | 0.02025 | -150.39452 | **31.8** | **-0.5** | **31.3** |
| **10** | **Bi_9_O_13_(OH)** | -2987.51507 | 0.09213 | -2987.60720 |  | | |
|  | **Diethyl Bromomalonate** | -3148.31212 | 0.05295 | -3148.36507 |  |  |  |
|  | **Bi_5_O_7_(OH)** | -1676.66890 | 0.06329 | -1676.73219 |  |  |  |
|  | **P10** | -6801.60770 | 0.09734 | -6801.70505 |  |  |  |
|  | **O_2_** | -150.37427 | 0.02025 | -150.39452 | **20.5** | **0.9** | **21.4** |
| **11** | **Bi_9_O_13_(OH)** | -2987.51507 | 0.09213 | -2987.60720 |  | | |
|  | **Diethyl Bromomalonate** | -3148.31212 | 0.05295 | -3148.36507 |  |  |  |
|  | **Bi_5_O_7_(OH)** | -1676.66890 | 0.06329 | -1676.73219 |  |  |  |
|  | **P11** | -2801.94578 | 0.13793 | -2802.08371 |  |  |  |
|  | **Br_2_** | -5148.11649 | 0.02491 | -5148.14140 |  |  |  |
|  | **O_2_** | -150.37427 | 0.02025 | -150.39452 | **37.2** | **5.7** | **42.9** |
| **12** | **Bi_9_O_13_(OH)** | -2987.51507 | 0.09213 | -2987.60720 |  | | |
|  | **Diethyl Bromomalonate** | -3148.31212 | 0.05295 | -3148.36507 |  |  |  |
|  | **Bi_5_O_7_(OH)** | -1676.66890 | 0.06329 | -1676.73219 |  |  |  |
|  | **P12** | -9658.01635 | 0.11550 | -9658.13184 |  |  |  |
|  | **H_2_O** | -76.42712 | 0.01907 | -76.44619 | **-5.7** | **13.8** | **8.1** |
| **13** | **Bi_9_O_13_(OH)** | -2987.51507 | 0.09213 | -2987.60720 |  | | |
|  | **Diethyl Bromomalonate** | -3148.31212 | 0.05295 | -3148.36507 |  |  |  |
|  | **Bi_5_O_7_(OH)** | -1676.66890 | 0.06329 | -1676.73219 |  |  |  |
|  | **P13** | -19316.07184 | 0.20118 | -19316.27303 |  |  |  |
|  | **H_2_O** | -76.42712 | 0.01907 | -76.44619 | **-18.0** | **23.1** | **5.1** |
| **14** | **Bi_9_O_13_(OH)** | -2987.51507 | 0.09213 | -2987.60720 |  | | |
|  | **Diethyl Bromomalonate** | -3148.31212 | 0.05295 | -3148.36507 |  |  |  |
|  | **Bi_5_O_7_(OH)** | -1676.66890 | 0.06329 | -1676.73219 |  |  |  |
|  | **P14** | -6875.56906 | 0.09451 | -6875.66357 |  |  |  |
|  | **H_2_O** | -76.42712 | 0.01907 | -76.44619 | **16.0** | **2.1** | **18.2** |
| **15** | **Bi_9_O_13_(OH)** | -2987.51507 | 0.09213 | -2987.60720 |  | | |
|  | **Diethyl Bromomalonate** | -3148.31212 | 0.05295 | -3148.36507 |  |  |  |
|  | **Bi_5_O_7_(OH)** | -1676.66890 | 0.06329 | -1676.73219 |  |  |  |
|  | **P15** | -13095.80452 | 0.14923 | -13095.95375 |  |  |  |
|  | **H_2_O** | -76.42712 | 0.01907 | -76.44619 | **4.0** | **12.2** | **16.2** |
| **Bi(III)-Br products** | | | | | | | |
| **16** | **Bi_9_O_13_(OH)** | -2987.51507 | 0.09213 | -2987.60720 |  | | |
|  | **Diethyl Bromomalonate** | -3148.31212 | 0.05295 | -3148.36507 |  |  |  |
|  | **Bi_5_O_7_(OH)** | -1676.66890 | 0.06329 | -1676.73219 |  |  |  |
|  | **P16** | -1148.51836 | 0.07301 | -1148.59137 |  |  |  |
|  | **P2** | -7937.09854 | 0.04109 | -7937.13963 |  |  |  |
|  | **O_2_** | -150.37427 | 0.02025 | -150.39452 | **-5.6** | **0.2** | **-5.4** |
| **17** | **Bi_9_O_13_(OH)** | -2987.51507 | 0.09213 | -2987.60720 |  | | |
|  | **Diethyl Bromomalonate** | -3148.31212 | 0.05295 | -3148.36507 |  |  |  |
|  | **Bi_5_O_7_(OH)** | -1676.66890 | 0.06329 | -1676.73219 |  |  |  |
|  | **P17** | -1147.31330 | 0.07303 | -1147.38633 |  |  |  |
|  | **P2** | -7937.09854 | 0.04109 | -7937.13963 |  |  |  |
|  | **H_2_O** | -76.42712 | 0.01907 | -76.44619 | **-38.4** | **-8.3** | **-46.7** |
| **18** | **Bi_9_O_13_(OH)** | -2987.51507 | 0.09213 | -2987.60720 |  | | |
|  | **Diethyl Bromomalonate** | -3148.31212 | 0.05295 | -3148.36507 |  |  |  |
|  | **Bi_5_O_7_(OH)** | -1676.66890 | 0.06329 | -1676.73219 |  |  |  |
|  | **P18** | -574.85394 | 0.04838 | -574.90232 |  |  |  |
|  | **P2** | -7937.09854 | 0.04109 | -7937.13963 |  |  |  |
|  | **P19** | -648.88661 | 0.05064 | -648.93725 | **-38.6** | **-14.8** | **-53.4** |
| **19** | **Bi_9_O_13_(OH)** | -2987.51507 | 0.09213 | -2987.60720 |  | | |
|  | **Diethyl Bromomalonate** | -3148.31212 | 0.05295 | -3148.36507 |  |  |  |
|  | **Bi_5_O_7_(OH)** | -1676.66890 | 0.06329 | -1676.73219 |  |  |  |
|  | **P16** | -1148.51836 | 0.07301 | -1148.59137 |  |  |  |
|  | **P20** | -15874.21230 | 0.06341 | -15874.27572 |  |  |  |
|  | **O_2_** | -150.37427 | 0.02025 | -150.39452 | **-10.3** | **6.1** | **-4.3** |
| **20** | **Bi_9_O_13_(OH)** | -2987.51507 | 0.09213 | -2987.60720 |  |  |  |
|  | **Diethyl Bromomalonate** | -3148.31212 | 0.05295 | -3148.36507 |  | | |
|  | **Bi_5_O_7_(OH)** | -1676.66890 | 0.06329 | -1676.73219 |  |  |  |
|  | **P17** | -1147.31330 | 0.07303 | -1147.38633 |  |  |  |
|  | **P20** | -15874.21230 | 0.06341 | -15874.27572 |  |  |  |
|  | **H_2_O** | -76.42712 | 0.01907 | -76.44619 | **-43.2** | **-2.4** | **-45.6** |
| **21** | **Bi_9_O_13_(OH)** | -2987.51507 | 0.09213 | -2987.60720 |  | | |
|  | **Diethyl Bromomalonate** | -3148.31212 | 0.05295 | -3148.36507 |  |  |  |
|  | **Bi_5_O_7_(OH)** | -1676.66890 | 0.06329 | -1676.73219 |  |  |  |
|  | **P18** | -574.85394 | 0.04838 | -574.90232 |  |  |  |
|  | **P20** | -15874.21230 | 0.06341 | -15874.27572 |  |  |  |
|  | **P19** | -648.88661 | 0.05064 | -648.93725 | **-43.3** | **-8.9** | **-52.2** |
| **Bi(III)-OBr products** | | | | | | | |
| **22** | **Bi_9_O_13_(OH)** | -2987.51507 | 0.09213 | -2987.60720 |  | | |
|  | **Diethyl Bromomalonate** | -3148.31212 | 0.05295 | -3148.36507 |  |  |  |
|  | **Bi_5_O_7_(OH)** | -1676.66890 | 0.06329 | -1676.73219 |  |  |  |
|  | **P16** | -1148.51836 | 0.07301 | -1148.59137 |  |  |  |
|  | **P2** | -7937.09854 | 0.04109 | -7937.13963 |  |  |  |
|  | **P21** | -8162.59595 | 0.04936 | -8162.64531 | **14.5** | **7.1** | **21.6** |
| **Bi(IV)-Br products** | | | | | | | |
| **23** | **Bi_9_O_13_(OH)** | -2987.51507 | 0.09213 | -2987.60720 |  | | |
|  | **Bromomalonate** | -3148.31212 | 0.05295 | -3148.36507 |  |  |  |
|  | **Bi_5_O_7_(OH)** | -1676.66890 | 0.06329 | -1676.73219 |  |  |  |
|  | **P16** | -1148.51836 | 0.07301 | -1148.59137 |  |  |  |
|  | **P22** | -10511.13899 | 0.04843 | -10511.18742 |  |  |  |
|  | **O_2_** | -150.37427 | 0.02025 | -150.39452 | **2.3** | **5.9** | **8.1** |
| **24** | **Bi_9_O_13_(OH)** | -2987.51507 | 0.09213 | -2987.60720 |  | | |
|  | **Bromomalonate** | -3148.31212 | 0.05295 | -3148.36507 |  |  |  |
|  | **Bi_5_O_7_(OH)** | -1676.66890 | 0.06329 | -1676.73219 |  |  |  |
|  | **P16** | -1148.51836 | 0.07301 | -1148.59137 |  |  |  |
|  | **P17** | -1147.31330 | 0.07303 | -1147.38633 |  |  |  |
|  | **P22** | -10511.13899 | 0.04843 | -10511.18742 |  |  |  |
|  | **H_2_O** | -76.42712 | 0.01907 | -76.44619 | **-30.6** | **-2.6** | **-33.2** |
| **25** | **Bi_9_O_13_(OH)** | -2987.51507 | 0.09213 | -2987.60720 |  |  |  |
|  | **Bromomalonate** | -3148.31212 | 0.05295 | -3148.36507 |  | | |
|  | **Bi_5_O_7_(OH)** | -1676.66890 | 0.06329 | -1676.73219 |  |  |  |
|  | **P16** | -1148.51836 | 0.07301 | -1148.59137 |  |  |  |
|  | **P18** | -574.85394 | 0.04838 | -574.90232 |  |  |  |
|  | **P22** | -10511.13899 | 0.04843 | -10511.18742 |  |  |  |
|  | **P19** | -648.88661 | 0.05064 | -648.93725 | **-30.7** | **-9.1** | **-39.8** |
| **26** | **Bi_9_O_13_(OH)** | -2987.51507 | 0.09213 | -2987.60720 |  | | |
|  | **Bromomalonate** | -3148.31212 | 0.05295 | -3148.36507 |  |  |  |
|  | **Bi_5_O_7_(OH)** | -1676.66890 | 0.06329 | -1676.73219 |  |  |  |
|  | **P16** | -1148.51836 | 0.07301 | -1148.59137 |  |  |  |
|  | **P23** | -21022.28958 | 0.07598 | -21022.36556 |  |  |  |
|  | **O_2_** | -150.37427 | 0.02025 | -150.39452 | **-1.4** | **12.4** | **11.1** |
| **27** | **Bi_9_O_13_(OH)** | -2987.51507 | 0.09213 | -2987.60720 |  | | |
|  | **Bromomalonate** | -3148.31212 | 0.05295 | -3148.36507 |  |  |  |
|  | **Bi_5_O_7_(OH)** | -1676.66890 | 0.06329 | -1676.73219 |  |  |  |
|  | **P16** | -1148.51836 | 0.07301 | -1148.59137 |  |  |  |
|  | **P17** | -1147.31330 | 0.07303 | -1147.38633 |  |  |  |
|  | **P23** | -21022.28958 | 0.07598 | -21022.36556 |  |  |  |
|  | **H_2_O** | -76.42712 | 0.01907 | -76.44619 | **-34.2** | **4.0** | **-30.3** |
| **28** | **Bi_9_O_13_(OH)** | -2987.51507 | 0.09213 | -2987.60720 |  | | |
|  | **Bromomalonate** | -3148.31212 | 0.05295 | -3148.36507 |  |  |  |
|  | **Bi_5_O_7_(OH)** | -1676.66890 | 0.06329 | -1676.73219 |  |  |  |
|  | **P16** | -1148.51836 | 0.07301 | -1148.59137 |  |  |  |
|  | **P18** | -574.85394 | 0.04838 | -574.90232 |  |  |  |
|  | **P23** | -21022.28958 | 0.07598 | -21022.36556 |  |  |  |
|  | **P19** | -648.88661 | 0.05064 | -648.93725 | **-34.4** | **-2.5** | **-36.9** |
| **29** | **Bi_9_O_13_(OH)** | -2987.51507 | 0.09213 | -2987.60720 |  | | |
|  | **Bromomalonate** | -3148.31212 | 0.05295 | -3148.36507 |  |  |  |
|  | **Bi_5_O_7_(OH)** | -1676.66890 | 0.06329 | -1676.73219 |  |  |  |
|  | **P24** | -6511.50481 | 0.09166 | -6511.59647 |  |  |  |
|  | **O_2_** | -150.37427 | 0.02025 | -150.39452 | **31.5** | **3.9** | **35.5** |
| **30** | **Bi_9_O_13_(OH)** | -2987.51507 | 0.09213 | -2987.60720 |  | | |
|  | **Bromomalonate** | -3148.31212 | 0.05295 | -3148.36507 |  |  |  |
|  | **Bi_5_O_7_(OH)** | -1676.66890 | 0.06329 | -1676.73219 |  |  |  |
|  | **P25** | -6511.50500 | 0.09179 | -6511.59679 |  |  |  |
|  | **O_2_** | -150.37427 | 0.02025 | -150.39452 | **31.4** | **3.8** | **35.3** |
| **Bi(V)-Br products** | | | | | | | |
| **31** | **Bi_9_O_13_(OH)** | -2987.51507 | 0.09213 | -2987.60720 |  | | |
|  | **Bromomalonate** | -3148.31212 | 0.05295 | -3148.36507 |  |  |  |
|  | **Bi_5_O_7_(OH)** | -1676.66890 | 0.06329 | -1676.73219 |  |  |  |
|  | **P16** | -1148.51836 | 0.07301 | -1148.59137 |  |  |  |
|  | **P26** | -13085.17841 | 0.05194 | -13085.23036 |  |  |  |
|  | **O_2_** | -150.37427 | 0.02025 | -150.39452 | **10.8** | **14.0** | **24.7** |
| **32** | **Bi_9_O_13_(OH)** | -2987.51507 | 0.09213 | -2987.60720 |  | | |
|  | **Bromomalonate** | -3148.31212 | 0.05295 | -3148.36507 |  |  |  |
|  | **Bi_5_O_7_(OH)** | -1676.66890 | 0.06329 | -1676.73219 |  |  |  |
|  | **P16** | -1148.51836 | 0.07301 | -1148.59137 |  |  |  |
|  | **P17** | -1147.31330 | 0.07303 | -1147.38633 |  |  |  |
|  | **P26** | -13085.17841 | 0.05194 | -13085.23036 |  |  |  |
|  | **H_2_O** | -76.42712 | 0.01907 | -76.44619 | **-22.1** | **5.5** | **-16.6** |
| **33** | **Bi_9_O_13_(OH)** | -2987.51507 | 0.09213 | -2987.60720 |  | | |
|  | **Bromomalonate** | -3148.31212 | 0.05295 | -3148.36507 |  |  |  |
|  | **Bi_5_O_7_(OH)** | -1676.66890 | 0.06329 | -1676.73219 |  |  |  |
|  | **P16** | -1148.51836 | 0.07301 | -1148.59137 |  |  |  |
|  | **P18** | -574.85394 | 0.04838 | -574.90232 |  |  |  |
|  | **P26** | -13085.17841 | 0.05194 | -13085.23036 |  |  |  |
|  | **P19** | -648.88661 | 0.05064 | -648.93725 | **-22.2** | **-1.0** | **-23.2** |
| **34** | **Bi_9_O_13_(OH)** | -2987.51507 | 0.09213 | -2987.60720 |  | | |
|  | **Bromomalonate** | -3148.31212 | 0.05295 | -3148.36507 |  |  |  |
|  | **Bi_5_O_7_(OH)** | -1676.66890 | 0.06329 | -1676.73219 |  |  |  |
|  | **P16** | -1148.51836 | 0.07301 | -1148.59137 |  |  |  |
|  | **P27** | -26170.38785 | 0.08675 | -26170.47460 |  |  |  |
|  | **O_2_** | -150.37427 | 0.02025 | -150.39452 | **1.0** | **19.4** | **20.4** |
| **35** | **Bi_9_O_13_(OH)** | -2987.51507 | 0.09213 | -2987.60720 |  | | |
|  | **Diethyl bromomalonate** | -3148.31212 | 0.05295 | -3148.36507 |  |  |  |
|  | **Bi_5_O_7_(OH)** | -1676.66890 | 0.06329 | -1676.73219 |  |  |  |
|  | **P16** | -1148.51836 | 0.07301 | -1148.59137 |  |  |  |
|  | **P17** | -1147.31330 | 0.07303 | -1147.38633 |  |  |  |
|  | **P27** | -26170.38785 | 0.08675 | -26170.47460 |  |  |  |
|  | **H_2_O** | -76.42712 | 0.01907 | -76.44619 | **-31.9** | **10.9** | **-20.9** |
| **36** | **Bi_9_O_13_(OH)** | -2987.51507 | 0.09213 | -2987.60720 |  | | |
|  | **Bromomalonate** | -3148.31212 | 0.05295 | -3148.36507 |  |  |  |
|  | **Bi_5_O_7_(OH)** | -1676.66890 | 0.06329 | -1676.73219 |  |  |  |
|  | **P16** | -1148.51836 | 0.07301 | -1148.59137 |  |  |  |
|  | **P18** | -574.85394 | 0.04838 | -574.90232 |  |  |  |
|  | **P27** | -26170.38785 | 0.08675 | -26170.47460 |  |  |  |
|  | **P19** | -648.88661 | 0.05064 | -648.93725 | **-32.0** | **4.4** | **-27.6** |
| **Bi(II)-Br products** | | | | | | | |
| **37** | **Bi_9_O_13_(OH)** | -2987.51507 | 0.09213 | -2987.60720 |  | | |
|  | **Diethyl bromomalonate** | -3148.31212 | 0.05295 | -3148.36507 |  |  |  |
|  | **Bi_5_O_7_(OH)** | -1676.66890 | 0.06329 | -1676.73219 |  |  |  |
|  | **P16** | -1148.51836 | 0.07301 | -1148.59137 |  |  |  |
|  | **P28** | -5362.97310 | 0.03640 | -5363.00950 |  |  |  |
|  | **O_2_** | -150.37427 | 0.02025 | -150.39452 | **39.9** | **-7.2** | **32.7** |
| **38** | **Bi_9_O_13_(OH)** | -2987.51507 | 0.09213 | -2987.60720 |  | | |
|  | **Bromomalonate** | -3148.31212 | 0.05295 | -3148.36507 |  |  |  |
|  | **Bi_5_O_7_(OH)** | -1676.66890 | 0.06329 | -1676.73219 |  |  |  |
|  | **O_2_** | -150.37427 | 0.02025 | -150.39452 |  |  |  |
|  | **P17** | -1147.31330 | 0.07303 | -1147.38633 |  |  |  |
|  | **P28** | -5362.97310 | 0.03640 | -5363.00950 |  |  |  |
|  | **H_2_O** | -76.42712 | 0.01907 | -76.44619 | **18.0** | **-12.8** | **5.2** |
| **39** | **Bi_9_O_13_(OH)** | -2987.51507 | 0.09213 | -2987.60720 |  | | |
|  | **Diethyl bromomalonate** | -3148.31212 | 0.05295 | -3148.36507 |  |  |  |
|  | **Bi_5_O_7_(OH)** | -1676.66890 | 0.06329 | -1676.73219 |  |  |  |
|  | **O_2_** | -150.37427 | 0.02025 | -150.39452 |  |  |  |
|  | **P18** | -574.85394 | 0.04838 | -574.90232 |  |  |  |
|  | **P28** | -5362.97310 | 0.03640 | -5363.00950 |  |  |  |
|  | **P19** | -648.88661 | 0.05064 | -648.93725 | **17.9** | **-17.2** | **0.7** |
| **40** | **Bi_9_O_13_(OH)** | -2987.51507 | 0.09213 | -2987.60720 |  | | |
|  | **Bromomalonate** | -3148.31212 | 0.05295 | -3148.36507 |  |  |  |
|  | **Bi_5_O_7_(OH)** | -1676.66890 | 0.06329 | -1676.73219 |  |  |  |
|  | **P16** | -1148.51836 | 0.07301 | -1148.59137 |  |  |  |
|  | **P29** | -10726.00213 | 0.05316 | -10726.05529 |  |  |  |
|  | **O_2_** | -150.37427 | 0.02025 | -150.39452 | **22.4** | **-1.0** | **21.3** |
| **41** | **Bi_9_O_13_(OH)** | -2987.51507 | 0.09213 | -2987.60720 |  | | |
|  | **Diethyl bromomalonate** | -3148.31212 | 0.05295 | -3148.36507 |  |  |  |
|  | **Bi_5_O_7_(OH)** | -1676.66890 | 0.06329 | -1676.73219 |  |  |  |
|  | **O_2_** | -150.37427 | 0.02025 | -150.39452 |  |  |  |
|  | **P17** | -1147.31330 | 0.07303 | -1147.38633 |  |  |  |
|  | **P29** | -10726.00213 | 0.05316 | -10726.05529 |  |  |  |
|  | **H_2_O** | -76.42712 | 0.01907 | -76.44619 | **0.5** | **-6.7** | **-6.2** |
| **42** | **Bi_9_O_13_(OH)** | -2987.51507 | 0.09213 | -2987.60720 |  | | |
|  | **Diethyl bromomalonate** | -3148.31212 | 0.05295 | -3148.36507 |  |  |  |
|  | **Bi_5_O_7_(OH)** | -1676.66890 | 0.06329 | -1676.73219 |  |  |  |
|  | **O_2_** | -150.37427 | 0.02025 | -150.39452 |  |  |  |
|  | **P18** | -574.85394 | 0.04838 | -574.90232 |  |  |  |
|  | **P29** | -10726.00213 | 0.05316 | -10726.05529 |  |  |  |
|  | **P19** | -648.88661 | 0.05064 | -648.93725 | **0.4** | **-11.0** | **-10.6** |
| **43** | **Bi_9_O_13_(OH)** | -2987.51507 | 0.09213 | -2987.60720 |  | | |
|  | **Bromomalonate** | -3148.31212 | 0.05295 | -3148.36507 |  |  |  |
|  | **Bi_5_O_7_(OH)** | -1676.66890 | 0.06329 | -1676.73219 |  |  |  |
|  | **P16** | -1148.51836 | 0.07301 | -1148.59137 |  |  |  |
|  | **P28** | -5362.97310 | 0.03640 | -5363.00950 |  |  |  |
|  | **Br_2_** | -5148.11649 | 0.02491 | -5148.14140 |  |  |  |
|  | **O_2_** | -150.37427 | 0.02025 | -150.39452 | **36.6** | **-4.7** | **31.9** |
| **44** | **Bi_9_O_13_(OH)** | -2987.51507 | 0.09213 | -2987.60720 |  | | |
|  | **Diethyl bromomalonate** | -3148.31212 | 0.05295 | -3148.36507 |  |  |  |
|  | **Bi_5_O_7_(OH)** | -1676.66890 | 0.06329 | -1676.73219 |  |  |  |
|  | **P17** | -1147.31330 | 0.07303 | -1147.38633 |  |  |  |
|  | **P28** | -5362.97310 | 0.03640 | -5363.00950 |  |  |  |
|  | **Br_2_** | -5148.11649 | 0.02491 | -5148.14140 |  |  |  |
|  | **H_2_O** | -76.42712 | 0.01907 | -76.44619 | **3.7** | **-13.1** | **-9.4** |
| **45** | **Bi_9_O_13_(OH)** | -2987.51507 | 0.09213 | -2987.60720 |  | | |
|  | **Bromomalonate** | -3148.31212 | 0.05295 | -3148.36507 |  |  |  |
|  | **Bi_5_O_7_(OH)** | -1676.66890 | 0.06329 | -1676.73219 |  |  |  |
|  | **P18** | -574.85394 | 0.04838 | -574.90232 |  |  |  |
|  | **P28** | -5362.97310 | 0.03640 | -5363.00950 |  |  |  |
|  | **Br_2_** | -5148.11649 | 0.02491 | -5148.14140 |  |  |  |
|  | **P19** | -648.88661 | 0.05064 | -648.93725 | **3.6** | **-19.7** | **-16.1** |
| **46** | **Bi_9_O_13_(OH)** | -2987.51507 | 0.09213 | -2987.60720 |  | | |
|  | **Diethyl bromomalonate** | -3148.31212 | 0.05295 | -3148.36507 |  |  |  |
|  | **Bi_5_O_7_(OH)** | -1676.66890 | 0.06329 | -1676.73219 |  |  |  |
|  | **P16** | -1148.51836 | 0.07301 | -1148.59137 |  |  |  |
|  | **P29** | -10726.00213 | 0.05316 | -10726.05529 |  |  |  |
|  | **Br_2_** | -5148.11649 | 0.02491 | -5148.14140 |  |  |  |
|  | **O_2_** | -150.37427 | 0.02025 | -150.39452 | **19.0** | **1.5** | **20.5** |
| **47** | **Bi_9_O_13_(OH)** | -2987.51507 | 0.09213 | -2987.60720 |  | | |
|  | **Diethyl bromomalonate** | -3148.31212 | 0.05295 | -3148.36507 |  |  |  |
|  | **Bi_5_O_7_(OH)** | -1676.66890 | 0.06329 | -1676.73219 |  |  |  |
|  | **P17** | -1147.31330 | 0.07303 | -1147.38633 |  |  |  |
|  | **P29** | -10726.00213 | 0.05316 | -10726.05529 |  |  |  |
|  | **Br_2_** | -5148.11649 | 0.02491 | -5148.14140 |  |  |  |
|  | **H_2_O** | -76.42712 | 0.01907 | -76.44619 | **-13.8** | **-7.0** | **-20.8** |
| **48** | **Bi_9_O_13_(OH)** | -2987.51507 | 0.09213 | -2987.60720 |  | | |
|  | **Diethyl bromomalonate** | -3148.31212 | 0.05295 | -3148.36507 |  |  |  |
|  | **Bi_5_O_7_(OH)** | -1676.66890 | 0.06329 | -1676.73219 |  |  |  |
|  | **P18** | -574.85394 | 0.04838 | -574.90232 |  |  |  |
|  | **P29** | -10726.00213 | 0.05316 | -10726.05529 |  |  |  |
|  | **Br_2_** | -5148.11649 | 0.02491 | -5148.14140 |  |  |  |
|  | **P19** | -648.88661 | 0.05064 | -648.93725 | **-13.9** | **-13.5** | **-27.4** |

**Supplementary Data Table 3| Absolute RRHO-corrected energies (in Hartrees) and relative RRHO-corrected energies (in kcal·mol^-1^) at 298 K (1M standard state) for Entries 49 to 72.**

| **Entry** | **Structures** | **H** | **T.qh-S** | **qh-G(T)** | **ΔH** | **-TΔS** | **ΔG** |
| --- | --- | --- | --- | --- | --- | --- | --- |
| **49** | **Bi_9_O_13_(OH)** | -2987.51507 | 0.09213 | -2987.60720 |  | | |
|  | **Diethyl bromomalonate** | -3148.31212 | 0.05295 | -3148.36507 |  |  |  |
|  | **DMSO** | -553.17979 | 0.03166 | -553.21145 |  |  |  |
|  | **Bi_5_O_7_(OH)** | -1676.66890 | 0.06329 | -1676.73219 |  |  |  |
|  | **P16** | -1148.51836 | 0.07301 | -1148.59137 |  |  |  |
|  | **P30** | -9596.69951 | 0.08445 | -9596.78397 |  |  |  |
|  | **O_2_** | -150.37427 | 0.02025 | -150.39452 | **-44.2** | **32.5** | **-11.7** |
| **50** | **Bi_9_O_13_(OH)** | -2987.51507 | 0.09213 | -2987.60720 |  | | |
|  | **Diethyl bromomalonate** | -3148.31212 | 0.05295 | -3148.36507 |  |  |  |
|  | **DMSO** | -553.17979 | 0.03166 | -553.21145 |  |  |  |
|  | **Bi_5_O_7_(OH)** | -1676.66890 | 0.06329 | -1676.73219 |  |  |  |
|  | **P17** | -1147.31330 | 0.07303 | -1147.38633 |  |  |  |
|  | **P30** | -9596.69951 | 0.08445 | -9596.78397 |  |  |  |
|  | **H_2_O** | -76.42712 | 0.01907 | -76.44619 | **-77.1** | **24.1** | **-53.0** |
| **51** | **Bi_9_O_13_(OH)** | -2987.51507 | 0.09213 | -2987.60720 |  | | |
|  | **Diethyl bromomalonate** | -3148.31212 | 0.05295 | -3148.36507 |  |  |  |
|  | **DMSO** | -553.17979 | 0.03166 | -553.21145 |  |  |  |
|  | **Bi_5_O_7_(OH)** | -1676.66890 | 0.06329 | -1676.73219 |  |  |  |
|  | **P18** | -574.85394 | 0.04838 | -574.90232 |  |  |  |
|  | **P30** | -9596.69951 | 0.08445 | -9596.78397 |  |  |  |
|  | **P19** | -648.88661 | 0.05064 | -648.93725 | **-77.2** | **17.6** | **-59.6** |
| **52** | **Bi_9_O_13_(OH)** | -2987.51507 | 0.09213 | -2987.60720 |  | | |
|  | **Diethyl bromomalonate** | -3148.31212 | 0.05295 | -3148.36507 |  |  |  |
|  | **DMSO** | -553.17979 | 0.03166 | -553.21145 |  |  |  |
|  | **Bi_5_O_7_(OH)** | -1676.66890 | 0.06329 | -1676.73219 |  |  |  |
|  | **P16** | -1148.51836 | 0.07301 | -1148.59137 |  |  |  |
|  | **P31** | -18087.03500 | 0.11707 | -18087.15206 |  |  |  |
|  | **O_2_** | -150.37427 | 0.02025 | -150.39452 | **-42.8** | **28.9** | **-13.9** |
| **53** | **Bi_9_O_13_(OH)** | -2987.51507 | 0.09213 | -2987.60720 |  | | |
|  | **Diethyl bromomalonate** | -3148.31212 | 0.05295 | -3148.36507 |  |  |  |
|  | **DMSO** | -553.17979 | 0.03166 | -553.21145 |  |  |  |
|  | **Bi_5_O_7_(OH)** | -1676.66890 | 0.06329 | -1676.73219 |  |  |  |
|  | **P17** | -1147.31330 | 0.07303 | -1147.38633 |  |  |  |
|  | **P31** | -18087.03500 | 0.11707 | -18087.15206 |  |  |  |
|  | **H_2_O** | -76.42712 | 0.01907 | -76.44619 | **-75.7** | **20.5** | **-55.2** |
| **54** | **Bi_9_O_13_(OH)** | -2987.51507 | 0.09213 | -2987.60720 |  | | |
|  | **Diethyl bromomalonate** | -3148.31212 | 0.05295 | -3148.36507 |  |  |  |
|  | **DMSO** | -553.17979 | 0.03166 | -553.21145 |  |  |  |
|  | **Bi_5_O_7_(OH)** | -1676.66890 | 0.06329 | -1676.73219 |  |  |  |
|  | **P18** | -574.85394 | 0.04838 | -574.90232 |  |  |  |
|  | **P31** | -18087.03500 | 0.11707 | -18087.15206 |  |  |  |
|  | **P19** | -648.88661 | 0.05064 | -648.93725 | **-75.8** | **14.0** | **-61.8** |
| **55** | **Bi_9_O_13_(OH)** | -2987.51507 | 0.09213 | -2987.60720 |  | | |
|  | **Diethyl bromomalonate** | -3148.31212 | 0.05295 | -3148.36507 |  |  |  |
|  | **DMSO** | -553.17979 | 0.03166 | -553.21145 |  |  |  |
|  | **Bi_5_O_7_(OH)** | -1676.66890 | 0.06329 | -1676.73219 |  |  |  |
|  | **P16** | -1148.51836 | 0.07301 | -1148.59137 |  |  |  |
|  | **P30** | -9596.69951 | 0.08445 | -9596.78397 |  |  |  |
|  | **P32** | -27130.55599 | 0.15924 | -27130.71523 |  |  |  |
|  | **O_2_** | -150.37427 | 0.02025 | -150.39452 | **-43.7** | **32.4** | **-11.3** |
| **56** | **Bi_9_O_13_(OH)** | -2987.51507 | 0.09213 | -2987.60720 |  | | |
|  | **Diethyl bromomalonate** | -3148.31212 | 0.05295 | -3148.36507 |  |  |  |
|  | **DMSO** | -553.17979 | 0.03166 | -553.21145 |  |  |  |
|  | **Bi_5_O_7_(OH)** | -1676.66890 | 0.06329 | -1676.73219 |  |  |  |
|  | **P17** | -1147.31330 | 0.07303 | -1147.38633 |  |  |  |
|  | **P30** | -9596.69951 | 0.08445 | -9596.78397 |  |  |  |
|  | **P32** | -27130.55599 | 0.15924 | -27130.71523 |  |  |  |
|  | **H_2_O** | -76.42712 | 0.01907 | -76.44619 | **-76.6** | **24.0** | **-52.6** |
| **57** | **Bi_9_O_13_(OH)** | -2987.51507 | 0.09213 | -2987.60720 |  | | |
|  | **Diethyl bromomalonate** | -3148.31212 | 0.05295 | -3148.36507 |  |  |  |
|  | **DMSO** | -553.17979 | 0.03166 | -553.21145 |  |  |  |
|  | **Bi_5_O_7_(OH)** | -1676.66890 | 0.06329 | -1676.73219 |  |  |  |
|  | **P18** | -574.85394 | 0.04838 | -574.90232 |  |  |  |
|  | **P30** | -9596.69951 | 0.08445 | -9596.78397 |  |  |  |
|  | **P32** | -27130.55599 | 0.15924 | -27130.71523 |  |  |  |
|  | **P19** | -648.88661 | 0.05064 | -648.93725 | **-76.7** | **17.5** | **-59.3** |
| **58** | **Bi_9_O_13_(OH)** | -2987.51507 | 0.09213 | -2987.60720 |  | | |
|  | **Diethyl bromomalonate** | -3148.31212 | 0.05295 | -3148.36507 |  |  |  |
|  | **DMSO** | -553.17979 | 0.03166 | -553.21145 |  |  |  |
|  | **Bi_5_O_7_(OH)** | -1676.66890 | 0.06329 | -1676.73219 |  |  |  |
|  | **P16** | -1148.51836 | 0.07301 | -1148.59137 |  |  |  |
|  | **P30** | -9596.69951 | 0.08445 | -9596.78397 |  |  |  |
|  | **P33** | -27683.73768 | 0.17525 | -27683.91294 |  |  |  |
|  | **O_2_** | -150.37427 | 0.02025 | -150.39452 | **-44.0** | **34.9** | **-9.2** |
| **59** | **Bi_9_O_13_(OH)** | -2987.51507 | 0.09213 | -2987.60720 |  | | |
|  | **Diethyl bromomalonate** | -3148.31212 | 0.05295 | -3148.36507 |  |  |  |
|  | **DMSO** | -553.17979 | 0.03166 | -553.21145 |  |  |  |
|  | **Bi_5_O_7_(OH)** | -1676.66890 | 0.06329 | -1676.73219 |  |  |  |
|  | **P17** | -1147.31330 | 0.07303 | -1147.38633 |  |  |  |
|  | **P30** | -9596.69951 | 0.08445 | -9596.78397 |  |  |  |
|  | **P33** | -27683.73768 | 0.17525 | -27683.91294 |  |  |  |
|  | **H_2_O** | -76.42712 | 0.01907 | -76.44619 | **-76.9** | **26.4** | **-50.5** |
| **60** | **Bi_9_O_13_(OH)** | -2987.51507 | 0.09213 | -2987.60720 |  | | |
|  | **Diethyl bromomalonate** | -3148.31212 | 0.05295 | -3148.36507 |  |  |  |
|  | **DMSO** | -553.17979 | 0.03166 | -553.21145 |  |  |  |
|  | **Bi_5_O_7_(OH)** | -1676.66890 | 0.06329 | -1676.73219 |  |  |  |
|  | **P18** | -574.85394 | 0.04838 | -574.90232 |  |  |  |
|  | **P30** | -9596.69951 | 0.08445 | -9596.78397 |  |  |  |
|  | **P33** | -27683.73768 | 0.17525 | -27683.91294 |  |  |  |
|  | **P19** | -648.88661 | 0.05064 | -648.93725 | **-77.0** | **19.9** | **-57.1** |
| **61** | **Bi_9_O_13_(OH)** | -2987.51507 | 0.09213 | -2987.60720 |  | | |
|  | **Diethyl bromomalonate** | -3148.31212 | 0.05295 | -3148.36507 |  |  |  |
|  | **DMSO** | -553.17979 | 0.03166 | -553.21145 |  |  |  |
|  | **Bi_5_O_7_(OH)** | -1676.66890 | 0.06329 | -1676.73219 |  |  |  |
|  | **P16** | -1148.51836 | 0.07301 | -1148.59137 |  |  |  |
|  | **P30** | -9596.69951 | 0.08445 | -9596.78397 |  |  |  |
|  | **P34** | -28236.94696 | 0.18733 | -28237.13429 |  |  |  |
|  | **O_2_** | -150.37427 | 0.02025 | -150.39452 | **-48.7** | **37.9** | **-10.7** |
| **62** | **Bi_9_O_13_(OH)** | -2987.51507 | 0.09213 | -2987.60720 |  | | |
|  | **Diethyl bromomalonate** | -3148.31212 | 0.05295 | -3148.36507 |  |  |  |
|  | **DMSO** | -553.17979 | 0.03166 | -553.21145 |  |  |  |
|  | **Bi_5_O_7_(OH)** | -1676.66890 | 0.06329 | -1676.73219 |  |  |  |
|  | **P17** | -1147.31330 | 0.07303 | -1147.38633 |  |  |  |
|  | **P30** | -9596.69951 | 0.08445 | -9596.78397 |  |  |  |
|  | **P34** | -28236.94696 | 0.18733 | -28237.13429 |  |  |  |
|  | **H_2_O** | -76.42712 | 0.01907 | -76.44619 | **-81.5** | **29.5** | **-52.0** |
| **63** | **Bi_9_O_13_(OH)** | -2987.51507 | 0.09213 | -2987.60720 |  | | |
|  | **Diethyl bromomalonate** | -3148.31212 | 0.05295 | -3148.36507 |  |  |  |
|  | **DMSO** | -553.17979 | 0.03166 | -553.21145 |  |  |  |
|  | **Bi_5_O_7_(OH)** | -1676.66890 | 0.06329 | -1676.73219 |  |  |  |
|  | **P18** | -574.85394 | 0.04838 | -574.90232 |  |  |  |
|  | **P30** | -9596.69951 | 0.08445 | -9596.78397 |  |  |  |
|  | **P34** | -28236.94696 | 0.18733 | -28237.13429 |  |  |  |
|  | **P19** | -648.88661 | 0.05064 | -648.93725 | **-81.6** | **23.0** | **-58.7** |
| **64** | **Bi_9_O_13_(OH)** | -2987.51507 | 0.09213 | -2987.60720 |  | | |
|  | **Diethyl bromomalonate** | -3148.31212 | 0.05295 | -3148.36507 |  |  |  |
|  | **DMSO** | -553.17979 | 0.03166 | -553.21145 |  |  |  |
|  | **Bi_5_O_7_(OH)** | -1676.66890 | 0.06329 | -1676.73219 |  |  |  |
|  | **P16** | -1148.51836 | 0.07301 | -1148.59137 |  |  |  |
|  | **P35** | -19193.41046 | 0.14077 | -19193.55123 |  |  |  |
|  | **O_2_** | -150.37427 | 0.02025 | -150.39452 | **-47.8** | **41.4** | **-6.4** |
| **65** | **Bi_9_O_13_(OH)** | -2987.51507 | 0.09213 | -2987.60720 |  | | |
|  | **Diethyl bromomalonate** | -3148.31212 | 0.05295 | -3148.36507 |  |  |  |
|  | **DMSO** | -553.17979 | 0.03166 | -553.21145 |  |  |  |
|  | **Bi_5_O_7_(OH)** | -1676.66890 | 0.06329 | -1676.73219 |  |  |  |
|  | **P17** | -1147.31330 | 0.07303 | -1147.38633 |  |  |  |
|  | **P35** | -19193.41046 | 0.14077 | -19193.55123 |  |  |  |
|  | **H_2_O** | -76.42712 | 0.01907 | -76.44619 | **-80.7** | **32.9** | **-47.8** |
| **66** | **Bi_9_O_13_(OH)** | -2987.51507 | 0.09213 | -2987.60720 |  | | |
|  | **Diethyl bromomalonate** | -3148.31212 | 0.05295 | -3148.36507 |  |  |  |
|  | **DMSO** | -553.17979 | 0.03166 | -553.21145 |  |  |  |
|  | **Bi_5_O_7_(OH)** | -1676.66890 | 0.06329 | -1676.73219 |  |  |  |
|  | **P18** | -574.85394 | 0.04838 | -574.90232 |  |  |  |
|  | **P35** | -19193.41046 | 0.14077 | -19193.55123 |  |  |  |
|  | **P19** | -648.88661 | 0.05064 | -648.93725 | **-80.8** | **26.4** | **-54.4** |
| **67** | **Bi_9_O_13_(OH)** | -2987.51507 | 0.09213 | -2987.60720 |  | | |
|  | **Diethyl bromomalonate** | -3148.31212 | 0.05295 | -3148.36507 |  |  |  |
|  | **DMSO** | -553.17979 | 0.03166 | -553.21145 |  |  |  |
|  | **Bi_5_O_7_(OH)** | -1676.66890 | 0.06329 | -1676.73219 |  |  |  |
|  | **P16** | -1148.51836 | 0.07301 | -1148.59137 |  |  |  |
|  | **P36** | -19746.57684 | 0.15406 | -19746.73089 |  |  |  |
|  | **O_2_** | -150.37427 | 0.02025 | -150.39452 | **-43.6** | **47.1** | **3.5** |
| **68** | **Bi_9_O_13_(OH)** | -2987.51507 | 0.09213 | -2987.60720 |  | | |
|  | **Diethyl bromomalonate** | -3148.31212 | 0.05295 | -3148.36507 |  |  |  |
|  | **DMSO** | -553.17979 | 0.03166 | -553.21145 |  |  |  |
|  | **Bi_5_O_7_(OH)** | -1676.66890 | 0.06329 | -1676.73219 |  |  |  |
|  | **P17** | -1147.31330 | 0.07303 | -1147.38633 |  |  |  |
|  | **P36** | -19746.57684 | 0.15406 | -19746.73089 |  |  |  |
|  | **H_2_O** | -76.42712 | 0.01907 | -76.44619 | **-76.5** | **38.7** | **-37.8** |
| **69** | **Bi_9_O_13_(OH)** | -2987.51507 | 0.09213 | -2987.60720 |  | | |
|  | **Diethyl bromomalonate** | -3148.31212 | 0.05295 | -3148.36507 |  |  |  |
|  | **DMSO** | -553.17979 | 0.03166 | -553.21145 |  |  |  |
|  | **Bi_5_O_7_(OH)** | -1676.66890 | 0.06329 | -1676.73219 |  |  |  |
|  | **P18** | -574.85394 | 0.04838 | -574.90232 |  |  |  |
|  | **P36** | -19746.57684 | 0.15406 | -19746.73089 |  |  |  |
|  | **P19** | -648.88661 | 0.05064 | -648.93725 | **-76.6** | **32.2** | **-44.4** |
| **70** | **Bi_9_O_13_(OH)** | -2987.51507 | 0.09213 | -2987.60720 |  | | |
|  | **Diethyl bromomalonate** | -3148.31212 | 0.05295 | -3148.36507 |  |  |  |
|  | **DMSO** | -553.17979 | 0.03166 | -553.21145 |  |  |  |
|  | **Bi_5_O_7_(OH)** | -1676.66890 | 0.06329 | -1676.73219 |  |  |  |
|  | **P16** | -1148.51836 | 0.07301 | -1148.59137 |  |  |  |
|  | **P37** | -20299.78442 | 0.16717 | -20299.95159 |  |  |  |
|  | **O_2_** | -150.37427 | 0.02025 | -150.39452 | **-52.3** | **53.0** | **0.6** |
| **71** | **Bi_9_O_13_(OH)** | -2987.51507 | 0.09213 | -2987.60720 |  | | |
|  | **Diethyl bromomalonate** | -3148.31212 | 0.05295 | -3148.36507 |  |  |  |
|  | **DMSO** | -553.17979 | 0.03166 | -553.21145 |  |  |  |
|  | **Bi_5_O_7_(OH)** | -1676.66890 | 0.06329 | -1676.73219 |  |  |  |
|  | **P17** | -1147.31330 | 0.07303 | -1147.38633 |  |  |  |
|  | **P37** | -20299.78442 | 0.16717 | -20299.95159 |  |  |  |
|  | **H_2_O** | -76.42712 | 0.01907 | -76.44619 | **-85.2** | **44.5** | **-40.7** |
| **72** | **Bi_9_O_13_(OH)** | -2987.51507 | 0.09213 | -2987.60720 |  | | |
|  | **Diethyl bromomalonate** | -3148.31212 | 0.05295 | -3148.36507 |  |  |  |
|  | **DMSO** | -553.17979 | 0.03166 | -553.21145 |  |  |  |
|  | **Bi_5_O_7_(OH)** | -1676.66890 | 0.06329 | -1676.73219 |  |  |  |
|  | **P18** | -574.85394 | 0.04838 | -574.90232 |  |  |  |
|  | **P37** | -20299.78442 | 0.16717 | -20299.95159 |  |  |  |
|  | **P19** | -648.88661 | 0.05064 | -648.93725 | **-85.3** | **38.0** | **-47.3** |

**Supplementary Data Table 4| Absolute RRHO-corrected energies (in Hartrees) and relative RRHO-corrected energies (in kcal·mol^-1^) at 298 K (1M standard state) for P38, {(Me_3_S)_3_[BiBr_6_]}.**

| **Structures** | **H** | **T.qh-S** | **qh-G(T)** | **ΔH** | | |
| --- | --- | --- | --- | --- | --- | --- |
| **(Me_3_S)Br** | -3091.89660 | 0.03974 | -3091.93633 |  | | |
| **P2** | -7937.09854 | 0.04109 | -7937.13963 |  |  |  |
| **P38** | -17212.88267 | 0.10468 | -17212.98735 | **-59.2** | **34.9** | **-24.3** |
